# Psychological inoculation improves resilience to and reduces willingness to share vaccine misinformation

**DOI:** 10.1038/s41598-025-09462-5

**Published:** 2025-08-18

**Authors:** Ruth E. Appel, Jon Roozenbeek, Rebecca Rayburn-Reeves, Melisa Basol, Jonathan Corbin, Josh Compton, Sander van der Linden

**Affiliations:** 1 Department of Communication, Stanford University, Stanford, USA; 2 Department of War Studies, King’s College London, London, UK; 3 Department of Psychology, University of Cambridge, Cambridge, UK; 4 Center for Advanced Hindsight, Duke University, Durham, USA; 5 Speech at Dartmouth, Dartmouth College, Hanover, USA

**Keywords:** Misinformation, Vaccine, Gamification, Inoculation theory, Technique recognition, Human behaviour, Health policy

## Abstract

Vaccine misinformation endangers public health by contributing to reduced vaccine uptake. We developed a short online game to reduce people’s susceptibility to vaccine misinformation. Building on inoculation theory, the *Bad Vaxx* game exposes people to weakened doses of manipulation techniques commonly used in vaccine misinformation and to strategies to identify these techniques. Across three preregistered randomized controlled trials (*N* = 2,326), we find that the game significantly improves participants’ ability to discern vaccine misinformation from non-misinformation, their confidence in their ability to do so, and the quality of their sharing decisions. Further, taking the perspective of a character *fighting* as opposed to *spreading* misinformation is more effective on some outcome measures. In line with the learning goals of the intervention, we show that participants improve their ability to correctly identify the use of specific misinformation techniques. This insight is important because teaching manipulation technique recognition is not only effective to help evaluate information about vaccines, but also more viable than trying to debunk myriads of constantly-evolving myths. Our findings suggest that a short, low-cost, gamified intervention can increase resilience to vaccine misinformation.

**Supplementary Information:**

The online version contains supplementary material available at 10.1038/s41598-025-09462-5.

## Introduction

Vaccine misinformation can have adverse consequences for public health^1^ at a significant cost to society^2^. A high prevalence of vaccine misinformation can contribute to lower immunization rates^3,4^ and more severe disease outbreaks^4^. Estimates suggest that misleading content about vaccines reduced vaccination rates in the US during the COVID-19 pandemic by more than 2%^5^. Susceptibility to misinformation about COVID-19 predicts lower compliance with public health regulations and lower willingness to get vaccinated^6,7^. Misinformation about COVID-19 has been estimated to have cost between $50 and $300 million each day in the US alone during the pandemic^2^. Despite large-scale efforts to inform the public^8^, the problem of vaccine misinformation persists, and scalable solutions are urgently needed^9,10^.

Inoculation theory^11–13^ has been proposed as a promising method for conferring broad-scale resistance against many forms of online misinformation. Psychological inoculation posits that exposure to a weakened form of a deceptive attack, much like exposure to a weakened version of a pathogen, protects against future exposure to persuasive misinformation^14^. A conventional inoculation message includes two components: (1) a *forewarning* that misleading arguments aiming to change people’s attitudes will be encountered and that they are vulnerable to these challenges; and (2) a *preemptive refutation* – or prebunking – of such arguments^15^. In recent years, researchers have explored ways to inoculate people more generally against manipulation techniques that are commonly used to mislead people instead of only inoculating against individual claims or myths^16,17^. This so-called *technique-based* inoculation approach has yielded several practical interventions such as the *Bad News*^18^, *Go Viral!*^19^, and *Cranky Uncle*^20^ games, which improved people’s ability to recognize manipulation techniques common in general and climate change-specific misinformation , respectively (for meta-analyses and reviews of inoculation interventions, see^13,16,21^). See Supplementary Information (SI) Section *Inoculation Theory* for a more detailed discussion of inoculation theory and its applications in misinformation research.

However, thus far there have been few inoculation interventions—especially scalable ones—that specifically tackle vaccine misinformation^22^. Further, the precise mechanisms underlying effective game-based inoculation interventions still remain unclear, leaving uncertainty about why an intervention works. For example, the perspective the player takes in the game (e.g., being tasked with *spreading* misinformation or, conversely, *fighting* misinformation) may moderate the effectiveness of game-based inoculation interventions. Existing interventions usually only allow for a single perspective For example, in the *Bad News* game^18^, players always take on an “evil” perspective. It is possible that taking on the role of an “evil” actor makes the intervention experience more effective because it may make people feel slightly threatened and uncomfortable about their in-game actions, thus increasing motivation to defend themselves from future manipulation, a key mechanism behind resistance to persuasion^23^. Conversely, taking on the role of a “good” actor tasked with fighting misinformation and reducing its influence might also be effective, for example if people enjoy this perspective more, or find it more natural, and therefore pay more attention to the game’s content. Furthermore, it is unclear what exactly participants learn in inoculation interventions—do they learn to distinguish true from false information, or do they learn to recognize specific manipulation techniques? It is essential to understand what participants learn to evaluate whether an intervention works as intended, and to inform whether educational interventions should focus on teaching techniques or facts.

To address these gaps, we developed the *Bad Vaxx* game, a gamified inoculation intervention designed to counter vaccine misinformation by teaching participants how to recognize specific manipulation techniques. We evaluate the game’s effectiveness, explore whether taking on a specific perspective in the game drives its efficacy, and test what exactly participants learn.

**Fig. 1:**
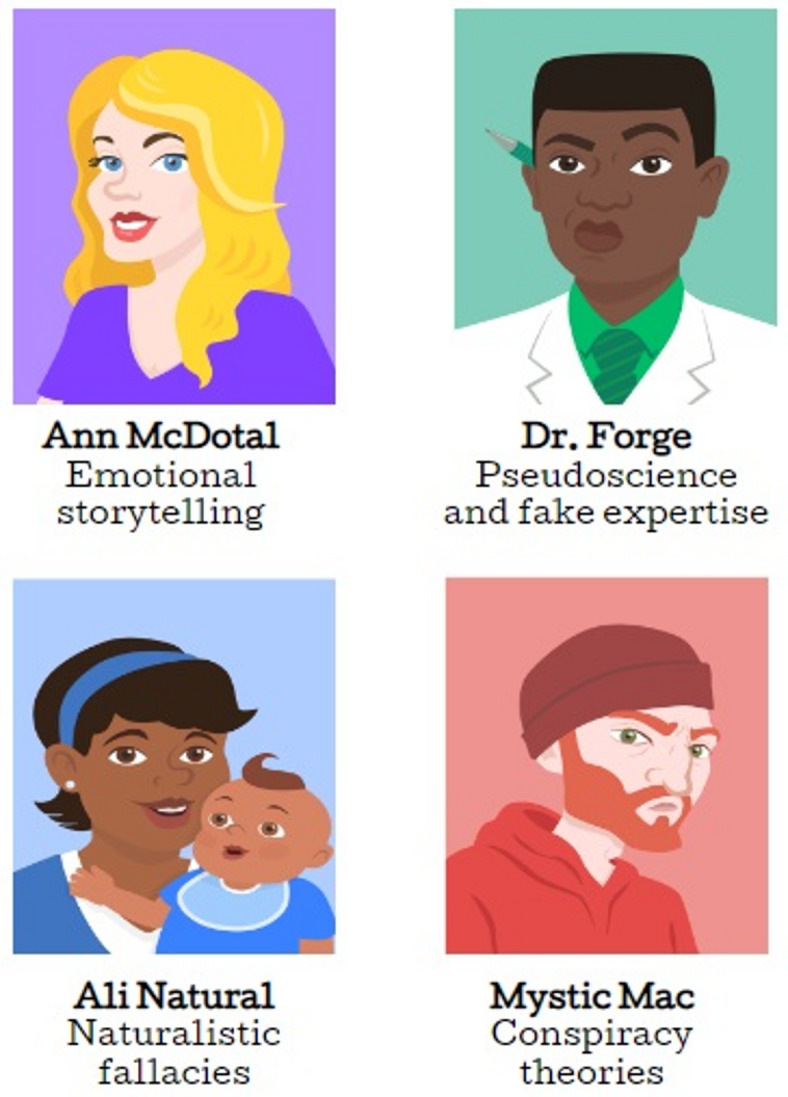
Game characters: The four characters that represent the misinformation addressed strategies throughout the game.

**Fig. 2:**
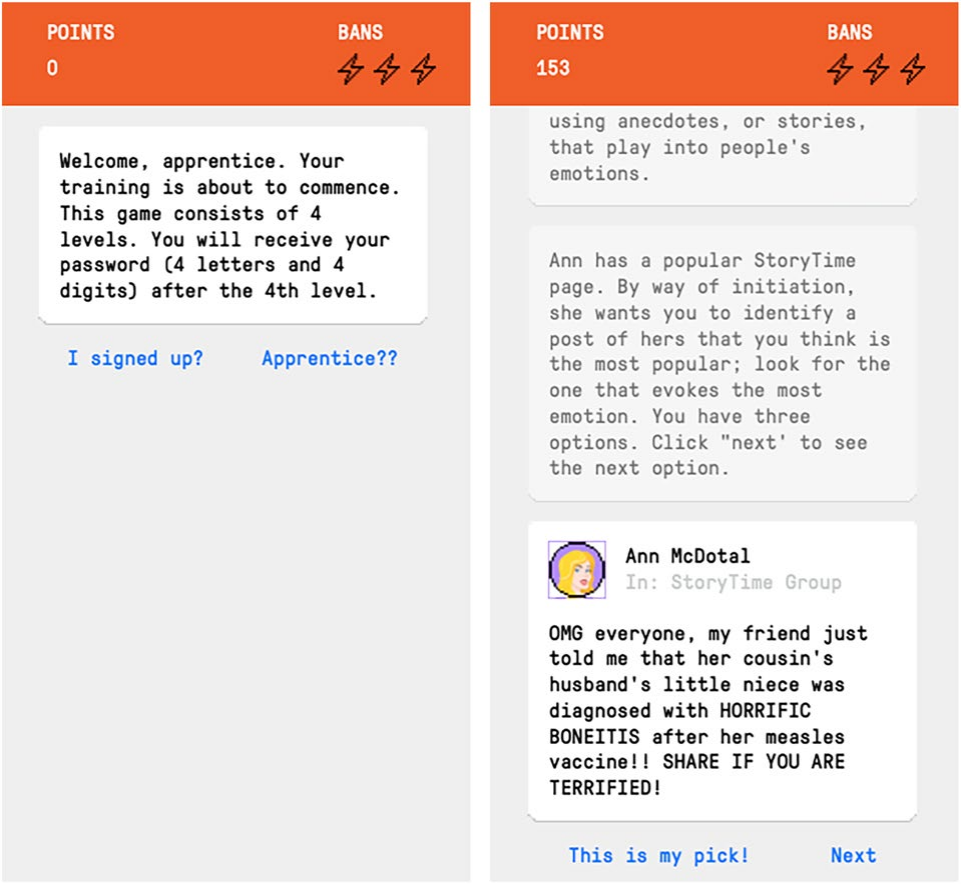
Game user interface: User interface of the *Bad Vaxx* game used for the experiments.

The *Bad Vaxx *game is a method of inoculating people against vaccine misinformation online that is scalable, entertaining, and economical—especially given that prevention is better than cure when it comes to vaccine misinformation^22^—, and even entertaining. Building on the *Bad News* game^18,24^, which inoculates players against misinformation by forewarning and exposing them to weakened doses of six manipulation techniques, we developed the *Bad Vaxx* game to inoculate players against vaccine misinformation (see Fig. 2 for screenshots of the game). The *Bad Vaxx* game has the potential for adoption at scale: Similar interventions like the *Bad News* game received significant engagement after public release, with about a million people being reached by the intervention within two years of its public release based purely on interest and not incentives^25^. The game is also relatively inexpensive to host online.

We focus on vaccine misinformation that is manipulative, i.e. uses manipulation techniques in order to persuade. The game trains people to spot four manipulation techniques, which previous studies have identified as being commonly used in the area of vaccine misinformation^26–28^: (1) emotional storytelling, (2) fake expertise and pseudoscience, (3) the naturalistic fallacy, and (4) conspiracy theories. Each technique is represented by an archetypal character (see Fig. 1): Ann McDotal (the emotional storyteller), Dr. Forge (the fake expert), Ali Natural (the naturalist), and Mystic Mac (the conspiracy theorist). For a detailed discussion about each of these manipulation techniques and their use in vaccine misinformation, see SI Section *Identifying Vaccine Misinformation Techniques*.

We created two versions of the game, which have the same content on manipulation techniques but differ in their perspective-taking. In the “good” version, the goal is to defeat the four characters, who are spreading misinformation about vaccines, and reduce their influence. In the “evil” version , players join the four characters as their apprentice, and help them spread vaccine misinformation. The final production version of the game, which was refined by game designers, is available at https://www.badvaxx.com/. 

The inoculation aspect of the game is embedded in the sequencing of events. Throughout the game, players are (1) forewarned about the tricks that manipulators use and (2) subsequently exposed to weakened doses of these misleading arguments (e.g., naturalistic fallacy) by actively engaging and creating their own content. As opposed to traditional “passive” inoculation where people are typically given specific (weakened) examples of myths and refutations of these myths^12,13,19,29^, with active inoculation players generate their own resistance from the start, for example through experiential learning in a simulated social media setting^13,19,30,31^. As such, the entire game experience constitutes the inoculation treatment and the full dose “attack” comes at the end of the game where players are confronted with a battery of persuasive items (in the form of social media posts) that either contain or do not contain the relevant manipulation technique. While it is possible that people have come across vaccine misinformation before in the wild, research has shown that so-called “therapeutic” (versus purely “prophylactic”) inoculation still boosts immunity regardless of prior exposure^32^. The control group receives no inoculation, but is exposed to the same “attacking” material (i.e., the vaccine misinformation/social media item rating task). This allows us to test whether the inoculation game induces resistance to vaccine misinformation.

The present study thus addresses (1) whether the *Bad Vaxx* game confers psychological resistance against vaccine misinformation (in the form of increasing people’s ability to identify manipulative social media content, increasing their confidence in doing so, and improving the quality of their sharing decisions), (2) whether the effectiveness of the game differs if players take a “good” versus an “evil” perspective, and (3) whether the game improves people’s ability to recognize specific manipulation techniques (exploratory).

We conducted three separate studies (total *N* = 2,326) testing the effectiveness of the *Bad Vaxx* game and the mechanisms underlying it. We find that the two different versions of the game significantly improve participants’ ability to discern manipulative from non-manipulative information about vaccines, increase their confidence in their ability to recognize manipulative information, and improve the quality of their social media sharing decisions. We find that perspective-taking is no decisive mechanism for the effectiveness of the game, although the “good” version outperforms the “evil” version across most outcomes. In Study 3, we find that the game boosts correct manipulation technique recognition. Importantly, study 3 also shows that the intervention induces skepticism in a targeted manner (as opposed to in general) in that participants rate manipulative and false content as significantly more manipulative, but this is *not* the case for non-manipulative and true content. For our preregistered hypotheses, see *Methods*. With the exception of disentangling the ratings for true versus false and manipulative versus non-manipulative content in Study 3, all analyses were pre-registered as detailed in the *Methods* Section.

## Study overview

Study 1 was a proof of concept aimed at testing the validity of our measures and ensuring a good participant experience with the game running smoothly. We recruited 690 US participants via Prolific Academic in November 2020. Median age was between 25 and 34, and 49.5% of the sample was male (48.5% female, 2% other; excluding missing values).

Study 2 aimed to replicate the findings from Study 1. Based on feedback from Study 1, we made slight modifications to the game’s content in order to improve its flow and clarity. We recruited 557 US participants on Prolific Academic in January 2021. Median age was between 25 and 34, and 42.2% of the sample was male (55.1% female, 2.7% other; excluding missing values).

Study 3 aimed to test the robustness of the effects of our intervention with a larger, representative sample given that the results of Study 2 did not fully align with those of Study 1. In addition, we sought to identify further mechanisms behind our findings from Studies 1 and 2, namely whether improved resilience to misinformation is due to an improved ability to distinguish true from false information about vaccinations, due to better recognition of whether a social media post is manipulative, or because people have learned to recognize the specific manipulation techniques taught in the game. We recruited 1,079 participants representative of the US population on Prolific Academic in October 2021. Median age was between 25 and 34, and 48.5% of the sample was male (49.3% female, 2.2% other; excluding missing values).

All experimental protocols were approved by the ethics review boards at the authors’ institutions. All information necessary to reproduce our results (including data, cleaning and analysis scripts, and item sets) are available on OSF (https://osf.io/rdh2b/). We present details of our experimental design in the *Methods* section.

## Results

We present the results of ANOVAs for Studies 1 to 3 individually, as well as the results of an internal network meta-analysis of the results of Studies 1 to 3. The individual study results highlight important nuance in the findings since not all versions of the game yielded the expected results for all outcomes. The internal meta-analysis allows for synthesized results across multiple studies and for more precise effect estimates than individual studies^33^. Our internal meta-analysis meets key validity criteria in that it consists of pre-registered studies that were all conducted following the pre-registered protocol^34^, and we do not selectively include studies. However, we note that some scholars argue that only internal meta-analyses pre-registered prior to running all studies should be considered valid^34^, and ours was pre-registered prior to running Study 3. The SI contains more detailed results, including the results of pairwise meta- analyses (see SI Section *Extended Data Tables and Figures*), and different linear models for each study on a study- or item-level (see SI Section *Detailed Results*).

### Intervention improves discernment between manipulative and non-manipulative social media content about vaccines

A one-way analysis of variance (see Table 1) shows that the effect of the *Bad Vaxx* game on ratings of misinformation items is significant (Study 1: *F* (2*,* 455*.*98) = 7*.*18*, p* < 0*.*001*, η*^2^ = 0*.*02; Study 2: *F* (2*,* 367*.*58) = 5*.*18*, p* = 0*.*006*, η*^2^ = 0*.*02; Study 3: *F* (2*,* 717*.*03) = 15*.*05*, p* < 0*.*001*, η*^2^ = 0*.*03). Pairwise comparisons (see Table 2) using the Games-Howell post hoc criterion for significance indicate that participants in the “good” condition (Study 1: *M* = 5*.*72*, SD* = 1*.*03; Study 2: *M* = 5*.*79*, SD* = 0*.*99; Study 3: *M* = 5*.*71*, SD* = 1*.*02) of the game rate misinformation as significantly more manipulative than participants in the control condition in Studies 1, 2 and 3 (Study 1: *M* = 5*.*32*, SD* = 1*.*21; *M*_*diff*_ = 0*.*4*, p* < 0*.*001; Study 2: *M* = 5*.*44*, SD* = 1*.*25; *M*_*diff*_ = 0*.*35*, p* = 0*.*008; Study 3: *M* = 5*.*35*, SD* = 1*.*2; *M*_*diff*_ = 0*.*36*, p* < 0*.*001). In Studies 2 and 3, participants in the “evil” condition (Study 2: *M* = 5*.*76*, SD* = 1*.*18; Study 3: *M* = 5*.*78*, SD* = 1*.*03) are significantly better at detecting manipulative headlines than participants in the control condition (Study 2: *M*_*diff*_ = 0*.*32*, p* = 0*.*027; Study 3: *M*_*diff*_ = 0*.*43*, p* < 0*.*001). Pairwise comparisons between the “evil” and the “good” condition are nonsignificant in all studies. Note that we did not preregister **H1a** for Study 2.

**Table 1:** One-Way ANOVA (Welch) for Studies 1, 2, and 3.

	Study 1				Study 2				Study 3			
Variable	F	df1	df2	p	F	df1	df2	p	F	df1	df2	p
Misinformation Manipulativeness	7.18	2	455.98	** < 0.001**	5.18	2	367.58	**0.006**	15.05	2	717.03	** < 0.001**
Non-Misinformation Manipulativeness	0.54	2	457.98	0.583	1.43	2	368.94	0.241	4.51	2	713.98	**0.011**
Manipulativeness Discernment	5.26	2	457.59	**0.006**	3.21	2	368.26	**0.042**	11.02	2	713.40	** < 0.001**
Misinformation Confidence	1.90	2	457.88	0.152	2.64	2	368.87	0.073	5.42	2	715.37	**0.005**
Non-Misinformation Confidence	0.48	2	456.53	0.620	1.36	2	366.05	0.258	0.65	2	713.03	0.522
Misinformation Sharing Intent	2.21	2	457.23	0.110	1.72	2	369.24	0.180	1.55	2	707.72	0.214
Non-Misinformation Sharing Intent	2.66	2	457.64	0.071	0.48	2	368.45	0.621	10.62	2	714.34	** < 0.001**
Sharing Intent Discernment	10.06	2	456.16	** < 0.001**	0.76	2	365.82	0.467	15.97	2	706.02	** < 0.001**

Bold *p*-values indicate statistical significance at the 5% significance level.

**Table 2:** Mean Differences and Cohen’s *d* for Studies 1, 2, and 3.

	Study 1				Study 2				Study 3			
Variable	Mean_*diff*_	95% CI	*p*	Cohen’s *d*	Mean_*diff*_	95% CI	*p*	Cohen’s *d*	Mean_*diff*_	95% CI	*p*	Cohen’s *d*
**Good-Control**												
Misinformation Manipulativeness	0.398	[0.149, 0.647]	** < 0.001**	0.354	0.350	[0.078, 0.623]	**0.008**	0.310	0.361	[0.168, 0.554]	** < 0.001**	0.323
Non-Misinformation Manipulativeness	-0.095	[-0.358, 0.168]	0.673	-0.080	-0.057	[-0.321, 0.207]	0.867	-0.053	-0.140	[-0.321, 0.042]	0.167	-0.133
Manipulativeness Discernment	0.493	[0.129, 0.857]	**0.004**	0.300	0.407	[0.028, 0.786]	**0.032**	0.261	0.501	[0.248, 0.754]	** < 0.001**	0.342
Misinformation Confidence	0.193	[-0.040, 0.426]	0.127	0.183	0.178	[-0.059, 0.416]	0.182	0.182	0.211	[0.039, 0.384]	**0.011**	0.212
Non-Misinformation Confidence	0.081	[-0.155, 0.317]	0.700	0.076	-0.071	[-0.311, 0.169]	0.766	-0.072	0.068	[-0.113, 0.249]	0.649	0.065
Misinformation Sharing Intent	-0.264	[-0.607, 0.078]	0.166	-0.171	-0.228	[-0.536, 0.079]	0.189	-0.180	-0.091	[-0.305, 0.123]	0.578	-0.074
Non-Misinformation Sharing Intent	0.327	[-0.013, 0.668]	0.063	0.213	-0.046	[-0.413, 0.322]	0.954	-0.030	0.494	[0.240, 0.748]	** < 0.001**	0.338
Sharing Intent Discernment	0.592	[0.271, 0.912]	** < 0.001**	0.409	0.183	[-0.173, 0.539]	0.450	0.126	0.585	[0.341, 0.828]	** < 0.001**	0.419
**Evil-Control**												
Misinformation Manipulativeness	0.190	[-0.068, 0.448]	0.195	0.161	0.324	[0.029, 0.618]	**0.027**	0.266	0.429	[0.234, 0.623]	** < 0.001**	0.381
Non-Misinformation Manipulativeness	0.013	[-0.259, 0.284]	0.994	0.010	0.129	[-0.139, 0.396]	0.495	0.117	0.095	[-0.097, 0.286]	0.476	0.086
Manipulativeness Discernment	0.177	[-0.194, 0.549]	0.501	0.104	0.195	[-0.207, 0.597]	0.490	0.118	0.334	[0.065, 0.603]	**0.010**	0.217
Misinformation Confidence	0.086	[-0.148, 0.321]	0.664	0.080	0.228	[-0.019, 0.475]	0.077	0.224	0.218	[0.039, 0.398]	**0.012**	0.212
Non-Misinformation Confidence	0.084	[-0.142, 0.309]	0.658	0.081	-0.170	[-0.413, 0.073]	0.226	-0.171	-0.016	[-0.208, 0.176]	0.979	-0.015
Misinformation Sharing Intent	-0.008	[-0.365, 0.349]	0.998	-0.005	-0.191	[-0.496, 0.114]	0.304	-0.151	0.082	[-0.158, 0.323]	0.700	0.060
Non-Misinformation Sharing Intent	0.108	[-0.231, 0.447]	0.734	0.070	-0.146	[-0.507, 0.214]	0.606	-0.098	0.297	[0.043, 0.551]	**0.017**	0.204
Sharing Intent Discernment	0.116	[-0.198, 0.430]	0.660	0.081	0.045	[-0.313, 0.402]	0.954	0.030	0.215	[-0.031, 0.460]	0.100	0.154
**Good-Evil**												
Misinformation Manipulativeness	0.208	[-0.030, 0.446]	0.100	0.191	0.027	[-0.244, 0.297]	0.971	0.025	-0.068	[-0.251, 0.115]	0.660	-0.066
Non-Misinformation Manipulativeness	-0.107	[-0.377, 0.163]	0.618	-0.087	-0.186	[-0.450, 0.078]	0.223	-0.175	-0.234	[-0.421, -0.048]	**0.009**	-0.225
Manipulativeness Discernment	0.315	[-0.044, 0.675]	0.099	0.192	0.212	[-0.177, 0.602]	0.405	0.135	0.167	[-0.094, 0.427]	0.291	0.114
Misinformation Confidence	0.107	[-0.130, 0.344]	0.540	0.099	-0.050	[-0.288, 0.189]	0.876	-0.052	-0.007	[-0.179, 0.165]	0.995	-0.007
Non-Misinformation Confidence	-0.003	[-0.239, 0.234]	1.000	-0.002	0.099	[-0.156, 0.355]	0.631	0.097	0.084	[-0.104, 0.273]	0.547	0.080
Misinformation Sharing Intent	-0.256	[-0.596, 0.084]	0.180	-0.165	-0.037	[-0.331, 0.256]	0.952	-0.032	-0.173	[-0.408, 0.061]	0.192	-0.132
Non-Misinformation Sharing Intent	0.219	[-0.118, 0.556]	0.279	0.142	0.101	[-0.265, 0.467]	0.794	0.068	0.197	[-0.062, 0.456]	0.175	0.136
Sharing Intent Discernment	0.476	[0.139, 0.812]	**0.003**	0.309	0.138	[-0.240, 0.516]	0.665	0.091	0.370	[0.107, 0.634]	**0.003**	0.250

Bold *p*-values indicate statistical significance at the 5% significance level. CI stands for confidence interval.

**Fig. 3:**
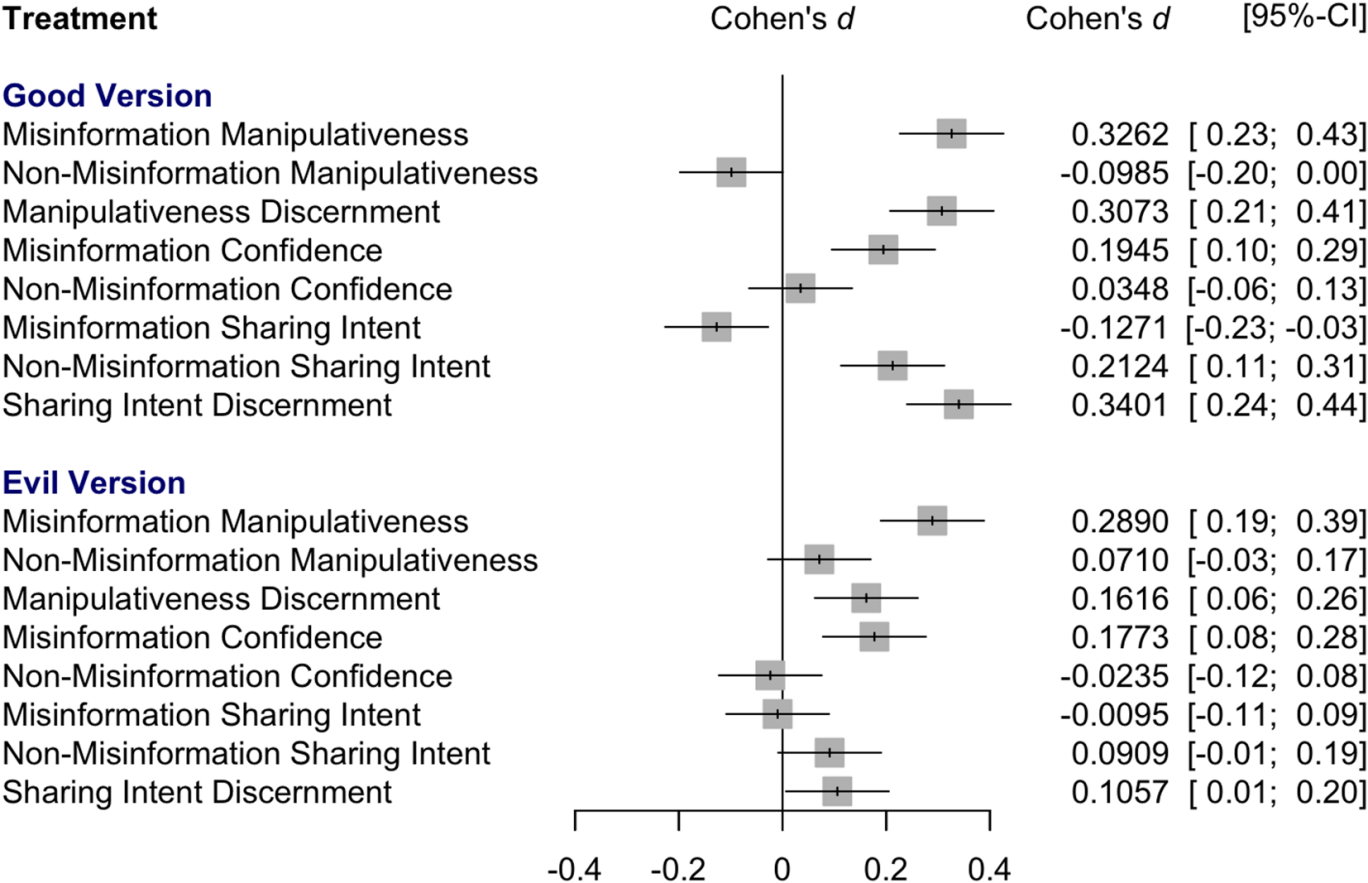
Cohen’s *d* by treatment and outcome from a network meta-analysis across Studies 1 to 3: Forest plot for a network meta-analysis of all outcome measures.

In the network meta-analysis (see Fig. 3), both the “good” (Cohen’s *d* = 0*.*33*, SE* = 0*.*05*, p* < 0*.*001) and the “evil” version of the game (Cohen’s *d* = 0*.*29*, SE* = 0*.*05*, p* < 0*.*001) have a significant effect across Studies 1 to 3 compared to the control group for the misinformation items.

Overall, this supports **H1a**: Playing the *Bad Vaxx* game increases players’ ability to detect manipulative information, and more consistently so for the “good” version of the game.

Conversely, a one-way analysis of variance shows that the effect of the *Bad Vaxx* game on participants’ ratings of non-misinformation items is nonsignificant in Studies 1 and 2. Further, all pairwise comparisons are nonsignificant in Studies 1 and 2. Only in Study 3, a one-way analysis of variance shows that the effect of the *Bad Vaxx* game on ratings of non-misinformation items is significant (Study 3: *F* (2*,* 713*.*98) = 4*.*51*, p* = 0*.*011*, η*^2^ = 0*.*01). This is because participants playing the “good” version (*M* = 3*.*07*, SD* = 0*.*99) rate non-misinformation as significantly less manipulative than those playing the “evil” version (*M* = 3*.*3*, SD* = 1.09; *M*_*diff*_ = *−*0*.*23*, p* = 0*.*009).

The meta-analysis shows that for non-misinformation posts, the effect of both the “good” and the “evil” version of the Bad Vaxx game is nonsignificant compared to the control group.

For discernment, that is, the difference between manipulativeness ratings for manipulative and non-manipulative content, a one-way analysis of variance shows that the effect of the *Bad Vaxx* game is significant in Studies 1, 2 and 3 (Study 1: *F* (2*,* 457*.*59) = 5*.*26*, p* = 0*.*006*, η*^2^ = 0*.*01; Study 2: *F* (2*,* 368*.*26) = 3*.*21*, p* = 0*.*042*, η*^2^ = 0*.*01; Study 3: *F* (2*,* 713*.*4) = 11*.*02*, p* < 0*.*001*, η*^2^ = 0*.*02). Pairwise comparisons using the Games-Howell post hoc criterion for significance indicate that participants in the “good” condition (Study 1: *M* = 2*.*43*, SD* = 1*.*59; Study 2: *M* = 2*.*87*, SD* = 1*.*47; Study 3: *M* = 2*.*64*, SD* = 1*.*38) of the game are significantly better at discerning manipulative from non-manipulative information than participants in the control condition in all studies (Study 1: *M* = 1*.*94*, SD* = 1*.*7; *M*_*diff*_ = 0*.*49*, p* = 0*.*004; Study 2: *M* = 2*.*46*, SD* = 1*.*65; *M*_*diff*_ = 0*.*41*, p* = 0*.*032; Study 3: *M* = 2*.*14*, SD* = 1*.*54; *M*_*diff*_ = 0*.*5*, p* < 0*.*001). Participants in the “evil” condition (*M* = 2*.*48*, SD* = 1*.*55) were significantly better at detecting manipulative headlines than participants in the control condition only in Study 3 (*M*_*diff*_ = 0*.*33*, p* = 0*.*010). The pairwise comparisons between the “evil” condition (Study 1: *M* = 2*.*11*, SD* = 1*.*7; Study 2: *M* = 2*.*66*, SD* = 1*.*67) and the control condition are nonsignificant in Studies 1 and 2. The pairwise comparison between the “good” and the “evil” condition is nonsignificant in all studies. Note that we did not preregister **H1b** for Study 1.

For discernment, the meta-analysis shows that both the “good” (Cohen’s *d* = 0*.*31*, SE* = 0*.*05*, p* < 0*.*001) and the “evil” version (Cohen’s *d* = 0*.*16*, SE* = 0*.*05*, p* = 0*.*001) have significantly higher discernment than the control group.

Overall, this supports the hypothesis **H1b** that playing the *Bad Vaxx* game increases players’ ability to discern manipulative information from non-manipulative information about vaccination.

### Intervention increases confidence in the assessment of the manipulativeness of vaccine misinformation

A one-way analysis of variance (see Table 1) shows that the effect of the *Bad Vaxx* game is significant for Study 3 (*F* (2*,* 715*.*37) = 5*.*42*, p* = 0*.*005*, η*^2^ = 0*.*01), but there are no significant differences in Studies 1 and 2. Similarly, for Study 3, pairwise comparisons using the Games-Howell post hoc criterion for significance indicate that participants in the “good” condition (*M* = 5*.*88*, SD* = 0*.*93) have significantly greater confidence in their manipulativeness ratings than participants in the control condition (*M* = 5*.*67*, SD* = 1*.*06; *M*_*diff*_ = 0*.*21*, p* = 0*.*011), but there are no significant differences in Studies 1 and 2. The pairwise comparisons between the “evil” condition and the control condition, as well as between the “evil” and the “good” condition are nonsignificant.

The meta-analysis shows that for misinformation posts, both the “good” (Cohen’s *d* = 0.19, SE = 0.05, *p *< 0.001) and the “evil” version of the game (Cohen’s *d *= 0.18, SE = 0.05, *p* < 0.001) significantly increase confidence in manipulativeness ratings across Studies 1 to 3 compared to the control group.

Overall, this partially supports hypothesis **H2**, that participants playing the *Bad Vaxx* game have greater confidence in their rating of manipulative information.

There is no significant effect of either the “good” or the “evil” condition on participants’ confidence in their ability to assess non-misinformation items in any of the individual studies or in the meta-analysis.

### Intervention improves the quality of sharing decisions

A one-way analysis of variance (see Table 1) shows that the effect of the *Bad Vaxx* game on participants’ willingness to share misinformation items is nonsignificant and all pairwise comparisons are nonsignificant. Thus, our omnibus ANOVA-based results do not support hypothesis **H3a** that the game decreases the willingness to share vaccine misinformation.

However, the network meta-analysis results that pool data from Studies 1 to 3 show that playing the “good” version of the game significantly reduces the likelihood to share misinformation (Cohen’s *d* =  *− *0*.*13*, SE* = 0*.*05*, p* = 0*.*012), while results for the “evil” version are nonsignificant. This partially supports hypothesis **H3a** that the game decreases the willingness to share vaccine misinformation.

With respect to non-misinformation, a one-way analysis of variance shows that the effect of the *Bad Vaxx* game is significant only in Study 3 (Study 3: *F* (2*,* 714*.*34) = 10*.*62*, p* < 0*.*001*, η*^2^ = 0*.*02), but not in Studies 1 and 2. Pairwise comparisons using the Games-Howell post hoc criterion for significance indicate that participants in the “good” condition (Study 3: *M* = 3*.*48*, SD* = 1*.*46) are significantly more willing to share non-manipulative posts than participants in the control condition in Study 3 (Study 3: *M* = 2*.*99*, SD* = 1*.*47; *M*_*diff*_ = 0*.*49*, p* < 0*.*001). The pairwise comparisons between the “evil” condition (Study 3: *M* = 3*.*28*, SD* = 1*.*45) and the control condition is significant only in Study 3 (*Mdiff* = 0*.*3*, p* = 0*.*017). The pairwise comparisons between the “good” and the “evil” condition are nonsignificant in all studies.

The meta-analysis shows that for non-misinformation posts, only the “good” (Cohen’s *d* = 0*.*21*, SE*= 0*.*05*, p* < 0*.*001), but not the “evil” version of the game (Cohen’s *d* = 0*.*09*, SE* = 0*.*05*, p* = 0*.*073) significantly increase sharing intent for non-misinformation across Studies 1 to 3 compared to the control group.

In terms of discernment, that is, the difference between intent to share non-misinformation and intent to share misinformation, a one-way analysis of variance shows that the effect of the *Bad Vaxx* game is significant in Studies 1 and 3 (Study 1: *F* (2*,* 456*.*16)= 10*.*06*, p* < 0*.*001*, η*^2^ = 0*.*03; Study 3: *F* (2*,* 706*.*02) =15*.*97*, p* < 0*.*001*, η*^2^ =0*.*03), but not in Study 2. Pairwise comparisons using the Games-Howell post hoc criterion for significance indicate that in Studies 1 and 3, participants in the “good” condition (Study 1: *M* = 1*.*65*, SD* = 1*.*55; Study 3: *M* = 1*.*61*, SD* = 1*.*48) have significantly higher sharing intent discernment than participants in the control condition (Study 1: *M* = 1*.*06*, SD* = 1*.*34; *M*_*diff*_ = 0*.*59*, p* < 0*.*001; Study 3: *M* = 1*.*03*, SD* = 1*.*32; *M*_*diff*_ = 0*.*58*, p* < 0*.*001) and in the “evil” condition (Study 1: *M* = 1*.*17*, SD* = 1*.*53; *M*_*diff*_ = 0*.*48*, p* = 0*.*003; Study 3: *M* = 1*.*03*, SD* = 1*.*32; *M*_*diff*_ = 0*.*37*, p* = 0*.*003). The pairwise comparison between the “evil” condition and the control condition is nonsignificant in all studies. Note that we did not preregister **H3b** for Study 1 and **H3a** for Study 2.

The meta-analysis shows that for discernment, both the “good” (Cohen’s *d* = 0*.*34*, SE* = 0*.*05*, p* < 0*.*001) and the “evil” version of the game (Cohen’s *d* = 0*.*11*, SE* = 0*.*05*, p* = 0*.*037) significantly increase the quality of people’s sharing decisions across Studies 1 to 3 compared to the control group.

Overall, this supports hypothesis **H3b** that playing the *Bad Vaxx* game increases the quality of people’s sharing decisions, in the sense that they have a higher difference in willingness to share non-manipulative versus manipulative information about vaccines.

### Intervention improves manipulation technique recognition

To disentangle whether the *Bad Vaxx* game improves the identification of true versus false information, manipulative versus non-manipulative information, or people’s ability to recognize specific manipulation techniques (emotional storytelling, pseudo-science/fake expertise, the naturalistic fallacy, and conspiracy theories), we measured participants’ accuracy in rating 16 headlines that differed along all three dimensions (true or false, manipulative or non-manipulative, and which manipulation technique, if any, is used). We focus on technique recognition accuracy here, and provide detailed results in SI Subsection *Disentangling Identification of Truthfulness, Manipulativeness, and Manipulation Techniques*.

**Fig. 4:**
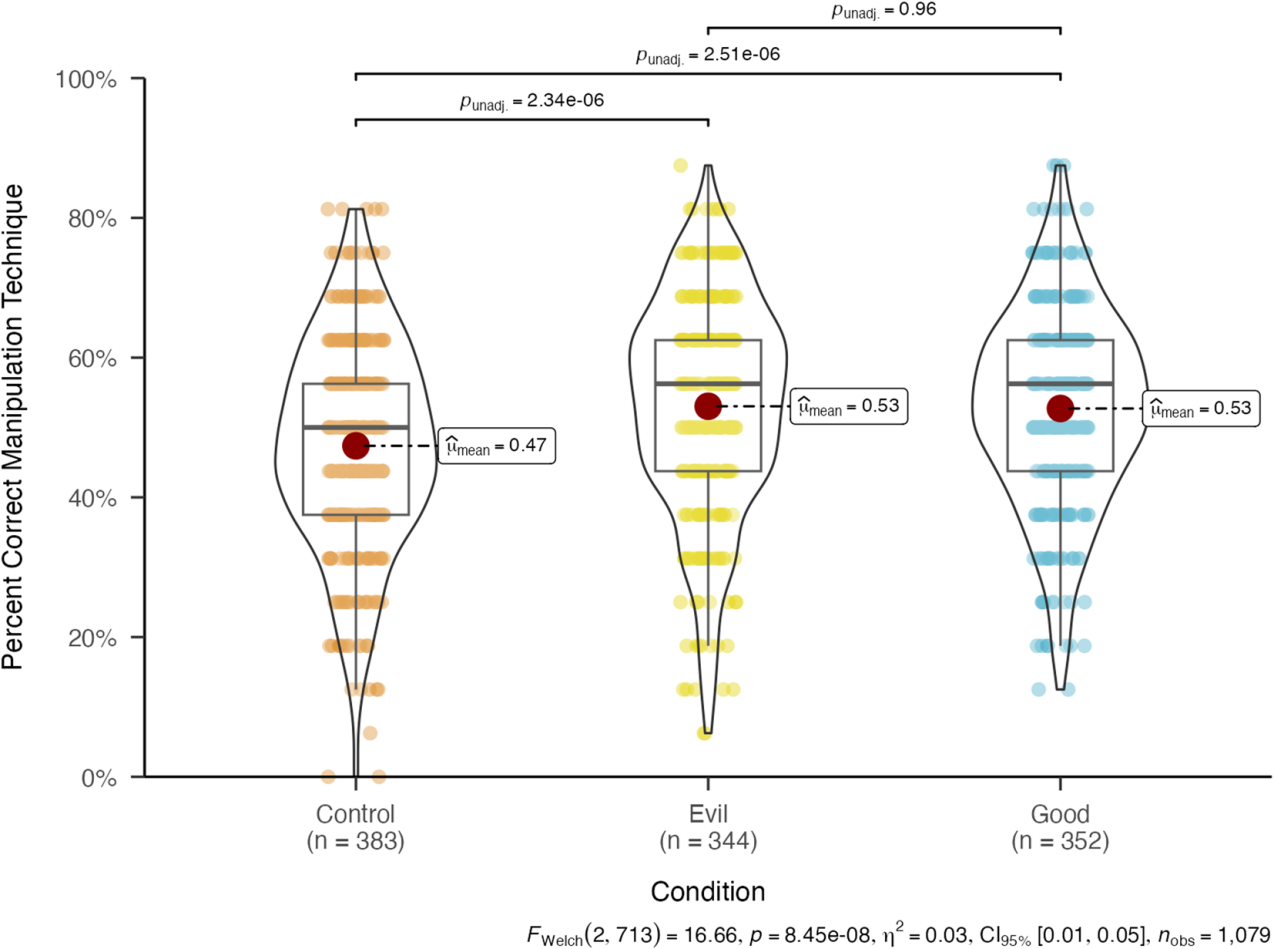
Manipulation technique recognition in Study 3: Overall and pairwise comparison of the effect of the *Bad Vaxx* game on Manipulation Technique Recogni- tion.

A one-way analysis of variance shows that the effect of the *Bad Vaxx* game on manipulation technique recognition is significant (*F* (2*,* 712*.*96) = 16*.*66*, p* < 0*.*001*, η*^2^ = 0*.*03) in Study 3 (see Fig. 4). Pairwise comparisons using the Games-Howell post hoc criterion for significance indicate that participants in the “good” condition (*M* = 0*.*53*, SD* = 0*.*14) are significantly more likely to recognize the correct manipulation technique than participants in the control condition (*M* = 0*.*47*, SD* = 0*.*15; *M*_*diff*_ = 0*.*05*, p* < 0*.*001*,* Cohen’s *d* = 0*.*37). Similarly, participants in the “evil” condition (*M* = 0*.*53*, SD* = 0*.*16) are significantly more likely to recognize the correct manipulation technique than participants in the control condition (*M*_*diff*_ = 0*.*06*, p* < 0*.*001*,* Cohen’s *d* = 0*.*37). The pairwise comparison between the “good” and the “evil” perspective condition is nonsignificant (see Fig. 4). Although not one of our main analyses, this preregistered exploratory analysis suggests that a mechanism underlying the effectiveness of the *Bad Vaxx* game is improved manipulation technique recognition.

It is worth noting that only the effect on the technique recognition accuracy score is significant. While we might be underpowered to detect an effect on truthfulness or manipulativeness accuracy scores across all content, this suggests that the *Bad Vaxx* game improves the identification of specific manipulation techniques, not the identification of truthfulness absent specific manipulation techniques. This may be explained in part because the game does not necessarily teach people what is true or false when it comes to vaccines, but rather how to identify markers or cues of manipulative argumentation.

In a non-preregistered analysis, we further disentangled the results for identification of true versus false content, manipulative versus non-manipulative content, and manipulation technique recognition. We find that the game works as intended in that playing the game increases people’s ability to correctly identify manipulation techniques only for manipulative posts, but not non-manipulative posts (see SI Figure S15). Further, the game makes participants more skeptical of false and manipulative content (effect only significant for the “good” version), but not of true and non-manipulative content (see SI Figure S16).

## Discussion

The *Bad Vaxx* game is designed to address vaccine misinformation and its specific narratives. It inoculates participants against vaccine misinformation by exposing them to weakened doses of manipulation techniques—rather than specific myths or arguments—that are commonly used to spread vaccine misinformation. In internal meta-analyses of a series of three pre-registered randomized controlled trials with 2,326 participants, we find that both versions of the game we developed, which differ in the perspective the participant takes, significantly increase participants’ ability to distinguish vaccine misinformation from non-misinformation. Furthermore, the internal meta-analyses show that participants who play either version of the *Bad Vaxx* game are significantly more confident in their assessment of the manipulativeness of vaccine misinformation than those in the control group. These findings are in line with previous research on gamified inoculation n interventions^18,19,35^. We also find that taking the perspective of a character *fighting* (“good” version) as opposed to *spreading* misinformation (“evil” version) is more effective.

We find consistent results across the three studies for outcomes such as misinformation manipulativeness ratings, but we also observe some differences between individual studies. We therefore conducted an internal meta-analysis to get more robust estimates of effect sizes. There are multiple potential reasons for the inconsistencies. First, Study 2 had the lowest sample size and therefore lowest power of all three studies. Reassuringly, the direction of effects was consistent for all significant effects discovered in Study 2, and consistent for all outcomes except for non-misinformation confidence and sharing-intent compared to Study 1 and 3. Second, sample composition differed. For example, Study 2 had a higher average education level (see SI Tables S13-S15 and S19-S21 for demographic information). Third, as detailed in the *Methods* Section, we made minor changes to some outcome measure items and the game itself between studies. Study 3 was the largest study and used a representative sample, so we think of that study and the internal meta-analysis as providing the most reliable guidance for future research and practitioners.

With respect to people’s willingness to share vaccine misinformation, our internal meta-analyses show that playing the game increases the quality of people’s sharing decisions, that is, the difference between intent to share non-manipulative versus manipulative vaccine information. This effect is stronger for the “good” version than for the “evil” version of the game. Interestingly, the difference in sharing intentions is driven more by a higher willingness to share non-manipulative information than by a lower willingness to share manipulative information. Because sharing intentions for misinformation and manipulative content are known to be very low^10,36^, including in the studies presented here, measuring the intent to share misinformation may be complicated by floor effects, where participants start out at the lower end of a distribution and cannot move any lower. Indeed, the distribution of willingness to share manipulative information in our data is highly right-skewed with most weight at the lowest scores, in contrast to willingness to share non-manipulative information. Instead of indicating lower willingness to share manipulative information, treatment group participants may therefore indicate higher willingness to share non-manipulative information , the distribution of which is not as constrained, implying that the observed effects could potentially result from the design of the item rating task rather than a true increase in willingness to share non-misinformation with others.

Our internal meta-analyses provide robust estimates of the effect sizes. As prior research on inoculation theory has noted, small effects in this context are meaningful^29,37,38^. In Study 3, we implemented a technique recognition task that shows that playing a 15-min online game improves individuals’ ability to recognize manipulation techniques commonly used to spread vaccine misinformation by 5.7% and 5.3% on average for the “evil” and the “good” versions of the game, respectively. Because accuracy in identifying manipulation techniques ranges from 0 to 1, these percentages can simply be calculated as the difference between the accuracy for participants in the treatment versus control groups (see Fig. 4).

Although we did not hypothesize which effect the *Bad Vaxx* game would have on the evaluation of non-misinformation, analyzing this effect is important because, following g the vaccination analogy, an intervention that improves participants’ ability to identify manipulative content could have negative side effects in that it might make participants suspicious of all content across the board^39,40^. Encouragingly, we do not see any significant negative effects on the perception of non-misinformation. On the contrary, our internal meta-analyses show that the sharing of non-misinformation—that is, broadly reliable social media content about vaccines—is significantly higher for players who play the “good” version of the game compared to participants in the control group. In a non-preregistered exploratory analysis of a rating task in Study 3 where content varies in terms of true versus false, manipulative versus non-manipulative, and manipulation technique used, we further see that the “good” version of the game induces skepticism of manipulative and false information, but not true and non-manipulative information. The effects for the “evil” version go in the same direction, but do not reach significance.

In additional, non-preregistered exploratory analyses, we find that the effect of the game does not vary significantly by potential moderator variables such as political ideology or COVID-19 vaccination intentions, except for significant interactions for confidence in manipulativeness ratings (see SI Subsection *Interaction Between Condition and Covariates*, SI Tables S5-S14). This implies that the game is an intervention that can work for a broad cross-section of the population. However, as with any voluntary intervention, our findings here apply only to people who choose to play the game in the first place^10^.

Importantly, this study does not only address *whether*, but also *why* the *Bad Vaxx* game might be effective by testing the importance of the player’s perspective and by analyzing what exactly participants learn through the game. Comparing two versions of the *Bad Vaxx* game, a “good” and an “evil” version, allows us to investigate whether the perspective that a player takes affects the efficacy of the inoculation intervention. We find that overall, both versions of the game are effective, but the “good” version trumps the “evil” version on most measures, although differences between the two versions are not always significant. Possible explanations for this include the suitability of the “good” perspective in the context of misinformation and higher appeal of the “good” version among survey respondents. First, perspective-taking might be easier when a participant plays as a good person trying to stop the spread of misinformation, as opposed to taking on the role of the apprentice of a misinformation spreader, because this might be closer to their own perspective. Better perspective-taking could enhance learning. Relatedly, the “good” version may be more suitable because it may serve as better practice for what a social media user who encounters misinformation might actually do. It is also possible that the “good” version works slightly better because being in the position of a hero helps better convey the learning content. For example, Chalaya et al.^41^ find that positioning an audience as a hero increases support for particular policies . Additionally, feeling a certain level of threat is an important mechanism in inoculation theory^42^, and the “good” version of the game may already induce sufficient threat by exposing participants to manipulative content. Second, despite a rigorous effort to keep the two versions as similar in content and presentation as possible, the “good” version may simply be more appealing . We developed the “good” version first and then created the “evil” version by mirroring the “good” version, with the only major change being the perspective as a good or bad actor. Therefore, it could be that the “good” version is more clear. However, participant feedback suggests that both versions of the game were similarly enjoyable. Comparing two versions of the same game contrasts most existing literature that tends to examine a single, static intervention and pays much more attention to the diversity in participants than the diversity in stimuli, which limits generalizability^43^ and makes it difficult to analyze which mechanism might make a particular stimulus more effective. The differences in effectiveness of the two game versions on some outcome measures suggests that a closer look at mechanisms and distinct stimuli is important to understand the effectiveness of an intervention and enable iterative improvement.

In terms of identifying what participants learn through the game, our exploratory results disentangle the game’s effect on participants’ ability to assess content with regards to its truthfulness (i.e., whether it is true or false), manipulativeness (i.e., whether it is manipulative or non-manipulative), and use of a manipulation technique (i.e., which, if any, manipulation technique was used). Consistent with the goal of the intervention, the results suggest that playing the game significantly enhances people’s ability to detect misinformation based on recognizing techniques that are employed to spread such misinformation, but not the truthfulness of the content overall. Encouragingly, this makes the intervention applicable to a broad range of content that people might see online, provided that content makes use of a manipulation technique that people were inoculated against.

The theory-guided design of the *Bad Vaxx* game combines the strengths of issue-based and technique-based inoculation that have traditionally been distinguished in the inoculation theory literature^15,16^. While the game focuses on the issue of vaccine misinformation, it successfully teaches participants techniques that are commonly used to spread different kinds of misinformation rather than specific counterarguments.

This could make the intervention effects more generalizable across different kinds of misinformation that people might encounter, and promote long-term resistance in the face of constantly evolving anti-vaccine rhetoric and vaccine misinformation.

While we do not test the generalizability of effects over time, there is encouraging evidence that inoculation interventions like the *Bad Vaxx* game can have lasting effects when post tests or boosters are administered^24,44^.

Our study is limited in terms of online panel sample quality, ecological validity, and generalizability. Even though the game itself simulates a social media setting, our study is limited in terms of its ecological validity because the experiments were conducted on an online survey platform geared towards internal validity that cannot perfectly mimic a social media environment. Relatedly, while our outcome measures are based on evaluations of headlines designed to match real misinformation seen on social media, these headlines might not generalize to the much more diverse set of vaccine misinformation headlines on social media. We also assessed only sharing intentions, not sharing behavior, and the two do not always align^45^, so we are not able to speak to how people’s sharing behavior might change post-gameplay. Finally, our studies were conducted in the United States, and our findings may not generalize to other audiences, particularly outside of Western or English-speaking countries. This is a substantial problem in misinformation research in general, which requires careful consideration^46,47^.

Future research may explore for whom interventions like the *Bad Vaxx* game work best and which groups should be targeted to maximize impact. Some populations, such as individuals with children^48^ or individuals prone to conspiratorial thinking or high in reactance^49^, might be more likely to have anti-vaccine attitudes. Whether and how well the game works for these populations, or whether it causes reactance (e.g., people with low vaccine confidence may be substantially more reluctant to play the game in the first place), remains an open question. Other populations, such as doctors and nurses, may be an effective audience of multipliers that can raise awareness about manipulation techniques used in vaccine misinformation among their patients^50^. Future research may also investigate more of the potential mechanisms that might drive the effectiveness of interventions like the *Bad Vaxx* game, including by using gamified or alternative control groups with more vaccine content. Moving towards a dynamic intervention framework could allow for testing several different mechanisms and iterate on an intervention to increase its effectiveness. With the aim of making a version of the experimentally evaluated game freely available to the public and facilitating further research, we have released a version that underwent final production by game designers at https://www.badvaxx.com/. Future work could also investigate how scalability and effect sizes differ for different types of interventions, for example comparing the *Bad Vaxx* game to static interventions like informational text. Indeed, recent research aimed at increasing people’s ability to identify content from coordinated misinformation campaigns showed that static content—such as a video- and text-based tutorials with relevant information—can increase people’s ability to identify coordinated misinformation, and adding short text-based summaries to the tutorials can increase the effect^51^. In a direct comparison between a gamified intervention and infographics, Basol et al.^19^ found comparable effect sizes for both interventions, but also noted that the game was more popular and had better longevity (significant effects were reported one week post-intervention for the game, but not for the infographics). More broadly, Roozenbeek et al.^52^ note that intervention efficacy (successful lab studies) does not necessarily translate to real-world effectiveness. They argue that auxiliary factors such as (re)playability, a stable online presence, entertainment value, and potential for use in educational settings are important considerations for whether an intervention “works” in the real world. We argue that gamified interventions such as the *Bad Vaxx* game have the potential to achieve meaningful impact at scale, as they generally enjoy additional benefits alongside boosting outcome measure performance.

In conclusion, we show that a practical, entertaining intervention in the form of an online game can induce broad-scale resilience against manipulation techniques commonly used to spread false and misleading information about vaccines. The dynamic nature of the game opens up many new possibilities for testing relevant mechanisms that might underlie its effectiveness. Consistent with findings from meta-analyses^21,29^, we show that inoculation theory-based treatments can improve discernment, whether the player takes on the perspective of a “good” actor that learns to fight misinformation or an “evil” actor that learns how spread vaccine misinformation. A framework similar to ours could be leveraged to study a variety of potential mechanisms for the effectiveness of persuasive interventions, including humor, the opportunity for visual learning, immersion into a story, and personalization of the game experience. Importantly, our finding that learning to recognize specific misinformation techniques reduces susceptibility to vaccine misinformation suggests that focusing on learning a few common techniques, rather than myriads of facts about vaccines, can be an effective and viable strategy.

## Methods

All experimental protocols were approved by the ethics review boards at the University of Cambridge, Duke University, and Stanford University. The methods were carried out in accordance with the relevant guidelines and regulations. Informed consent was obtained from all participants.

We test the following preregistered hypotheses, which are similar for all three studies (see the pre-registrations for Studies 1, 2 and 3) and broadly follow Basol et al.^19^:

### H1

Participants who play either version of the *Bad Vaxx* game will have a significantly greater ability to distinguish manipulative from non-manipulative information about vaccines than those in the control group.

### H1a

They will rate manipulative information as more manipulative.

### H1b

They will have higher discernment ratings (difference between ratings of manipulative versus non-manipulative information).

### H2

Participants who play either version of the *Bad Vaxx* game will be significantly more confident in their ability to assess the manipulativeness of manipulative vaccine-related information than those in the control group.

### H3

Participants who play either version of the *Bad Vaxx* game will be significantly less willing to share vaccine misinformation after playing the game compared to the control group.

### H3a

They will be less willing to share manipulative information.

### H3b

They will have a higher difference in willingness to share (difference between willingness to share non-manipulative versus manipulative information).

### Detailed study overview

Study 1 was a proof of concept aimed at testing the validity of our measures and ensuring a good participant experience with the game running smoothly. Based on a power analysis conducted in GPower (F-tests, one-way ANOVA, 3 groups, 95% power, expected effect size Cohen’s *f* = 0.15, or Cohen’s *d* = 0.30, which gives a target sample of *N* = 690), we recruited 690 US participants via Prolific Academic in November 2020. Median age was between 25 and 34, and 49.5% of the sample was male (48.5% female, 2% other; excluding missing values).

Study 2 aimed to replicate the findings from Study 1. Based on feedback from Study 1, we made slight modifications to the game’s content in order to improve its flow and clarity. We also implemented CAPTCHAs and additional attention checks to ensure the highest possible sample quality. We also changed the wording of a few items, as several participants seemed confused about the distinction between manipulative and non-manipulative items (and upon review we agreed with this). These changes did not impact the overall results (see meta-analyses in the “*Results* Section”). See the Supplementary Information folder on OSF for the full item wordings for Study 1 and Studies 2 and 3. We made small revisions to the “evil” version of the game, which performed worse than the “good” version in Study 1. These changes involved improving the flow and entertainment value of the game, and moving slightly away from the “evil” version being an exact copy in terms of content of the “good” version. We recruited 557 US participants on Prolific Academic in January 2021. Median age was between 25 and 34, and 42.2% of the sample was male (55.1% female, 2.7% other; excluding missing values).

Study 3 aimed to test the robustness of the effects of our intervention with a larger, representative sample given that the results of Study 2 did not fully align with those of Study 1. In addition, we sought to identify further mechanisms behind our findings from Studies 1 and 2, namely whether improved resilience to misinformation is due to an improved ability to distinguish true from false information about vaccinations, due to better recognition of whether a social media post is manipulative, or because people have learned to recognize the specific manipulation techniques taught in the game. We recruited 1,079 participants representative of the US population on Prolific Academic in October 2021. Median age was between 25 and 34, and 48.5% of the sample was male (49.3% female, 2.2% other; excluding missing values).

For all studies, our screening criteria required that all participants resided in the United States, were at least 18 years old, and had at least one active social media account. In line with our preregistration, we excluded participants who did not complete the game successfully or failed the attention check. See the SI Section *Descriptive Statistics* for further information about the sample, and SI Section *Deviations and Clarifications* for further clarifications about the exclusion criteria.

### Procedure

**Fig. 5:**
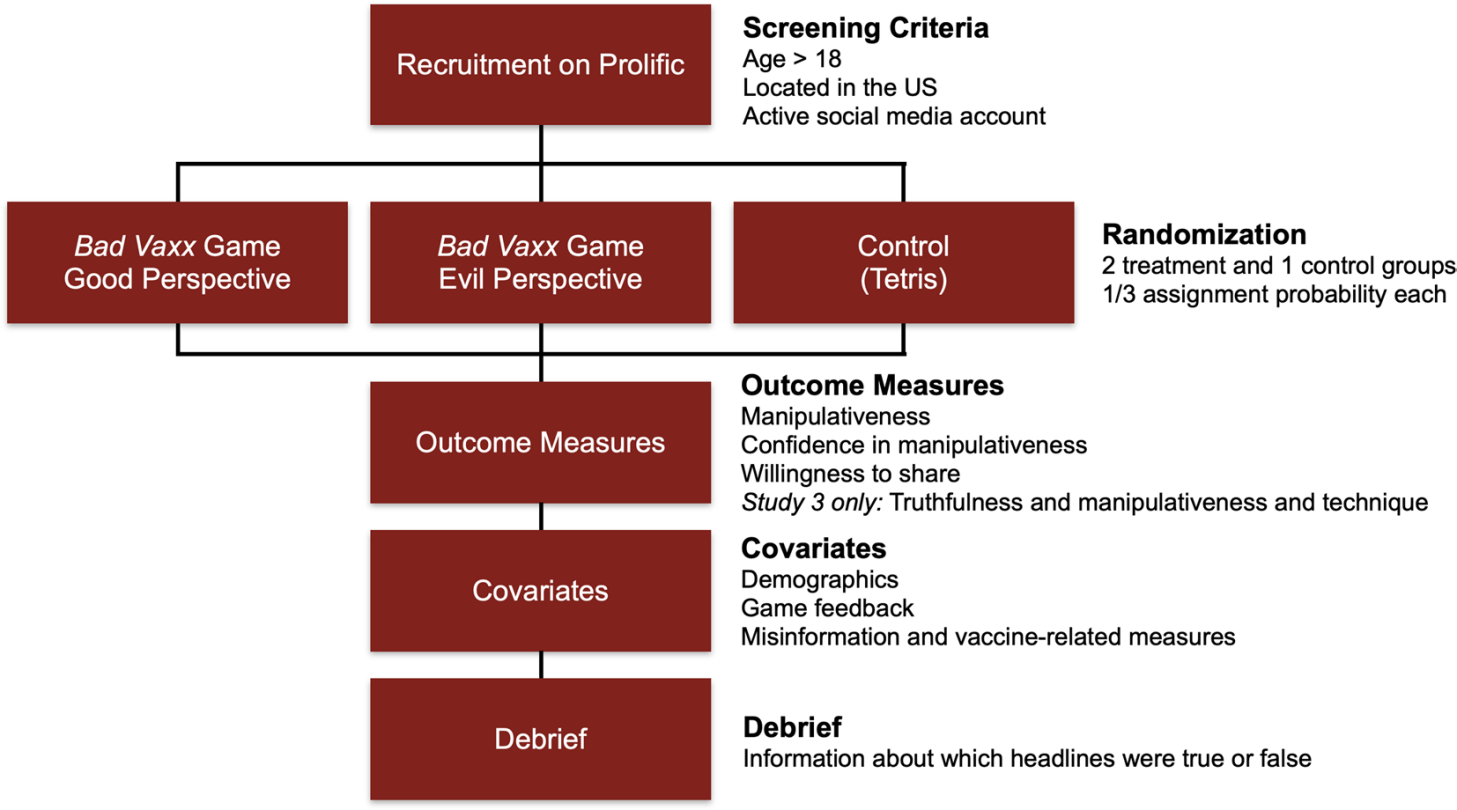
Experiment design overview.

Consenting participants were randomly assigned to one of three conditions: (1) playing the *Bad Vaxx* game from a “good” perspective, such that the player tries to fight vaccine misinformation (treatment condition 1), (2) playing the *Bad Vaxx* game from an “evil” perspective, such that the player tries to spread simulated vaccine misinformation (treatment condition 2), or (3) playing *Tetris* (control condition; see Fig. 5 for an overview of the experiment design). The two treatment conditions are identical in length, aesthetics and content, and only differ in their perspective (good versus evil). Players in the control condition had to play *Tetris* for at least 7 mins, ensuring that all conditions involved playing a game for a similar duration. The final production "good" and "evil" versions of the game, which were refined by game designers, are available at https://www.badvaxx.com/, and Tetris is publicly available at https://rakoen.maertens.international/research-tetris/.

Immediately after completing the game, participants were shown a series of 12 social media posts about vaccines, presented in random order. Participants randomly saw either a misinformation item (we created 3 items for each of the 4 manipulation techniques from the game) or its matched neutral control item so that, on average, each participant saw 6 misinformation items and 6 non-misinformation (neutral) items. This approach has been previously validated and used, e.g. by Roozenbeek et al.^36^. The misinformation items were inspired by content found on anti-vaccine websites, and used one of the four manipulation techniques encountered in the game. For example, a misinformation item using the “conspiracy theory” technique read “Vaccine database wiped by government to hide uptick in admitted vaccine injuries.” For each misinformation item, we created a matched neutral control item that was as similar as possible to its misinformation counterpart in terms of content, but did *not* make use of a manipulation technique, following the procedure laid out by Roozenbeek et al.^36^. For example, the matched control for the above “conspiracy” item read “The US database on vaccine injuries launched.” We sought to mimic (but not copy exactly) real vaccine misinformation examples not only in terms of content, but also in terms of style, and so the final items were displayed in a format similar to a social media post on platforms such as X (formerly Twitter).

Beneath each item, participants were shown three outcome measures, all assessed on a 7-point Likert scale, with 1 being “strongly disagree” and 7 being “strongly agree”: manipulativeness (“This post is manipulative”)^36^, confidence (“I am confident about my assessment of this post’s manipulativeness”)^19^, and sharing intentions (“I would share this post with people in my network”)^53^. Collecting ratings of both misinformation and non-misinformation allowed us to calculate a discernment score (defined as the difference between ratings on manipulative items and ratings on neutral items) for the manipulativeness and sharing measures. We follow the procedure laid out by Roozenbeek et al.^36^.

Study 3 also included a separate manipulation technique recognition task. Here, participants were shown 16 headlines (different from those used in the item rating task above), presented as text (without further formatting), and in random order. These items were constructed in a similar way to the item rating task above. However, with this task, our goal was to disentangle whether playing the game improves people’s ability to detect specific manipulation techniques (learned in the game), and whether a given headline is manipulative, contains false information, or both. To this end, we created four headlines that varied on two dimensions (true vs. false and manipulative vs. non-manipulative). In other words, we created headlines that were either: manipulative and false, non-manipulative and false, manipulative and true, or non- manipulative and true. For example, a false and manipulative headline reads “mRNA vaccine contains “luciferin” in a 66.6 solution, what are they hiding?”; this is false, but also implies a conspiracy. Conversely, a false and non-manipulative headline reads “mRNA vaccines contain “luciferin” enzyme”; this is a false claim but no other manipulation technique is used. Participants rated each of the headlines as (1) true or false and (2) manipulative or non-manipulative. In addition, we (3) asked participants to indicate which manipulation technique was being used (i.e., emotional storytelling, fake expertise, the naturalistic fallacy, conspiracy theories, or none of these). Doing so allowed us to assess if playing the game improves accuracy in detecting manipulative vs. non-manipulative headlines, true vs. false headlines, and explicit manipulation technique recognition. All items are available on OSF (https://osf.io/rdh2b/).

Finally, we administered a range of covariates, including age, gender, political ideology and COVID-19 vaccine intentions. We collected the covariates at the end, which could result in post-treatment bias, but most measures like demographics should not be affected by this type of bias.

### Analyses

We conducted balance checks and found that covariates are relatively balanced across conditions in all studies (see SI Tables S22-S24).

We conducted a series of ANOVAs to analyze between-group differences between each of the three conditions for discernment (for the manipulativeness and sharing measures), and for the misinformation and non-misinformation items separately (see *Results* Section, and SI Section *Overview of ANOVA Results for Studies 1 to 3* for additional visualizations of the results; see SI Tables S37-S39, S55-S57 and S73-S75 for item-level results). We also conducted a series of linear regressions with the outcome variables of interest as the dependent variable, condition as the main regressor of interest, and a series of covariates (see SI Tables S23-S30, S41-S48, and S59-S66). Finally, we preregistered that we would conduct linear regressions at the rating level, clustered on study participants and misinformation versus matched control outcome measures, following Pennycook et al.^53^. Departing from our preregistration, we instead ran a series of multi-level models with participants and items modelled as random effects, and relevant covariates. The latter models are reported in the Supplementary Information (see SI Tables S31-S33, S49-S51 and S67-S69).

We then conducted internal meta-analyses for the manipulativeness, confidence, and sharing measures from Studies 1 to 3. This was preregistered for Study 3. We show results for both a network meta-analysis and a series of pairwise meta-analyses (see *Results* section for a visualization of the network meta-analysis results, SI Table S1 for network meta-analysis results in table form, and SI Figures S18-S25 for detailed results of the pairwise meta-analyses). A network meta-analysis is appropriate if outcomes from multiple studies are compared and there are multiple treatments in each study^33^, and we focus on the results of this internal meta-analysis in the main text. The pairwise meta-analyses allow for a pairwise comparison of all conditions. We present fixed effects estimates given that the study design and measures were highly similar across studies and a random effects model would give more weight to studies with smaller sample sizes that are potentially more biased. We used the meta^54^ and netmeta^55^ packages to conduct the analysis and produce the plots, and by default standard errors for this type of analysis are adjusted for the correlation between the different comparisons in multi-arm studies. All data were analyzed using R^56^.

## Electronic supplementary material

Below is the link to the electronic supplementary material.

Supplementary Information 1.

Supplementary Information 2.

## Data Availability

All information necessary to reproduce our results (including data, items) are available on OSF (https://osf.io/rdh2b/). The pre-registrations are available on AsPredicted for Study 1, 2 and 3.

## Code availability

All information necessary to reproduce our results (including analysis scripts) are available on OSF (https://osf.io/rdh2b/).
