# Supplementary material for: Psychological inoculation improves resilience to and reduces willingness to share vaccine misinformation
Source: Sci Rep. 2025 Aug 18;15:29830. doi: 10.1038/s41598-025-09462-5 (PMC12358572; doi:10.1038/s41598-025-09462-5)
Supplement: Supplementary file 1 — Supplementary Information 1. [file 41598_2025_9462_MOESM1_ESM.docx]

**Supplementary Information for**

**Psychological inoculation improves discernment and reduces willingness to share vaccine misinformation**

**Ruth E. Appel, Jon Roozenbeek, Rebecca Rayburn-Reeves, Melisa Basol, Jonathan Corbin, Josh Compton and Sander van der Linden**

**Jon Roozenbeek.**

**E-mail:** [**jjr51@cam.ac.uk**](mailto:jjr51@cam.ac.uk)

**This PDF file includes:**

Supplementary text Figs. S1 to S25 Tables S1 to S75

SI References

**1 of** [**75**](#_bookmark21)

**Contents**

[**Deviations and Clarifications**](#_bookmark0) [**Extended Data Tables and Figures**](#_bookmark0)

**3**

**3**

3

3

16

16

18

21

21

22

22

22

22

**31**

31

36

**41**

**41**

**41**

41

41

46

**48**

**51**

51

58

66

[Overview of ANOVA Results for Studies 1 to 3](#_bookmark1)

. . . . . . . . . . . . . . . . . . . . . . . . . . . . . . . . . . . . . . .

[Figures and Tables for Overview of ANOVA Results for Studies 1 to 3](#_bookmark1) . . . . . . . . . . . . . . . . . . . . . . .

[Disentangling Identification of Truthfulness, Manipulativeness, and Manipulation Technique](#_bookmark2) . . . . . . . . . . . . . . [Correct Identification](#_bookmark3) . . . . . . . . . . . . . . . . . . . . . . . . . . . . . . . . . . . . . . . . . . . . . . . . . . .

[Manipulativeness, Truthfulness, and Technique Recognition by Post Type](#_bookmark4) . . . . . . . . . . . . . . . . . . . . . [Internal Meta-Analyses](#_bookmark5) . . . . . . . . . . . . . . . . . . . . . . . . . . . . . . . . . . . . . . . . . . . . . . . . . . . .

[Overall Network Meta-Analysis Effects by Treatment and by Outcome](#_bookmark6) . . . . . . . . . . . . . . . . . . . . . . . [Pairwise Meta-Analyses by Outcome](#_bookmark7) . . . . . . . . . . . . . . . . . . . . . . . . . . . . . . . . . . . . . . . . . .

[Manipulativeness](#_bookmark7) . . . . . . . . . . . . . . . . . . . . . . . . . . . . . . . . . . . . . . . . . . . . . . . . . . . . .

[Confidence](#_bookmark7) . . . . . . . . . . . . . . . . . . . . . . . . . . . . . . . . . . . . . . . . . . . . . . . . . . . . . . . .

[Sharing Intent](#_bookmark7) . . . . . . . . . . . . . . . . . . . . . . . . . . . . . . . . . . . . . . . . . . . . . . . . . . . . . . .

[**Interaction Between Condition and Covariates**](#_bookmark8)

[COVID-19 Vaccine Intentions](#_bookmark9) . . . . . . . . . . . . . . . . . . . . . . . . . . . . . . . . . . . . . . . . . . . . . . . . .

[Political Ideology](#_bookmark10) . . . . . . . . . . . . . . . . . . . . . . . . . . . . . . . . . . . . . . . . . . . . . . . . . . . . . . . .

[**Inoculation Theory**](#_bookmark11)

[**Identifying Vaccine Misinformation Techniques**](#_bookmark11)

[**Descriptive Statistics**](#_bookmark11)

[Number of Observations](#_bookmark12) . . . . . . . . . . . . . . . . . . . . . . . . . . . . . . . . . . . . . . . . . . . . . . . . . . . .

[Demographics](#_bookmark13) . . . . . . . . . . . . . . . . . . . . . . . . . . . . . . . . . . . . . . . . . . . . . . . . . . . . . . . . . .

[Group Means for Outcome Variables](#_bookmark15) . . . . . . . . . . . . . . . . . . . . . . . . . . . . . . . . . . . . . . . . . . . . .

[**Balance Tables**](#_bookmark16)

[**Detailed Results**](#_bookmark17)

[Study 1](#_bookmark18) . . . . . . . . . . . . . . . . . . . . . . . . . . . . . . . . . . . . . . . . . . . . . . . . . . . . . . . . . . . . .

[Study 2](#_bookmark19) . . . . . . . . . . . . . . . . . . . . . . . . . . . . . . . . . . . . . . . . . . . . . . . . . . . . . . . . . . . . .

[Study 3](#_bookmark20) . . . . . . . . . . . . . . . . . . . . . . . . . . . . . . . . . . . . . . . . . . . . . . . . . . . . . . . . . . . . .

**2 of** [**75**](#_bookmark21)

**Supplementary Information Text Deviations and Clarifications**

Clarification #1: In the preregistration for Study 1, we wrote “Participants who do not successfully complete a “fake news” game will be excluded; we will check this by including a password at the end of the game that participants have to provide. Participants who fail an attention check will be excluded.” Only participants who played the Bad Vaxx game received a password, so only treatment group participants were excluded based on an incorrect password.

Clarification #2: For Studies 1, 2 and 3, we considered the game attention check password as correct when it was either an exact match for the password “INOC1983”, or a close variant (e.g., “IN0C1983” or “INO”).

Clarification #3: In Studies 1, 2 and 3, we excluded preview responses from the research team, as well as responses from non-consenting participants, participants who did not finish the survey, and participants who had empty Prolific IDs (the latter only occurred in Study 3).

Clarification #4: In the preregistration for Study 2, we wrote “Participants who fail two attention checks will be excluded and resampled.” Consistent with Study 1 and 3, we also excluded participants who failed one attention check, but these participants were compensated in the same way as participants who completed the study.

Clarification #4: In the preregistration for Study 2, we had not specified what we would do with participants with missing values on the main outcome variables like Manipulativeness Discernment. While many regression functions drop these rows automatically, the function we use did not, so we excluded two observations where Manipulativeness Discernment could not be calculated.

Clarification #5: To assess confidence in manipulativeness ratings, we focus on manipulative posts. We mention in the preregistration for Study 3 that we will look at confidence in manipulativeness ratings for both manipulativeness and non-manipulative posts separately, and we present results of both analyses. However, the main outcome of interest is confidence in the manipulativeness ratings of manipulative posts because a positive effect indicates higher confidence and a negative effect lower confidence. A discernment measure that combines manipulative and non-manipulative posts is not clear to interpret since a participant could, for example, have both higher confidence in correctly assessing the manipulativeness of a non-manipulative post as low, and higher confidence in assessing the manipulativeness of a manipulative post as high, resulting in no change in the difference although the confidence for ratings of each post category increased. We did not have prior expectations on how confidence in manipulativeness ratings of non-manipulative information would change, so our hypothesis on confidence in manipulativeness ratings focuses only on manipulative posts.

Clarification #6: We ran additional analyses that were not preregisted, such as testing an interaction between COVID-19 vaccination intentions and condition, or political ideology and condition with regards to our main outcome measures. We preregistered that we would analyze covariates like vaccination intentions, but not that we would include them in an interaction model.

Clarification #7: In the preregistration, we used the terms “vaccine” and “vaccination” interchangeably. Our intervention targets misinformation related both to the process of introducing a vaccine into the body (vaccination) and vaccines more broadly, therefore we opted to use the term “vaccine” consistently in the paper and supplement.

Deviation #1: We preregistered that we would conduct linear regressions at the rating level, clustered on study participants and misinformation vs. matched control outcome measures. Departing from our preregistration, we instead ran multi-level models with participants and items modelled as random effects, and a series of relevant covariates. While both types of analyses have the same aim to account for participant- and item-level variation, we decided to use multi-level modeling since the participant- and item-level variation is more appropriately taken into account in this type of model.

**Extended Data Tables and Figures**

**Overview of ANOVA Results for Studies 1 to 3.**

***Figures for Overview of ANOVA Results for Studies 1 to 3. Manipulativeness.***

**3 of** [**75**](#_bookmark21)


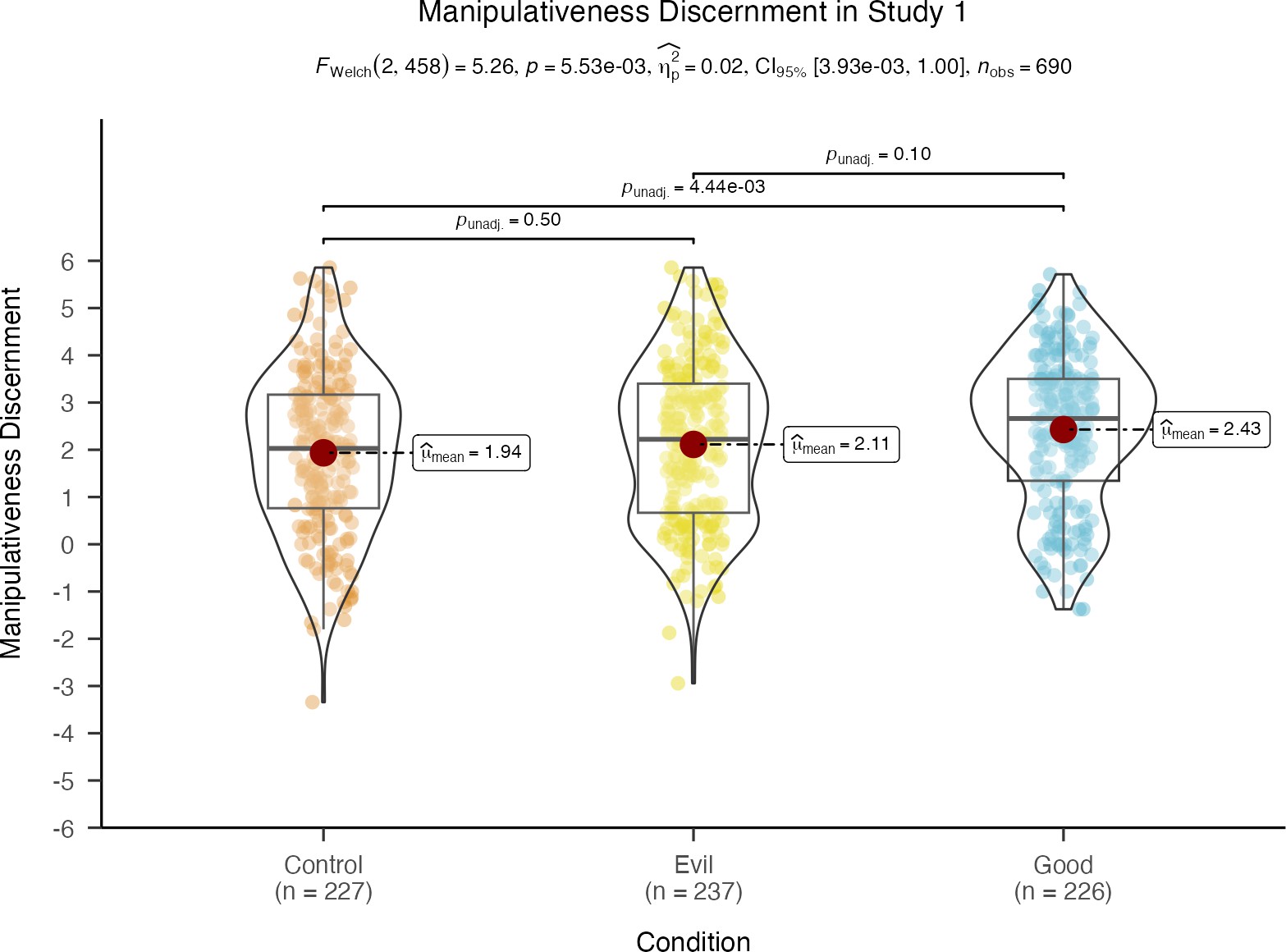


**Fig. S1.** Study 1: Overall and pairwise comparison of the effect of the *Bad Vaxx* game on manipulativeness discernment.

**4 of** [**75**](#_bookmark21)


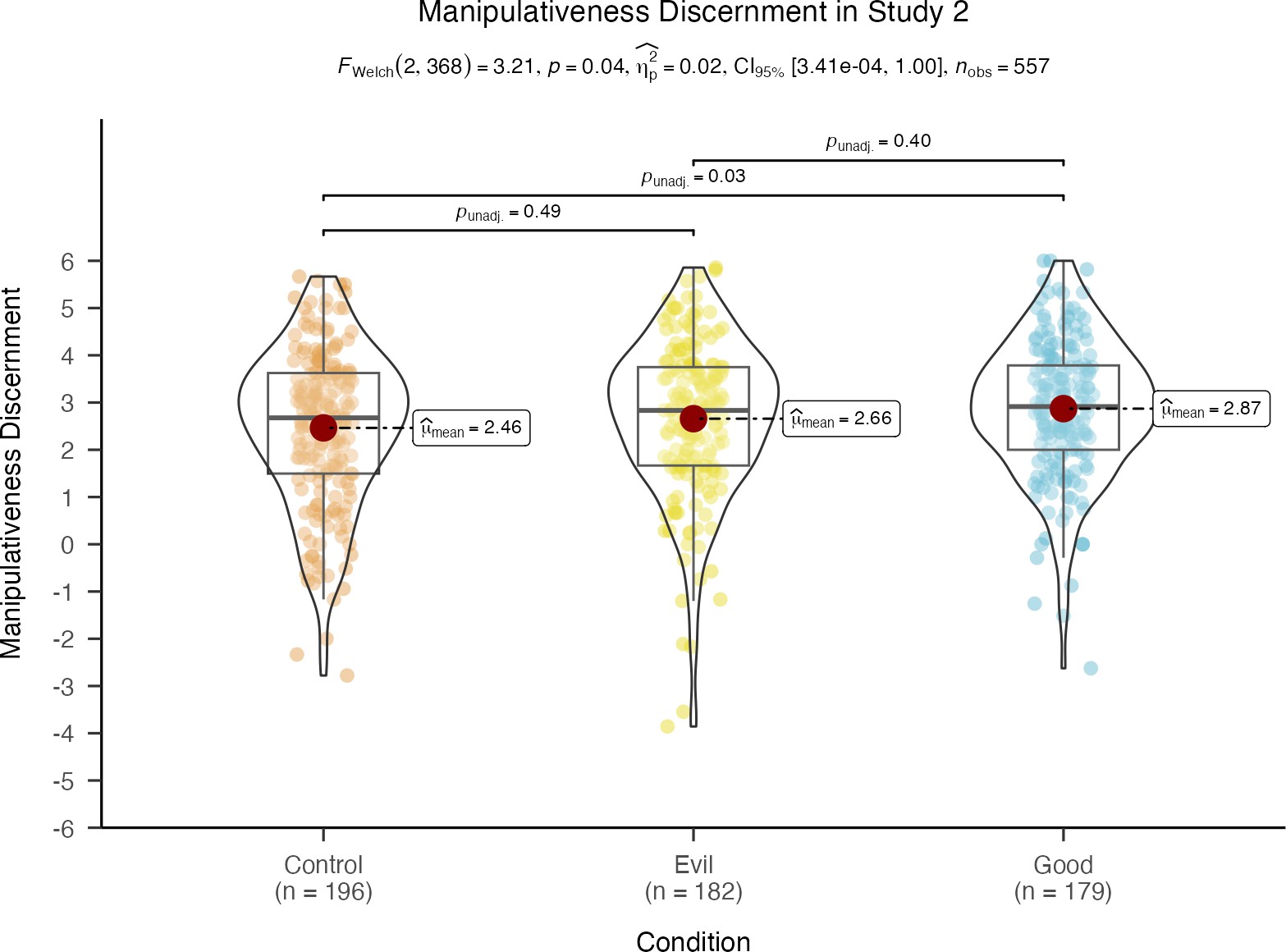


**Fig. S2.** Study 2: Overall and pairwise comparison of the effect of the *Bad Vaxx* game on manipulativeness discernment.

**5 of** [**75**](#_bookmark21)


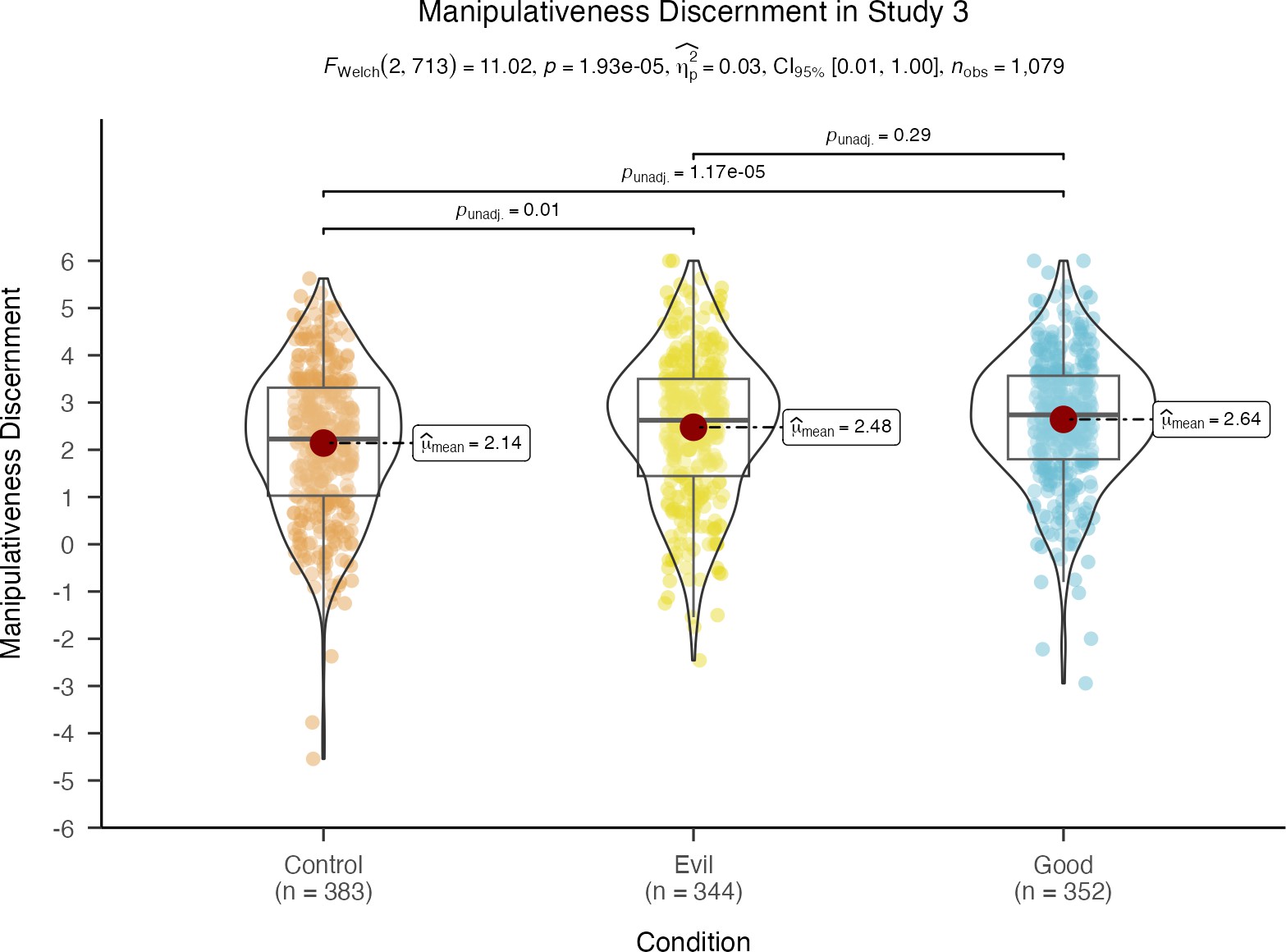


**Fig. S3.** Study 3: Overall and pairwise comparison of the effect of the *Bad Vaxx* game on manipulativeness discernment.

**6 of** [**75**](#_bookmark21)


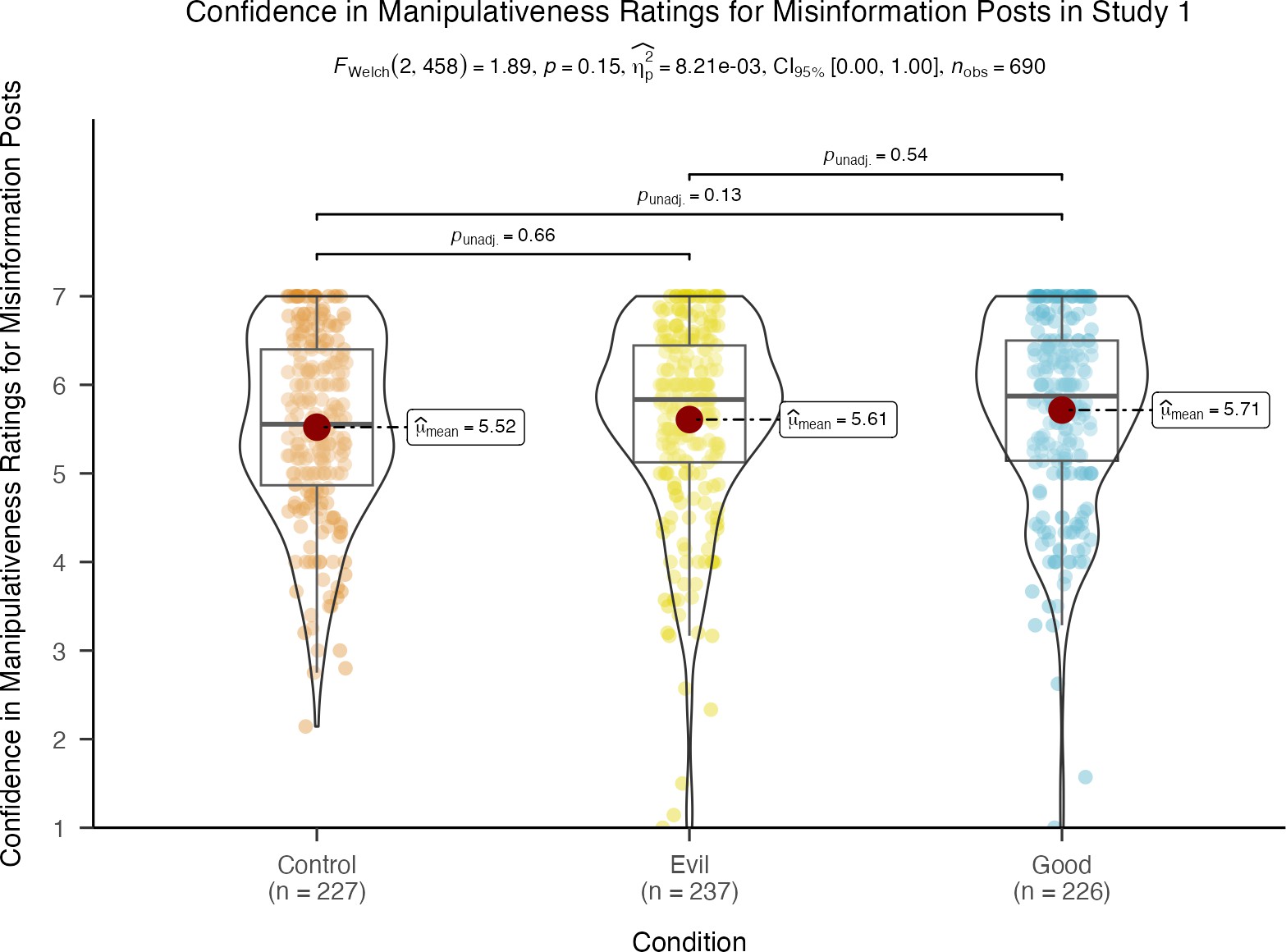


***Confidence.***

**Fig. S4.** Study 1: Overall and pairwise comparison of the effect of the *Bad Vaxx* game on confidence in manipulativeness ratings for misinformation posts.

**7 of** [**75**](#_bookmark21)


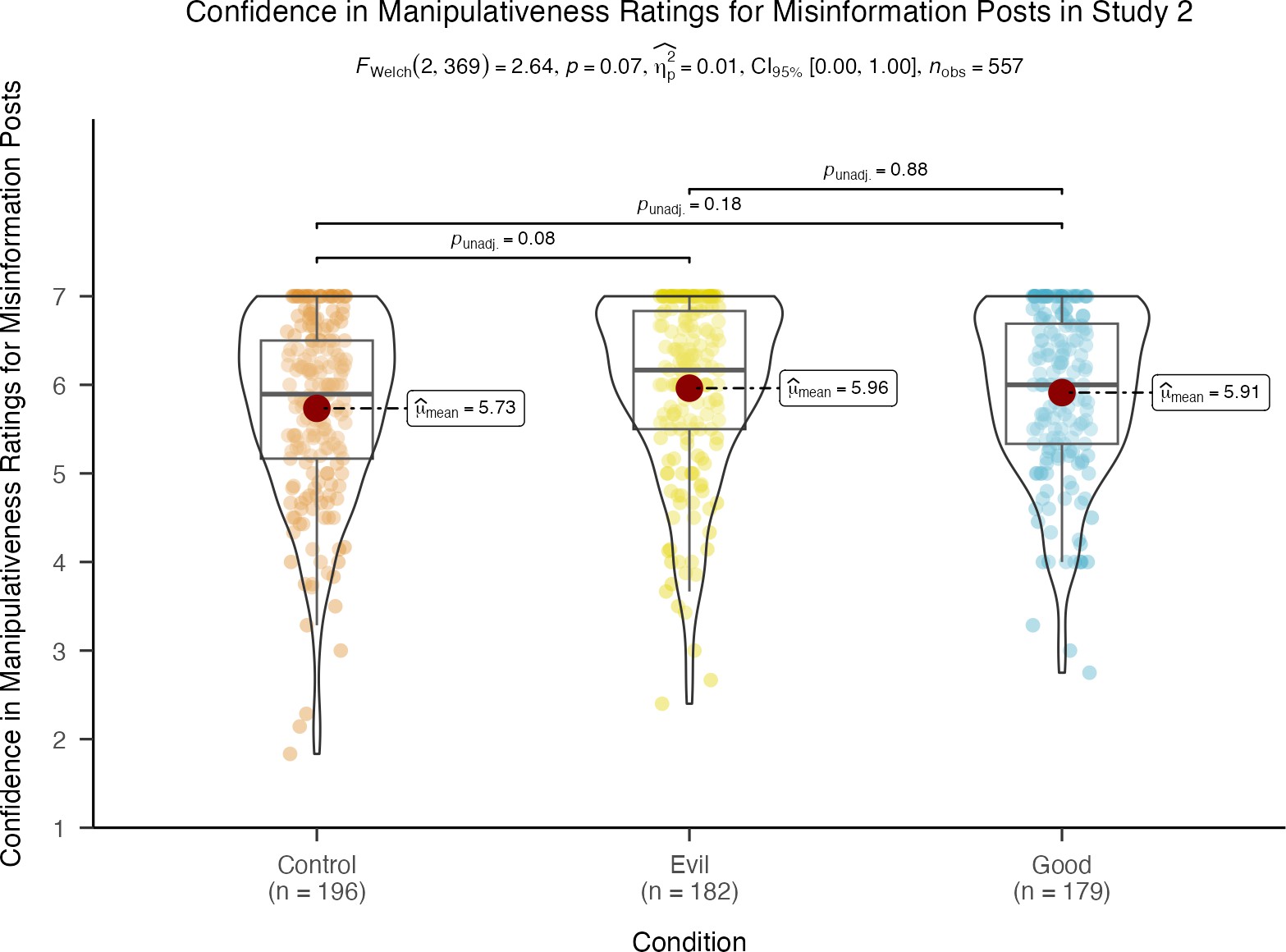


**Fig. S5.** Study 2: Overall and pairwise comparison of the effect of the *Bad Vaxx* game on confidence in manipulativeness ratings for misinformation posts.

**8 of** [**75**](#_bookmark21)


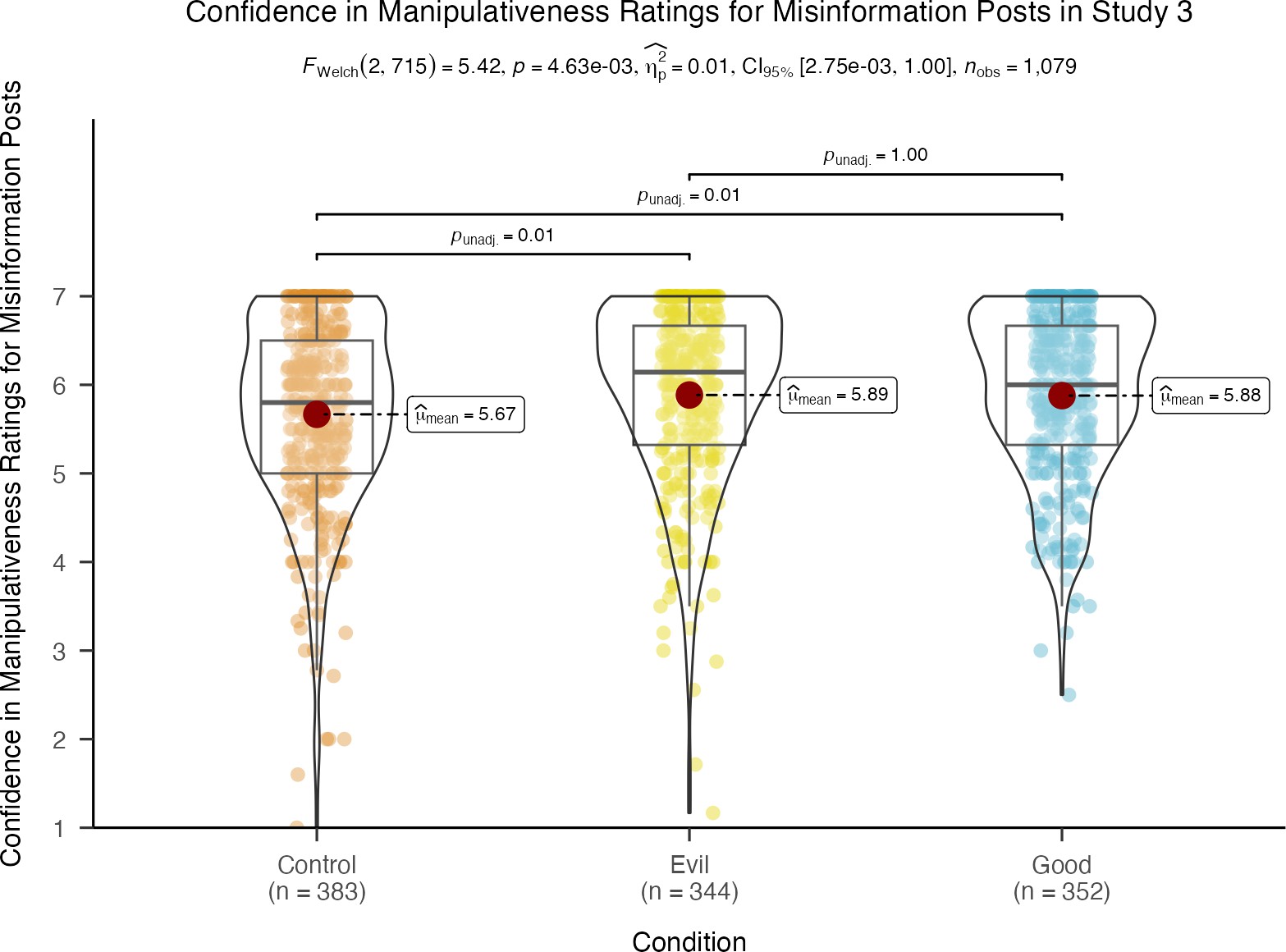


**Fig. S6.** Study 3: Overall and pairwise comparison of the effect of the *Bad Vaxx* game on confidence in manipulativeness ratings for misinformation posts.

**9 of** [**75**](#_bookmark21)


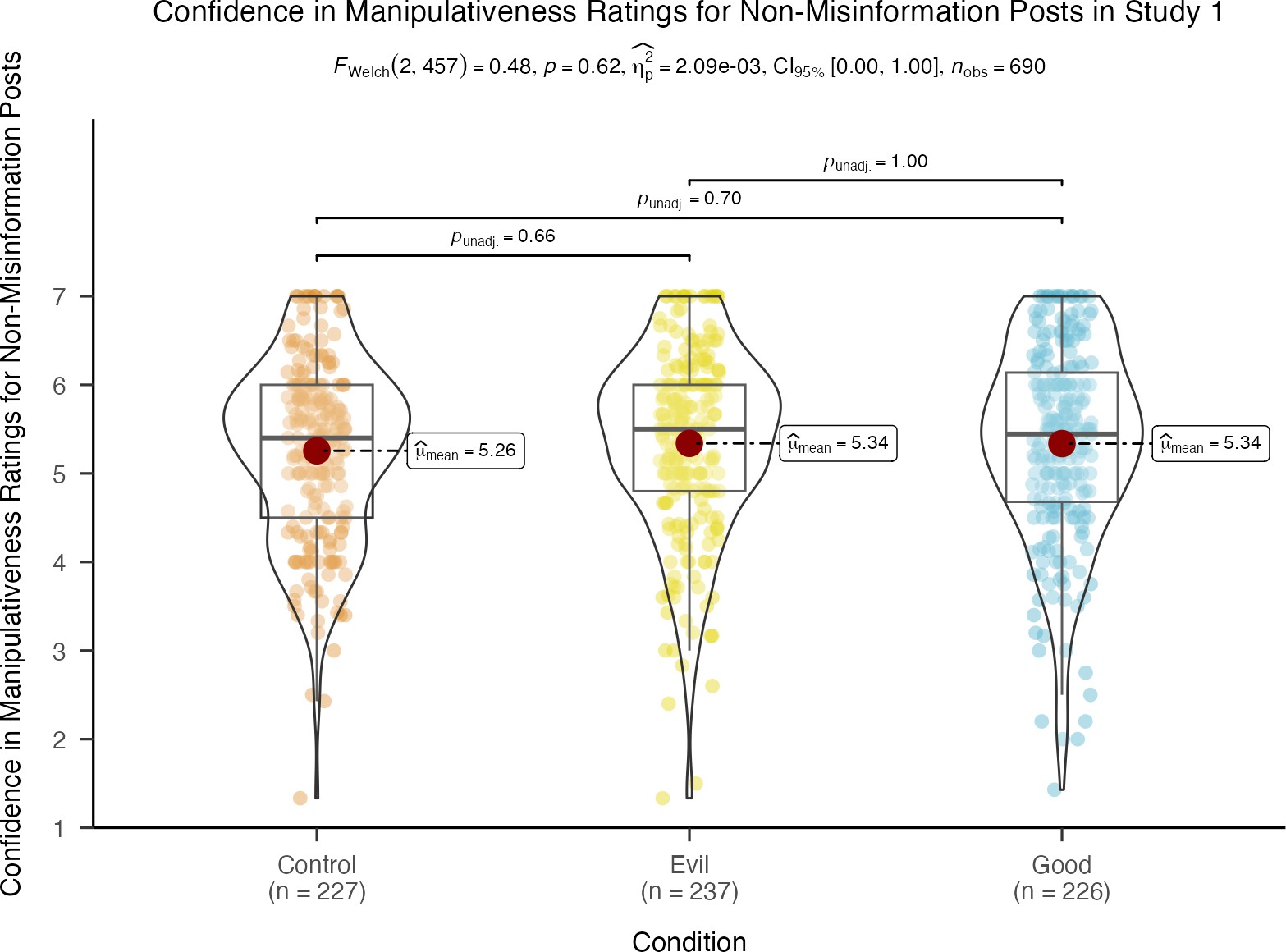


**Fig. S7.** Study 1: Overall and pairwise comparison of the effect of the *Bad Vaxx* game on confidence in manipulativeness ratings for non-misinformation posts.

**10 of** [**75**](#_bookmark21)


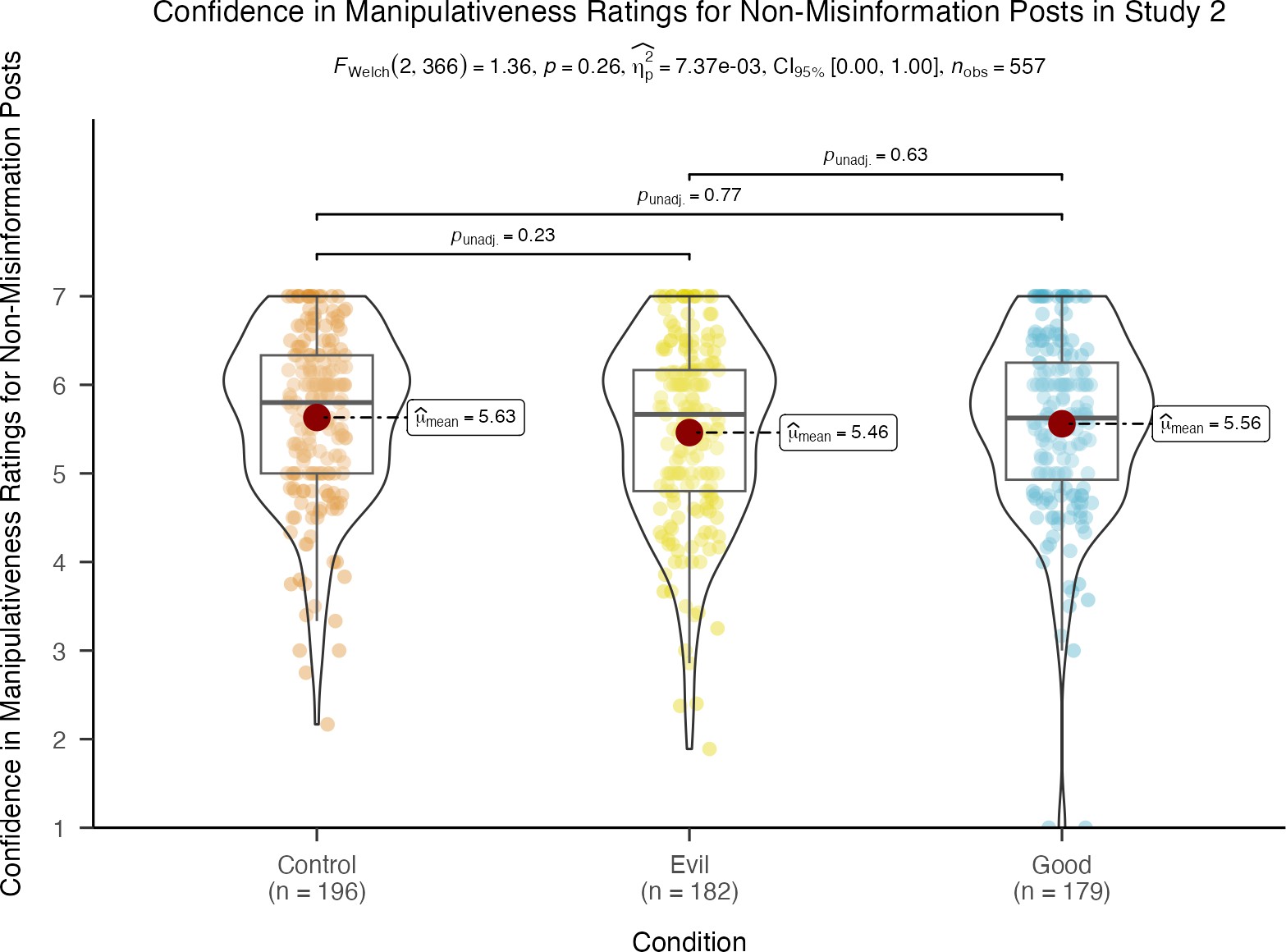


**Fig. S8.** Study 2: Overall and pairwise comparison of the effect of the *Bad Vaxx* game on confidence in manipulativeness ratings for non-misinformation posts.

**11 of** [**75**](#_bookmark21)


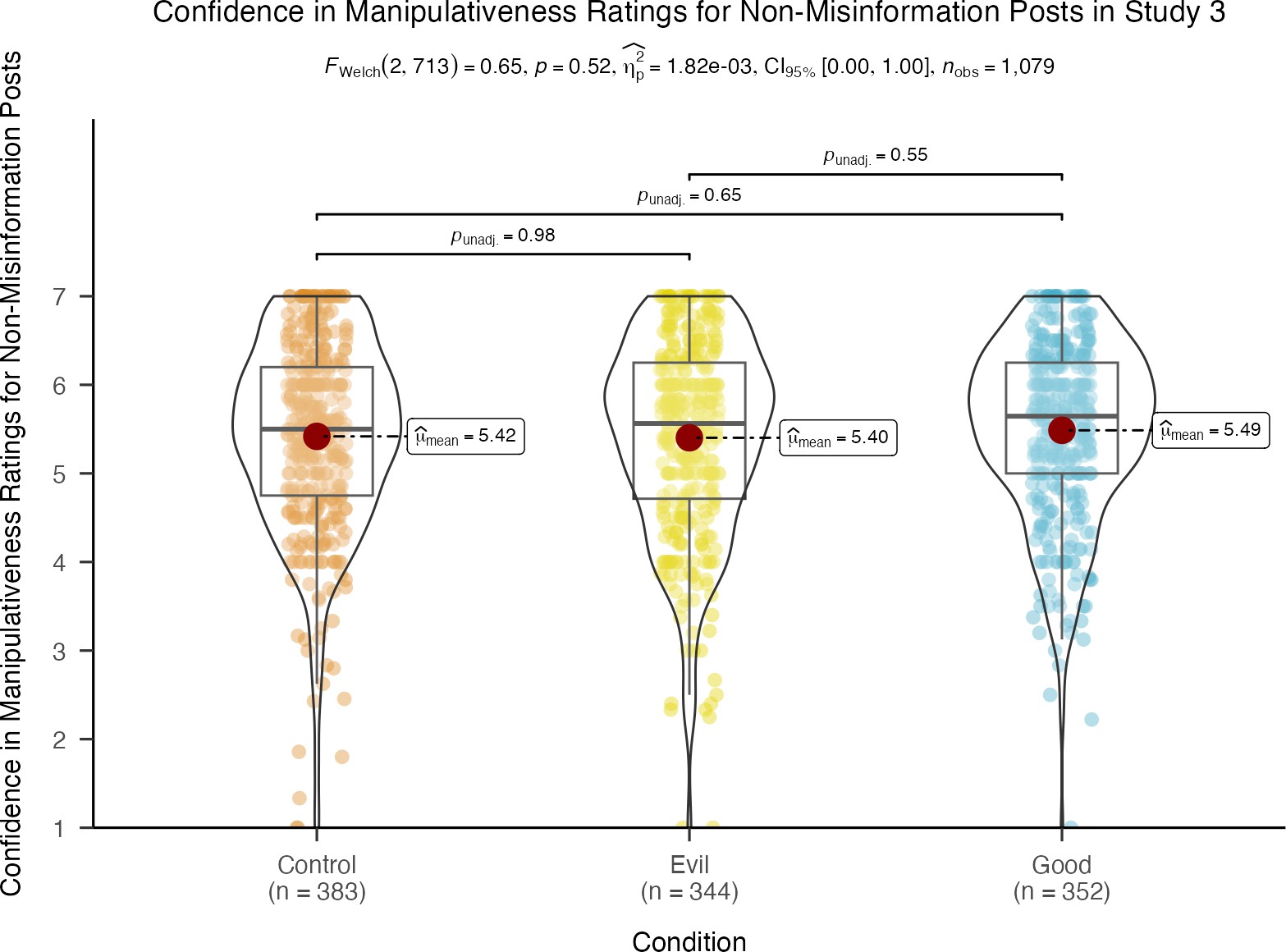


**Fig. S9.** Study 3: Overall and pairwise comparison of the effect of the *Bad Vaxx* game on confidence in manipulativeness ratings for non-misinformation posts.

**12 of** [**75**](#_bookmark21)


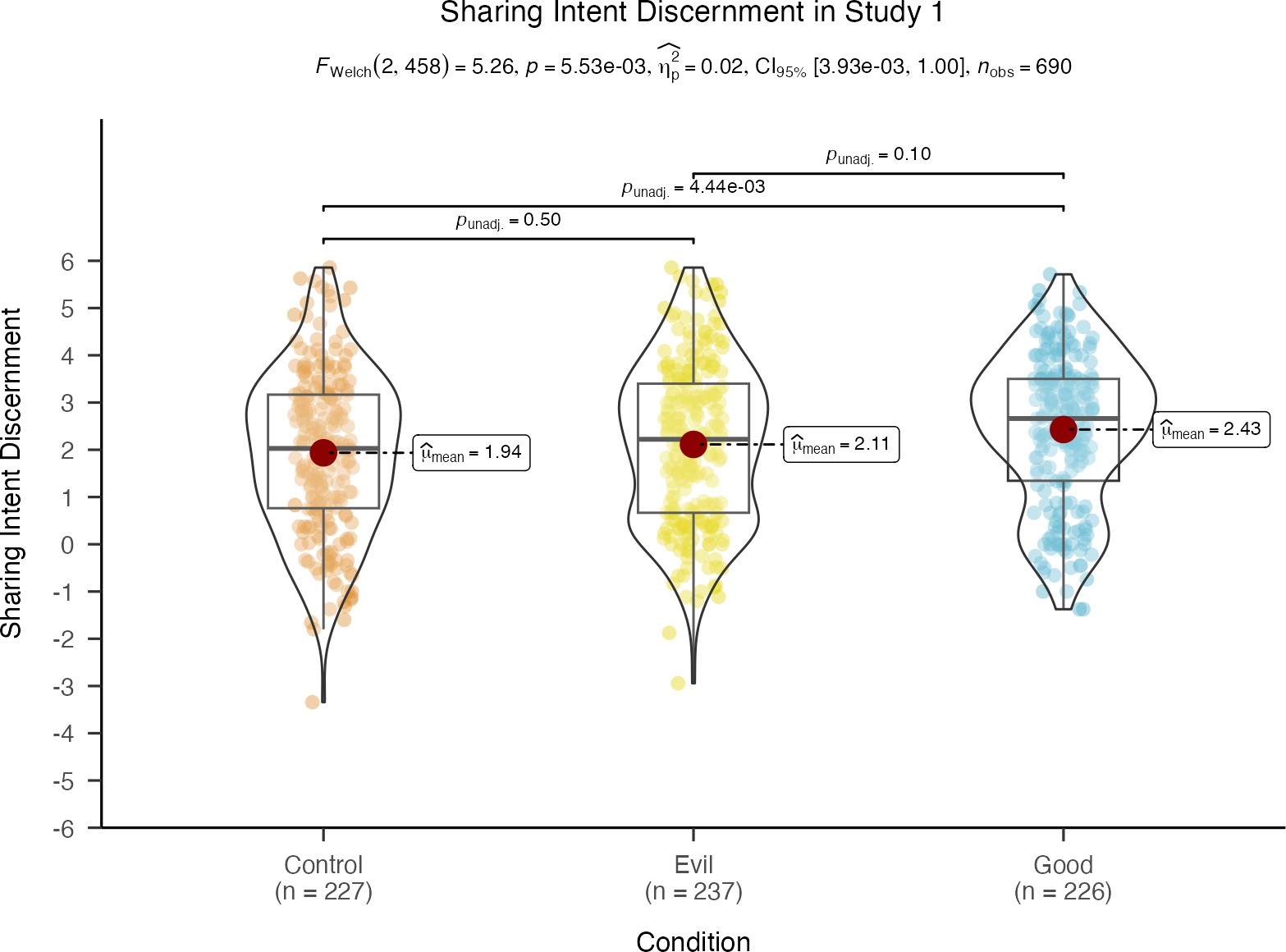


***Sharing Intent.***

**Fig. S10.** Study 1: Overall and pairwise comparison of the effect of the *Bad Vaxx* game on sharing intent discernment.

**13 of** [**75**](#_bookmark21)


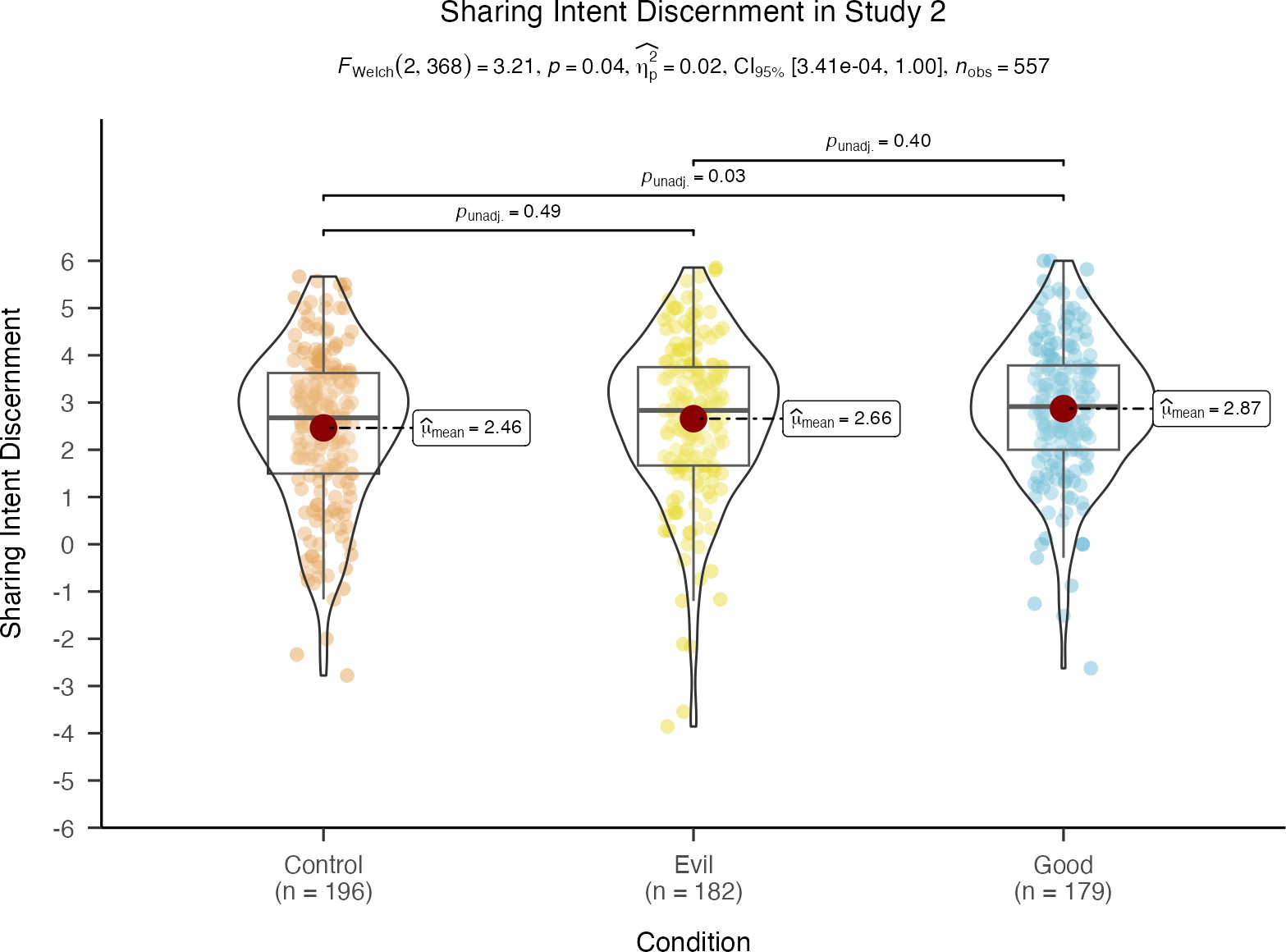


**Fig. S11.** Study 2: Overall and pairwise comparison of the effect of the *Bad Vaxx* game on sharing intent discernment.

**14 of** [**75**](#_bookmark21)


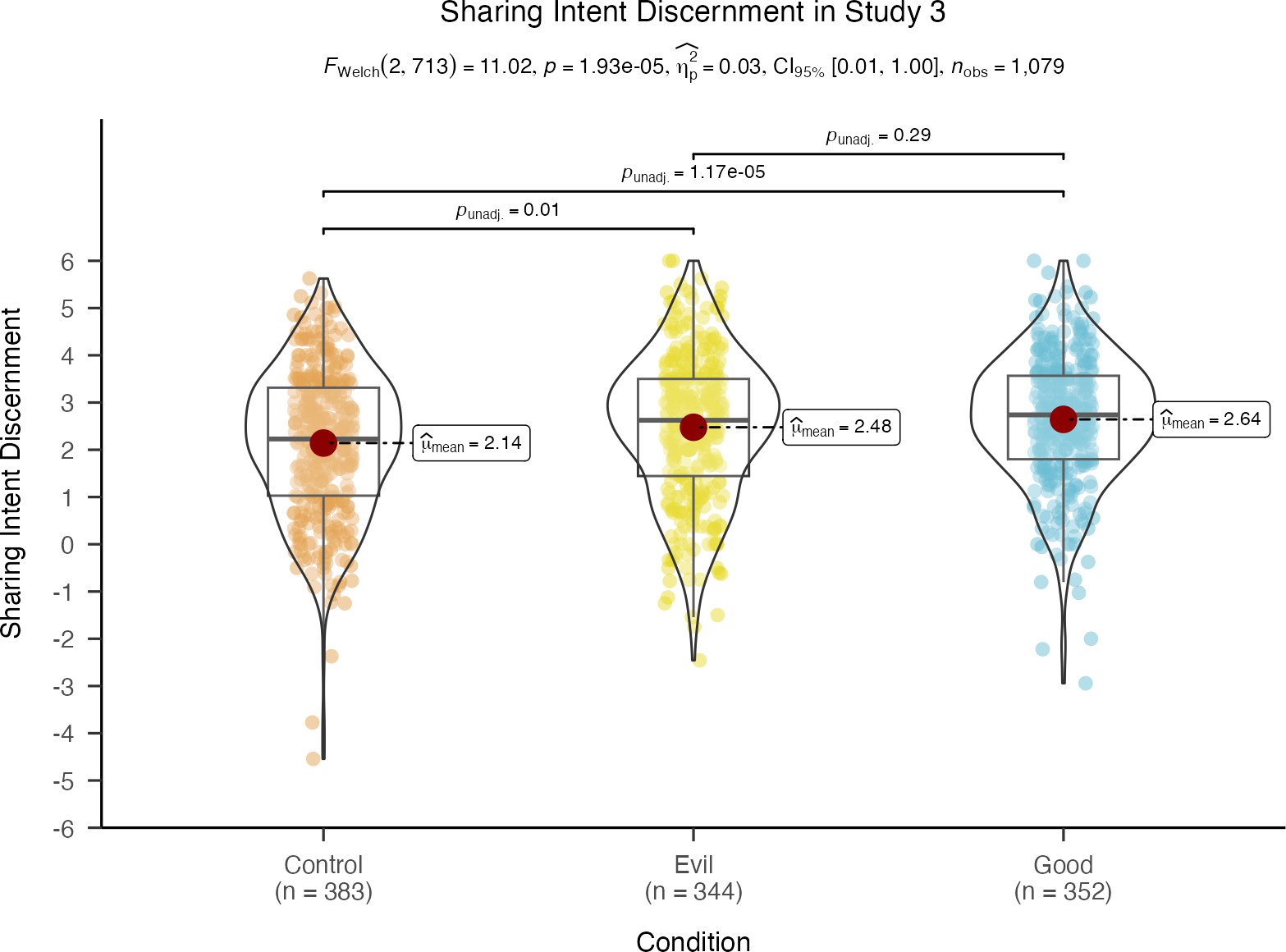


**Fig. S12.** Study 3: Overall and pairwise comparison of the effect of the *Bad Vaxx* game on sharing intent discernment.

**15 of** [**75**](#_bookmark21)


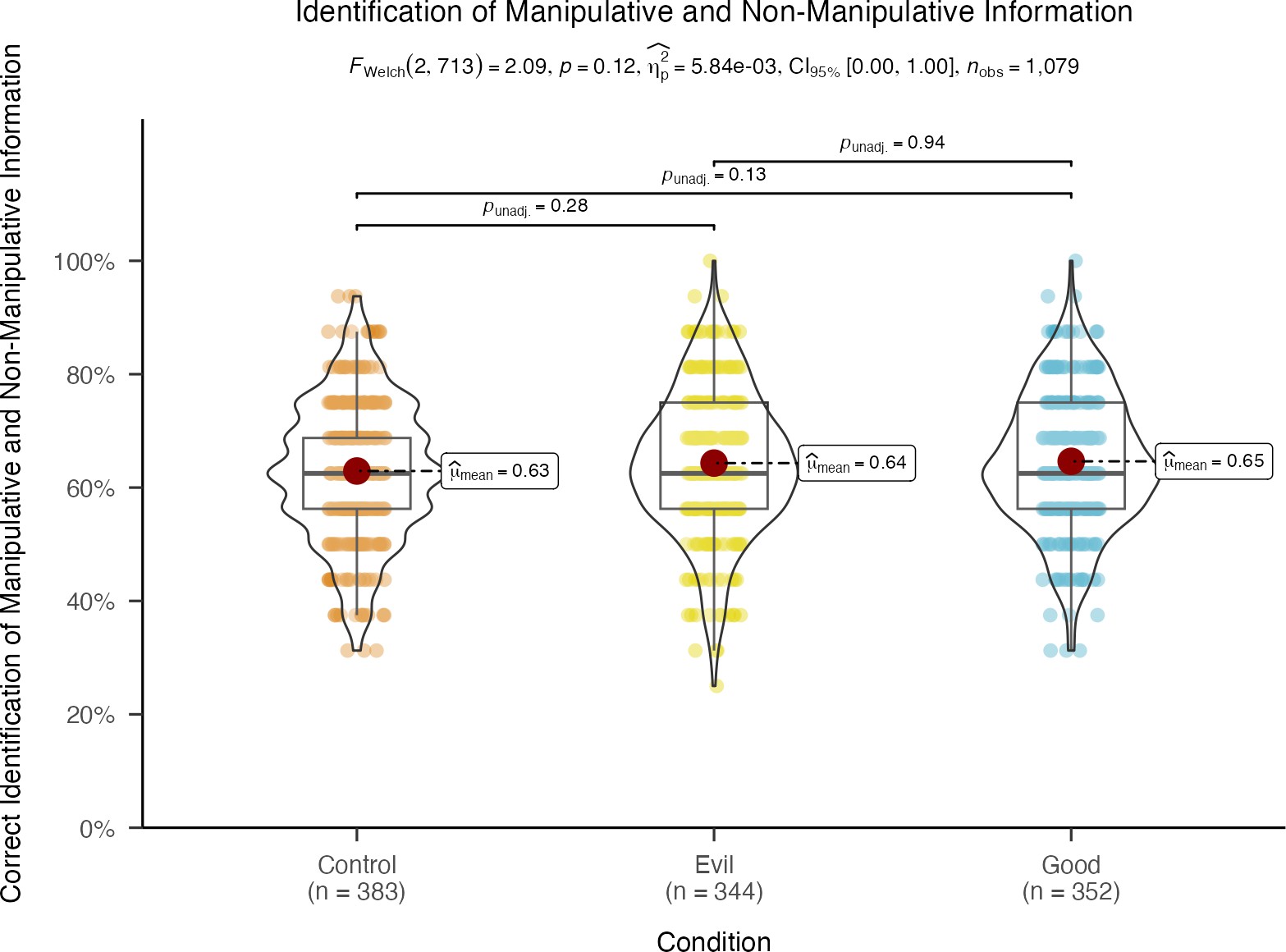


**Disentangling Identification of Truthfulness, Manipulativeness, and Manipulation Technique.**

***Correct Identification.*** This section contains the results of a preregistered exploratory analysis aiming to disentangle whether the *Bad Vaxx* game affects the identification of true vs. false information, manipulative vs. non-manipulative information, or the recognition of misinformation techniques.

**Fig. S13.** Study 3: Overall and pairwise comparison of the effect of the *Bad Vaxx* game on the correct identification of manipulative vs. non-manipulative information.

**16 of** [**75**](#_bookmark21)


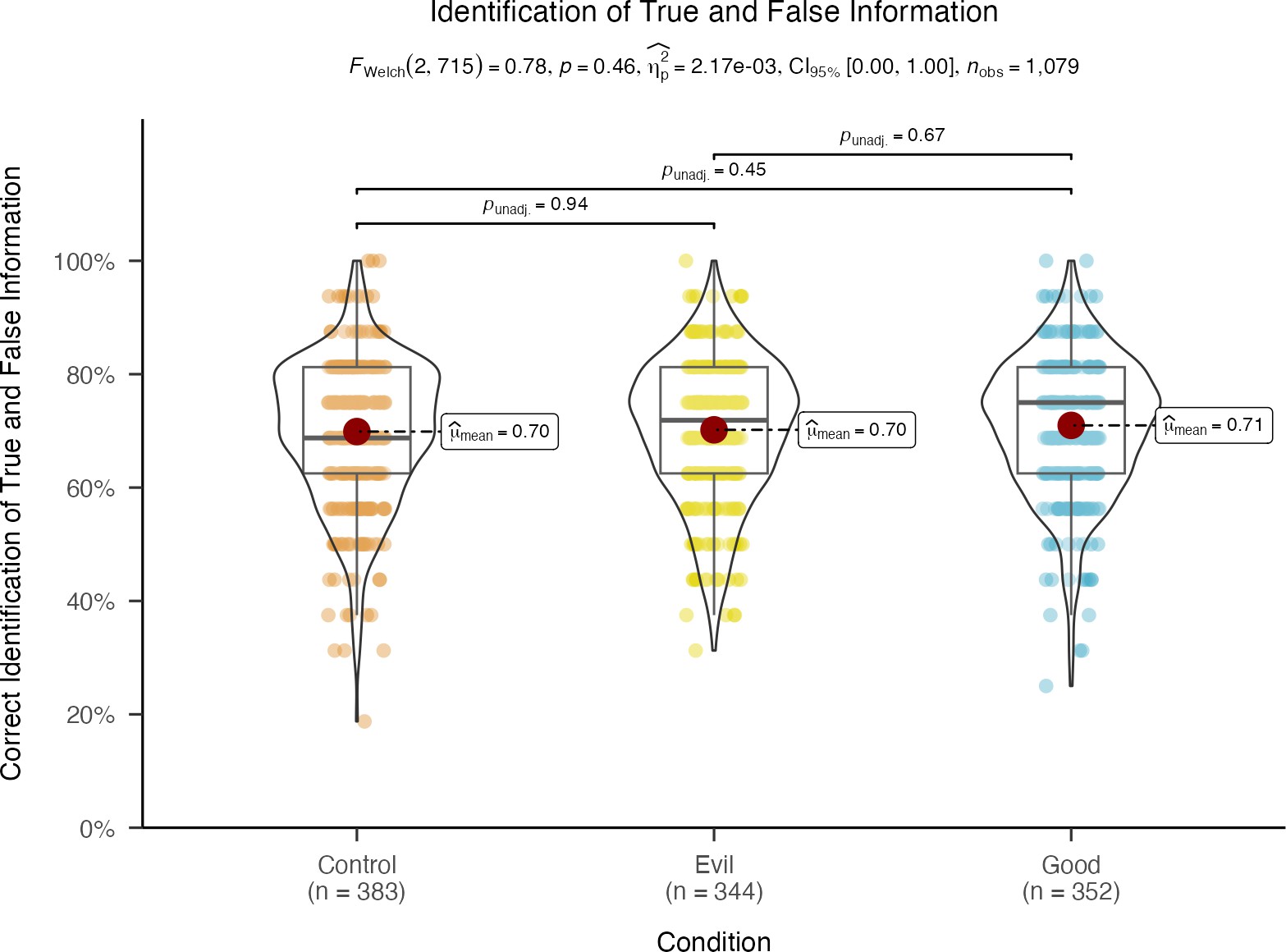


**Fig. S14.** Study 3: Overall and pairwise comparison of the effect of the *Bad Vaxx* game on the correct identification of true vs. false information.

**17 of** [**75**](#_bookmark21)


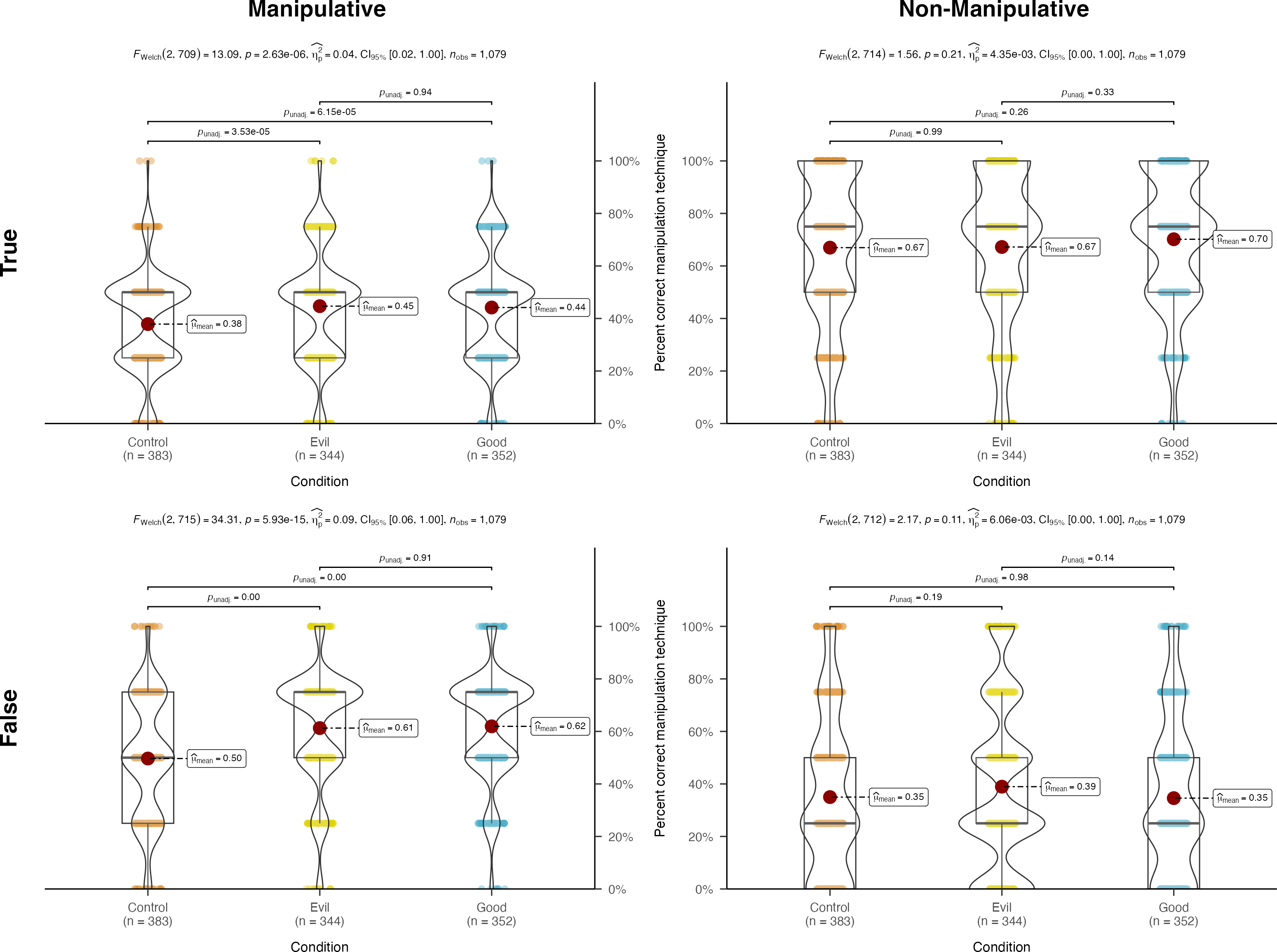


***Manipulativeness, Truthfulness, and Technique Recognition by Post Type.*** Going beyond the preregistered exploratory analysis aiming to disentangle whether the *Bad Vaxx* game affects the identification of true vs. false information, manipulative vs. non-manipulative information, or the recognition of misinformation techniques, we provide the results for effects on identification of true vs. false, manipulative vs. non manipulative, and technique recognition by post type along the dimensions manipulativeness (manipulative or non-manipulative) and truthfulness (true or false).

**Fig. S15.** Study 3: Overall and pairwise comparison of the effect of the *Bad Vaxx* game on correct technique recognition by post characteristics (manipulative vs. non-manipulative, true vs. false).

**18 of** [**75**](#_bookmark21)


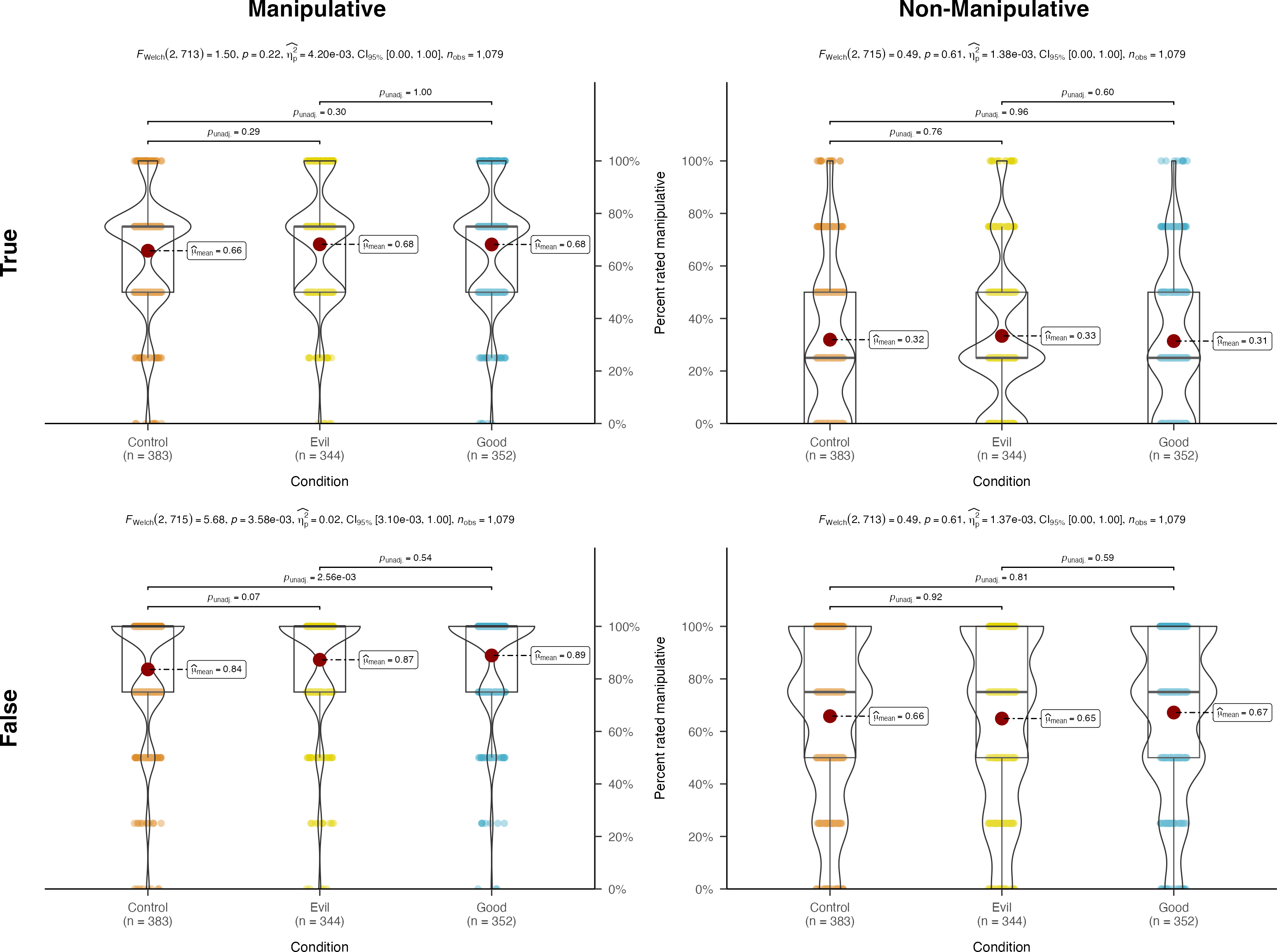


**Fig. S16.** Study 3: Overall and pairwise comparison of the effect of the *Bad Vaxx* game on the rating of social media posts as manipulative by post characteristics (manipulative vs. non-manipulative, true vs. false).

**19 of** [**75**](#_bookmark21)


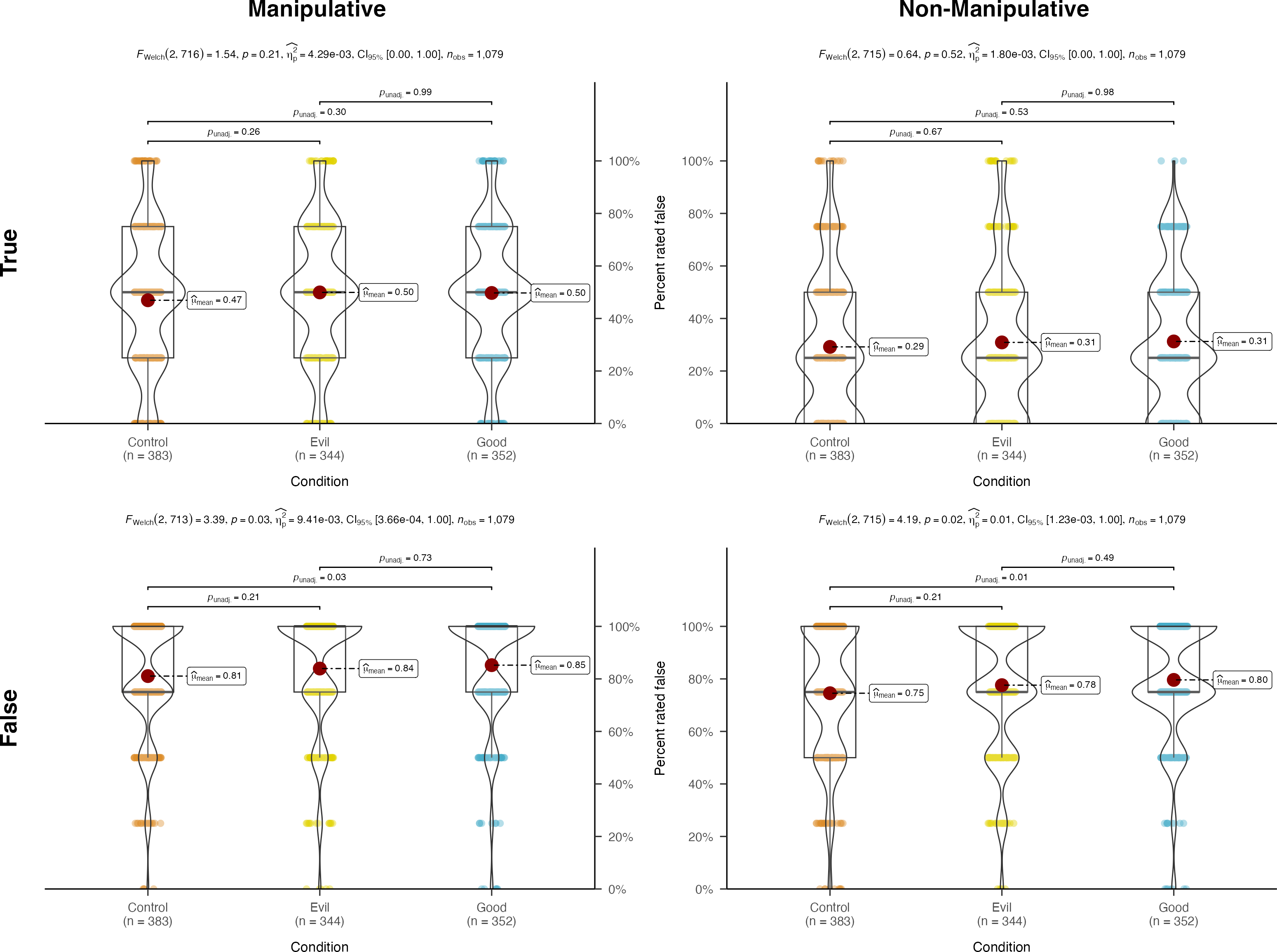


**Fig. S17.** Study 3: Overall and pairwise comparison of the effect of the *Bad Vaxx* game on the rating of social media posts as false by post characteristics (manipulative vs. non-manipulative, true vs. false).

**20 of** [**75**](#_bookmark21)

**Internal Meta-Analyses.**

***Overall Network Meta-Analysis Effects by Treatment and by Outcome.***

**Table S1. Overall Effects by Treatment and by Outcome**

Variable

Treatment

Cohen’s d

Cohen’s d SE

CI lower

CI upper

p-value

**<0.001**

Misinformation Manipulativeness

Non-Misinformation Manipulativeness Manipulativeness Discernment Misinformation Confidence

Non-Misinformation Confidence Misinformation Sharing Intent

Non-Misinformation Sharing Intent Sharing Intent Discernment Misinformation Manipulativeness

Non-Misinformation Manipulativeness Manipulativeness Discernment Misinformation Confidence

Non-Misinformation Confidence Misinformation Sharing Intent

Non-Misinformation Sharing Intent Sharing Intent Discernment

Good Version Good Version Good Version Good Version Good Version Good Version Good Version Good Version Evil Version Evil Version Evil Version Evil Version Evil Version Evil Version Evil Version Evil Version

0.33

-0.10

0.31

0.19

0.03

-0.13

0.21

0.34

0.29

0.07

0.16

0.18

-0.02

-0.01

0.09

0.11

0.05

0.05

0.05

0.05

0.05

0.05

0.05

0.05

0.05

0.05

0.05

0.05

0.05

0.05

0.05

0.05

0.23

-0.20

0.21

0.10

-0.06

-0.23

0.11

0.24

0.19

-0.03

0.06

0.08

-0.12

-0.11

-0.01

0.01

0.43

0.00

0.41

0.29

0.13

-0.03

0.31

0.44

0.39

0.17

0.26

0.28

0.08

0.09

0.19

0.20

0.052

**<0.001**

**<0.001**

0.492

**0.012**

**<0.001**

**<0.001**

**<0.001**

0.160

**0.001**

**<0.001**

0.641

0.852

0.073

**0.037**

*Note.* Results are based on a network meta-analysis using fixed effects for Studies 1, 2 and 3. By default standard errors for this type of analysis are adjusted for the correlation between the different comparisons in multi-arm studies.

**21 of** [**75**](#_bookmark21)

***Pairwise Meta-Analyses by Outcome.***

Below, we provide the full set of forest plots for the meta-analyses, one for each outcome. The plots below show the pairwise meta-analyses results across Studies 1 to 3 by treatment and outcome variable. We used the meta ([1](#_bookmark22)) and netmeta ([2](#_bookmark23)) packages to conduct the analyses and produce the plots, and by default standard errors for the pairwise analyses are unadjusted.

***Manipulativeness.*** For the misinformation items, pairwise meta-analyses show that both the “good” (Cohen’s *d* = 0*.*33*, SE* = 0*.*05*, p <* 0*.*001) and the “evil” version of the game (Cohen’s *d* = 0*.*29*, SE* = 0*.*05*, p <* 0*.*001) have a significant effect across Studies 1 to 3 compared to the control group. The pairwise comparison between the “good” and the “evil” version is nonsignificant. This supports **H1a**: The *Bad Vaxx* game increases players’ ability to identify manipulative social media content about vaccines.

With respect to non-misinformation, the meta-analyses show that the effect of both the “good” and the “evil” version of the Bad Vaxx game is nonsignificant compared to the control group. Participants playing the “evil” version rate non-misinformation as significantly more manipulative compared to participants playing the “good” version (Cohen’s *d* = 0*.*17*, SE* = 0*.*05*, p <* 0*.*001). For discernment, that is, the difference between manipulativeness ratings for manipulative and non-manipulative content (see Figure **??**), the meta-analyses show that both the “good” (Cohen’s *d* = 0*.*31*, SE* = 0*.*05*, p <* 0*.*001) and the “evil” version (Cohen’s *d* = 0*.*16*, SE* = 0*.*05*, p* = 0*.*002) have significantly higher discernment than the control group. Further, participants playing the “evil” version have significantly lower discernment than those playing the “good” version (Cohen’s *d* = *−*0*.*14*, SE* = 0*.*05*, p* = 0*.*006). This supports **H1b**: Playing the *Bad Vaxx* game increases players’ ability to discern manipulative from

non-manipulative vaccine information.

***Confidence.*** Pairwise meta-analyses show that both the “good” (Cohen’s *d* = 0*.*2*, SE* = 0*.*05*, p <* 0*.*001) and the “evil” version of the game (Cohen’s *d* = 0*.*18*, SE* = 0*.*05*, p <* 0*.*001) have a significant and positive effect on confidence in manipulativeness ratings for vaccine misinformation across Studies 1 to 3 compared to the control group. The pairwise comparison between the “good” and the “evil” version is nonsignificant. Confidence in manipulativeness ratings for non-misinformation does not differ significantly between treatment and control groups. These results support **H2**: Participants playing the *Bad Vaxx* game have greater confidence in their assessment of the manipulativeness of vaccine misinformation.

***Sharing Intent.*** For the misinformation items, pairwise meta-analyses show that the “good” version of the *Bad Vaxx* game has a significant negative effect on willingness to share compared to the control condition (Cohen’s *d* = *−*0*.*13*, SE* = 0*.*05*, p* = 0*.*012), but the effect of the “evil” version compared to the control condition is nonsignificant. Further, participants playing the “evil” version are significantly more willing to share misinformation than those playing the “good” version (Cohen’s *d* = 0*.*12*, SE* = 0*.*05*, p* = 0*.*022). This partially supports hypothesis **H3a**, namely that the game reduces willingness to share vaccine misinformation, notably only for the “good” version of the game.

With respect to non-misinformation, the meta-analyses show that participants playing the “good” version are significantly more willing to share non-misinformation than those in the control condition (Cohen’s *d* = 0*.*21*, SE* = 0*.*05*, p <* 0*.*001), while there is no effect for participants playing the “evil” version compared to the control condition. Participants playing the “evil” version have significantly lower willingness to share non-misinformation compared to those playing the “good” version (Cohen’s *d* = *−*0*.*12*, SE* = 0*.*05*, p* = 0*.*018).

In terms of discernment, that is, the difference between intent to share non-misinformation and intent to share misinformation (see Figure **??**), the meta-analyses show that the “good” version (Cohen’s *d* = 0*.*34*, SE* = 0*.*05*, p <* 0*.*001) and the “evil” version (Cohen’s *d* = 0*.*1*, SE* = 0*.*05*, p* = 0*.*043) of the game significantly increase discernment in willingness to share compared to the control condition. Further, participants playing the “evil” version have significantly lower discernment in willingness to share compared to those playing the “good” version (Cohen’s *d* = *−*0*.*23*, SE* = 0*.*05*, p <* 0*.*001). This supports hypothesis **H3b**: Playing the *Bad Vaxx* game increases the quality of people’s sharing decisions, in the sense that they have a higher difference in willingness to share non-manipulative versus manipulative information about vaccines.

**22 of** [**75**](#_bookmark21)


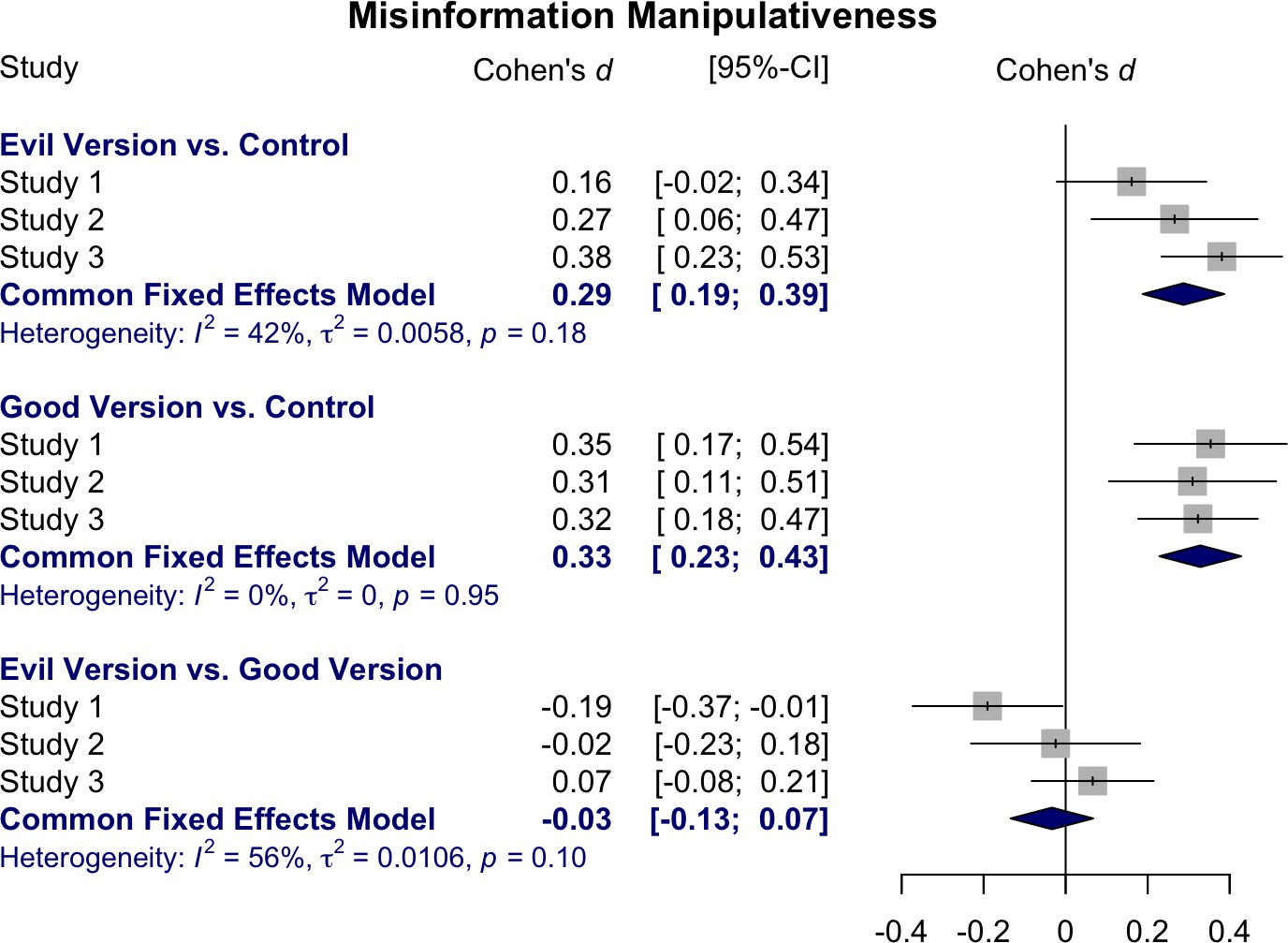


**Misinformation Manipulativeness**

**Fig. S18.** Forest plot for a pairwise meta-analysis of the Misinformation Manipulativeness measure across Studies 1 to 3.

**23 of** [**75**](#_bookmark21)


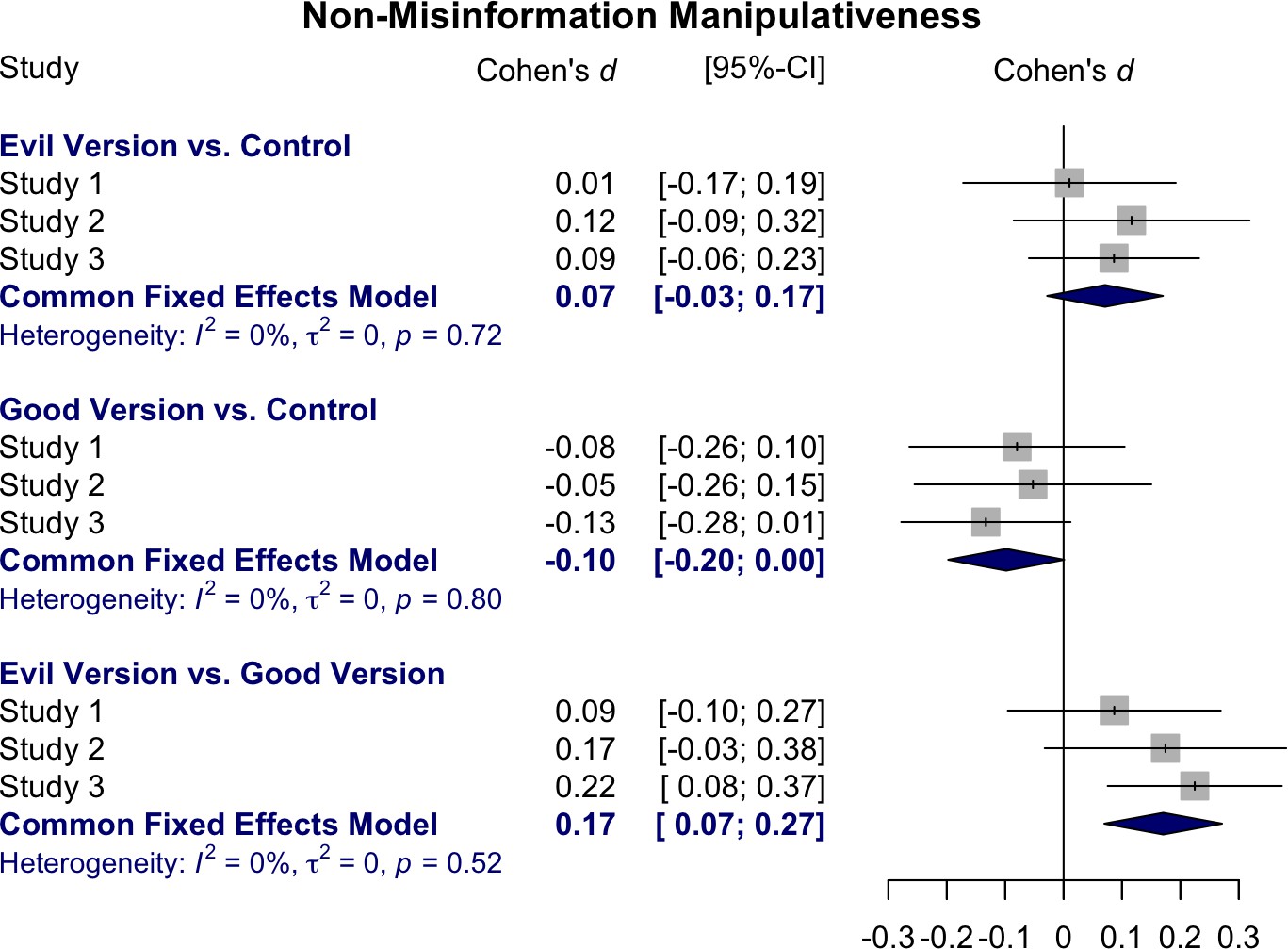


**Non-Misinformation Manipulativeness**

**Fig. S19.** Forest plot for a pairwise meta-analysis of the Non-Misinformation Manipulativeness measure across Studies 1 to 3.

**24 of** [**75**](#_bookmark21)


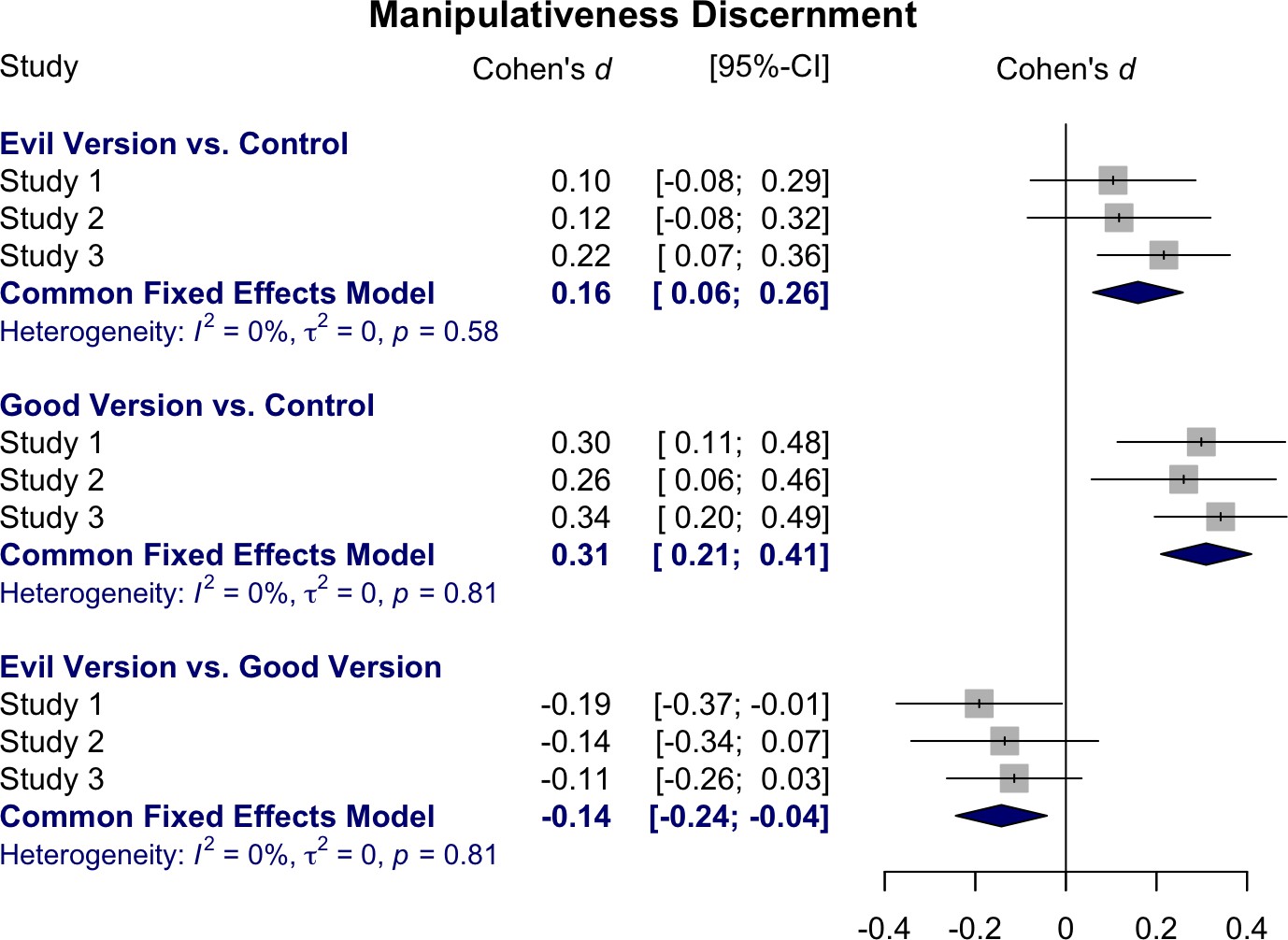


**Manipulativeness Discernment**

**Fig. S20.** Forest plot for a pairwise meta-analysis of the Manipulativeness Discernment measure across Studies 1 to 3.

**25 of** [**75**](#_bookmark21)


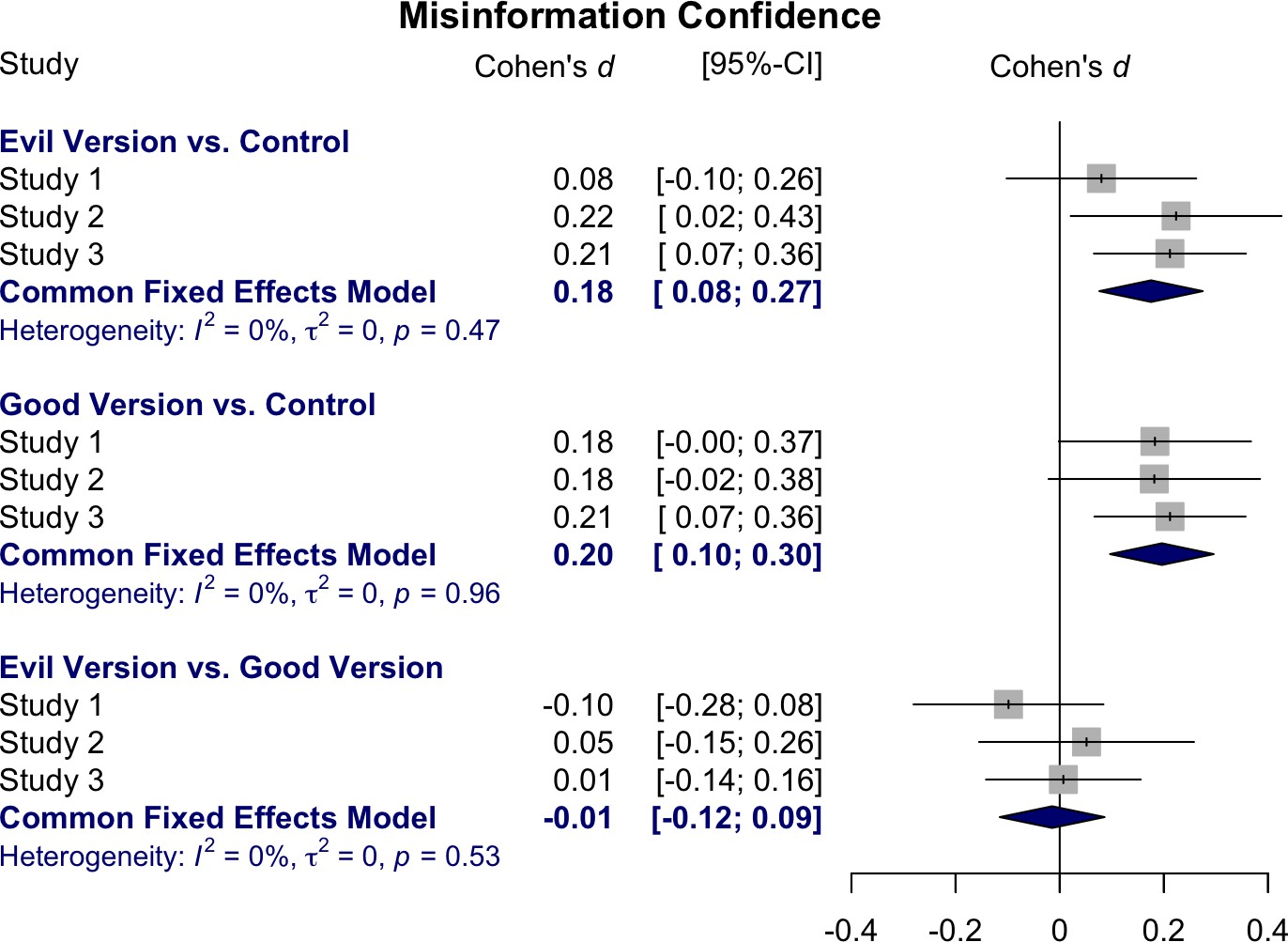


**Misinformation Confidence**

**Fig. S21.** Forest plot for a pairwise meta-analysis of the Misinformation Confidence measure across Studies 1 to 3.

**26 of** [**75**](#_bookmark21)


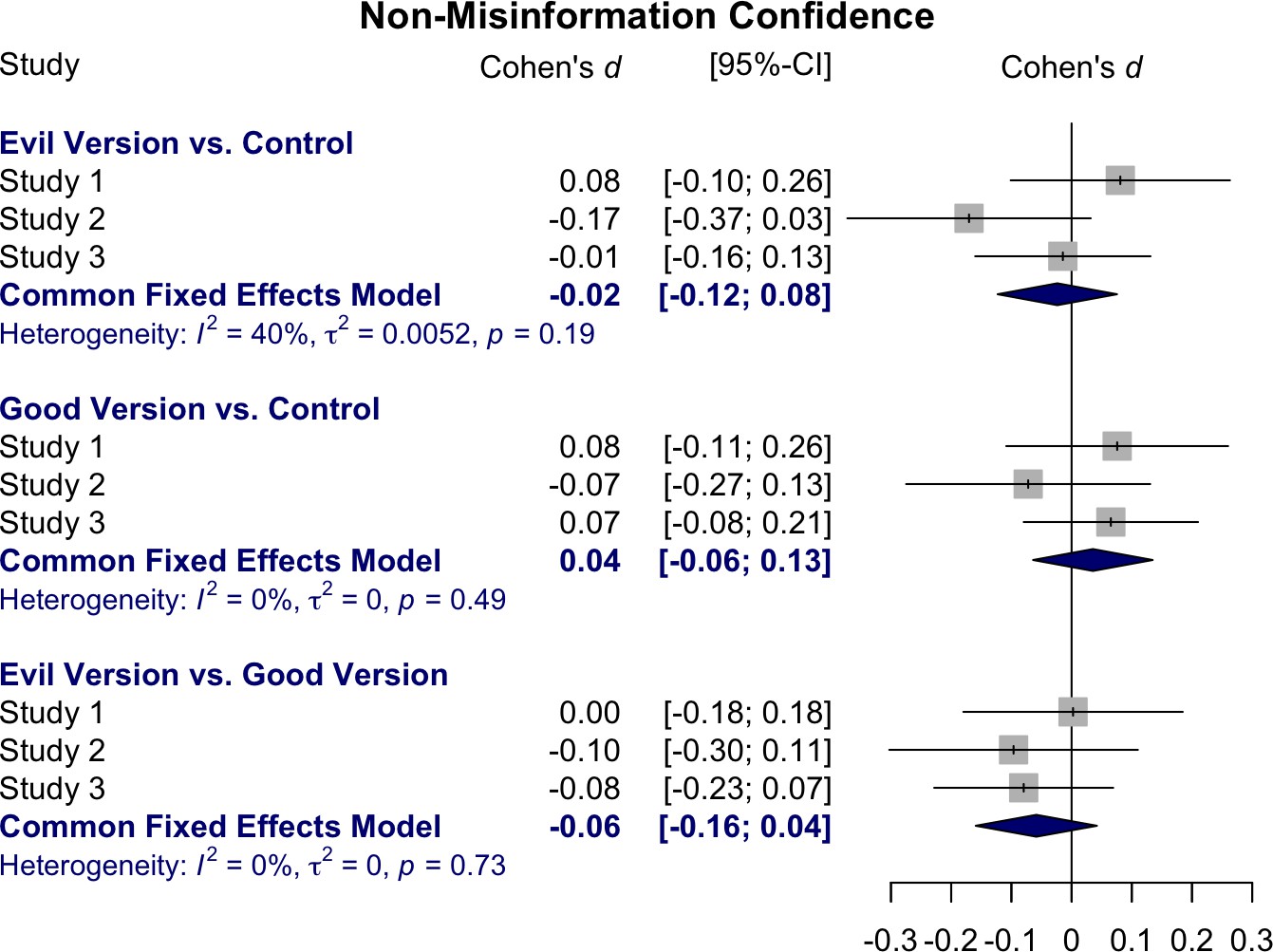


**Non-Misinformation Confidence**

**Fig. S22.** Forest plot for a pairwise meta-analysis of the Non-Misinformation Confidence measure across Studies 1 to 3.

**27 of** [**75**](#_bookmark21)


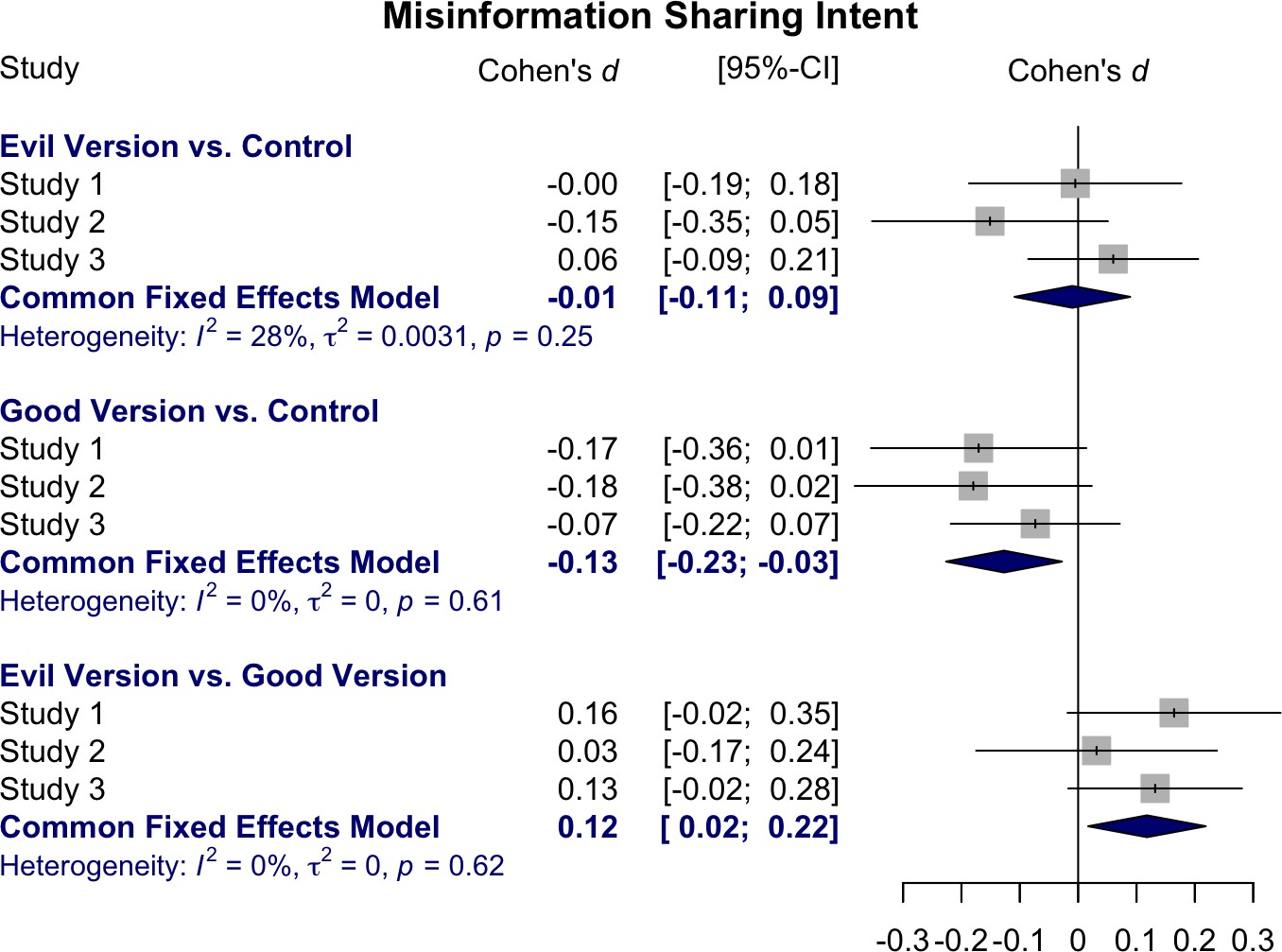


**Misinformation Sharing**

**Fig. S23.** Forest plot for a pairwise meta-analysis of Misinformation Sharing measure across Studies 1 to 3.

**28 of** [**75**](#_bookmark21)


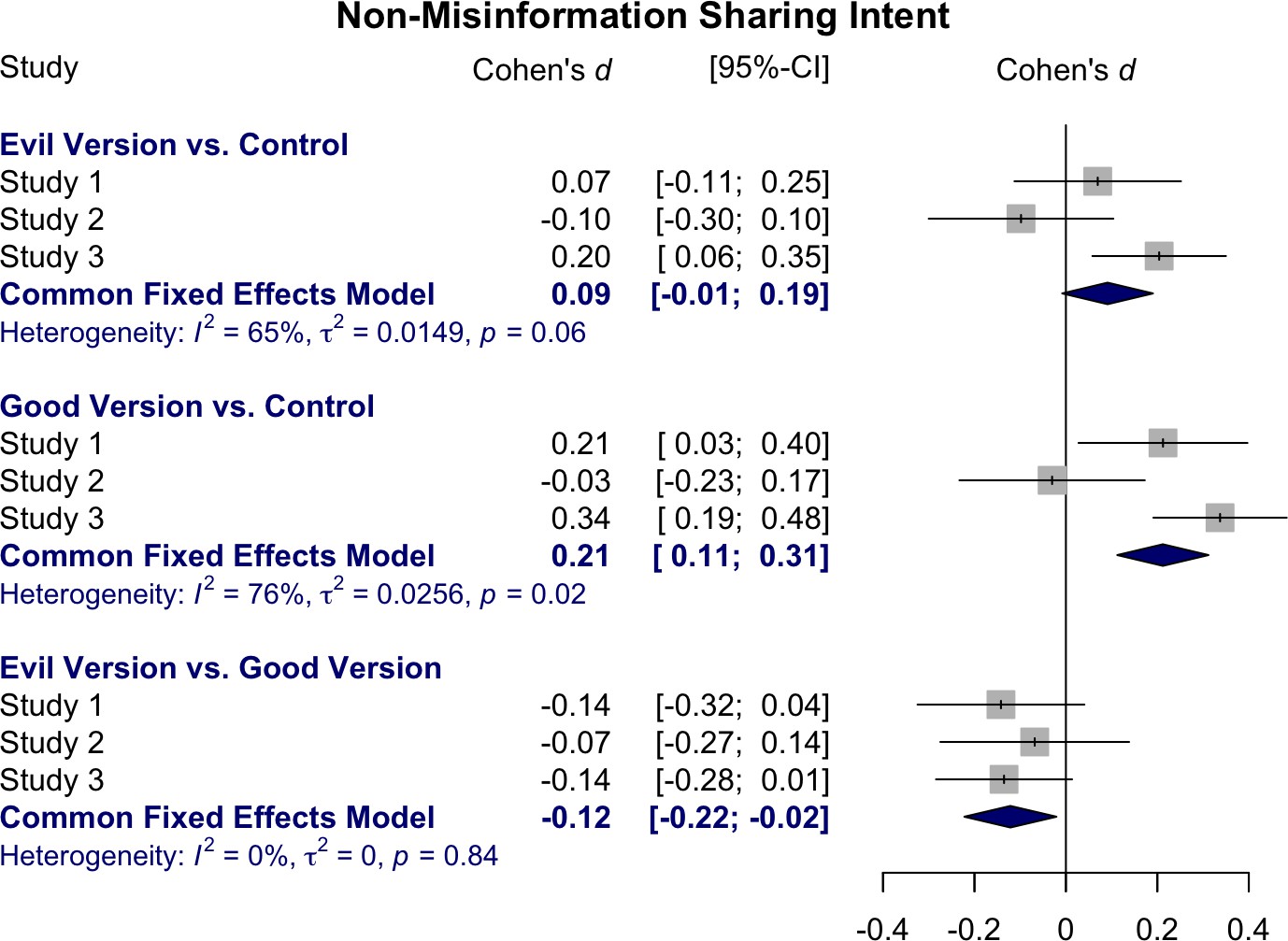


**Non-Misinformation Sharing**

**Fig. S24.** Forest plot for a pairwise meta-analysis of the Non-Misinformation Sharing measure across Studies 1 to 3.

**29 of** [**75**](#_bookmark21)


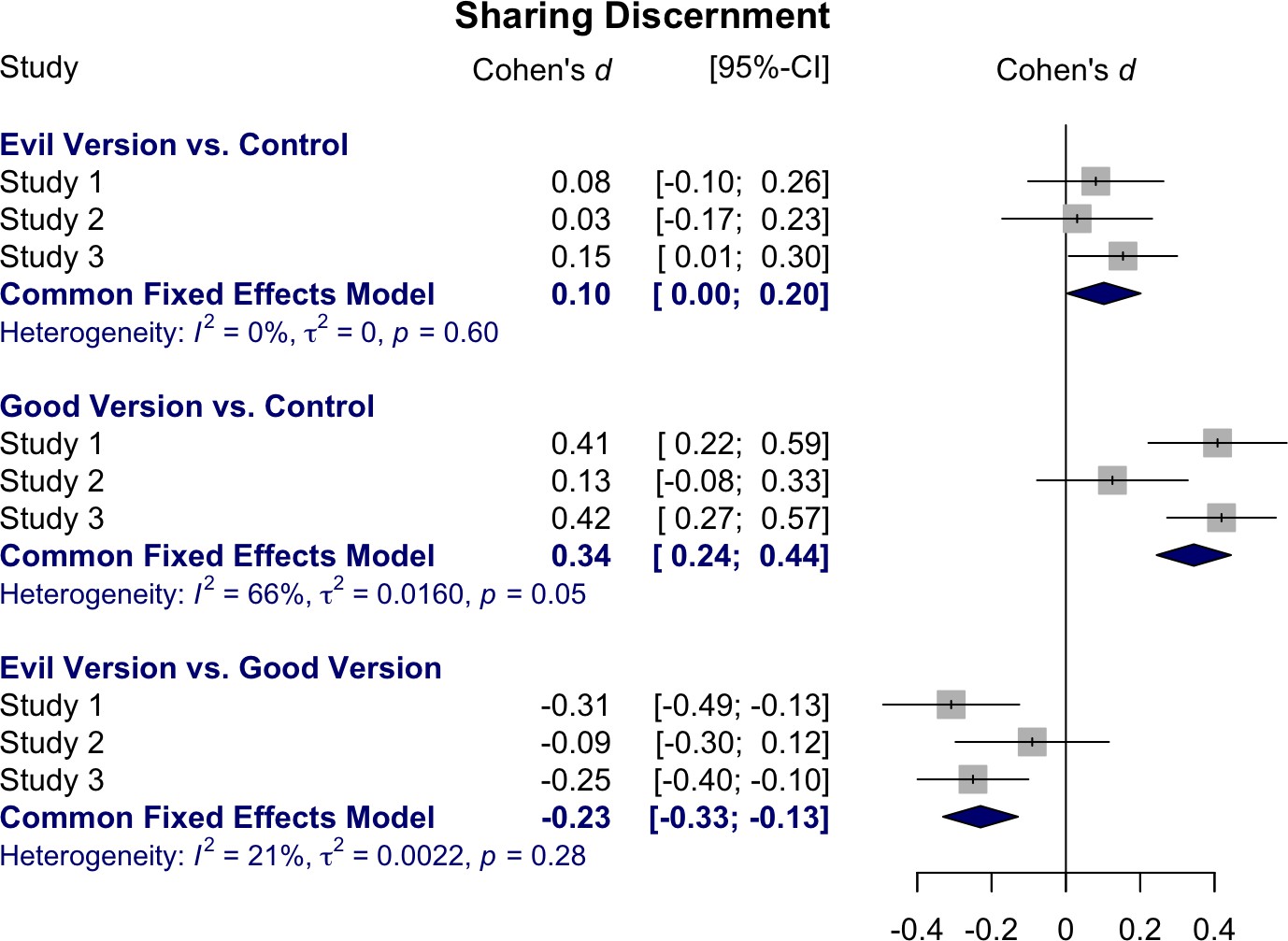


**Sharing Discernment**

**Fig. S25.** Forest plot for a pairwise meta-analysis of the Sharing Discernment measure across Studies 1 to 3.

**30 of** [**75**](#_bookmark21)

**Interaction Between Condition and Covariates**

This section contains the results for non-preregistered analyses on the interaction between condition and the covariates COVID-19 vaccine intentions and political ideology.

**COVID-19 Vaccine Intentions.**

**Table S2. Linear Regression Model for Manipulativeness Ratings (Fake Items Only) Including Interaction Between Condition and COVID-Vaccine Intentions**

Study 1

**5***.***15***∗∗∗*

Study 2

**4***.***84***∗∗∗*

Study 3

**3***.***58***∗∗∗*

(Intercept) COVID.Vaccine.Intentions ConditionEvil ConditionGood

Having.Children

(0*.*31)

**0***.***53***∗∗*

(0*.*17)

*−*0*.*00

(0*.*19) **0***.***52***∗* (0*.*21)

*−***0***.***33***∗∗∗*

(0*.*10)

*−*0*.*10

(0*.*08)

0*.*21

(0*.*30)

*−*0*.*01

(0*.*13) **0***.***22***∗* (0*.*11)

0*.*05

(0*.*04)

*−*0*.*00

(0*.*05)

0*.*06

(0*.*05)

*−***0***.***13***∗∗*

(0*.*04)

0*.*30

(0*.*22)

*−*0*.*13

(0*.*24)

(0*.*37)

**0***.***92***∗∗∗*

(0*.*20)

0*.*34

(0*.*24)

0*.*42

(0*.*25)

*−***0***.***32***∗∗*

(0*.*10)

*−***0***.***27***∗∗*

(0*.*09)

0*.*23

(0*.*28)

0*.*02

(0*.*15)

0*.*20

(0*.*13)

0*.*08

(0*.*04)

*−*0*.*03

(0*.*04)

0*.*03

(0*.*06)

*−*0*.*05

(0*.*06)

0*.*02

(0*.*27)

*−*0*.*12

(0*.*27)

(0*.*24)

**1***.***03***∗∗∗*

(0*.*15)

**0***.***76***∗∗∗* (0*.*18) **0***.***46***∗* (0*.*19)

*−*0*.*06

(0*.*07)

0*.*07

(0*.*06)

*−*0*.*01

(0*.*21)

0*.*10

(0*.*10)

**0***.***48***∗∗∗*

(0*.*08)

0*.*05

(0*.*03)

*−*0*.*02

(0*.*02)

**0***.***14***∗∗∗*

(0*.*04)

0*.*01

(0*.*03)

*−*0*.*39

(0*.*20)

*−*0*.*16

(0*.*21)

Gender_simpleMale

Gender_simpleOther

Political.Ideology.AllConservative

Political.Ideology.AllLiberal

Age

Education

News.Checking

Social.Media.Use

COVID.Vaccine.Intentions:ConditionEvil COVID.Vaccine.Intentions:ConditionGood

R2

Adj. R2 Num. obs.

0*.*14

0*.*13

689

0*.*20

0*.*17

555

0*.*21

0*.*19

1075

*∗∗∗p <* 0*.*001; *∗∗p <* 0*.*01; *∗p <* 0*.*05

**31 of** [**75**](#_bookmark21)

**Table S3. Linear Regression Model for Manipulativeness Ratings (Difference Between Fake and Real) Including Interaction Between Condition and COVID-Vaccine Intentions**

Study 1

**2***.***63***∗∗∗*

Study 2

**1***.***19***∗*

Study 3

(Intercept)

*−*0*.*06

(0*.*33)

**1***.***26***∗∗∗* (0*.*21) **0***.***65***∗* (0*.*25) **0***.***69***∗* (0*.*27)

*−*0*.*14

(0*.*10)

0*.*02

(0*.*09)

0*.*13

(0*.*29)

0*.*09

(0*.*14)

**0***.***63***∗∗∗*

(0*.*11)

0*.*05

(0*.*04)

*−*0*.*02

(0*.*03)

**0***.***19***∗∗∗*

(0*.*05)

0*.*02

(0*.*05)

*−*0*.*38

(0*.*28)

*−*0*.*27

(0*.*29)

(0*.*43) **0***.***54***∗* (0*.*23)

*−*0*.*09

(0*.*27)

**0***.***75***∗∗*

(0*.*29)

*−***0***.***77***∗∗∗*

(0*.*14)

*−***0***.***53***∗∗∗*

(0*.*12)

0*.*56

(0*.*41)

*−*0*.*03

(0*.*18)

**0***.***61***∗∗∗*

(0*.*15)

0*.*07

(0*.*06)

*−***0***.***21***∗∗* (0*.*07) **0***.***18***∗* (0*.*08)

*−***0***.***23***∗∗∗*

(0*.*06)

0*.*38

(0*.*31)

*−*0*.*31

(0*.*33)

(0*.*51)

**0***.***96***∗∗∗*

(0*.*28)

0*.*16

(0*.*34)

0*.*41

(0*.*34)

*−***0***.***52***∗∗∗*

(0*.*14)

*−***0***.***38***∗∗*

(0*.*13)

0*.*53

(0*.*39)

*−*0*.*01

(0*.*21) **0***.***40***∗* (0*.*18)

0*.*12

(0*.*06)

*−*0*.*03

(0*.*05) **0***.***18***∗* (0*.*08)

*−*0*.*05

(0*.*08)

0*.*07

(0*.*38)

*−*0*.*08

(0*.*38)

COVID.Vaccine.Intentions

ConditionEvil

ConditionGood

Having.Children

Gender_simpleMale

Gender_simpleOther

Political.Ideology.AllConservative

Political.Ideology.AllLiberal Age

Education News.Checking Social.Media.Use

COVID.Vaccine.Intentions:ConditionEvil

COVID.Vaccine.Intentions:ConditionGood

R2

Adj. R2 Num. obs.

0*.*23

0*.*22

689

0*.*19

0*.*17

555

0*.*18

0*.*17

1075

*∗∗∗p <* 0*.*001; *∗∗p <* 0*.*01; *∗p <* 0*.*05

**32 of** [**75**](#_bookmark21)

**Table S4. Linear Regression Model for Confidence Ratings (Fake Items Only) Including Interaction Between Condition and COVID-Vaccine Intentions**

Study 1

**4***.***93***∗∗∗*

Study 2

**5***.***01***∗∗∗*

Study 3

**4***.***73***∗∗∗*

(Intercept)

(0*.*30)

*−*0*.*02

(0*.*16)

*−*0*.*32

(0*.*19)

*−*0*.*03

(0*.*20)

*−***0***.***21***∗*

(0*.*10)

*−*0*.*14

(0*.*08)

0*.*07

(0*.*29)

*−*0*.*02

(0*.*13) **0***.***26***∗* (0*.*10)

**0***.***12***∗∗*

(0*.*04)

0*.*02

(0*.*05)

0*.*06

(0*.*05)

0*.*00

(0*.*04)

**0***.***57***∗∗*

(0*.*22)

0*.*30

(0*.*23)

(0*.*33) **0***.***42***∗* (0*.*19)

0*.*30

(0*.*22)

0*.*23

(0*.*22)

*−*0*.*10

(0*.*09)

*−*0*.*09

(0*.*09)

0*.*20

(0*.*26)

*−*0*.*16

(0*.*14)

0*.*12

(0*.*12)

0*.*04

(0*.*04)

*−*0*.*02

(0*.*03) **0***.***13***∗* (0*.*05)

*−*0*.*02

(0*.*05)

*−*0*.*06

(0*.*25)

*−*0*.*08

(0*.*25)

(0*.*23) **0***.***36***∗* (0*.*15)

0*.*26

(0*.*18)

*−*0*.*08

(0*.*19)

*−*0*.*03

(0*.*07)

*−*0*.*04

(0*.*06)

0*.*24

(0*.*21)

0*.*08

(0*.*10)

**0***.***31***∗∗∗*

(0*.*07)

0*.*05

(0*.*03)

*−*0*.*04

(0*.*02)

**0***.***11***∗∗*

(0*.*03)

0*.*03

(0*.*03)

*−*0*.*06

(0*.*20)

0*.*32

(0*.*21)

COVID.Vaccine.Intentions

ConditionEvil

ConditionGood

Having.Children

Gender_simpleMale

Gender_simpleOther

Political.Ideology.AllConservative

Political.Ideology.AllLiberal Age

Education News.Checking Social.Media.Use

COVID.Vaccine.Intentions:ConditionEvil

COVID.Vaccine.Intentions:ConditionGood

R2

Adj. R2 Num. obs.

0*.*09

0*.*07

689

0*.*09

0*.*06

555

0*.*09

0*.*08

1075

*∗∗∗p <* 0*.*001; *∗∗p <* 0*.*01; *∗p <* 0*.*05

**33 of** [**75**](#_bookmark21)

**Table S5. Linear Regression Model for Sharing Ratings (Fake Items Only) Including Interaction Between Condition and COVID-Vaccine Intentions**

Study 1

Study 2

**1***.***77***∗∗∗*

Study 3

**2***.***55***∗∗∗*

(Intercept)

0*.*35

(0*.*41)

*−*0*.*19

(0*.*22)

0*.*26

(0*.*25)

*−*0*.*18

(0*.*27)

**0***.***74***∗∗∗*

(0*.*13)

**0***.***69***∗∗∗*

(0*.*11)

0*.*03

(0*.*39)

0*.*28

(0*.*18)

*−*0*.*23

(0*.*14)

*−*0*.*03

(0*.*05)

**0***.***24***∗∗∗*

(0*.*07)

*−*0*.*10

(0*.*07)

**0***.***29***∗∗∗*

(0*.*06)

*−*0*.*39

(0*.*30)

*−*0*.*19

(0*.*31)

(0*.*40)

*−***0***.***75***∗∗∗*

(0*.*22)

*−*0*.*22

(0*.*27)

*−*0*.*22

(0*.*27)

**0***.***41***∗∗∗*

(0*.*11)

**0***.***40***∗∗∗*

(0*.*10)

*−*0*.*14

(0*.*31)

0*.*14

(0*.*17)

0*.*01

(0*.*14)

*−*0*.*08

(0*.*05)

*−*0*.*01

(0*.*04)

0*.*07

(0*.*06)

0*.*09

(0*.*06)

0*.*07

(0*.*30)

0*.*08

(0*.*30)

(0*.*30)

*−***0***.***47***∗*

(0*.*19)

0*.*28

(0*.*23)

*−*0*.*12

(0*.*25)

**0***.***26***∗∗*

(0*.*09)

0*.*09

(0*.*08)

*−*0*.*32

(0*.*27)

*−*0*.*05

(0*.*13)

*−***0***.***51***∗∗∗*

(0*.*10)

*−*0*.*00

(0*.*04)

*−*0*.*02

(0*.*03)

*−*0*.*03

(0*.*04)

0*.*05

(0*.*04)

*−*0*.*25

(0*.*25)

0*.*07

(0*.*27)

COVID.Vaccine.Intentions

ConditionEvil

ConditionGood

Having.Children

Gender_simpleMale

Gender_simpleOther

Political.Ideology.AllConservative

Political.Ideology.AllLiberal Age

Education News.Checking Social.Media.Use

COVID.Vaccine.Intentions:ConditionEvil

COVID.Vaccine.Intentions:ConditionGood

R2

Adj. R2 Num. obs.

0*.*21

0*.*19

689

0*.*14

0*.*11

555

0*.*10

0*.*09

1075

*∗∗∗p <* 0*.*001; *∗∗p <* 0*.*01; *∗p <* 0*.*05

**34 of** [**75**](#_bookmark21)

**Table S6. Linear Regression Model for Sharing Ratings (Difference Between Real and Fake) Including Interaction Between Condition and COVID-Vaccine Intentions**

Study 1

Study 2

Study 3

*−***1***.***11***∗∗∗*

(Intercept)

0*.*53

(0*.*39)

**0***.***70***∗∗*

(0*.*22)

*−*0*.*10

(0*.*25) **0***.***56***∗* (0*.*26)

*−***0***.***50***∗∗∗*

(0*.*13)

*−***0***.***25***∗*

(0*.*11)

*−*0*.*34

(0*.*38)

*−*0*.*04

(0*.*17)

**0***.***39***∗∗*

(0*.*14)

**0***.***14***∗∗*

(0*.*05)

*−*0*.*12

(0*.*06)

0*.*02

(0*.*07)

0*.*05

(0*.*06)

0*.*33

(0*.*29)

0*.*02

(0*.*30)

*−*0*.*70

(0*.*48)

**0***.***82***∗∗*

(0*.*27)

*−*0*.*18

(0*.*32)

0*.*10

(0*.*32)

*−*0*.*08

(0*.*13)

*−*0*.*04

(0*.*12)

**1***.***00***∗∗*

(0*.*37)

*−*0*.*18

(0*.*20)

0*.*04

(0*.*17)

0*.*09

(0*.*06)

0*.*01

(0*.*05)

0*.*13

(0*.*07) **0***.***17***∗* (0*.*07)

0*.*33

(0*.*36)

0*.*11

(0*.*36)

(0*.*32)

**0***.***88***∗∗∗* (0*.*20) **0***.***53***∗* (0*.*24) **0***.***62***∗* (0*.*26)

*−*0*.*16

(0*.*10)

0*.*03

(0*.*08)

**0***.***85***∗∗*

(0*.*28)

*−*0*.*10

(0*.*13)

**0***.***51***∗∗∗*

(0*.*10)

0*.*04

(0*.*04)

0*.*03

(0*.*03)

**0***.***25***∗∗∗*

(0*.*05)

0*.*00

(0*.*04)

*−*0*.*37

(0*.*27)

*−*0*.*07

(0*.*28)

COVID.Vaccine.Intentions

ConditionEvil

ConditionGood

Having.Children

Gender_simpleMale

Gender_simpleOther

Political.Ideology.AllConservative

Political.Ideology.AllLiberal Age

Education News.Checking Social.Media.Use

COVID.Vaccine.Intentions:ConditionEvil

COVID.Vaccine.Intentions:ConditionGood

R2

Adj. R2 Num. obs.

0*.*17

0*.*16

689

0*.*14

0*.*12

555

0*.*18

0*.*17

1075

*∗∗∗p <* 0*.*001; *∗∗p <* 0*.*01; *∗p <* 0*.*05

**35 of** [**75**](#_bookmark21)

**Political Ideology.**

**Table S7. Linear Regression Model for Manipulativeness Ratings (Fake Items Only) Including Interaction Between Condition and Political Ideology**

Study 1

Study 2

Study 3

(Intercept)

**5***.***01***∗∗∗*

(0*.*31)

0*.*16

(0*.*23)

0*.*34

(0*.*18)

0*.*39

(0*.*20)

**0***.***55***∗∗*

(0*.*21)

*−***0***.***33***∗∗*

(0*.*10)

*−*0*.*11

(0*.*08)

0*.*23

(0*.*30)

**0***.***60***∗∗∗*

(0*.*11)

0*.*05

(0*.*04)

*−*0*.*00

(0*.*05)

0*.*06

(0*.*05)

*−***0***.***13***∗∗*

(0*.*05)

*−*0*.*28

(0*.*32)

*−*0*.*22

(0*.*24)

*−*0*.*28

(0*.*32)

*−*0*.*14

(0*.*25)

**4***.***90***∗∗∗*

(0*.*36)

*−*0*.*16

(0*.*25)

0*.*12

(0*.*21)

0*.*33

(0*.*27)

0*.*06

(0*.*27)

*−***0***.***31***∗∗*

(0*.*10)

*−***0***.***27***∗∗*

(0*.*09)

0*.*23

(0*.*28)

**0***.***90***∗∗∗*

(0*.*13)

0*.*08

(0*.*04)

*−*0*.*03

(0*.*04)

0*.*03

(0*.*06)

*−*0*.*05

(0*.*06)

0*.*05

(0*.*37)

0*.*01

(0*.*30)

0*.*57

(0*.*37)

0*.*25

(0*.*30)

**3***.***72***∗∗∗*

(0*.*22)

0*.*11

(0*.*16)

**0***.***56***∗∗∗*

(0*.*12)

**0***.***56***∗∗∗* (0*.*15) **0***.***35***∗* (0*.*15)

*−*0*.*06

(0*.*07)

0*.*07

(0*.*06)

*−*0*.*01

(0*.*21)

**0***.***84***∗∗∗*

(0*.*09)

0*.*05

(0*.*03)

*−*0*.*02

(0*.*02)

**0***.***13***∗∗∗*

(0*.*04)

0*.*01

(0*.*03)

*−*0*.*11

(0*.*24)

*−*0*.*18

(0*.*18)

0*.*05

(0*.*24)

*−*0*.*05

(0*.*18)

Political.Ideology.AllConservative

Political.Ideology.AllLiberal

ConditionEvil

ConditionGood Having.Children Gender_simpleMale Gender_simpleOther COVID.Vaccine.Intentions

Age

Education

News.Checking

Social.Media.Use

Political.Ideology.AllConservative:ConditionEvil

Political.Ideology.AllLiberal:ConditionEvil

Political.Ideology.AllConservative:ConditionGood

Political.Ideology.AllLiberal:ConditionGood

R2

Adj. R2 Num. obs.

0*.*14

0*.*12

689

0*.*20

0*.*18

555

0*.*20

0*.*19

1075

*∗∗∗p <* 0*.*001; *∗∗p <* 0*.*01; *∗p <* 0*.*05

**36 of** [**75**](#_bookmark21)

**Table S8. Linear Regression Model for Manipulativeness Ratings (Difference Between Fake and Real) Including Interaction Between Condition and Political Ideology**

Study 1

**2***.***51***∗∗∗*

Study 2

**1***.***19***∗*

Study 3

(Intercept)

Political.Ideology.AllConservative

0*.*14

(0*.*31)

0*.*03

(0*.*22)

**0***.***68***∗∗∗* (0*.*17) **0***.***45***∗* (0*.*21)

0*.*41

(0*.*21)

*−*0*.*14

(0*.*10)

0*.*02

(0*.*09)

0*.*13

(0*.*30)

**1***.***04***∗∗∗*

(0*.*13)

0*.*05

(0*.*04)

*−*0*.*02

(0*.*03)

**0***.***18***∗∗∗*

(0*.*05)

0*.*01

(0*.*05)

*−*0*.*04

(0*.*33)

*−*0*.*19

(0*.*25)

0*.*20

(0*.*33)

0*.*02

(0*.*25)

(0*.*44)

0*.*19

(0*.*32)

**0***.***73***∗∗*

(0*.*25)

0*.*37

(0*.*28) **0***.***68***∗* (0*.*29)

*−***0***.***76***∗∗∗*

(0*.*14)

*−***0***.***55***∗∗∗*

(0*.*12)

0*.*60

(0*.*41)

**0***.***60***∗∗∗*

(0*.*15)

0*.*08

(0*.*06)

*−***0***.***21***∗∗* (0*.*07) **0***.***17***∗* (0*.*08)

*−***0***.***23***∗∗∗*

(0*.*06)

*−*0*.*35

(0*.*45)

*−*0*.*21

(0*.*33)

*−*0*.*35

(0*.*45)

*−*0*.*18

(0*.*34)

(0*.*51)

*−*0*.*18

(0*.*34)

0*.*44

(0*.*29)

0*.*16

(0*.*38)

0*.*37

(0*.*37)

*−***0***.***52***∗∗∗*

(0*.*14)

*−***0***.***39***∗∗*

(0*.*13)

0*.*53

(0*.*39)

**0***.***98***∗∗∗*

(0*.*18)

0*.*12

(0*.*06)

*−*0*.*03

(0*.*05) **0***.***17***∗* (0*.*08)

*−*0*.*05

(0*.*08)

0*.*45

(0*.*51)

*−*0*.*05

(0*.*42)

0*.*11

(0*.*52)

*−*0*.*07

(0*.*42)

Political.Ideology.AllLiberal

ConditionEvil

ConditionGood

Having.Children

Gender_simpleMale

Gender_simpleOther

COVID.Vaccine.Intentions

Age Education

News.Checking Social.Media.Use

Political.Ideology.AllConservative:ConditionEvil

Political.Ideology.AllLiberal:ConditionEvil

Political.Ideology.AllConservative:ConditionGood

Political.Ideology.AllLiberal:ConditionGood

R2

Adj. R2 Num. obs.

0*.*23

0*.*21

689

0*.*19

0*.*17

555

0*.*18

0*.*16

1075

*∗∗∗p <* 0*.*001; *∗∗p <* 0*.*01; *∗p <* 0*.*05

**37 of** [**75**](#_bookmark21)

**Table S9. Linear Regression Model for Confidence Ratings (Fake Items Only) Including Interaction Between Condition and Political Ideology**

Study 1

**4***.***58***∗∗∗*

Study 2

**4***.***79***∗∗∗*

Study 3

**4***.***69***∗∗∗*

(Intercept)

Political.Ideology.AllConservative

(0*.*31)

0*.*36

(0*.*22) **0***.***38***∗* (0*.*17)

0*.*25

(0*.*19) **0***.***46***∗* (0*.*20)

*−***0***.***20***∗*

(0*.*10)

*−*0*.*15

(0*.*08)

0*.*10

(0*.*29)

**0***.***29***∗∗*

(0*.*10)

**0***.***11***∗∗*

(0*.*04)

0*.*02

(0*.*05)

0*.*06

(0*.*05)

0*.*00

(0*.*04)

*−*0*.*45

(0*.*31)

*−*0*.*14

(0*.*23)

*−***0***.***70***∗*

(0*.*31)

*−*0*.*25

(0*.*24)

(0*.*33)

0*.*29

(0*.*22)

0*.*36

(0*.*19)

**0***.***70***∗∗* (0*.*24) **0***.***49***∗* (0*.*24)

*−*0*.*12

(0*.*09)

*−*0*.*07

(0*.*09)

0*.*21

(0*.*26)

**0***.***35***∗∗*

(0*.*12)

0*.*04

(0*.*04)

*−*0*.*03

(0*.*03)

**0***.***16***∗∗*

(0*.*05)

*−*0*.*03

(0*.*05)

*−***0***.***94***∗∗*

(0*.*33)

*−*0*.*41

(0*.*27)

*−*0*.*48

(0*.*34)

*−*0*.*34

(0*.*27)

(0*.*22)

0*.*01

(0*.*16) **0***.***30***∗* (0*.*12)

0*.*04

(0*.*15) **0***.***30***∗* (0*.*15)

*−*0*.*04

(0*.*07)

*−*0*.*06

(0*.*06)

0*.*24

(0*.*21)

**0***.***42***∗∗∗*

(0*.*09)

0*.*05

(0*.*03)

*−*0*.*04

(0*.*02)

**0***.***11***∗∗*

(0*.*03)

0*.*03

(0*.*03)

0*.*21

(0*.*23)

0*.*22

(0*.*18)

0*.*04

(0*.*23)

*−*0*.*18

(0*.*17)

Political.Ideology.AllLiberal

ConditionEvil

ConditionGood

Having.Children

Gender_simpleMale

Gender_simpleOther

COVID.Vaccine.Intentions

Age Education

News.Checking Social.Media.Use

Political.Ideology.AllConservative:ConditionEvil

Political.Ideology.AllLiberal:ConditionEvil

Political.Ideology.AllConservative:ConditionGood

Political.Ideology.AllLiberal:ConditionGood

R2

Adj. R2 Num. obs.

0*.*09

0*.*07

689

0*.*10

0*.*08

555

0*.*09

0*.*08

1075

*∗∗∗p <* 0*.*001; *∗∗p <* 0*.*01; *∗p <* 0*.*05

**38 of** [**75**](#_bookmark21)

**Table S10. Linear Regression Model for Sharing Ratings (Fake Items Only) Including Interaction Between Condition and Political Ideology**

Study 1

Study 2

**1***.***82***∗∗∗*

Study 3

**2***.***53***∗∗∗*

(Intercept)

Political.Ideology.AllConservative

0*.*35

(0*.*42) **0***.***63***∗* (0*.*30)

*−*0*.*16

(0*.*23)

0*.*01

(0*.*26)

*−*0*.*04

(0*.*28)

**0***.***75***∗∗∗*

(0*.*13)

**0***.***70***∗∗∗*

(0*.*11)

*−*0*.*00

(0*.*39)

*−***0***.***39***∗∗*

(0*.*14)

*−*0*.*03

(0*.*05)

**0***.***25***∗∗∗*

(0*.*07)

*−*0*.*10

(0*.*07)

**0***.***30***∗∗∗*

(0*.*06)

*−*0*.*46

(0*.*43)

0*.*06

(0*.*32)

*−*0*.*64

(0*.*43)

*−*0*.*28

(0*.*33)

(0*.*40)

0*.*12

(0*.*27)

*−*0*.*15

(0*.*23)

*−*0*.*29

(0*.*30)

*−*0*.*36

(0*.*29)

**0***.***41***∗∗∗*

(0*.*11)

**0***.***40***∗∗∗*

(0*.*10)

*−*0*.*14

(0*.*31)

*−***0***.***71***∗∗∗*

(0*.*14)

*−*0*.*08

(0*.*05)

*−*0*.*01

(0*.*04)

0*.*08

(0*.*06)

0*.*09

(0*.*06)

*−*0*.*08

(0*.*40)

0*.*22

(0*.*33)

0*.*14

(0*.*41)

0*.*30

(0*.*33)

(0*.*28)

0*.*02

(0*.*20)

*−***0***.***37***∗*

(0*.*16)

0*.*25

(0*.*19)

0*.*06

(0*.*19)

**0***.***25***∗∗*

(0*.*09)

0*.*08

(0*.*08)

*−*0*.*32

(0*.*27)

*−***0***.***54***∗∗∗*

(0*.*12)

*−*0*.*00

(0*.*04)

*−*0*.*02

(0*.*03)

*−*0*.*03

(0*.*04)

0*.*05

(0*.*04)

*−*0*.*17

(0*.*30)

*−*0*.*26

(0*.*23)

*−*0*.*07

(0*.*30)

*−*0*.*19

(0*.*22)

Political.Ideology.AllLiberal

ConditionEvil

ConditionGood

Having.Children

Gender_simpleMale

Gender_simpleOther

COVID.Vaccine.Intentions

Age Education

News.Checking Social.Media.Use

Political.Ideology.AllConservative:ConditionEvil

Political.Ideology.AllLiberal:ConditionEvil

Political.Ideology.AllConservative:ConditionGood

Political.Ideology.AllLiberal:ConditionGood

R2

Adj. R2 Num. obs.

0*.*21

0*.*19

689

0*.*14

0*.*11

555

0*.*10

0*.*09

1075

*∗∗∗p <* 0*.*001; *∗∗p <* 0*.*01; *∗p <* 0*.*05

**39 of** [**75**](#_bookmark21)

**Table S11. Linear Regression Model for Sharing Ratings (Difference Between Real and Fake) Including Interaction Between Condition and Political Ideology**

Study 1

Study 2

Study 3

*−***0***.***97***∗∗*

(Intercept)

Political.Ideology.AllConservative

0*.*49

(0*.*40)

*−*0*.*10

(0*.*29)

0*.*28

(0*.*23)

*−*0*.*03

(0*.*25) **0***.***54***∗* (0*.*27)

*−***0***.***50***∗∗∗*

(0*.*13)

*−***0***.***26***∗*

(0*.*11)

*−*0*.*34

(0*.*38)

**0***.***85***∗∗∗* (0*.*13) **0***.***13***∗* (0*.*05)

*−*0*.*12

(0*.*06)

0*.*01

(0*.*07)

0*.*05

(0*.*06)

0*.*39

(0*.*41)

0*.*18

(0*.*30)

*−*0*.*23

(0*.*41)

0*.*12

(0*.*32)

*−*0*.*89

(0*.*48)

*−*0*.*33

(0*.*33)

0*.*17

(0*.*28)

0*.*05

(0*.*35)

0*.*36

(0*.*35)

*−*0*.*07

(0*.*13)

*−*0*.*03

(0*.*12)

**1***.***01***∗∗*

(0*.*37)

**0***.***99***∗∗∗*

(0*.*17)

0*.*09

(0*.*06)

0*.*01

(0*.*05)

0*.*12

(0*.*07) **0***.***18***∗* (0*.*07)

0*.*36

(0*.*48)

*−*0*.*06

(0*.*40)

0*.*17

(0*.*49)

*−*0*.*31

(0*.*39)

(0*.*30)

*−*0*.*12

(0*.*21)

**0***.***56***∗∗∗*

(0*.*17)

0*.*19

(0*.*20)

**0***.***67***∗∗∗*

(0*.*20)

*−*0*.*17

(0*.*10)

0*.*02

(0*.*09)

**0***.***87***∗∗*

(0*.*28)

**0***.***71***∗∗∗*

(0*.*12)

0*.*04

(0*.*04)

0*.*03

(0*.*03)

**0***.***24***∗∗∗*

(0*.*05)

*−*0*.*00

(0*.*04)

*−*0*.*04

(0*.*31)

0*.*05

(0*.*24)

0*.*07

(0*.*32)

*−*0*.*20

(0*.*24)

Political.Ideology.AllLiberal

ConditionEvil

ConditionGood

Having.Children

Gender_simpleMale

Gender_simpleOther

COVID.Vaccine.Intentions

Age Education

News.Checking Social.Media.Use

Political.Ideology.AllConservative:ConditionEvil

Political.Ideology.AllLiberal:ConditionEvil

Political.Ideology.AllConservative:ConditionGood

Political.Ideology.AllLiberal:ConditionGood

R2

Adj. R2 Num. obs.

0*.*18

0*.*16

689

0*.*14

0*.*12

555

0*.*18

0*.*16

1075

*∗∗∗p <* 0*.*001; *∗∗p <* 0*.*01; *∗p <* 0*.*05

**40 of** [**75**](#_bookmark21)

**Inoculation Theory**

Inoculation theory is built on an immunization analogy ([3](#_bookmark24)), but focuses on psychological rather than physiological immunization. It posits that exposure to a weakened persuasive argument, much like exposure to a weakened version of a pathogen, protects against future exposure to persuasive arguments ([4](#_bookmark25)). Interventions based on inoculation theory have been shown to protect against persuasive arguments in a range of contexts, including health (see [5](#_bookmark26)) and politics (see [6](#_bookmark27)). See ([7](#_bookmark28)) for a meta-analysis and ([5](#_bookmark26)) and ([8](#_bookmark29)) for reviews.

Inoculation strategies can be differentiated along three theoretical lines: therapeutic vs. prophylactic (i.e., the timing of the intervention), active vs. passive (i.e., the level of engagement by the individual), and issue-based vs. technique-based inoculation (i.e., the design of the intervention). *Therapeutic* inoculation aims to confer resistance to persuasive arguments to individuals who have already been exposed to such arguments, while *prophylactic* inoculation aims to confer resistance to individuals who have not yet been exposed ([9](#_bookmark30)). Importantly, inoculation can work in both therapeutic and prophylactic contexts ([9](#_bookmark30)), making this approach amenable for use with the general population, which includes individuals with different pre-exposure levels. However, inoculation might be less effective for individuals who already believe in vaccine misinformation like anti-vaccine conspiracy theories ([10](#_bookmark31)).

*Active* inoculation implies that individuals actively take part in the inoculation process, for example by playing a browser game where they can make choices. In contrast, *passive* inoculation only provides individuals with information aimed at inoculating them, for example in the form of an informational text. Most traditional inoculation interventions focused on passive inoculation methods ([7](#_bookmark28), [11](#_bookmark32)), but more recent studies have utilized active inoculation approaches, such as online games ([12](#_bookmark33)), which may be more effective than passive inoculation ([13](#_bookmark34)).

*Issue-based* inoculation focuses on a specific domain where inoculation is to be achieved, whereas *technique-based* inoculation focuses on the strategies that underlie the spread of persuasive arguments and is expected to be domain-general ([13](#_bookmark34)). Issue-based approaches, such as those with a narrow focus on specific arguments against vaccines ([10](#_bookmark31)) or climate change ([14](#_bookmark35)), work by providing counterarguments to persuasive arguments in one specific area. Technique-based approaches are informed by common misinformation techniques. For example, the *Bad News* game ([https://getbadnews.com](https://getbadnews.com/)) aiming to inoculate players against generic misinformation includes the techniques of polarizing audiences and appealing to emotion ([12](#_bookmark33), [15](#_bookmark36)).

**Identifying Vaccine Misinformation Techniques**

Vaccine misinformation follows predictable tropes or misinformation techniques ([16](#_bookmark37)–[18](#_bookmark39)). We identified four such techniques that are commonly encountered online to develop our intervention: emotional storytelling, fake expertise and pseudoscience, the naturalistic fallacy, and conspiracy theories ([17](#_bookmark38), [18](#_bookmark39)).

Emotional storytelling is the use of emotional narratives to provoke strong emotional responses. This technique is commonly found in anti-vaccine information channels ([17](#_bookmark38)–[19](#_bookmark40)). For example, an analysis of anti-vaccination websites showed that 88% used emotional appeals to promote anti-vaccine misinformation ([17](#_bookmark38)).

The second technique combines fake expertise, where an individual pretends to be an expert on vaccines, and pseudoscience, where false information is given an air of credibility by drawing on scientific-seeming language ([18](#_bookmark39), [20](#_bookmark41)). ([18](#_bookmark39)) summarizes this technique as “skewing the science”, where pro-vaccine content is rejected, but false scientific claims that align with anti-vaccine sentiment are promoted.

The naturalistic fallacy asserts that everything that is natural is good, and everything that is unnatural is bad ([17](#_bookmark38), [18](#_bookmark39)). For example, vaccines might be depicted as something unnatural and unsafe because they allegedly contain toxins ([17](#_bookmark38)). Proponents of this fallacy argue in favor of “naturally” acquiring immunity from diseases by contracting them, and oppose vaccination ([18](#_bookmark39)). Finally, conspiracy theories that assert that vaccines are part of a conspiracy by actors such as the government or pharmaceutical companies are frequently used to spread vaccine misinformation ([17](#_bookmark38), [18](#_bookmark39), [21](#_bookmark42), [22](#_bookmark43)). For example, one conspiracy theory claims that vaccination supporters are only supportive of vaccines because they are paid by “Big Pharma” ([18](#_bookmark39)). Further, a survey analysis suggests that anti-vaccination attitudes are especially common among individuals high in conspiratorial

thinking ([23](#_bookmark44)).

**Descriptive Statistics**

**Number of Observations.**

**Demographics.** Tables [S13](#_bookmark14)-[S15](#_bookmark15) show basic demographic information about study participants in Studies 1 to 3. Age was coded in five buckets (18-24 = 1, 25-34 = 2, 35-44 = 3, 45-54 = 4 , >55 = 5), parental status in two buckets (not having children = 0, having children = 1), and political ideology in three buckets (Liberal = -1, Moderate = 0, Conservative = 1).

Education was coded in six buckets in Study 1 (No formal education above age 16 = 1, Professional or technical qualifications above age 16 = 2, School education up to age 18 = 3, Degree (Bachelor’s) or equivalent = 4, Degree (Master’s) or other postgraduate qualification = 5, Doctorate = 6) and in seven buckets in Studies 2 and 3 (Less than high school = 1, High school graduate = 2, Some college = 3, 2 year degree = 4, 4 year degree = 5, Professional degree = 6, Doctorate = 7).

We use a binarized gender variable to calculate mean and standard deviation (Female = 0, Male = 1). For the proportions of gender measure with three categories (Female, Male, Other), see the Balance Tables section.

**41 of** [**75**](#_bookmark21)

**Table S12. Number of Observations for Studies 1, 2 and 3**

Study 1

Study 2

Study 3

Preregistered target sample size Observations in raw data

Observations after removing preview responses Observations after removing non-consenting participants

Observations after removing participants who did not finish the survey Observations after removing participants with empty Prolific IDs Observations after removing participants who failed attentions check Observations after removing participants who got password wrong

Observations after removing participants with missing values on Manipulativeness Discernment

720

863

863

861

763

763

695

690

690

600

671

667

666

599

599

563

559

557

1200

1353

1353

1342

1187

1157

1082

1079

1079

**42 of** [**75**](#_bookmark21)

**Table S13. Demographics for Study 1**

Variable

n

mean

sd

median

min

max

Age Group (18-24 = 1, 25-34 = 2, 35-44 = 3, 45-54 = 4 , >55 = 5)

Gender (Female = 0, Male = 1)

Parental Status (not having children = 0, having children = 1) Political Ideology (Liberal = -1, Moderate = 0, Conservative = 1) Education (No formal education above age 16 = 1, ..., Doctorate = 6)

690

675

690

690

690

2.51

0.51

0.40

-0.43

3.98

1.22

0.50

0.49

0.76

0.88

2.00

1.00

0.00

-1.00

4.00

1

0

0

-1

1

5

1

1

1

6

**43 of** [**75**](#_bookmark21)

**Table S14. Demographics for Study 2**

Variable

n

mean

sd

median

min

max

Age Group (18-24 = 1, 25-34 = 2, 35-44 = 3, 45-54 = 4 , >55 = 5)

Gender (Female = 0, Male = 1)

Parental Status (not having children = 0, having children = 1) Political Ideology (Liberal = -1, Moderate = 0, Conservative = 1) Education (Less than high school = 1, ..., Doctorate = 7)

557

540

557

557

557

2.71

0.43

0.40

-0.44

4.54

1.19

0.50

0.49

0.80

1.32

2.00

0.00

0.00

-1.00

5.00

1

0

0

-1

1

5

1

1

1

7

**44 of** [**75**](#_bookmark21)

**Table S15. Demographics for Study 3**

Variable

n

mean

sd

median

min

max

Age Group (18-24 = 1, 25-34 = 2, 35-44 = 3, 45-54 = 4 , >55 = 5)

Gender (Female = 0, Male = 1)

Parental Status (not having children = 0, having children = 1) Political Ideology (Liberal = -1, Moderate = 0, Conservative = 1) Education (Less than high school = 1, ..., Doctorate = 7)

1079

1051

1079

1079

1079

2.65

0.50

0.38

-0.44

4.21

1.39

0.50

0.49

0.76

1.39

2.00

0.00

0.00

-1.00

5.00

1

0

0

-1

1

5

1

1

1

7

**45 of** [**75**](#_bookmark21)

**Group Means for Outcome Variables.**

**Table S16. Group means for Study 1**

Variable

Condition

N

Mean

Median

SD

SE

Misinformation Manipulativeness

Control Evil Good Control Evil Good Control Evil Good Control Evil Good Control Evil Good Control Evil Good Control Evil Good Control Evil Good

227

237

226

227

237

226

227

237

226

227

237

226

227

237

226

227

237

226

227

237

226

227

237

226

5.324

5.514

5.722

5.324

5.514

5.722

5.324

5.514

5.722

5.324

5.514

5.722

5.324

5.514

5.722

5.324

5.514

5.722

5.324

5.514

5.722

5.324

5.514

5.722

5.500

5.750

6.000

5.500

5.750

6.000

5.500

5.750

6.000

5.500

5.750

6.000

5.500

5.750

6.000

5.500

5.750

6.000

5.500

5.750

6.000

5.500

5.750

6.000

1.213

1.147

1.030

1.213

1.147

1.030

1.213

1.147

1.030

1.213

1.147

1.030

1.213

1.147

1.030

1.213

1.147

1.030

1.213

1.147

1.030

1.213

1.147

1.030

0.080

0.075

0.069

0.080

0.075

0.069

0.080

0.075

0.069

0.080

0.075

0.069

0.080

0.075

0.069

0.080

0.075

0.069

0.080

0.075

0.069

0.080

0.075

0.069

Non-Misinformation Manipulativeness

Manipulativeness Discernment

Misinformation Confidence

Non-Misinformation Confidence

Misinformation Sharing Intent

Non-Misinformation Sharing Intent

Sharing Intent Discernment

**Table S17. Group means for Study 2**

Variable

Condition

N

Mean

Median

SD

SE

Misinformation Manipulativeness

Control Evil Good Control Evil Good Control Evil Good Control Evil Good Control Evil Good Control Evil Good Control Evil Good Control Evil Good

196

182

179

196

182

179

196

182

179

196

182

179

196

182

179

196

182

179

196

182

179

196

182

179

5.438

5.761

5.788

5.438

5.761

5.788

5.438

5.761

5.788

5.438

5.761

5.788

5.438

5.761

5.788

5.438

5.761

5.788

5.438

5.761

5.788

5.438

5.761

5.788

5.667

6.000

5.875

5.667

6.000

5.875

5.667

6.000

5.875

5.667

6.000

5.875

5.667

6.000

5.875

5.667

6.000

5.875

5.667

6.000

5.875

5.667

6.000

5.875

1.248

1.184

0.990

1.248

1.184

0.990

1.248

1.184

0.990

1.248

1.184

0.990

1.248

1.184

0.990

1.248

1.184

0.990

1.248

1.184

0.990

1.248

1.184

0.990

0.089

0.088

0.074

0.089

0.088

0.074

0.089

0.088

0.074

0.089

0.088

0.074

0.089

0.088

0.074

0.089

0.088

0.074

0.089

0.088

0.074

0.089

0.088

0.074

Non-Misinformation Manipulativeness

Manipulativeness Discernment

Misinformation Confidence

Non-Misinformation Confidence

Misinformation Sharing Intent

Non-Misinformation Sharing Intent

Sharing Intent Discernment

**46 of** [**75**](#_bookmark21)

**Table S18. Group means for Study 3**

Variable

Condition

N

Mean

Median

SD

SE

Misinformation Manipulativeness

Control Evil Good Control Evil Good Control Evil Good Control Evil Good Control Evil Good Control Evil Good Control Evil Good Control Evil Good

383

344

352

383

344

352

383

344

352

383

344

352

383

344

352

383

344

352

383

344

352

383

344

352

5.352

5.781

5.713

5.352

5.781

5.713

5.352

5.781

5.713

5.352

5.781

5.713

5.352

5.781

5.713

5.352

5.781

5.713

5.352

5.781

5.713

5.352

5.781

5.713

5.600

6.000

5.857

5.600

6.000

5.857

5.600

6.000

5.857

5.600

6.000

5.857

5.600

6.000

5.857

5.600

6.000

5.857

5.600

6.000

5.857

5.600

6.000

5.857

1.202

1.034

1.020

1.202

1.034

1.020

1.202

1.034

1.020

1.202

1.034

1.020

1.202

1.034

1.020

1.202

1.034

1.020

1.202

1.034

1.020

1.202

1.034

1.020

0.061

0.056

0.054

0.061

0.056

0.054

0.061

0.056

0.054

0.061

0.056

0.054

0.061

0.056

0.054

0.061

0.056

0.054

0.061

0.056

0.054

0.061

0.056

0.054

Non-Misinformation Manipulativeness

Manipulativeness Discernment

Misinformation Confidence

Non-Misinformation Confidence

Misinformation Sharing Intent

Non-Misinformation Sharing Intent

Sharing Intent Discernment

**47 of** [**75**](#_bookmark21)

**Balance Tables**

**Table S19. Balance Table for Treatment Condition for Study 1**

**Condition**

**Evil**

**Variable**

**Control**

**Good**

**p-value**

**SMD**

*Note:* p-values result from a joint F-test for continuous variables and from a Chi-squared test for categorical variables.

**48 of** [**75**](#_bookmark21)

Number of Observations Participant (mean (SD))

Parental Status = Having Children (N (%)) Gender (N (%))

... Female

... Male

... Other

Political Ideology (N (%))

... Liberal

... Moderate

... Conservative

Covid Vaccinination Intentions = Yes (N (%)) Age (N (%))

... 18-24

... 25-34

... 35-44

... 45-54

... >55

Education (N (%))

... No formal education above age 16

... Professional or technical qualifications above age 16

... School education up to age 18

... Degree (Bachelor’s) or equivalent

... Degree (Master’s) or other postgraduate qualification

... Doctorate

News Checking Frequency (N (%))

... Never

... Rarely

... Sometimes

... Frequently

... All the time

Social Media Use Frequency (N (%))

... Never

... Rarely

... Sometimes

... Frequently

... All the time

Marital Status Married (mean (SD)) Percentage Treatment Posts (mean (SD))

227

343.63 (186.59)

87 (38.3)

106 (46.7)

118 (52.0)

3 ( 1.3)

135 (59.5)

54 (23.8)

38 (16.7)

167 (73.6)

44 (19.4)

85 (37.4)

46 (20.3)

27 (11.9)

25 (11.0)

0 ( 0.0)

4 ( 1.8)

70 (30.8)

82 (36.1)

61 (26.9)

10 ( 4.4)

4 ( 1.8)

15 ( 6.6)

61 (26.9)

110 (48.5)

37 (16.3)

7 ( 3.1)

11 ( 4.8)

48 (21.1)

88 (38.8)

73 (32.2)

0.43 (0.50)

0.45 (0.14)

237

339.68 (206.33)

92 (38.8)

116 (49.2)

114 (48.3)

6 ( 2.5)

139 (58.6)

64 (27.0)

34 (14.3)

170 (71.7)

51 (21.5)

90 (38.0)

41 (17.3)

36 (15.2)

19 ( 8.0)

0 ( 0.0)

6 ( 2.5)

70 (29.5)

97 (40.9)

57 (24.1)

7 ( 3.0)

2 ( 0.8)

15 ( 6.3)

62 (26.2)

108 (45.6)

50 (21.1)

2 ( 0.8)

10 ( 4.2)

44 (18.6)

93 (39.2)

88 (37.1)

0.42 (0.49)

0.47 (0.14)

226

353.48 (204.80)

97 (42.9)

112 (49.6)

109 (48.2)

5 ( 2.2)

135 (59.7)

50 (22.1)

41 (18.1)

176 (77.9)

59 (26.1)

69 (30.5)

51 (22.6)

32 (14.2)

15 ( 6.6)

1 ( 0.4)

4 ( 1.8)

65 (28.8)

95 (42.0)

54 (23.9)

7 ( 3.1)

0 ( 0.0)

14 ( 6.2)

54 (23.9)

106 (46.9)

52 (23.0)

4 ( 1.8)

10 ( 4.4)

28 (12.4)

81 (35.8)

103 (45.6)

0.42 (0.50)

0.45 (0.13)

0.747 0.046

0.547 0.062

0.817 0.075

0.691 0.092

0.301 0.095

0.276 0.205

0.880 0.137

0.494 0.181

0.073 0.248

0.955 0.019

0.388 0.077

**Table S20. Balance Table for Treatment Condition for Study 2**

**Condition**

**Evil**

**Variable**

**Control**

**Good**

**p-value**

**SMD**

*Note:* p-values result from a joint F-test for continuous variables and from a Chi-squared test for categorical variables.

**49 of** [**75**](#_bookmark21)

Number of Observations Participant (mean (SD))

Parental Status = Having Children (N (%)) Gender (N (%))

... Female

... Male

... Other

Political Ideology (N (%))

... Liberal

... Moderate

... Conservative

Covid Vaccinination Intentions = Yes (N (%)) Age (N (%))

... 18-24

... 25-34

... 35-44

... 45-54

... >55

Education (N (%))

... Less than high school

... High school graduate

... Some college

... 2 year degree

... 4 year degree

... Professional degree

... Doctorate

News Checking Frequency (N (%))

... Never

... Rarely

... Sometimes

... Frequently

... All the time

Social Media Use Frequency (N (%))

... Never

... Rarely

... Sometimes

... Frequently

... All the time

Urban Rural Status = Urban (N (%)) Income (mean (SD))

Percentage Treatment Posts (mean (SD))

196

286.66 (164.16)

90 (45.9)

107 (54.9)

84 (43.1)

4 ( 2.1)

120 (61.2)

34 (17.3)

42 (21.4)

161 (82.1)

23 (11.7)

80 (40.8)

45 (23.0)

24 (12.2)

24 (12.2)

2 ( 1.0)

17 ( 8.7)

35 (17.9)

15 ( 7.7)

80 (40.8)

44 (22.4)

3 ( 1.5)

2 ( 1.0)

9 ( 4.6)

54 (27.6)

91 (46.4)

40 (20.4)

0 ( 0.0)

5 ( 2.6)

34 (17.3)

88 (44.9)

69 (35.2)

148 (75.5)

7.00 (3.21)

0.47 (0.14)

182

285.49 (163.41)

67 (36.8)

93 (51.4)

83 (45.9)

5 ( 2.8)

116 (63.7)

30 (16.5)

36 (19.8)

140 (76.9)

29 (15.9)

73 (40.1)

42 (23.1)

18 ( 9.9)

20 (11.0)

1 ( 0.5)

14 ( 7.7)

37 (20.3)

15 ( 8.2)

81 (44.5)

28 (15.4)

6 ( 3.3)

4 ( 2.2)

16 ( 8.8)

41 (22.5)

88 (48.4)

33 (18.1)

1 ( 0.5)

10 ( 5.5)

25 (13.7)

84 (46.2)

62 (34.1)

139 (76.4)

6.96 (3.32)

0.43 (0.14)

179

267.58 (157.42)

68 (38.0)

106 (59.2)

67 (37.4)

6 ( 3.4)

119 (66.5)

30 (16.8)

30 (16.8)

140 (78.2)

18 (10.1)

69 (38.5)

44 (24.6)

27 (15.1)

21 (11.7)

0 ( 0.0)

18 (10.1)

25 (14.0)

15 ( 8.4)

78 (43.6)

37 (20.7)

6 ( 3.4)

2 ( 1.1)

14 ( 7.8)

46 (25.7)

82 (45.8)

35 (19.6)

1 ( 0.6)

9 ( 5.0)

32 (17.9)

77 (43.0)

60 (33.5)

134 (74.9)

7.31 (3.21)

0.47 (0.14)

0.450 0.079

0.143 0.124

0.530 0.128

0.823 0.086

0.426 0.086

0.758 0.157

0.703 0.230

0.749 0.160

0.787 0.164

0.945 0.023

0.532 0.072

0.020 0.180

**Table S21. Balance Table for Treatment Condition for Study 3**

**Condition**

**Evil**

**Variable**

**Control**

**Good**

**p-value**

**SMD**

*Note:* p-values result from a joint F-test for continuous variables and from a Chi-squared test for categorical variables.

**50 of** [**75**](#_bookmark21)

Number of Observations Participant (mean (SD))

Parental Status = Having Children (N (%)) Gender (N (%))

... Female

... Male

... Other

Political Ideology (N (%))

... Liberal

... Moderate

... Conservative

Covid Vaccinination Intentions = Yes (N (%)) Age (N (%))

... 18-24

... 25-34

... 35-44

... 45-54

... >55

Education (N (%))

... Less than high school

... High school graduate

... Some college

... 2 year degree

... 4 year degree

... Professional degree

... Doctorate

News Checking Frequency (N (%))

... Never

... Rarely

... Sometimes

... Frequently

... All the time

Social Media Use Frequency (N (%))

... Never

... Rarely

... Sometimes

... Frequently

... All the time Political Party (N (%))

... Democrat

... Independent

... Republican

... Other

Percentage Treatment Posts (mean (SD))

383

531.53 (311.91)

155 (40.5)

183 (47.8)

186 (48.6)

14 ( 3.7)

223 (58.2)

93 (24.3)

67 (17.5)

325 (84.9)

95 (24.8)

105 (27.4)

71 (18.5)

45 (11.7)

67 (17.5)

6 ( 1.6)

44 (11.5)

91 (23.8)

33 ( 8.6)

141 (36.8)

61 (15.9)

7 ( 1.8)

8 ( 2.1)

44 (11.5)

110 (28.7)

170 (44.4)

51 (13.3)

10 ( 2.6)

30 ( 7.8)

67 (17.5)

141 (36.8)

135 (35.2)

178 (47.7)

129 (34.6)

48 (12.9)

18 ( 4.8)

0.45 (0.14)

344

537.62 (313.25)

138 (40.1)

175 (51.3)

163 (47.8)

3 ( 0.9)

210 (61.0)

77 (22.4)

57 (16.6)

283 (82.3)

91 (26.5)

95 (27.6)

65 (18.9)

41 (11.9)

52 (15.1)

0 ( 0.0)

41 (11.9)

84 (24.4)

38 (11.0)

121 (35.2)

54 (15.7)

6 ( 1.7)

6 ( 1.7)

30 ( 8.7)

95 (27.6)

156 (45.3)

57 (16.6)

2 ( 0.6)

19 ( 5.5)

62 (18.0)

140 (40.7)

121 (35.2)

178 (53.1)

103 (30.7)

39 (11.6)

15 ( 4.5)

0.45 (0.14)

352

551.55 (310.25)

121 (34.4)

172 (49.0)

172 (49.0)

7 ( 2.0)

219 (62.2)

81 (23.0)

52 (14.8)

304 (86.4)

83 (23.6)

110 (31.2)

63 (17.9)

42 (11.9)

54 (15.3)

4 ( 1.1)

52 (14.8)

77 (21.9)

28 ( 8.0)

136 (38.6)

46 (13.1)

9 ( 2.6)

8 ( 2.3)

41 (11.6)

119 (33.8)

121 (34.4)

63 (17.9)

8 ( 2.3)

27 ( 7.7)

52 (14.8)

135 (38.4)

130 (36.9)

180 (53.4)

91 (27.0)

44 (13.1)

22 ( 6.5)

0.47 (0.14)

0.675 0.043

0.171 0.084

0.142 0.131

0.797 0.066

0.320 0.075

0.955 0.084

0.458 0.191

0.096 0.193

0.398 0.155

0.358 0.134

0.236 0.077

**Detailed Results**

**Study 1.**

***Bartlett Test of Homogeneity of Variances.***

**Table S22. Bartlett Test of Homogeneity of Variances for Study 1**

Variable

Bartlett’s K-squared

*df*

*p*

Misinformation Manipulativeness

Non-Misinformation Manipulativeness Misinformation Manipulativeness Manipulativeness Discernment Misinformation Confidence

Non-Misinformation Confidence Misinformation Sharing Intent

Non-Misinformation Sharing Intent Sharing Intent Discernment

6.09

2.11

6.09

1.35

0.76

2.07

3.43

0.06

5.61

2

2

2

2

2

2

2

2

2

**0.05**

0.35

**0.05**

0.51

0.69

0.36

0.18

0.97

0.06

***Linear Regression Models.***

**Manipulativess**

**Table S23. Linear Regression Model for Manipulativeness Ratings (Fake Items Only) for Study 1**

Predictor

95% CI

*df*

*b*

*t*

*p*

Intercept ConditionEvil ConditionGood Having Children Gender simpleMale Gender simpleOther

Political Ideology AllLiberal Political Ideology AllConservative COVID Vaccine Intentions

Age Education

News Checking Social Media Use

5.12

0.21

0.41

-0.33

-0.10

0.23

0.22

-0.02

0.60

0.05

0.00

0.06

-0.13

[4.54, 5.70]

[0.02, 0.41]

[0.21, 0.61]

[-0.52, -0.13]

[-0.27, 0.06]

[-0.35, 0.81]

[0.01, 0.42]

[-0.28, 0.24]

[0.40, 0.81]

[-0.03, 0.13]

[-0.10, 0.09]

[-0.05, 0.16]

[-0.22, -0.04]

17.38

2.13

4.06

-3.33

-1.24

0.78

2.06

-0.18

5.75

1.23

-0.08

1.03

-2.89

676

676

676

676

676

676

676

676

676

676

676

676

676

< .001

.034

< .001

< .001

.216

.433

.040

.857

< .001

.218

.933

.304

.004

**Table S24. Linear Regression Model for Manipulativeness Ratings (Real Items Only) for Study 1**

Predictor

95% CI

*df*

*b*

*t*

*p*

Intercept ConditionEvil ConditionGood Having Children Gender simpleMale Gender simpleOther

Political Ideology AllLiberal Political Ideology AllConservative COVID Vaccine Intentions

Age Education

News Checking Social Media Use

2.49

0.03

-0.10

0.44

0.44

-0.36

-0.38

0.02

0.00

-0.03

0.21

-0.11

0.10

[1.87, 3.10]

[-0.18, 0.24]

[-0.31, 0.12]

[0.23, 0.64]

[0.27, 0.62]

[-0.98, 0.26]

[-0.60, -0.16]

[-0.25, 0.30]

[-0.21, 0.22]

[-0.12, 0.06]

[0.10, 0.31]

[-0.23, 0.00]

[0.01, 0.20]

7.93

0.27

-0.90

4.19

4.95

-1.15

-3.42

0.18

0.04

-0.68

3.90

-1.97

2.12

676

676

676

676

676

676

676

676

676

676

676

676

676

< .001

.784

.367

< .001

< .001

.249

< .001

.859

.966

.495

< .001

.049

.034

**51 of** [**75**](#_bookmark21)

**Table S25. Linear Regression Model for Manipulativeness Ratings (Difference between Fake and Real) for Study 1**

Predictor

95% CI

*df*

*b*

*t*

*p*

Intercept ConditionEvil ConditionGood Having Children Gender simpleMale Gender simpleOther

Political Ideology AllLiberal Political Ideology AllConservative COVID Vaccine Intentions

Age Education

News Checking Social Media Use

2.63

0.18

0.51

-0.77

-0.55

0.60

0.60

-0.05

0.60

0.08

-0.21

0.17

-0.23

[1.83, 3.44]

[-0.09, 0.46]

[0.23, 0.79]

[-1.04, -0.50]

[-0.77, -0.32]

[-0.21, 1.41]

[0.31, 0.89]

[-0.41, 0.31]

[0.31, 0.88]

[-0.03, 0.19]

[-0.35, -0.08]

[0.02, 0.32]

[-0.35, -0.11]

6.43

1.32

3.61

-5.61

-4.68

1.45

4.11

-0.27

4.11

1.41

-3.05

2.25

-3.70

676

676

676

676

676

676

676

676

676

676

676

676

676

< .001

.187

< .001

< .001

< .001

.148

< .001

.791

< .001

.159

.002

.025

< .001

**Confidence**

**Table S26. Linear Regression Model for Confidence Ratings (Fake Items Only) for Study 1**

Predictor

95% CI

*df*

*b*

*t*

*p*

Intercept ConditionEvil ConditionGood Having Children Gender simpleMale Gender simpleOther

Political Ideology AllLiberal Political Ideology AllConservative COVID Vaccine Intentions

Age Education

News Checking Social Media Use

4.75

0.09

0.19

-0.21

-0.14

0.11

0.26

-0.02

0.28

0.11

0.02

0.06

0.00

[4.18, 5.31]

[-0.10, 0.28]

[0.00, 0.38]

[-0.40, -0.02]

[-0.30, 0.02]

[-0.46, 0.67]

[0.06, 0.46]

[-0.28, 0.23]

[0.08, 0.48]

[0.04, 0.19]

[-0.08, 0.11]

[-0.04, 0.16]

[-0.09, 0.08]

16.56

0.94

1.92

-2.17

-1.78

0.37

2.53

-0.18

2.79

2.87

0.32

1.12

-0.04

676

676

676

676

676

676

676

676

676

676

676

676

676

< .001

.345

.055

.031

.076

.711

.012

.857

.005

.004

.750

.264

.971

**Table S27. Linear Regression Model for Confidence Ratings (Real Items Only) for Study 1**

Predictor

95% CI

*df*

*b*

*t*

*p*

Intercept ConditionEvil ConditionGood Having Children Gender simpleMale Gender simpleOther

Political Ideology AllLiberal Political Ideology AllConservative COVID Vaccine Intentions

Age Education

News Checking Social Media Use

4.13

0.09

0.08

-0.25

-0.01

0.10

0.17

-0.08

0.11

0.16

0.04

0.07

0.06

[3.57, 4.69]

[-0.10, 0.28]

[-0.11, 0.28]

[-0.44, -0.06]

[-0.17, 0.15]

[-0.46, 0.67]

[-0.03, 0.37]

[-0.34, 0.17]

[-0.09, 0.31]

[0.08, 0.23]

[-0.05, 0.14]

[-0.03, 0.17]

[-0.03, 0.14]

14.49

0.89

0.84

-2.61

-0.11

0.36

1.69

-0.66

1.12

3.92

0.86

1.34

1.30

676

676

676

676

676

676

676

676

676

676

676

676

676

< .001

.373

.401

.009

.912

.717

.091

.512

.265

< .001

.391

.180

.195

**52 of** [**75**](#_bookmark21)

**Sharing Intent**

**Table S28. Linear Regression Model for Sharing Ratings (Fake Items Only) for Study 1**

Predictor

95% CI

*df*

*b*

*t*

*p*

Intercept ConditionEvil ConditionGood Having Children Gender simpleMale Gender simpleOther

Political Ideology AllLiberal Political Ideology AllConservative COVID Vaccine Intentions

Age Education

News Checking Social Media Use

0.47

-0.03

-0.31

0.74

0.70

0.00

-0.22

0.28

-0.40

-0.03

0.24

-0.10

0.30

[-0.30, 1.23]

[-0.29, 0.23]

[-0.58, -0.05]

[0.48, 1.00]

[0.48, 0.92]

[-0.77, 0.78]

[-0.50, 0.05]

[-0.07, 0.62]

[-0.67, -0.13]

[-0.13, 0.08]

[0.11, 0.37]

[-0.24, 0.05]

[0.18, 0.41]

1.19

-0.20

-2.33

5.65

6.28

0.01

-1.60

1.58

-2.86

-0.51

3.69

-1.34

4.98

676

676

676

676

676

676

676

676

676

676

676

676

676

.235

.840

.020

< .001

< .001

.993

.111

.114

.004

.608

< .001

.180

< .001

**Table S29. Linear Regression Model for Sharing Ratings (Real Items Only) for Study 1**

Predictor

95% CI

*df*

*b*

*t*

*p*

Intercept ConditionEvil ConditionGood Having Children Gender simpleMale Gender simpleOther

Political Ideology AllLiberal Political Ideology AllConservative COVID Vaccine Intentions

Age Education

News Checking Social Media Use

0.92

0.11

0.25

0.24

0.44

-0.31

0.16

0.23

0.43

0.11

0.12

-0.08

0.34

[0.13, 1.72]

[-0.16, 0.38]

[-0.02, 0.53]

[-0.03, 0.50]

[0.21, 0.66]

[-1.11, 0.49]

[-0.12, 0.45]

[-0.13, 0.58]

[0.15, 0.71]

[0.00, 0.22]

[-0.01, 0.26]

[-0.23, 0.06]

[0.22, 0.47]

2.29

0.79

1.82

1.77

3.82

-0.76

1.12

1.25

2.99

1.96

1.78

-1.12

5.59

676

676

676

676

676

676

676

676

676

676

676

676

676

.023

.431

.069

.078

< .001

.445

.261

.211

.003

.051

.075

.264

< .001

**Table S30. Linear Regression Model for Sharing Ratings (Difference between Fake and Real) for Study 1**

Predictor

95% CI

*df*

*b*

*t*

*p*

Intercept ConditionEvil ConditionGood Having Children Gender simpleMale Gender simpleOther

Political Ideology AllLiberal Political Ideology AllConservative COVID Vaccine Intentions

Age Education

News Checking Social Media Use

0.46

0.13

0.57

-0.50

-0.26

-0.31

0.39

-0.05

0.83

0.14

-0.12

0.01

0.05

[-0.28, 1.20]

[-0.12, 0.39]

[0.31, 0.83]

[-0.75, -0.25]

[-0.47, -0.05]

[-1.06, 0.43]

[0.12, 0.65]

[-0.38, 0.28]

[0.56, 1.09]

[0.03, 0.24]

[-0.25, 0.00]

[-0.12, 0.15]

[-0.07, 0.16]

1.21

1.05

4.36

-3.97

-2.42

-0.83

2.86

-0.30

6.17

2.63

-1.92

0.19

0.82

676

676

676

676

676

676

676

676

676

676

676

676

676

.225

.294

< .001

< .001

.016

.409

.004

.764

< .001

.009

.056

.846

.412

**53 of** [**75**](#_bookmark21)

***Multilevel Models.***

**Manipulativess**

**Table S31. Multilevel Model for Manipulativeness Ratings (All Items) for Study 1**

*β*ˆ

Term

95% CI

*df*

*t*

*p*

Intercept ConditionEvil ConditionGood Matched controlTRUE Having Children Gender simpleMale Gender simpleOther

Political Ideology AllLiberal Political Ideology AllConservative COVID Vaccine Intentions

Age scaled Education scaled

News Checking scaled Social Media Use scaled

ConditionEvil *×* Matched controlTRUE ConditionGood *×* Matched controlTRUE **Random Effects**

*σ*2

*τ*00 Participant *τ*00 Item NParticipant NItem Marginal R2 Conditional R2

5.06

0.23

0.38

-1.82

0.07

0.17

-0.06

-0.08

-0.02

0.29

0.01

0.09

-0.01

-0.02

-0.14

-0.42

[4.65, 5.48]

[0.05, 0.41]

[0.20, 0.56]

[-2.33, -1.32]

[-0.08, 0.22]

[0.05, 0.30]

[-0.50, 0.39]

[-0.24, 0.07]

[-0.21, 0.18]

[0.13, 0.44]

[-0.06, 0.09]

[0.02, 0.15]

[-0.08, 0.06]

[-0.08, 0.04]

[-0.31, 0.03]

[-0.59, -0.25]

23.84

2.57

4.18

-7.14

0.91

2.69

-0.25

-1.03

-0.15

3.58

0.32

2.59

-0.19

-0.59

-1.65

-4.87

38.50

1230.45

1238.96

23.85

675.83

676.69

675.78

675.96

675.88

676.02

676.36

676.16

675.99

675.92

7894.93

7878.42

< .001

.010

< .001

< .001

.363

.007

.802

.301

.882

< .001

.751

.010

.846

.557

.100

< .001

2.39

0.47

0.37

689

24

0.247

0.442

**Confidence**

**Table S32. Multilevel Model for Confidence Ratings (All Items) for Study 1**

*β*ˆ

Term

95% CI

*df*

*t*

*p*

Intercept ConditionEvil ConditionGood Matched controlTRUE Having Children Gender simpleMale Gender simpleOther

Political Ideology AllLiberal Political Ideology AllConservative COVID Vaccine Intentions

Age scaled Education scaled

News Checking scaled Social Media Use scaled

ConditionEvil *×* Matched controlTRUE ConditionGood *×* Matched controlTRUE **Random Effects**

*σ*2

*τ*00 Participant *τ*00 Item NParticipant NItem Marginal R2 Conditional R2

5.41

0.09

0.20

-0.25

-0.24

-0.08

0.15

0.20

-0.05

0.20

0.17

0.02

0.07

0.02

-0.04

-0.14

1.24

0.73

0.06

689

24

0.050

0.421

[5.14, 5.68]

[-0.09, 0.27]

[0.02, 0.38]

[-0.47, -0.03]

[-0.41, -0.08]

[-0.22, 0.06]

[-0.35, 0.64]

[0.02, 0.37]

[-0.27, 0.17]

[0.03, 0.38]

[0.09, 0.26]

[-0.05, 0.10]

[-0.01, 0.15]

[-0.05, 0.09]

[-0.16, 0.08]

[-0.27, -0.02]

38.74

0.98

2.12

-2.25

-2.88

-1.16

0.58

2.18

-0.41

2.29

4.03

0.62

1.79

0.51

-0.60

-2.26

193.74

898.86

901.64

27.62

675.99

676.35

675.97

676.05

676.01

676.07

676.21

676.13

676.06

676.03

7711.26

7702.73

< .001

.330

.034

.033

.004

.247

.563

.030

.681

.022

< .001

.536

.074

.612

.549

.024

**54 of** [**75**](#_bookmark21)

**Sharing Intent**

**Table S33. Multilevel Model for Sharing Ratings (All Items) for Study 1**

*β*ˆ

Term

95% CI

*df*

*t*

*p*

Intercept ConditionEvil ConditionGood Matched controlTRUE Having Children Gender simpleMale Gender simpleOther

Political Ideology AllLiberal Political Ideology AllConservative COVID Vaccine Intentions

Age scaled Education scaled

News Checking scaled Social Media Use scaled

ConditionEvil *×* Matched controlTRUE ConditionGood *×* Matched controlTRUE **Random Effects**

*σ*2

*τ*00 Participant *τ*00 Item NParticipant NItem Marginal R2 Conditional R2

1.95

-0.05

-0.32

0.95

0.49

0.57

-0.08

-0.03

0.23

0.03

0.03

0.17

-0.08

0.30

0.17

0.55

1.81

1.45

0.23

689

24

0.158

0.564

[1.52, 2.37]

[-0.29, 0.20]

[-0.57, -0.07]

[0.54, 1.35]

[0.26, 0.71]

[0.38, 0.77]

[-0.76, 0.61]

[-0.28, 0.21]

[-0.07, 0.54]

[-0.21, 0.27]

[-0.08, 0.15]

[0.07, 0.27]

[-0.19, 0.03]

[0.20, 0.40]

[0.02, 0.32]

[0.40, 0.70]

8.94

-0.39

-2.47

4.61

4.18

5.78

-0.22

-0.27

1.49

0.22

0.52

3.30

-1.50

5.93

2.27

7.18

100.40

843.86

845.88

24.27

675.96

676.24

675.94

676.00

675.98

676.02

676.13

676.07

676.01

675.99

7674.73

7668.12

< .001

.696

.014

< .001

< .001

< .001

.827

.789

.136

.823

.601

.001

.135

< .001

.023

< .001

***Item-Level Results.***

**Bartlett test items**

**Table S34. Item-Level Bartlett Test of Homogeneity of Variances for Study 1: Manipulativeness**

Variable

Bartlett’s K-squared

*df*

*p*

Conspir.Database Manipulativeness Conspir.Database.C Manipulativeness Conspir.Leaders Manipulativeness Conspir.Leaders.C Manipulativeness Conspir.Greatergood Manipulativeness Conspir.Greatergood.C Manipulativeness Expert.FluVaccine Manipulativeness Expert.FluVaccine.C Manipulativeness Expert.HPV Manipulativeness Expert.HPV.C Manipulativeness Expert.Autism Manipulativeness Expert.Autism.C Manipulativeness Natural.Glyphosate Manipulativeness Natural.Glyphosate.C Manipulativeness Natural.Homeopathy Manipulativeness Natural.Homeopathy.C Manipulativeness Natural.Viruses Manipulativeness Natural.Viruses.C Manipulativeness Story.Multiple Manipulativeness Story.Multiple.C Manipulativeness Story.VaccineCourt Manipulativeness Story.VaccineCourt.C Manipulativeness Story.Hepatitis Manipulativeness Story.Hepatitis.C Manipulativeness

3.480

0.611

7.754

1.532

11.104

0.058

1.402

0.362

3.478

0.383

9.969

2.371

1.491

0.604

4.253

1.165

0.064

0.216

37.815

3.285

2.145

1.641

3.532

1.390

2

2

2

2

2

2

2

2

2

2

2

2

2

2

2

2

2

2

2

2

2

2

2

2

0.176

0.737

**0.021**

0.465

**0.004**

0.972

0.496

0.834

0.176

0.826

**0.007**

0.306

0.474

0.739

0.119

0.558

0.968

0.898

**<0.001**

0.193

0.342

0.440

0.171

0.499

**55 of** [**75**](#_bookmark21)

**Table S35. Item-Level Bartlett Test of Homogeneity of Variances for Study 1: Confidence**

Variable

Bartlett’s K-squared

*df*

*p*

Conspir.Database Confidence Conspir.Database.C Confidence Conspir.Leaders Confidence Conspir.Leaders.C Confidence Conspir.Greatergood Confidence Conspir.Greatergood.C Confidence Expert.FluVaccine Confidence Expert.FluVaccine.C Confidence Expert.HPV Confidence Expert.HPV.C Confidence Expert.Autism Confidence Expert.Autism.C Confidence Natural.Glyphosate Confidence Natural.Glyphosate.C Confidence Natural.Homeopathy Confidence Natural.Homeopathy.C Confidence Natural.Viruses Confidence Natural.Viruses.C Confidence Story.Multiple Confidence Story.Multiple.C Confidence Story.VaccineCourt Confidence Story.VaccineCourt.C Confidence Story.Hepatitis Confidence Story.Hepatitis.C Confidence

0.204

0.983

0.883

5.096

4.024

3.015

0.779

3.267

4.474

0.090

1.193

3.934

1.854

0.272

0.843

2.126

0.649

1.903

0.242

0.008

1.546

0.334

1.656

11.365

2

2

2

2

2

2

2

2

2

2

2

2

2

2

2

2

2

2

2

2

2

2

2

2

0.903

0.612

0.643

0.078

0.134

0.221

0.677

0.195

0.107

0.956

0.551

0.140

0.396

0.873

0.656

0.345

0.723

0.386

0.886

0.996

0.462

0.846

0.437

**0.003**

**Table S36. Item-Level Bartlett Test of Homogeneity of Variances for Study 1: Sharing**

Variable

Bartlett’s K-squared

*df*

*p*

**0.027**

Conspir.Database Sharing Conspir.Database.C Sharing Conspir.Leaders Sharing Conspir.Leaders.C Sharing Conspir.Greatergood Sharing Conspir.Greatergood.C Sharing Expert.FluVaccine Sharing Expert.FluVaccine.C Sharing Expert.HPV Sharing Expert.HPV.C Sharing Expert.Autism Sharing Expert.Autism.C Sharing Natural.Glyphosate Sharing Natural.Glyphosate.C Sharing Natural.Homeopathy Sharing Natural.Homeopathy.C Sharing Natural.Viruses Sharing Natural.Viruses.C Sharing Story.Multiple Sharing Story.Multiple.C Sharing Story.VaccineCourt Sharing Story.VaccineCourt.C Sharing Story.Hepatitis Sharing Story.Hepatitis.C Sharing

7.198

2.519

2.636

1.437

0.373

0.412

5.160

1.256

0.628

2.145

1.374

0.198

1.383

0.848

1.140

2.198

0.008

0.170

6.649

0.434

0.770

0.298

3.989

0.221

2

2

2

2

2

2

2

2

2

2

2

2

2

2

2

2

2

2

2

2

2

2

2

2

0.284

0.268

0.487

0.830

0.814

0.076

0.534

0.731

0.342

0.503

0.906

0.501

0.654

0.566

0.333

0.996

0.918

**0.036**

0.805

0.681

0.861

0.136

0.895

**56 of** [**75**](#_bookmark21)

**One-Way ANOVA items**

**Table S37. Item-Level One-Way ANOVA (Welch) for Study 1: Manipulativeness**

Variable

F

df1

df2

p

Conspir.Database Manipulativeness Conspir.Database.C Manipulativeness Conspir.Leaders Manipulativeness Conspir.Leaders.C Manipulativeness Conspir.Greatergood Manipulativeness Conspir.Greatergood.C Manipulativeness Expert.FluVaccine Manipulativeness Expert.FluVaccine.C Manipulativeness Expert.HPV Manipulativeness Expert.HPV.C Manipulativeness Expert.Autism Manipulativeness Expert.Autism.C Manipulativeness Natural.Glyphosate Manipulativeness Natural.Glyphosate.C Manipulativeness Natural.Homeopathy Manipulativeness Natural.Homeopathy.C Manipulativeness Natural.Viruses Manipulativeness Natural.Viruses.C Manipulativeness Story.Multiple Manipulativeness Story.Multiple.C Manipulativeness Story.VaccineCourt Manipulativeness Story.VaccineCourt.C Manipulativeness Story.Hepatitis Manipulativeness Story.Hepatitis.C Manipulativeness

1.113

0.654

1.385

0.413

3.044

0.237

0.730

3.591

2.517

2.218

1.682

0.892

0.241

1.538

2.884

1.234

0.700

1.386

9.466

0.663

4.104

1.503

6.631

1.513

2

2

2

2

2

2

2

2

2

2

2

2

2

2

2

2

2

2

2

2

2

2

2

2

224.919

225.405

220.015

232.067

216.289

227.396

227.658

226.300

218.557

225.217

219.893

225.254

225.650

224.535

223.300

228.851

218.893

227.902

218.485

223.543

229.218

221.425

227.423

227.268

0.330

0.521

0.252

0.662

**0.050**

0.789

0.483

**0.029**

0.083

0.111

0.188

0.411

0.786

0.217

0.058

0.293

0.498

0.252

**<0.001**

0.517

**0.018**

0.225

**0.002**

0.222

**Table S38. Item-Level One-Way ANOVA (Welch) for Study 1: Confidence**

Variable

F

df1

df2

p

Conspir.Database Confidence Conspir.Database.C Confidence Conspir.Leaders Confidence Conspir.Leaders.C Confidence Conspir.Greatergood Confidence Conspir.Greatergood.C Confidence Expert.FluVaccine Confidence Expert.FluVaccine.C Confidence Expert.HPV Confidence Expert.HPV.C Confidence Expert.Autism Confidence Expert.Autism.C Confidence Natural.Glyphosate Confidence Natural.Glyphosate.C Confidence Natural.Homeopathy Confidence Natural.Homeopathy.C Confidence Natural.Viruses Confidence Natural.Viruses.C Confidence Story.Multiple Confidence Story.Multiple.C Confidence Story.VaccineCourt Confidence Story.VaccineCourt.C Confidence Story.Hepatitis Confidence Story.Hepatitis.C Confidence

1.258

0.180

0.529

0.508

0.531

0.608

0.517

0.071

0.555

0.677

0.899

0.504

0.579

0.457

1.806

2.747

2.626

0.666

0.711

0.778

0.268

0.111

2.372

1.774

2

2

2

2

2

2

2

2

2

2

2

2

2

2

2

2

2

2

2

2

2

2

2

2

221.127

225.341

222.763

228.902

219.548

224.881

228.385

224.497

226.904

224.055

226.241

225.638

229.285

224.804

224.792

225.603

216.414

226.677

231.558

223.801

227.687

221.035

227.950

225.096

0.286

0.836

0.590

0.603

0.589

0.545

0.597

0.932

0.575

0.509

0.408

0.605

0.561

0.634

0.167

0.066

0.075

0.515

0.492

0.460

0.766

0.895

0.096

0.172

**57 of** [**75**](#_bookmark21)

**Table S39. Item-Level One-Way ANOVA (Welch) for Study 1: Sharing**

Variable

F

df1

df2

p

**0.016**

Conspir.Database Sharing Conspir.Database.C Sharing Conspir.Leaders Sharing Conspir.Leaders.C Sharing Conspir.Greatergood Sharing Conspir.Greatergood.C Sharing Expert.FluVaccine Sharing Expert.FluVaccine.C Sharing Expert.HPV Sharing Expert.HPV.C Sharing Expert.Autism Sharing Expert.Autism.C Sharing Natural.Glyphosate Sharing Natural.Glyphosate.C Sharing Natural.Homeopathy Sharing Natural.Homeopathy.C Sharing Natural.Viruses Sharing Natural.Viruses.C Sharing Story.Multiple Sharing Story.Multiple.C Sharing Story.VaccineCourt Sharing Story.VaccineCourt.C Sharing Story.Hepatitis Sharing Story.Hepatitis.C Sharing

4.197

0.752

1.449

0.028

0.643

3.478

1.124

1.145

0.112

4.480

0.542

3.460

1.026

0.036

0.191

1.682

0.214

0.660

3.102

2.103

1.058

0.200

1.282

1.736

2

2

2

2

2

2

2

2

2

2

2

2

2

2

2

2

2

2

2

2

2

2

2

2

225.896

222.845

222.397

230.523

222.798

227.202

228.297

226.359

221.611

221.420

223.618

227.363

229.286

225.143

225.952

226.673

218.901

228.508

230.455

223.941

228.486

221.161

227.688

226.004

0.472

0.237

0.972

0.527

**0.033**

0.327

0.320

0.894

**0.012**

0.582

**0.033**

0.360

0.964

0.826

0.188

0.807

0.518

**0.047**

0.125

0.349

0.819

0.279

0.179

**Study 2.**

***Bartlett Test of Homogeneity of Variances.***

**Table S40. Bartlett Test of Homogeneity of Variances for Study 2**

Variable

Bartlett’s K-squared

*df*

*p*

**0.01**

Misinformation Manipulativeness

Non-Misinformation Manipulativeness Misinformation Manipulativeness Manipulativeness Discernment Misinformation Confidence

Non-Misinformation Confidence Misinformation Sharing Intent

Non-Misinformation Sharing Intent Sharing Intent Discernment

10.31

0.94

10.31

3.62

2.66

1.53

3.91

0.40

1.65

2

2

2

2

2

2

2

2

2

0.62

**0.01**

0.16

0.27

0.47

0.14

0.82

0.44

**58 of** [**75**](#_bookmark21)

***Linear Regression Models.***

**Manipulativess**

**Table S41. Linear Regression Model for Manipulativeness Ratings (Fake Items Only) for Study 2**

Predictor

95% CI

*df*

*b*

*t*

*p*

Intercept ConditionEvil ConditionGood Having Children Gender simpleMale Gender simpleOther

Political Ideology AllLiberal Political Ideology AllConservative COVID Vaccine Intentions

Age Education

News Checking Social Media Use

4.86

0.35

0.32

-0.32

-0.27

0.23

0.21

0.03

0.89

0.08

-0.03

0.03

-0.05

[4.20, 5.52]

[0.13, 0.56]

[0.11, 0.54]

[-0.52, -0.12]

[-0.46, -0.09]

[-0.32, 0.79]

[-0.05, 0.46]

[-0.27, 0.32]

[0.64, 1.14]

[0.00, 0.16]

[-0.10, 0.04]

[-0.08, 0.14]

[-0.16, 0.05]

14.44

3.18

2.94

-3.15

-2.92

0.83

1.60

0.17

7.02

1.86

-0.80

0.56

-0.97

542

542

542

542

542

542

542

542

542

542

542

542

542

< .001

.002

.003

.002

.004

.409

.109

.865

< .001

.063

.427

.578

.330

**Table S42. Linear Regression Model for Manipulativeness Ratings (Real Items Only) for Study 2**

Predictor

95% CI

*df*

*b*

*t*

*p*

Intercept ConditionEvil ConditionGood Having Children Gender simpleMale Gender simpleOther

Political Ideology AllLiberal Political Ideology AllConservative COVID Vaccine Intentions

Age Education

News Checking Social Media Use

3.68

0.13

-0.03

0.20

0.11

-0.30

-0.20

0.03

-0.07

-0.04

0.00

-0.15

0.00

[3.00, 4.35]

[-0.09, 0.35]

[-0.25, 0.19]

[0.00, 0.41]

[-0.08, 0.30]

[-0.86, 0.27]

[-0.45, 0.06]

[-0.27, 0.34]

[-0.32, 0.18]

[-0.13, 0.05]

[-0.07, 0.08]

[-0.27, -0.04]

[-0.11, 0.11]

10.73

1.20

-0.23

1.94

1.14

-1.03

-1.48

0.20

-0.53

-0.91

0.13

-2.70

0.01

542

542

542

542

542

542

542

542

542

542

542

542

542

< .001

.232

.815

.053

.253

.305

.139

.842

.593

.361

.894

.007

.992

**Table S43. Linear Regression Model for Manipulativeness Ratings (Difference between Fake and Real) for Study 2**

Predictor

95% CI

*df*

*b*

*t*

*p*

Intercept ConditionEvil ConditionGood Having Children Gender simpleMale Gender simpleOther

Political Ideology AllLiberal Political Ideology AllConservative COVID Vaccine Intentions

Age Education

News Checking Social Media Use

1.18

0.21

0.35

-0.52

-0.38

0.53

0.40

-0.01

0.96

0.12

-0.03

0.18

-0.05

[0.26, 2.10]

[-0.08, 0.51]

[0.05, 0.65]

[-0.80, -0.24]

[-0.64, -0.13]

[-0.24, 1.30]

[0.05, 0.76]

[-0.42, 0.41]

[0.61, 1.31]

[0.00, 0.24]

[-0.13, 0.07]

[0.03, 0.34]

[-0.21, 0.10]

2.52

1.41

2.28

-3.68

-2.94

1.34

2.24

-0.02

5.43

2.01

-0.67

2.38

-0.71

542

542

542

542

542

542

542

542

542

542

542

542

542

.012

.160

.023

< .001

.003

.179

.026

.981

< .001

.045

.504

.018

.480

**59 of** [**75**](#_bookmark21)

**Confidence**

**Table S44. Linear Regression Model for Confidence Ratings (Fake Items Only) for Study 2**

Predictor

95% CI

*df*

*b*

*t*

*p*

Intercept ConditionEvil ConditionGood Having Children Gender simpleMale Gender simpleOther

Political Ideology AllLiberal Political Ideology AllConservative COVID Vaccine Intentions

Age Education

News Checking Social Media Use

5.05

0.25

0.17

-0.10

-0.09

0.20

0.12

-0.16

0.37

0.04

-0.02

0.13

-0.02

[4.45, 5.65]

[0.06, 0.45]

[-0.03, 0.37]

[-0.29, 0.08]

[-0.25, 0.08]

[-0.31, 0.70]

[-0.11, 0.35]

[-0.43, 0.11]

[0.14, 0.60]

[-0.04, 0.11]

[-0.08, 0.05]

[0.03, 0.23]

[-0.12, 0.08]

16.47

2.53

1.69

-1.13

-1.00

0.76

1.00

-1.14

3.20

0.96

-0.54

2.53

-0.41

542

542

542

542

542

542

542

542

542

542

542

542

542

< .001

.012

.092

.260

.315

.448

.320

.256

.001

.339

.588

.012

.680

**Table S45. Linear Regression Model for Confidence Ratings (Real Items Only) for Study 2**

Predictor

95% CI

*df*

*b*

*t*

*p*

Intercept ConditionEvil ConditionGood Having Children Gender simpleMale Gender simpleOther

Political Ideology AllLiberal Political Ideology AllConservative COVID Vaccine Intentions

Age Education

News Checking Social Media Use

5.20

-0.17

-0.11

0.07

-0.09

0.60

0.09

-0.25

-0.14

0.06

0.04

0.02

0.03

[4.57, 5.82]

[-0.37, 0.03]

[-0.31, 0.10]

[-0.12, 0.26]

[-0.27, 0.08]

[0.07, 1.12]

[-0.15, 0.33]

[-0.54, 0.03]

[-0.37, 0.10]

[-0.02, 0.14]

[-0.03, 0.11]

[-0.08, 0.12]

[-0.07, 0.13]

16.31

-1.63

-1.02

0.76

-1.05

2.23

0.74

-1.77

-1.15

1.48

1.16

0.36

0.58

542

542

542

542

542

542

542

542

542

542

542

542

542

< .001

.104

.306

.450

.294

.026

.461

.078

.251

.139

.248

.720

.563

**60 of** [**75**](#_bookmark21)

**Sharing Intent**

**Table S46. Linear Regression Model for Sharing Ratings (Fake Items Only) for Study 2**

Predictor

95% CI

*df*

*b*

*t*

*p*

Intercept ConditionEvil ConditionGood Having Children Gender simpleMale Gender simpleOther

Political Ideology AllLiberal Political Ideology AllConservative COVID Vaccine Intentions

Age Education

News Checking Social Media Use

1.72

-0.17

-0.15

0.42

0.40

-0.14

0.01

0.14

-0.70

-0.08

-0.01

0.07

0.09

[0.99, 2.45]

[-0.41, 0.07]

[-0.39, 0.09]

[0.19, 0.64]

[0.20, 0.61]

[-0.75, 0.47]

[-0.27, 0.29]

[-0.19, 0.47]

[-0.97, -0.42]

[-0.17, 0.01]

[-0.09, 0.07]

[-0.05, 0.19]

[-0.03, 0.21]

4.65

-1.42

-1.24

3.70

3.93

-0.45

0.08

0.84

-5.00

-1.68

-0.23

1.20

1.43

542

542

542

542

542

542

542

542

542

542

542

542

542

< .001

.157

.215

< .001

< .001

.650

.937

.399

< .001

.094

.822

.231

.154

**Table S47. Linear Regression Model for Sharing Ratings (Real Items Only) for Study 2**

Predictor

95% CI

*df*

*b*

*t*

*p*

Intercept ConditionEvil ConditionGood Having Children Gender simpleMale Gender simpleOther

Political Ideology AllLiberal Political Ideology AllConservative COVID Vaccine Intentions

Age Education

News Checking Social Media Use

0.87

-0.09

0.04

0.33

0.37

0.87

0.07

-0.04

0.27

0.02

0.00

0.20

0.26

[-0.03, 1.78]

[-0.38, 0.20]

[-0.26, 0.34]

[0.06, 0.61]

[0.12, 0.62]

[0.11, 1.63]

[-0.28, 0.41]

[-0.45, 0.37]

[-0.07, 0.61]

[-0.10, 0.13]

[-0.09, 0.10]

[0.05, 0.35]

[0.11, 0.41]

1.90

-0.60

0.26

2.38

2.88

2.24

0.37

-0.18

1.57

0.26

0.07

2.63

3.41

542

542

542

542

542

542

542

542

542

542

542

542

542

.058

.551

.798

.018

.004

.026

.714

.858

.118

.792

.941

.009

< .001

**Table S48. Linear Regression Model for Sharing Ratings (Difference between Fake and Real) for Study 2**

Predictor

95% CI

*df*

*b*

*t*

*p*

Intercept ConditionEvil ConditionGood Having Children Gender simpleMale Gender simpleOther

Political Ideology AllLiberal Political Ideology AllConservative COVID Vaccine Intentions

Age Education

News Checking Social Media Use

-0.85

0.08

0.19

-0.08

-0.03

1.01

0.05

-0.18

0.97

0.09

0.01

0.13

0.17

[-1.72, 0.02]

[-0.20, 0.36]

[-0.10, 0.47]

[-0.35, 0.18]

[-0.28, 0.21]

[0.28, 1.74]

[-0.28, 0.39]

[-0.57, 0.21]

[0.64, 1.30]

[-0.02, 0.21]

[-0.08, 0.11]

[-0.02, 0.27]

[0.03, 0.31]

-1.92

0.56

1.31

-0.61

-0.28

2.72

0.32

-0.89

5.82

1.68

0.27

1.73

2.36

542

542

542

542

542

542

542

542

542

542

542

542

542

.056

.573

.192

.540

.777

.007

.752

.372

< .001

.093

.790

.084

.019

**61 of** [**75**](#_bookmark21)

***Multilevel Models.***

**Manipulativess**

**Table S49. Multilevel Model for Manipulativeness Ratings (All Items) for Study 2**

*β*ˆ

Term

95% CI

*df*

*t*

*p*

Intercept ConditionEvil ConditionGood Matched controlTRUE Having Children Gender simpleMale Gender simpleOther

Political Ideology AllLiberal Political Ideology AllConservative COVID Vaccine Intentions

Age scaled Education scaled

News Checking scaled Social Media Use scaled

ConditionEvil *×* Matched controlTRUE ConditionGood *×* Matched controlTRUE **Random Effects**

*σ*2

*τ*00 Participant *τ*00 Item NParticipant NItem Marginal R2 Conditional R2

5.15

0.38

0.41

-2.33

-0.07

-0.08

0.07

0.00

0.04

0.40

0.04

-0.01

-0.06

0.00

-0.15

-0.37

[4.64, 5.67]

[0.19, 0.57]

[0.22, 0.59]

[-2.95, -1.70]

[-0.22, 0.07]

[-0.21, 0.06]

[-0.34, 0.47]

[-0.19, 0.18]

[-0.17, 0.26]

[0.22, 0.58]

[-0.03, 0.12]

[-0.08, 0.06]

[-0.13, 0.01]

[-0.07, 0.06]

[-0.33, 0.04]

[-0.56, -0.19]

19.58

4.01

4.32

-7.35

-0.97

-1.15

0.32

-0.01

0.39

4.30

1.17

-0.36

-1.73

-0.08

-1.55

-3.97

37.77

1044.79

1000.35

23.27

542.13

541.83

542.09

542.37

542.08

541.97

541.96

541.68

542.00

543.23

6360.18

6358.60

< .001

< .001

< .001

< .001

.331

.252

.751

.992

.695

< .001

.243

.716

.085

.933

.121

< .001

2.32

0.40

0.57

555

24

0.327

0.525

**Confidence**

**Table S50. Multilevel Model for Confidence Ratings (All Items) for Study 2**

*β*ˆ

Term

95% CI

*df*

*t*

*p*

Intercept ConditionEvil ConditionGood Matched controlTRUE Having Children Gender simpleMale Gender simpleOther

Political Ideology AllLiberal Political Ideology AllConservative COVID Vaccine Intentions

Age scaled Education scaled

News Checking scaled Social Media Use scaled

ConditionEvil *×* Matched controlTRUE ConditionGood *×* Matched controlTRUE **Random Effects**

*σ*2

*τ*00 Participant *τ*00 Item NParticipant NItem Marginal R2 Conditional R2

5.63

0.25

0.18

-0.12

0.00

-0.09

0.39

0.12

-0.19

0.12

0.06

0.02

0.07

0.01

-0.36

-0.25

1.23

0.62

0.09

555

24

0.037

0.389

[5.30, 5.96]

[0.06, 0.44]

[-0.01, 0.37]

[-0.38, 0.14]

[-0.16, 0.16]

[-0.24, 0.06]

[-0.05, 0.84]

[-0.09, 0.32]

[-0.43, 0.05]

[-0.08, 0.32]

[-0.03, 0.14]

[-0.06, 0.09]

[-0.01, 0.15]

[-0.06, 0.09]

[-0.49, -0.23]

[-0.39, -0.12]

33.49

2.57

1.90

-0.92

0.00

-1.22

1.73

1.11

-1.57

1.21

1.34

0.44

1.77

0.30

-5.24

-3.67

203.56

751.78

733.93

26.41

542.14

542.01

542.12

542.24

542.11

542.07

542.06

541.94

542.08

542.61

6214.56

6213.73

< .001

.010

.058

.364

.997

.224

.085

.267

.117

.228

.180

.658

.078

.763

< .001

< .001

**62 of** [**75**](#_bookmark21)

**Sharing Intent**

**Table S51. Multilevel Model for Sharing Ratings (All Items) for Study 2**

*β*ˆ

Term

95% CI

*df*

*t*

*p*

Intercept ConditionEvil ConditionGood Matched controlTRUE Having Children Gender simpleMale Gender simpleOther

Political Ideology AllLiberal Political Ideology AllConservative COVID Vaccine Intentions

Age scaled Education scaled

News Checking scaled Social Media Use scaled

ConditionEvil *×* Matched controlTRUE ConditionGood *×* Matched controlTRUE **Random Effects**

*σ*2

*τ*00 Participant *τ*00 Item NParticipant NItem Marginal R2 Conditional R2

1.71

-0.13

-0.16

1.32

0.38

0.42

0.25

0.04

0.01

-0.19

-0.03

-0.01

0.13

0.14

-0.01

0.12

1.84

1.09

0.34

555

24

0.150

0.521

[1.21, 2.21]

[-0.38, 0.11]

[-0.41, 0.08]

[0.83, 1.80]

[0.16, 0.59]

[0.22, 0.61]

[-0.34, 0.83]

[-0.23, 0.31]

[-0.31, 0.32]

[-0.45, 0.07]

[-0.14, 0.07]

[-0.11, 0.09]

[0.02, 0.23]

[0.04, 0.23]

[-0.17, 0.16]

[-0.04, 0.29]

6.67

-1.07

-1.30

5.35

3.48

4.22

0.83

0.30

0.04

-1.43

-0.62

-0.18

2.37

2.75

-0.08

1.48

90.98

724.34

708.91

23.72

542.00

541.88

541.98

542.09

541.98

541.94

541.93

541.82

541.95

542.43

6198.22

6197.45

< .001

.285

.193

< .001

< .001

< .001

.407

.768

.968

.154

.535

.854

.018

.006

.934

.138

***Item-Level Results.***

**Bartlett test items**

**Table S52. Item-Level Bartlett Test of Homogeneity of Variances for Study 2: Manipulativeness**

Variable

Bartlett’s K-squared

*df*

*p*

**0.001**

Conspir.Database Manipulativeness Conspir.Database.C Manipulativeness Conspir.Leaders Manipulativeness Conspir.Leaders.C Manipulativeness Conspir.Greatergood Manipulativeness Conspir.Greatergood.C Manipulativeness Expert.FluVaccine Manipulativeness Expert.FluVaccine.C Manipulativeness Expert.HPV Manipulativeness Expert.HPV.C Manipulativeness Expert.Autism Manipulativeness Expert.Autism.C Manipulativeness Natural.Glyphosate Manipulativeness Natural.Glyphosate.C Manipulativeness Natural.Homeopathy Manipulativeness Natural.Homeopathy.C Manipulativeness Natural.Viruses Manipulativeness Natural.Viruses.C Manipulativeness Story.Multiple Manipulativeness Story.Multiple.C Manipulativeness Story.VaccineCourt Manipulativeness Story.VaccineCourt.C Manipulativeness Story.Hepatitis Manipulativeness Story.Hepatitis.C Manipulativeness

13.279

0.017

7.339

0.105

6.722

1.652

1.776

1.101

3.452

3.741

7.708

0.065

9.626

0.202

6.598

2.436

0.314

0.605

3.052

0.072

2.492

1.599

7.860

22.459

2

2

2

2

2

2

2

2

2

2

2

2

2

2

2

2

2

2

2

2

2

2

2

2

0.991

**0.025**

0.949

**0.035**

0.438

0.412

0.577

0.178

0.154

**0.021**

0.968

**0.008**

0.904

**0.037**

0.296

0.855

0.739

0.217

0.964

0.288

0.450

**0.020**

**<0.001**

**63 of** [**75**](#_bookmark21)

**Table S53. Item-Level Bartlett Test of Homogeneity of Variances for Study 2: Confidence**

Variable

Bartlett’s K-squared

*df*

*p*

**0.043**

Conspir.Database Confidence Conspir.Database.C Confidence Conspir.Leaders Confidence Conspir.Leaders.C Confidence Conspir.Greatergood Confidence Conspir.Greatergood.C Confidence Expert.FluVaccine Confidence Expert.FluVaccine.C Confidence Expert.HPV Confidence Expert.HPV.C Confidence Expert.Autism Confidence Expert.Autism.C Confidence Natural.Glyphosate Confidence Natural.Glyphosate.C Confidence Natural.Homeopathy Confidence Natural.Homeopathy.C Confidence Natural.Viruses Confidence Natural.Viruses.C Confidence Story.Multiple Confidence Story.Multiple.C Confidence Story.VaccineCourt Confidence Story.VaccineCourt.C Confidence Story.Hepatitis Confidence Story.Hepatitis.C Confidence

6.273

1.162

12.450

1.717

2.107

3.109

1.335

0.547

4.331

17.894

1.859

2.725

7.411

0.945

1.026

1.082

2.787

2.721

0.465

1.899

2.744

2.250

0.904

21.679

2

2

2

2

2

2

2

2

2

2

2

2

2

2

2

2

2

2

2

2

2

2

2

2

0.559

**0.002**

0.424

0.349

0.211

0.513

0.761

0.115

**<0.001**

0.395

0.256

**0.025**

0.624

0.599

0.582

0.248

0.256

0.793

0.387

0.254

0.325

0.636

**<0.001**

**Table S54. Item-Level Bartlett Test of Homogeneity of Variances for Study 2: Sharing**

Variable

Bartlett’s K-squared

*df*

*p*

**0.018**

Conspir.Database Sharing Conspir.Database.C Sharing Conspir.Leaders Sharing Conspir.Leaders.C Sharing Conspir.Greatergood Sharing Conspir.Greatergood.C Sharing Expert.FluVaccine Sharing Expert.FluVaccine.C Sharing Expert.HPV Sharing Expert.HPV.C Sharing Expert.Autism Sharing Expert.Autism.C Sharing Natural.Glyphosate Sharing Natural.Glyphosate.C Sharing Natural.Homeopathy Sharing Natural.Homeopathy.C Sharing Natural.Viruses Sharing Natural.Viruses.C Sharing Story.Multiple Sharing Story.Multiple.C Sharing Story.VaccineCourt Sharing Story.VaccineCourt.C Sharing Story.Hepatitis Sharing Story.Hepatitis.C Sharing

8.083

0.445

1.429

0.030

7.347

2.036

1.272

0.125

7.961

0.289

4.447

0.239

2.291

0.648

7.535

0.694

2.623

2.345

9.543

0.158

8.611

0.509

11.482

3.079

2

2

2

2

2

2

2

2

2

2

2

2

2

2

2

2

2

2

2

2

2

2

2

2

0.801

0.490

0.985

**0.025**

0.361

0.529

0.939

**0.019**

0.865

0.108

0.887

0.318

0.723

**0.023**

0.707

0.269

0.310

**0.008**

0.924

**0.013**

0.775

**0.003**

0.214

**64 of** [**75**](#_bookmark21)

**One-Way ANOVA items**

**Table S55. Item-Level One-Way ANOVA (Welch) for Study 2: Manipulativeness**

Variable

F

df1

df2

p

**0.022**

Conspir.Database Manipulativeness Conspir.Database.C Manipulativeness Conspir.Leaders Manipulativeness Conspir.Leaders.C Manipulativeness Conspir.Greatergood Manipulativeness Conspir.Greatergood.C Manipulativeness Expert.FluVaccine Manipulativeness Expert.FluVaccine.C Manipulativeness Expert.HPV Manipulativeness Expert.HPV.C Manipulativeness Expert.Autism Manipulativeness Expert.Autism.C Manipulativeness Natural.Glyphosate Manipulativeness Natural.Glyphosate.C Manipulativeness Natural.Homeopathy Manipulativeness Natural.Homeopathy.C Manipulativeness Natural.Viruses Manipulativeness Natural.Viruses.C Manipulativeness Story.Multiple Manipulativeness Story.Multiple.C Manipulativeness Story.VaccineCourt Manipulativeness Story.VaccineCourt.C Manipulativeness Story.Hepatitis Manipulativeness Story.Hepatitis.C Manipulativeness

3.877

2.480

0.841

0.077

0.573

1.491

0.360

0.120

5.873

2.781

0.907

0.001

4.650

1.342

8.127

0.773

2.731

3.547

2.733

0.046

1.542

0.311

5.735

10.216

2

2

2

2

2

2

2

2

2

2

2

2

2

2

2

2

2

2

2

2

2

2

2

2

177.650

183.030

184.971

181.865

180.072

185.189

179.807

181.665

179.040

186.093

182.756

181.941

185.785

180.405

182.321

177.343

184.693

178.298

174.798

172.809

177.236

183.124

175.941

185.190

0.087

0.433

0.926

0.565

0.228

0.698

0.887

**0.003**

0.065

0.406

0.999

**0.011**

0.264

**<0.001**

0.463

0.068

**0.031**

0.068

0.955

0.217

0.733

**0.004**

**<0.001**

**Table S56. Item-Level One-Way ANOVA (Welch) for Study 2: Confidence**

Variable

F

df1

df2

p

Conspir.Database Confidence Conspir.Database.C Confidence Conspir.Leaders Confidence Conspir.Leaders.C Confidence Conspir.Greatergood Confidence Conspir.Greatergood.C Confidence Expert.FluVaccine Confidence Expert.FluVaccine.C Confidence Expert.HPV Confidence Expert.HPV.C Confidence Expert.Autism Confidence Expert.Autism.C Confidence Natural.Glyphosate Confidence Natural.Glyphosate.C Confidence Natural.Homeopathy Confidence Natural.Homeopathy.C Confidence Natural.Viruses Confidence Natural.Viruses.C Confidence Story.Multiple Confidence Story.Multiple.C Confidence Story.VaccineCourt Confidence Story.VaccineCourt.C Confidence Story.Hepatitis Confidence Story.Hepatitis.C Confidence

2.328

0.344

1.675

0.111

0.006

0.430

0.628

0.171

1.030

2.091

0.574

0.107

5.569

1.398

1.407

0.133

1.737

0.867

3.142

2.092

3.813

0.889

2.196

3.238

2

2

2

2

2

2

2

2

2

2

2

2

2

2

2

2

2

2

2

2

2

2

2

2

181.925

181.809

184.865

181.283

177.326

185.897

181.839

178.767

178.738

181.054

183.933

181.136

185.757

181.309

184.129

177.579

181.467

178.545

178.986

169.389

181.635

184.663

171.538

176.832

0.100

0.709

0.190

0.895

0.994

0.651

0.535

0.843

0.359

0.127

0.564

0.899

**0.004**

0.250

0.247

0.875

0.179

0.422

**0.046**

0.127

**0.024**

0.413

0.114

**0.042**

**65 of** [**75**](#_bookmark21)

**Table S57. Item-Level One-Way ANOVA (Welch) for Study 2: Sharing**

Variable

F

df1

df2

p

Conspir.Database Sharing Conspir.Database.C Sharing Conspir.Leaders Sharing Conspir.Leaders.C Sharing Conspir.Greatergood Sharing Conspir.Greatergood.C Sharing Expert.FluVaccine Sharing Expert.FluVaccine.C Sharing Expert.HPV Sharing Expert.HPV.C Sharing Expert.Autism Sharing Expert.Autism.C Sharing Natural.Glyphosate Sharing Natural.Glyphosate.C Sharing Natural.Homeopathy Sharing Natural.Homeopathy.C Sharing Natural.Viruses Sharing Natural.Viruses.C Sharing Story.Multiple Sharing Story.Multiple.C Sharing Story.VaccineCourt Sharing Story.VaccineCourt.C Sharing Story.Hepatitis Sharing Story.Hepatitis.C Sharing

1.423

0.304

0.003

0.593

0.113

0.924

0.252

0.124

1.926

0.459

1.238

0.478

1.319

0.005

3.324

0.098

1.557

2.495

3.050

0.638

1.202

0.409

2.391

1.448

2

2

2

2

2

2

2

2

2

2

2

2

2

2

2

2

2

2

2

2

2

2

2

2

181.317

182.319

180.010

181.866

179.147

186.577

181.222

179.645

178.812

187.278

179.812

181.654

185.533

180.611

182.072

177.940

184.782

179.287

169.592

173.715

177.544

183.209

174.318

179.916

0.244

0.738

0.997

0.554

0.893

0.399

0.777

0.884

0.149

0.632

0.292

0.621

0.270

0.995

**0.038**

0.907

0.213

0.085

**0.050**

0.530

0.303

0.665

0.095

0.238

**Study 3.**

***Bartlett Test of Homogeneity of Variances.***

**Table S58. Bartlett Test of Homogeneity of Variances for Study 3**

Variable

Bartlett’s K-squared

*df*

*p*

**0.00**

Misinformation Manipulativeness

Non-Misinformation Manipulativeness Misinformation Manipulativeness Manipulativeness Discernment Misinformation Confidence

Non-Misinformation Confidence Misinformation Sharing Intent

Non-Misinformation Sharing Intent Sharing Intent Discernment

12.59

4.70

12.59

6.13

5.92

3.93

15.31

0.08

6.60

2

2

2

2

2

2

2

2

2

0.10

**0.00**

**0.05**

0.05

0.14

**<0.001**

0.96

**0.04**

**66 of** [**75**](#_bookmark21)

***Linear Regression Models.***

**Manipulativess**

**Table S59. Linear Regression Model for Manipulativeness Ratings (Fake Items Only) for Study 3**

Predictor

95% CI

*df*

*b*

*t*

*p*

Intercept ConditionEvil ConditionGood Having Children Gender simpleMale Gender simpleOther

Political Ideology AllLiberal Political Ideology AllConservative COVID Vaccine Intentions

Age Education

News Checking Social Media Use

3.77

0.43

0.33

-0.06

0.06

0.00

0.49

0.09

0.84

0.05

-0.01

0.13

0.01

[3.36, 4.18]

[0.29, 0.58]

[0.18, 0.47]

[-0.21, 0.08]

[-0.06, 0.19]

[-0.42, 0.42]

[0.33, 0.64]

[-0.11, 0.28]

[0.66, 1.02]

[-0.01, 0.10]

[-0.06, 0.03]

[0.06, 0.20]

[-0.06, 0.07]

17.98

5.80

4.45

-0.88

1.00

-0.01

6.29

0.87

9.07

1.74

-0.61

3.76

0.23

1062

1062

1062

1062

1062

1062

1062

1062

1062

1062

1062

1062

1062

< .001

< .001

< .001

.379

.318

.994

< .001

.382

< .001

.082

.542

< .001

.818

**Table S60. Linear Regression Model for Manipulativeness Ratings (Real Items Only) for Study 3**

Predictor

95% CI

*df*

*b*

*t*

*p*

Intercept ConditionEvil ConditionGood Having Children Gender simpleMale Gender simpleOther

Political Ideology AllLiberal Political Ideology AllConservative COVID Vaccine Intentions

Age Education

News Checking Social Media Use

3.61

0.10

-0.13

0.07

0.05

-0.14

-0.15

0.01

-0.20

0.00

0.00

-0.05

0.00

[3.17, 4.05]

[-0.06, 0.25]

[-0.28, 0.02]

[-0.08, 0.23]

[-0.09, 0.18]

[-0.58, 0.31]

[-0.31, 0.02]

[-0.20, 0.22]

[-0.39, 0.00]

[-0.06, 0.06]

[-0.05, 0.05]

[-0.12, 0.02]

[-0.07, 0.06]

16.11

1.24

-1.65

0.90

0.67

-0.61

-1.76

0.10

-2.01

-0.11

0.13

-1.34

-0.13

1062

1062

1062

1062

1062

1062

1062

1062

1062

1062

1062

1062

1062

< .001

.214

.098

.366

.501

.542

.079

.924

.045

.910

.899

.181

.897

**Table S61. Linear Regression Model for Manipulativeness Ratings (Difference between Fake and Real) for Study 3**

Predictor

95% CI

*df*

*b*

*t*

*p*

Intercept ConditionEvil ConditionGood Having Children Gender simpleMale Gender simpleOther

Political Ideology AllLiberal Political Ideology AllConservative COVID Vaccine Intentions

Age Education

News Checking Social Media Use

0.16

0.33

0.46

-0.14

0.02

0.14

0.63

0.08

1.04

0.05

-0.02

0.18

0.01

[-0.41, 0.73]

[0.13, 0.53]

[0.26, 0.66]

[-0.34, 0.06]

[-0.16, 0.19]

[-0.44, 0.72]

[0.42, 0.84]

[-0.20, 0.35]

[0.79, 1.29]

[-0.02, 0.13]

[-0.08, 0.05]

[0.09, 0.28]

[-0.08, 0.10]

0.56

3.23

4.49

-1.33

0.20

0.47

5.90

0.56

8.10

1.35

-0.54

3.74

0.27

1062

1062

1062

1062

1062

1062

1062

1062

1062

1062

1062

1062

1062

.577

.001

< .001

.183

.840

.642

< .001

.577

< .001

.179

.590

< .001

.790

**67 of** [**75**](#_bookmark21)

**Confidence**

**Table S62. Linear Regression Model for Confidence Ratings (Fake Items Only) for Study 3**

Predictor

95% CI

*df*

*b*

*t*

*p*

Intercept ConditionEvil ConditionGood Having Children Gender simpleMale Gender simpleOther

Political Ideology AllLiberal Political Ideology AllConservative COVID Vaccine Intentions

Age Education

News Checking Social Media Use

4.66

0.21

0.20

-0.04

-0.05

0.24

0.31

0.09

0.43

0.05

-0.04

0.11

0.03

[4.26, 5.06]

[0.07, 0.35]

[0.05, 0.34]

[-0.18, 0.10]

[-0.17, 0.07]

[-0.17, 0.64]

[0.16, 0.46]

[-0.11, 0.28]

[0.26, 0.61]

[0.00, 0.10]

[-0.09, 0.00]

[0.04, 0.18]

[-0.03, 0.10]

22.87

2.93

2.73

-0.52

-0.76

1.14

4.11

0.88

4.82

1.79

-1.84

3.21

1.03

1062

1062

1062

1062

1062

1062

1062

1062

1062

1062

1062

1062

1062

< .001

.003

.006

.603

.448

.253

< .001

.379

< .001

.074

.066

.001

.303

**Table S63. Linear Regression Model for Confidence Ratings (Real Items Only) for Study 3**

Predictor

95% CI

*df*

*b*

*t*

*p*

Intercept ConditionEvil ConditionGood Having Children Gender simpleMale Gender simpleOther

Political Ideology AllLiberal Political Ideology AllConservative COVID Vaccine Intentions

Age Education

News Checking Social Media Use

4.59

-0.03

0.07

-0.01

-0.05

0.24

0.36

0.21

0.10

0.04

-0.03

0.14

0.01

[4.16, 5.02]

[-0.18, 0.13]

[-0.09, 0.22]

[-0.16, 0.15]

[-0.18, 0.08]

[-0.20, 0.68]

[0.20, 0.52]

[0.01, 0.42]

[-0.09, 0.29]

[-0.01, 0.10]

[-0.08, 0.01]

[0.07, 0.21]

[-0.06, 0.08]

20.77

-0.33

0.86

-0.07

-0.71

1.05

4.37

2.03

1.00

1.47

-1.41

3.79

0.37

1062

1062

1062

1062

1062

1062

1062

1062

1062

1062

1062

1062

1062

< .001

.741

.389

.946

.477

.294

< .001

.042

.317

.141

.159

< .001

.714

**68 of** [**75**](#_bookmark21)

**Sharing Intent**

**Table S64. Linear Regression Model for Sharing Ratings (Fake Items Only) for Study 3**

Predictor

95% CI

*df*

*b*

*t*

*p*

Intercept ConditionEvil ConditionGood Having Children Gender simpleMale Gender simpleOther

Political Ideology AllLiberal Political Ideology AllConservative COVID Vaccine Intentions

Age Education

News Checking Social Media Use

2.62

0.07

-0.06

0.25

0.08

-0.31

-0.51

-0.05

-0.54

0.00

-0.02

-0.03

0.05

[2.10, 3.14]

[-0.11, 0.26]

[-0.24, 0.13]

[0.07, 0.43]

[-0.07, 0.24]

[-0.84, 0.21]

[-0.70, -0.32]

[-0.30, 0.19]

[-0.76, -0.31]

[-0.07, 0.07]

[-0.08, 0.04]

[-0.12, 0.05]

[-0.03, 0.13]

9.94

0.78

-0.61

2.70

1.04

-1.17

-5.29

-0.43

-4.60

-0.08

-0.66

-0.79

1.12

1062

1062

1062

1062

1062

1062

1062

1062

1062

1062

1062

1062

1062

< .001

.436

.541

.007

.300

.243

< .001

.665

< .001

.938

.512

.432

.265

**Table S65. Linear Regression Model for Sharing Ratings (Real Items Only) for Study 3**

Predictor

95% CI

*df*

*b*

*t*

*p*

Intercept ConditionEvil ConditionGood Having Children Gender simpleMale Gender simpleOther

Political Ideology AllLiberal Political Ideology AllConservative COVID Vaccine Intentions

Age Education

News Checking Social Media Use

1.67

0.29

0.50

0.08

0.11

0.55

0.00

-0.16

0.19

0.04

0.01

0.21

0.05

[1.07, 2.26]

[0.08, 0.50]

[0.29, 0.71]

[-0.13, 0.29]

[-0.07, 0.29]

[-0.06, 1.16]

[-0.22, 0.22]

[-0.45, 0.12]

[-0.08, 0.45]

[-0.04, 0.12]

[-0.06, 0.08]

[0.11, 0.31]

[-0.05, 0.14]

5.47

2.68

4.71

0.78

1.20

1.78

-0.03

-1.13

1.40

0.99

0.31

4.11

0.98

1062

1062

1062

1062

1062

1062

1062

1062

1062

1062

1062

1062

1062

< .001

.007

< .001

.433

.229

.076

.975

.258

.161

.323

.757

< .001

.330

**Table S66. Linear Regression Model for Sharing Ratings (Difference between Fake and Real) for Study 3**

Predictor

95% CI

*df*

*b*

*t*

*p*

Intercept ConditionEvil ConditionGood Having Children Gender simpleMale Gender simpleOther

Political Ideology AllLiberal Political Ideology AllConservative COVID Vaccine Intentions

Age Education

News Checking Social Media Use

-0.96

0.22

0.56

-0.17

0.03

0.86

0.51

-0.11

0.72

0.04

0.03

0.24

0.00

[-1.50, -0.41]

[0.02, 0.41]

[0.37, 0.75]

[-0.36, 0.03]

[-0.14, 0.19]

[0.31, 1.42]

[0.31, 0.71]

[-0.37, 0.15]

[0.48, 0.97]

[-0.03, 0.12]

[-0.03, 0.09]

[0.15, 0.34]

[-0.09, 0.09]

-3.43

2.19

5.72

-1.70

0.33

3.05

4.97

-0.83

5.89

1.15

0.96

5.23

0.01

1062

1062

1062

1062

1062

1062

1062

1062

1062

1062

1062

1062

1062

< .001

.028

< .001

.090

.739

.002

< .001

.409

< .001

.249

.337

< .001

.993

**69 of** [**75**](#_bookmark21)

***Multilevel Models.***

**Manipulativess**

**Table S67. Multilevel Model for Manipulativeness Ratings (All Items) for Study 3**

*β*ˆ

Term

95% CI

*df*

*t*

*p*

Intercept ConditionEvil ConditionGood Matched controlTRUE Having Children Gender simpleMale Gender simpleOther

Political Ideology AllLiberal Political Ideology AllConservative COVID Vaccine Intentions

Age scaled Education scaled

News Checking scaled Social Media Use scaled

ConditionEvil *×* Matched controlTRUE ConditionGood *×* Matched controlTRUE **Random Effects**

*σ*2

*τ*00 Participant *τ*00 Item NParticipant NItem Marginal R2 Conditional R2

5.04

0.41

0.34

-2.00

-0.01

0.03

0.00

0.15

0.00

0.26

0.02

0.01

0.06

-0.01

-0.28

-0.47

[4.58, 5.50]

[0.28, 0.54]

[0.21, 0.48]

[-2.57, -1.42]

[-0.12, 0.10]

[-0.06, 0.13]

[-0.32, 0.32]

[0.03, 0.26]

[-0.15, 0.15]

[0.12, 0.40]

[-0.04, 0.08]

[-0.04, 0.06]

[0.01, 0.11]

[-0.06, 0.04]

[-0.42, -0.15]

[-0.60, -0.34]

21.55

6.04

5.14

-6.77

-0.18

0.65

0.03

2.46

0.04

3.64

0.65

0.42

2.24

-0.48

-4.22

-7.02

30.60

2050.90

2022.71

22.72

1061.36

1061.08

1061.95

1062.30

1062.04

1061.30

1061.41

1061.77

1061.78

1061.84

12334.67

12344.57

< .001

< .001

< .001

< .001

.855

.514

.979

.014

.969

< .001

.516

.675

.025

.628

< .001

< .001

2.30

0.39

0.51

1,075

24

0.288

0.487

**Confidence**

**Table S68. Multilevel Model for Confidence Ratings (All Items) for Study 3**

*β*ˆ

Term

95% CI

*df*

*t*

*p*

Intercept ConditionEvil ConditionGood Matched controlTRUE Having Children Gender simpleMale Gender simpleOther

Political Ideology AllLiberal Political Ideology AllConservative COVID Vaccine Intentions

Age scaled Education scaled

News Checking scaled Social Media Use scaled

ConditionEvil *×* Matched controlTRUE ConditionGood *×* Matched controlTRUE **Random Effects**

*σ*2

*τ*00 Participant *τ*00 Item NParticipant NItem Marginal R2 Conditional R2

5.31

0.19

0.19

-0.25

-0.03

-0.06

0.20

0.35

0.14

0.22

0.06

-0.04

0.12

0.01

-0.21

-0.12

1.22

0.68

0.08

1,075

24

0.046

0.412

[5.04, 5.57]

[0.05, 0.33]

[0.05, 0.32]

[-0.48, -0.02]

[-0.16, 0.10]

[-0.17, 0.05]

[-0.17, 0.57]

[0.21, 0.48]

[-0.03, 0.32]

[0.06, 0.38]

[-0.01, 0.13]

[-0.10, 0.02]

[0.06, 0.18]

[-0.04, 0.07]

[-0.31, -0.11]

[-0.22, -0.03]

39.19

2.70

2.62

-2.12

-0.46

-1.09

1.05

5.04

1.60

2.65

1.78

-1.39

3.94

0.51

-4.25

-2.50

135.45

1433.50

1423.76

24.57

1061.86

1061.75

1062.09

1062.24

1062.14

1061.84

1061.88

1062.03

1062.03

1062.05

12030.97

12036.02

< .001

.007

.009

.044

.643

.274

.292

< .001

.109

.008

.075

.164

< .001

.609

< .001

.012

**70 of** [**75**](#_bookmark21)

**Sharing Intent**

**Table S69. Multilevel Model for Sharing Ratings (All Items) for Study 3**

*β*ˆ

Term

95% CI

*df*

*t*

*p*

Intercept ConditionEvil ConditionGood Matched controlTRUE Having Children Gender simpleMale Gender simpleOther

Political Ideology AllLiberal Political Ideology AllConservative COVID Vaccine Intentions

Age scaled Education scaled

News Checking scaled Social Media Use scaled

ConditionEvil *×* Matched controlTRUE ConditionGood *×* Matched controlTRUE **Random Effects**

*σ*2

*τ*00 Participant *τ*00 Item NParticipant NItem Marginal R2 Conditional R2

2.16

0.07

-0.09

0.92

0.15

0.11

0.02

-0.27

-0.10

-0.13

0.04

-0.01

0.08

0.06

0.21

0.57

1.86

1.23

0.28

1,075

24

0.109

0.507

[1.74, 2.57]

[-0.11, 0.26]

[-0.27, 0.10]

[0.49, 1.35]

[-0.03, 0.32]

[-0.04, 0.26]

[-0.47, 0.52]

[-0.45, -0.09]

[-0.33, 0.14]

[-0.35, 0.08]

[-0.05, 0.13]

[-0.08, 0.07]

[0.00, 0.16]

[-0.02, 0.13]

[0.09, 0.33]

[0.45, 0.69]

10.18

0.79

-0.92

4.22

1.66

1.42

0.09

-2.97

-0.81

-1.20

0.90

-0.21

2.02

1.45

3.43

9.38

68.37

1380.01

1371.74

23.10

1061.69

1061.59

1061.90

1062.02

1061.93

1061.67

1061.71

1061.84

1061.84

1061.86

12000.37

12004.70

< .001

.432

.358

< .001

.097

.156

.928

.003

.419

.229

.369

.830

.044

.146

< .001

< .001

***Item-Level Results.***

**Bartlett test items**

**Table S70. Item-Level Bartlett Test of Homogeneity of Variances for Study 3: Manipulativeness**

Variable

Bartlett’s K-squared

*df*

*p*

**0.011**

Conspir.Database Manipulativeness Conspir.Database.C Manipulativeness Conspir.Leaders Manipulativeness Conspir.Leaders.C Manipulativeness Conspir.Greatergood Manipulativeness Conspir.Greatergood.C Manipulativeness Expert.FluVaccine Manipulativeness Expert.FluVaccine.C Manipulativeness Expert.HPV Manipulativeness Expert.HPV.C Manipulativeness Expert.Autism Manipulativeness Expert.Autism.C Manipulativeness Natural.Glyphosate Manipulativeness Natural.Glyphosate.C Manipulativeness Natural.Homeopathy Manipulativeness Natural.Homeopathy.C Manipulativeness Natural.Viruses Manipulativeness Natural.Viruses.C Manipulativeness Story.Multiple Manipulativeness Story.Multiple.C Manipulativeness Story.VaccineCourt Manipulativeness Story.VaccineCourt.C Manipulativeness Story.Hepatitis Manipulativeness Story.Hepatitis.C Manipulativeness

9.106

0.789

1.243

0.465

14.819

0.747

1.292

1.602

0.380

4.236

23.330

1.578

3.003

2.754

2.638

1.370

2.428

1.147

0.179

4.256

1.893

0.065

4.877

9.469

2

2

2

2

2

2

2

2

2

2

2

2

2

2

2

2

2

2

2

2

2

2

2

2

0.674

0.537

0.793

**<0.001**

0.688

0.524

0.449

0.827

0.120

**<0.001**

0.454

0.223

0.252

0.267

0.504

0.297

0.563

0.914

0.119

0.388

0.968

0.087

**0.009**

**71 of** [**75**](#_bookmark21)

**Table S71. Item-Level Bartlett Test of Homogeneity of Variances for Study 3: Confidence**

Variable

Bartlett’s K-squared

*df*

*p*

Conspir.Database Confidence Conspir.Database.C Confidence Conspir.Leaders Confidence Conspir.Leaders.C Confidence Conspir.Greatergood Confidence Conspir.Greatergood.C Confidence Expert.FluVaccine Confidence Expert.FluVaccine.C Confidence Expert.HPV Confidence Expert.HPV.C Confidence Expert.Autism Confidence Expert.Autism.C Confidence Natural.Glyphosate Confidence Natural.Glyphosate.C Confidence Natural.Homeopathy Confidence Natural.Homeopathy.C Confidence Natural.Viruses Confidence Natural.Viruses.C Confidence Story.Multiple Confidence Story.Multiple.C Confidence Story.VaccineCourt Confidence Story.VaccineCourt.C Confidence Story.Hepatitis Confidence Story.Hepatitis.C Confidence

2.421

1.089

1.162

1.193

6.438

4.125

4.056

3.248

1.285

0.164

5.440

0.182

1.715

0.401

1.848

12.291

3.554

7.663

9.165

3.419

3.231

5.404

5.629

9.237

2

2

2

2

2

2

2

2

2

2

2

2

2

2

2

2

2

2

2

2

2

2

2

2

0.298

0.580

0.559

0.551

**0.040**

0.127

0.132

0.197

0.526

0.921

0.066

0.913

0.424

0.818

0.397

**0.002**

0.169

**0.022**

**0.010**

0.181

0.199

0.067

0.060

**0.010**

**Table S72. Item-Level Bartlett Test of Homogeneity of Variances for Study 3: Sharing**

Variable

Bartlett’s K-squared

*df*

*p*

**0.008**

Conspir.Database Sharing Conspir.Database.C Sharing Conspir.Leaders Sharing Conspir.Leaders.C Sharing Conspir.Greatergood Sharing Conspir.Greatergood.C Sharing Expert.FluVaccine Sharing Expert.FluVaccine.C Sharing Expert.HPV Sharing Expert.HPV.C Sharing Expert.Autism Sharing Expert.Autism.C Sharing Natural.Glyphosate Sharing Natural.Glyphosate.C Sharing Natural.Homeopathy Sharing Natural.Homeopathy.C Sharing Natural.Viruses Sharing Natural.Viruses.C Sharing Story.Multiple Sharing Story.Multiple.C Sharing Story.VaccineCourt Sharing Story.VaccineCourt.C Sharing Story.Hepatitis Sharing Story.Hepatitis.C Sharing

9.614

0.875

6.393

0.733

3.695

1.078

5.766

1.931

3.459

0.014

15.374

0.165

16.011

0.969

8.610

5.532

4.639

0.298

12.477

0.586

3.881

0.196

7.099

0.134

2

2

2

2

2

2

2

2

2

2

2

2

2

2

2

2

2

2

2

2

2

2

2

2

0.646

**0.041**

0.693

0.158

0.583

0.056

0.381

0.177

0.993

**<0.001**

0.921

**<0.001**

0.616

**0.014**

0.063

0.098

0.862

**0.002**

0.746

0.144

0.907

**0.029**

0.935

**72 of** [**75**](#_bookmark21)

**One-Way ANOVA items**

**Table S73. Item-Level One-Way ANOVA (Welch) for Study 3: Manipulativeness**

Variable

F

df1

df2

p

Conspir.Database Manipulativeness Conspir.Database.C Manipulativeness Conspir.Leaders Manipulativeness Conspir.Leaders.C Manipulativeness Conspir.Greatergood Manipulativeness Conspir.Greatergood.C Manipulativeness Expert.FluVaccine Manipulativeness Expert.FluVaccine.C Manipulativeness Expert.HPV Manipulativeness Expert.HPV.C Manipulativeness Expert.Autism Manipulativeness Expert.Autism.C Manipulativeness Natural.Glyphosate Manipulativeness Natural.Glyphosate.C Manipulativeness Natural.Homeopathy Manipulativeness Natural.Homeopathy.C Manipulativeness Natural.Viruses Manipulativeness Natural.Viruses.C Manipulativeness Story.Multiple Manipulativeness Story.Multiple.C Manipulativeness Story.VaccineCourt Manipulativeness Story.VaccineCourt.C Manipulativeness Story.Hepatitis Manipulativeness Story.Hepatitis.C Manipulativeness

2.900

1.701

0.977

0.831

3.977

1.629

2.519

2.396

5.425

2.226

4.809

2.935

0.359

6.934

6.567

0.578

12.797

2.865

0.728

3.426

3.242

0.459

10.108

1.225

2

2

2

2

2

2

2

2

2

2

2

2

2

2

2

2

2

2

2

2

2

2

2

2

355.613

352.551

356.226

356.474

358.525

353.391

357.209

354.572

356.624

350.332

355.729

357.561

359.229

350.056

357.651

349.805

357.834

353.514

350.011

361.930

354.362

358.130

352.475

359.126

0.056

0.184

0.378

0.437

**0.020**

0.198

0.082

0.093

**0.005**

0.110

**0.009**

0.054

0.698

**0.001**

**0.002**

0.562

**<0.001**

0.058

0.484

**0.034**

**0.040**

0.632

**<0.001**

0.295

**Table S74. Item-Level One-Way ANOVA (Welch) for Study 3: Confidence**

Variable

F

df1

df2

p

Conspir.Database Confidence Conspir.Database.C Confidence Conspir.Leaders Confidence Conspir.Leaders.C Confidence Conspir.Greatergood Confidence Conspir.Greatergood.C Confidence Expert.FluVaccine Confidence Expert.FluVaccine.C Confidence Expert.HPV Confidence Expert.HPV.C Confidence Expert.Autism Confidence Expert.Autism.C Confidence Natural.Glyphosate Confidence Natural.Glyphosate.C Confidence Natural.Homeopathy Confidence Natural.Homeopathy.C Confidence Natural.Viruses Confidence Natural.Viruses.C Confidence Story.Multiple Confidence Story.Multiple.C Confidence Story.VaccineCourt Confidence Story.VaccineCourt.C Confidence Story.Hepatitis Confidence Story.Hepatitis.C Confidence

1.503

0.040

0.225

0.650

1.803

0.974

5.648

0.246

3.358

0.434

0.906

1.015

0.001

3.950

1.827

1.744

4.503

2.097

3.159

0.610

0.216

1.617

5.671

2.875

2

2

2

2

2

2

2

2

2

2

2

2

2

2

2

2

2

2

2

2

2

2

2

2

356.755

352.471

353.676

356.582

359.433

349.550

357.705

356.298

357.752

351.067

354.390

358.563

358.809

354.652

357.904

343.643

355.452

353.950

346.359

360.839

348.073

357.159

352.998

352.654

0.224

0.961

0.799

0.523

0.166

0.378

**0.004**

0.782

**0.036**

0.648

0.405

0.363

0.999

**0.020**

0.162

0.176

**0.012**

0.124

**0.044**

0.544

0.806

0.200

**0.004**

0.058

**73 of** [**75**](#_bookmark21)

**Table S75. Item-Level One-Way ANOVA (Welch) for Study 3: Sharing**

Variable

F

df1

df2

p

Conspir.Database Sharing Conspir.Database.C Sharing Conspir.Leaders Sharing Conspir.Leaders.C Sharing Conspir.Greatergood Sharing Conspir.Greatergood.C Sharing Expert.FluVaccine Sharing Expert.FluVaccine.C Sharing Expert.HPV Sharing Expert.HPV.C Sharing Expert.Autism Sharing Expert.Autism.C Sharing Natural.Glyphosate Sharing Natural.Glyphosate.C Sharing Natural.Homeopathy Sharing Natural.Homeopathy.C Sharing Natural.Viruses Sharing Natural.Viruses.C Sharing Story.Multiple Sharing Story.Multiple.C Sharing Story.VaccineCourt Sharing Story.VaccineCourt.C Sharing Story.Hepatitis Sharing Story.Hepatitis.C Sharing

1.485

0.598

1.464

4.980

0.846

4.258

1.746

5.461

0.060

1.502

4.501

8.001

1.643

1.577

1.834

8.768

1.111

1.962

3.325

7.453

0.183

0.151

2.113

8.180

2

2

2

2

2

2

2

2

2

2

2

2

2

2

2

2

2

2

2

2

2

2

2

2

351.811

352.464

350.910

354.328

354.857

353.655

356.784

352.186

351.896

350.500

349.509

359.083

352.226

353.188

354.145

345.405

356.748

353.124

344.056

358.727

348.936

357.521

347.621

354.097

0.228

0.550

0.233

**0.007**

0.430

**0.015**

0.176

**0.005**

0.942

0.224

**0.012**

**<0.001**

0.195

0.208

0.161

**<0.001**

0.330

0.142

**0.037**

**<0.001**

0.833

0.860

0.122

**<0.001**

**74 of** [**75**](#_bookmark21)

**References**

1.

2.

Sara Balduzzi, Gerta Rücker, and Guido Schwarzer. How to perform a meta-analysis with R: a practical tutorial. *BMJ Ment Health*, 22(4):153–160, 2019. ISSN 1362-0347. .

Gerta Rücker, Ulrike Krahn, Jochem König, Orestis Efthimiou, Annabel Davies, Theodoros Papakonstantinou, and Guido Schwarzer. *netmeta: Network Meta-Analysis using Frequentist Methods*, 2022. URL [https://CRAN.R-project.org/](https://CRAN.R-project.org/package%3Dnetmeta) [package=netmeta](https://CRAN.R-project.org/package%3Dnetmeta). R package version 2.1-0.

William J. McGuire. Inducing Resistance to Persuasion: Some Contemporary Approaches. *Advances in Experimental* *Social Psychology*, 1(C):191–229, 1964. ISSN 00652601. .

D. Papageorgis and W. J. McGuire. The generality of immunity to persuasion produced by pre-exposure to weakened counterarguments. *Journal of Abnormal and Social Psychology*, 62(3):475–481, 1961. ISSN 0096851X. .

Josh Compton and Michael Pfau. Inoculation Theory of Resistance to Influence at Maturity: Recent Progress In Theory Development and Application and Suggestions for Future Research. *Annals of the International Communication* *Association*, 29(1):97–146, 2005. ISSN 2380-8985. .

Josh Compton and Bobi Ivanov. Vaccinating Voters: Surveying Political Campaign Inoculation Scholarship. *Annals of the* *International Communication Association*, 37(1):251–283, 2013. ISSN 2380-8985. .

John A. Banas and Stephen A. Rains. A meta-analysis of research on inoculation theory. *Communication Monographs*, 77 (3):281–311, 2010. ISSN 03637751. .

Cecilie S. Traberg, Jon Roozenbeek, and Sander van der Linden. Psychological Inoculation against Misinformation: Current Evidence and Future Directions. *The ANNALS of the American Academy of Political and Social Science*, 700(1): 136–151, 2022. .

Josh Compton. Prophylactic Versus Therapeutic Inoculation Treatments for Resistance to Influence. *Communication* *Theory*, 30(3):330–343, 2020. ISSN 1050-3293. .

Daniel Jolley and Karen M. Douglas. Prevention is better than cure: Addressing anti-vaccine conspiracy theories. *Journal* *of Applied Social Psychology*, 47(8):459–469, 2017. ISSN 15591816. .

Josh Compton. Inoculation Theory. In James Price Dillard and Lijiang Shen, editors, *The SAGE Handbook of Persuasion*, chapter 14, pages 220–236. SAGE Publications Inc., Thousand Oaks, CA, second edition, 2013. .

Rakoen Maertens, Jon Roozenbeek, Melisa Basol, and Sander van der Linden. Long-Term Effectiveness of Inoculation Against Misinformation: Three Longitudinal Experiments. *Journal of Experimental Psychology: Applied*, 27, 2021. ISSN 1076898X. .

Melisa Basol, Jon Roozenbeek, Manon Berriche, Fatih Uenal, William P. McClanahan, and Sander van der Linden. Towards psychological herd immunity: Cross-cultural evidence for two prebunking interventions against COVID-19 misinformation. *Big Data and Society*, 8(1), 2021. ISSN 20539517. .

Sander van der Linden, Anthony Leiserowitz, Seth Rosenthal, and Edward Maibach. Inoculating the Public against Misinformation about Climate Change. *Global Challenges*, 1(2):1600008, 2017. ISSN 2056-6646. .

Jon Roozenbeek and Sander van der Linden. The fake news game: actively inoculating against the risk of misinformation.

*Journal of Risk Research*, 22(5):570–580, 2019. ISSN 14664461. .

Sandra J. Bean. Emerging and continuing trends in vaccine opposition website content. *Vaccine*, 29(10):1874–1880, 2011. ISSN 0264410X. . URL <http://dx.doi.org/10.1016/j.vaccine.2011.01.003>.

Anna Kata. A postmodern Pandora’s box: Anti-vaccination misinformation on the Internet. *Vaccine*, 28(7):1709–1716, 2010. ISSN 0264410X. .

Anna Kata. Anti-vaccine activists, Web 2.0, and the postmodern paradigm - An overview of tactics and tropes used online by the anti-vaccination movement. *Vaccine*, 30(25):3778–3789, 2012. ISSN 0264410X. . URL [http:](http://dx.doi.org/10.1016/j.vaccine.2011.11.112)

[//dx.doi.org/10.1016/j.vaccine.2011.11.112](http://dx.doi.org/10.1016/j.vaccine.2011.11.112).

Wen Ying Sylvia Chou and Alexandra Budenz. Considering Emotion in COVID-19 Vaccine Communication: Addressing Vaccine Hesitancy and Fostering Vaccine Confidence. *Health Communication*, 35(14):1718–1722, 2020. ISSN 15327027. . URL <https://doi.org/10.1080/10410236.2020.1838096>.

Martin G. Myers and Diego Pineda. Misinformation about Vaccines. In Alan D.T. Barrett and Lawrence R. Stanberry, editors, *Vaccines for Biodefense and Emerging and Neglected Diseases*, chapter 17, pages 255–270. Academic Press, London, 2009. ISBN 978-0-12-369408-9. .

David A. Broniatowski, Amelia M. Jamison, Si Hua Qi, Lulwah AlKulaib, Tao Chen, Adrian Benton, Sandra C. Quinn, and Mark Dredze. Weaponized health communication: Twitter bots and Russian trolls amplify the vaccine debate. *American* *Journal of Public Health*, 108(10):1378–1384, 2018. ISSN 15410048. .

Wen Ying Sylvia Chou, Anna Gaysynsky, and Joseph N. Cappella. Where we go from here: Health misinformation on social media. *American Journal of Public Health*, 110(S3):S273–S275, 2020. ISSN 15410048. .

Matthew J. Hornsey, Emily A. Harris, and Kelly S. Fielding. The psychological roots of anti-vaccination attitudes: A 24-nation investigation. *Health Psychology*, 37(4):307–315, 2018. ISSN 19307810. .

3.

4.

5.

6.

7.

8.

9.

10.

11.

12.

13.

14.

15.

16.

17.

18.

19.

20.

21.

22.

23.

**75 of** [**75**](#_bookmark21)
