# Supplementary material for: Psychological inoculation improves resilience to and reduces willingness to share vaccine misinformation
Source: Sci Rep. 2025 Aug 18;15:29830. doi: 10.1038/s41598-025-09462-5 (PMC12358572; doi:10.1038/s41598-025-09462-5)
Supplement: Supplementary file 2 — Supplementary Information 2. [file 41598_2025_9462_MOESM2_ESM.pdf]

Supplementary Information for

Psychological inoculation improves resilience to and reduces willingness to share vaccine misinformation

Ruth E. Appel, Jon Roozenbeek, Rebecca Rayburn-Reeves, Melisa Basol, Jonathan Corbin, Josh Compton and Sander van der Linden

Jon Roozenbeek.
E-mail: jjr51@cam.ac.uk

This PDF file includes:

Supplementary text
Figs. S1 to S25
Tables S1 to S75
SI References

Contents

Deviations and Clarifications **3**

Extended Data Tables and Figures **3**
Overview of ANOVA Results for Studies 1 to 3 . . . 3
Figures and Tables for Overview of ANOVA Results for Studies 1 to 3 . . . 3
Disentangling Identification of Truthfulness, Manipulativeness, and Manipulation Technique . . . 16
Correct Identification . . . 16
Manipulativeness, Truthfulness, and Technique Recognition by Post Type . . . 18
Internal Meta-Analyses . . . 21
Overall Network Meta-Analysis Effects by Treatment and by Outcome . . . 21
Pairwise Meta-Analyses by Outcome . . . 22
Manipulativeness . . . 22
Confidence . . . 22
Sharing Intent . . . 22

Interaction Between Condition and Covariates **31**
COVID-19 Vaccine Intentions . . . 31
Political Ideology . . . 36

Inoculation Theory **41**

Identifying Vaccine Misinformation Techniques **41**

Descriptive Statistics **41**
Number of Observations . . . 41
Demographics . . . 41
Group Means for Outcome Variables . . . 46

Balance Tables **48**

Detailed Results **51**
Study 1 . . . 51
Study 2 . . . 58
Study 3 . . . 66

Supplementary Information Text

Deviations and Clarifications

Clarification #1: In the preregistration for Study 1, we wrote "Participants who do not successfully complete a "fake news" game will be excluded; we will check this by including a password at the end of the game that participants have to provide. Participants who fail an attention check will be excluded." Only participants who played the Bad Vaxx game received a password, so only treatment group participants were excluded based on an incorrect password.

Clarification #2: For Studies 1, 2 and 3, we considered the game attention check password as correct when it was either an exact match for the password "INOC1983", or a close variant (e.g., "IN0C1983" or "INO").

Clarification #3: In Studies 1, 2 and 3, we excluded preview responses from the research team, as well as responses from non-consenting participants, participants who did not finish the survey, and participants who had empty Prolific IDs (the latter only occurred in Study 3).

Clarification #4: In the preregistration for Study 2, we wrote "Participants who fail two attention checks will be excluded and resampled." Consistent with Study 1 and 3, we also excluded participants who failed one attention check, but these participants were compensated in the same way as participants who completed the study.

Clarification #4: In the preregistration for Study 2, we had not specified what we would do with participants with missing values on the main outcome variables like Manipulativeness Discernment. While many regression functions drop these rows automatically, the function we use did not, so we excluded two observations where Manipulativeness Discernment could not be calculated.

Clarification #5: To assess confidence in manipulativeness ratings, we focus on manipulative posts. We mention in the preregistration for Study 3 that we will look at confidence in manipulativeness ratings for both manipulativeness and non-manipulative posts separately, and we present results of both analyses. However, the main outcome of interest is confidence in the manipulativeness ratings of manipulative posts because a positive effect indicates higher confidence and a negative effect lower confidence. A discernment measure that combines manipulative and non-manipulative posts is not clear to interpret since a participant could, for example, have both higher confidence in correctly assessing the manipulativeness of a non-manipulative post as low, and higher confidence in assessing the manipulativeness of a manipulative post as high, resulting in no change in the difference although the confidence for ratings of each post category increased. We did not have prior expectations on how confidence in manipulativeness ratings of non-manipulative information would change, so our hypothesis on confidence in manipulativeness ratings focuses only on manipulative posts.

Clarification #6: We ran additional analyses that were not preregisted, such as testing an interaction between COVID-19 vaccination intentions and condition, or political ideology and condition with regards to our main outcome measures. We preregistered that we would analyze covariates like vaccination intentions, but not that we would include them in an interaction model.

Clarification #7: In the preregistration, we used the terms "vaccine" and "vaccination" interchangeably. Our intervention targets misinformation related both to the process of introducing a vaccine into the body (vaccination) and vaccines more broadly, therefore we opted to use the term "vaccine" consistently in the paper and supplement.

Deviation #1: We preregistered that we would conduct linear regressions at the rating level, clustered on study participants and misinformation vs. matched control outcome measures. Departing from our preregistration, we instead ran multi-level models with participants and items modelled as random effects, and a series of relevant covariates. While both types of analyses have the same aim to account for participant- and item-level variation, we decided to use multi-level modeling since the participant- and item-level variation is more appropriately taken into account in this type of model.

Extended Data Tables and Figures

Overview of ANOVA Results for Studies 1 to 3.

Figures for Overview of ANOVA Results for Studies 1 to 3.

Manipulativeness.

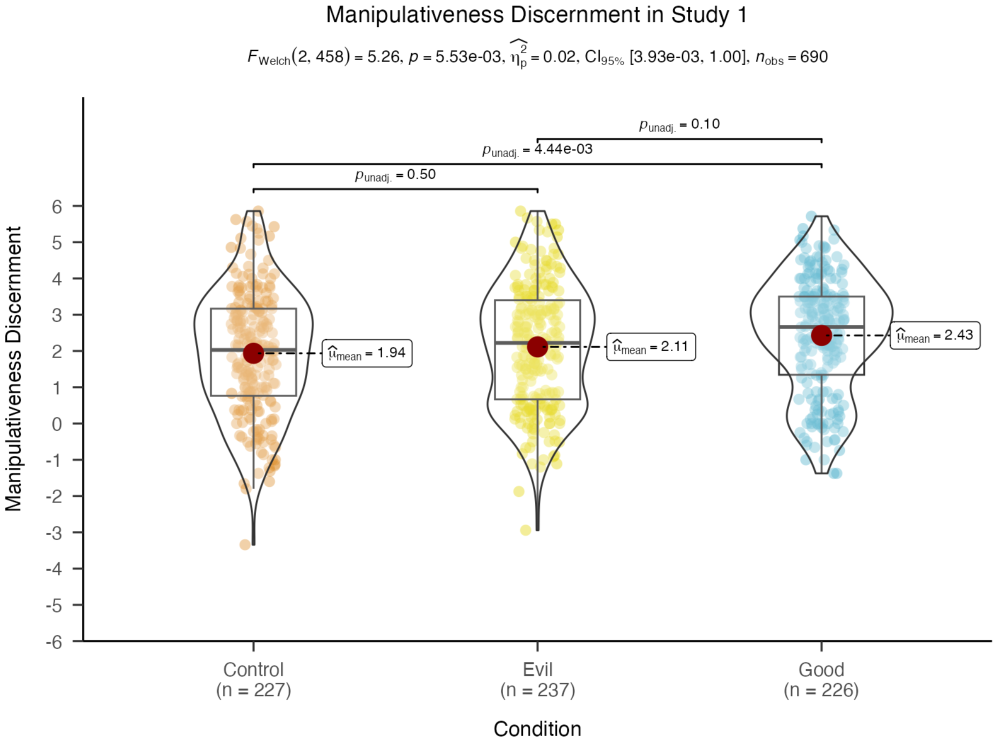

Fig. S1. Study 1: Overall and pairwise comparison of the effect of the *Bad Vaxx* game on manipulativeness discernment.

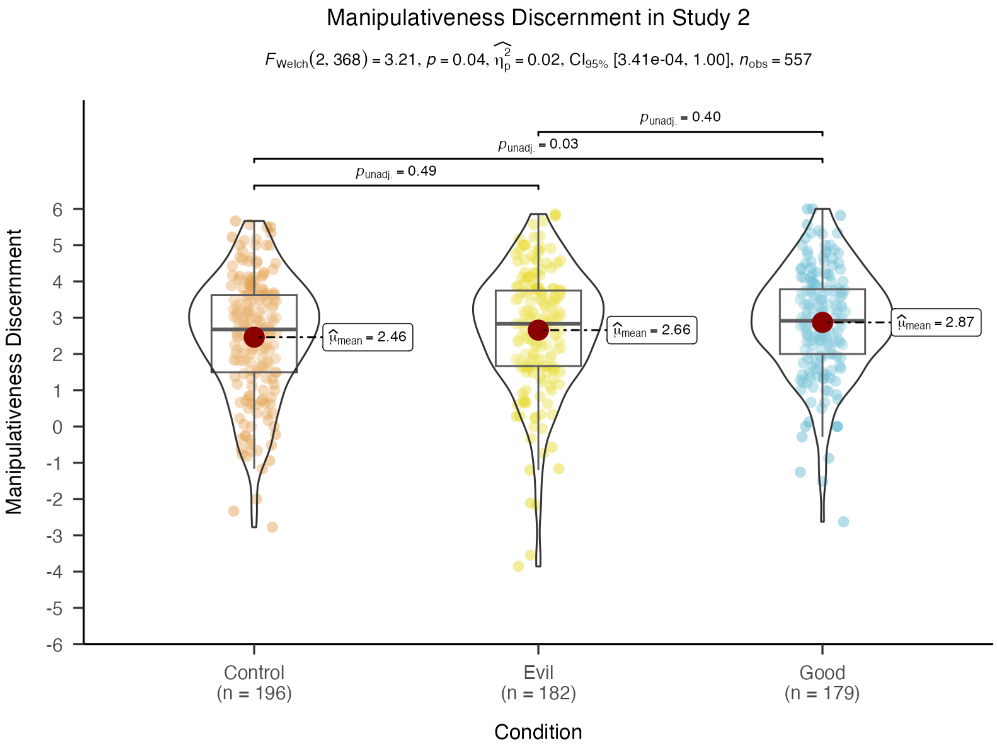

Fig. S2. Study 2: Overall and pairwise comparison of the effect of the *Bad Vaxx* game on manipulativeness discernment.

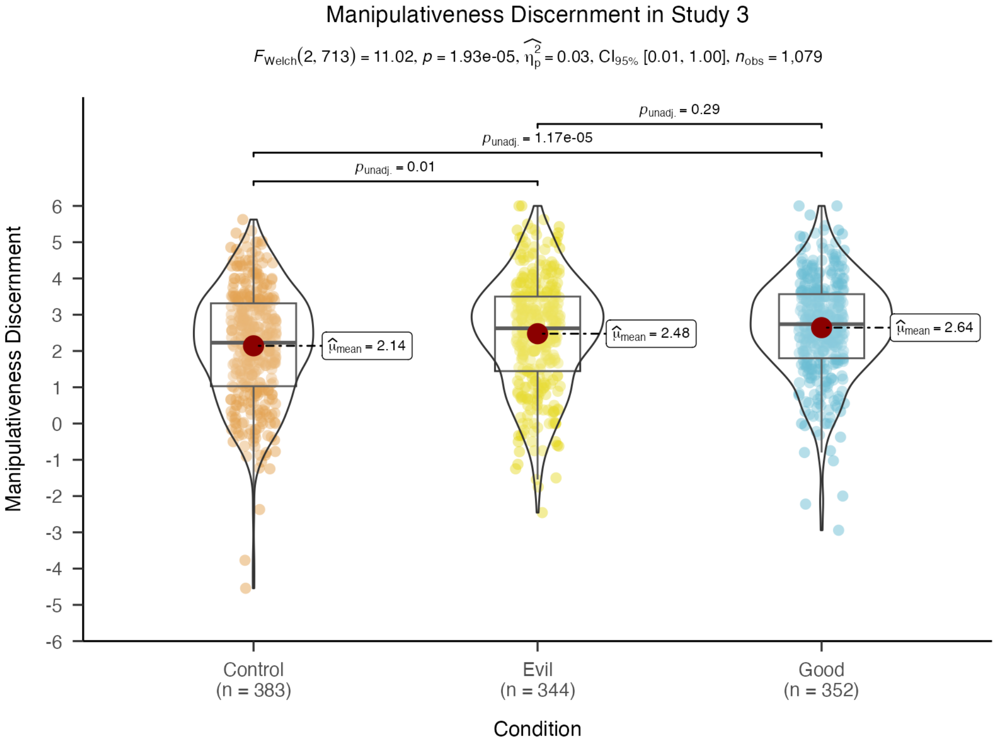

Fig. S3. Study 3: Overall and pairwise comparison of the effect of the *Bad Vaxx* game on manipulativeness discernment.

Confidence.

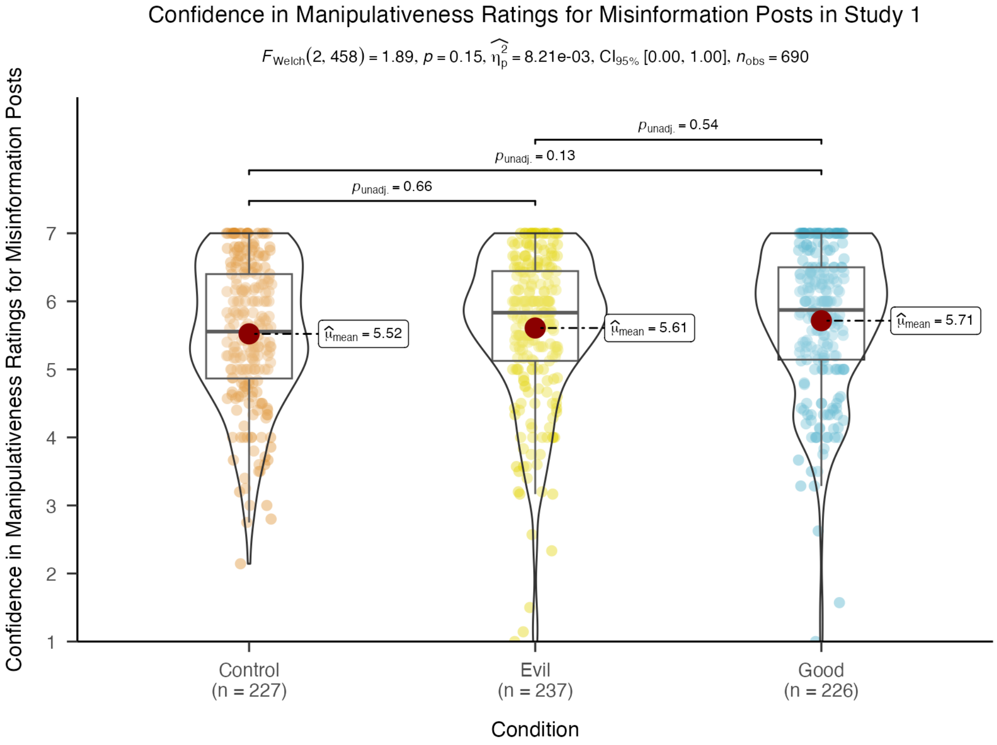

Fig. S4. Study 1: Overall and pairwise comparison of the effect of the *Bad Vaxx* game on confidence in manipulativeness ratings for misinformation posts.

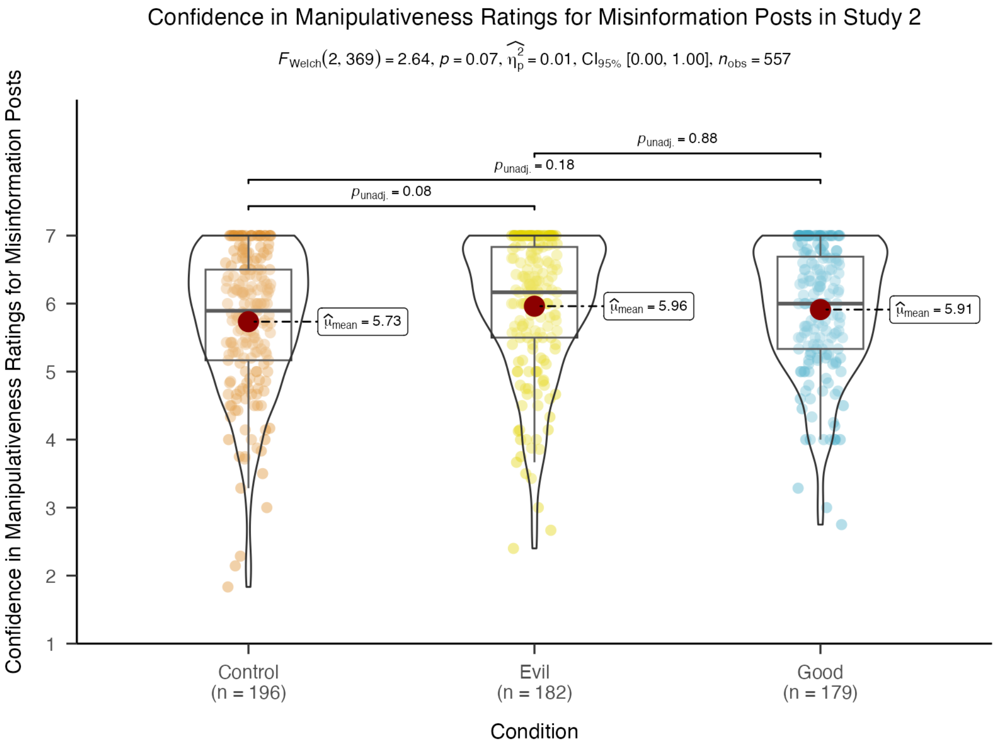

Fig. S5. Study 2: Overall and pairwise comparison of the effect of the *Bad Vaxx* game on confidence in manipulativeness ratings for misinformation posts.

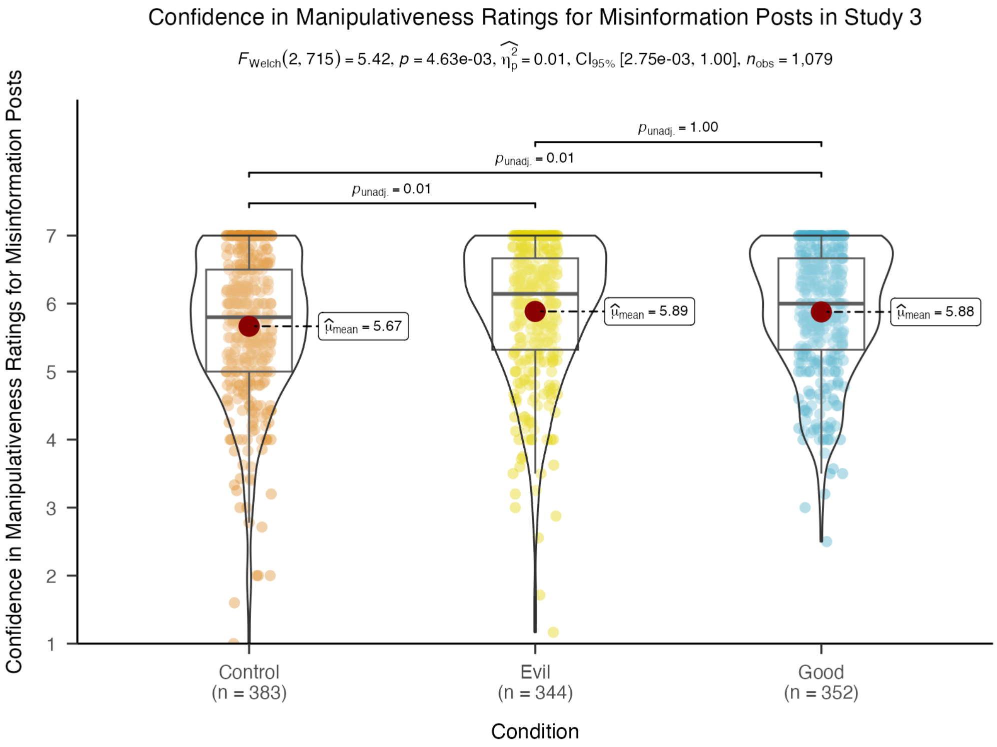

Fig. S6. Study 3: Overall and pairwise comparison of the effect of the *Bad Vaxx* game on confidence in manipulativeness ratings for misinformation posts.

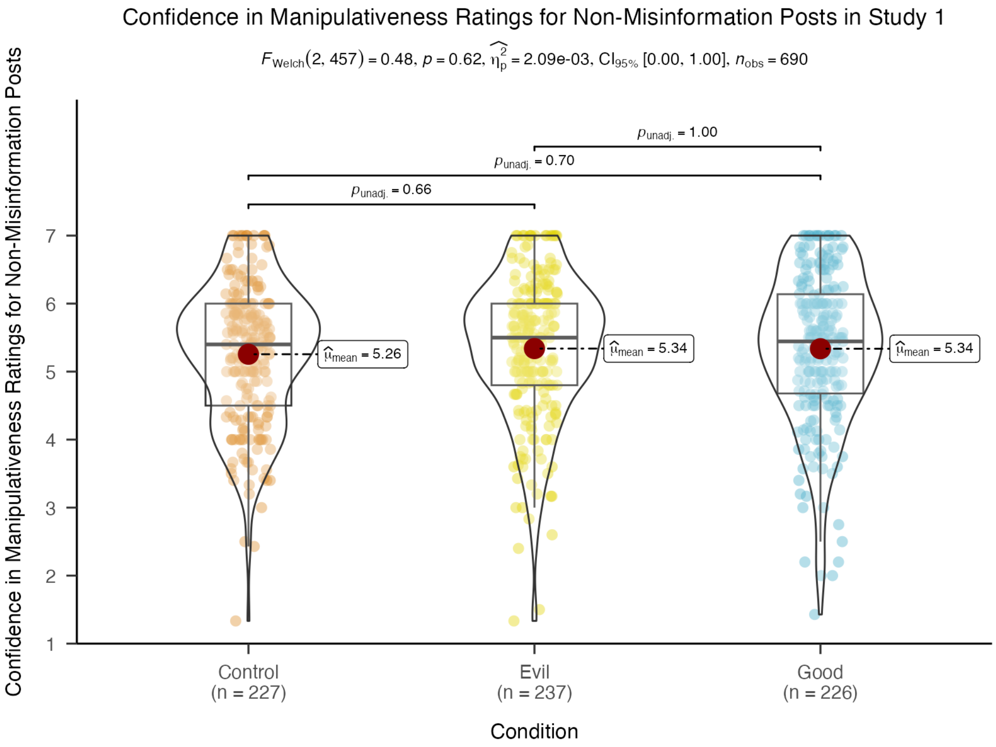

Fig. S7. Study 1: Overall and pairwise comparison of the effect of the *Bad Vaxx* game on confidence in manipulativeness ratings for non-misinformation posts.

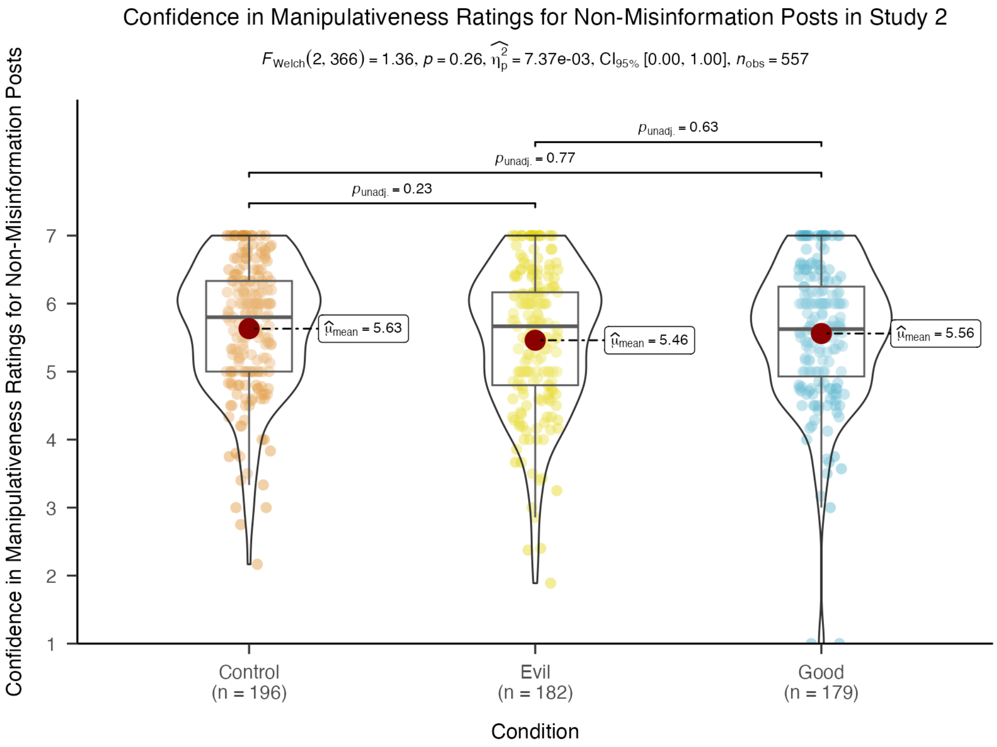

Fig. S8. Study 2: Overall and pairwise comparison of the effect of the *Bad Vaxx* game on confidence in manipulativeness ratings for non-misinformation posts.

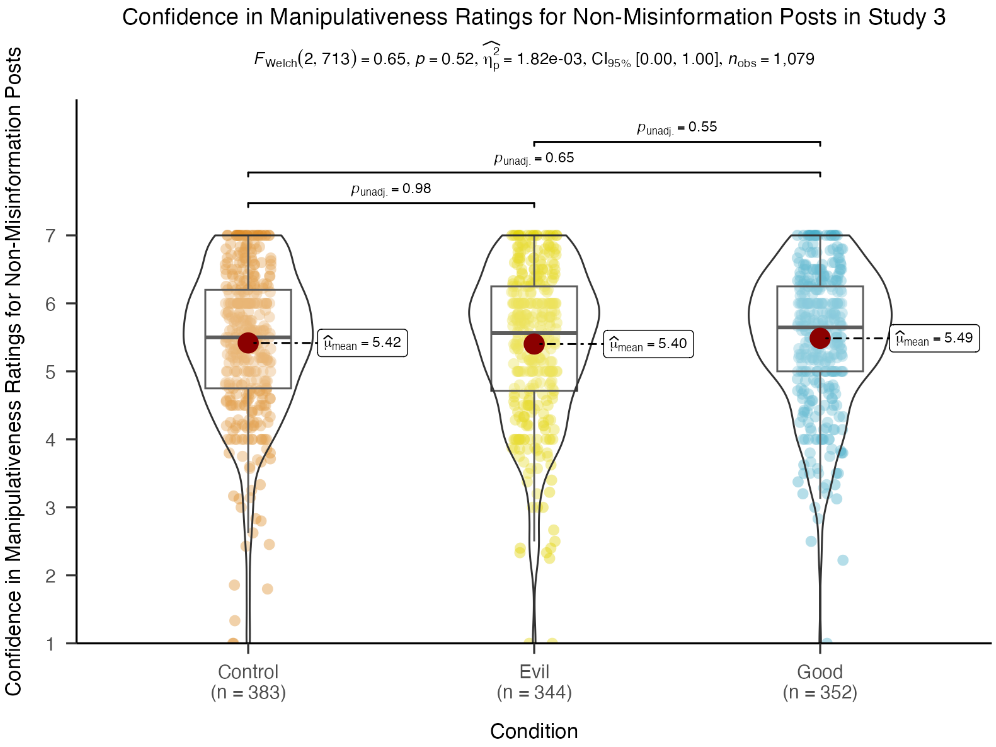

Fig. S9. Study 3: Overall and pairwise comparison of the effect of the *Bad Vaxx* game on confidence in manipulativeness ratings for non-misinformation posts.

Sharing Intent.

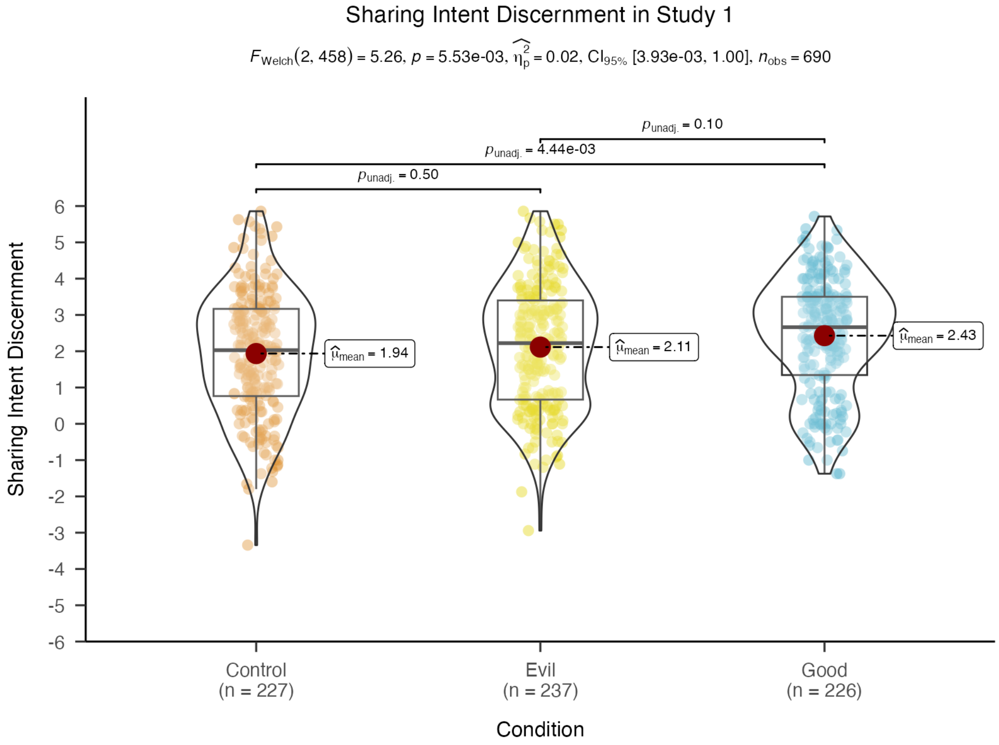

Fig. S10. Study 1: Overall and pairwise comparison of the effect of the *Bad Vaxx* game on sharing intent discernment.

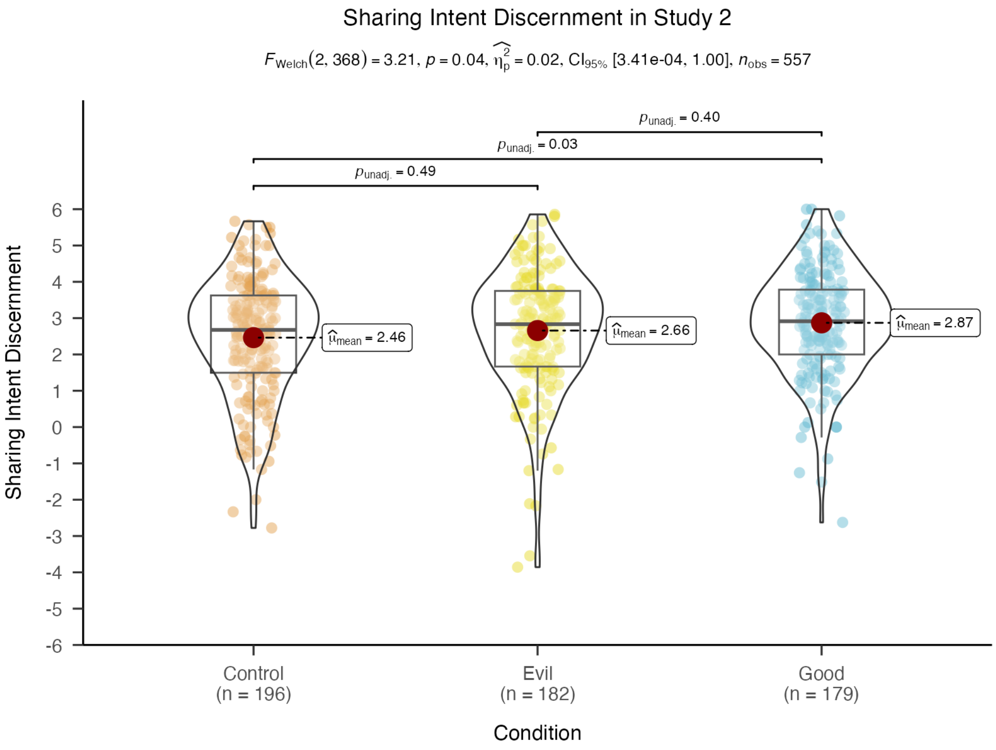

Fig. S11. Study 2: Overall and pairwise comparison of the effect of the *Bad Vaxx* game on sharing intent discernment.

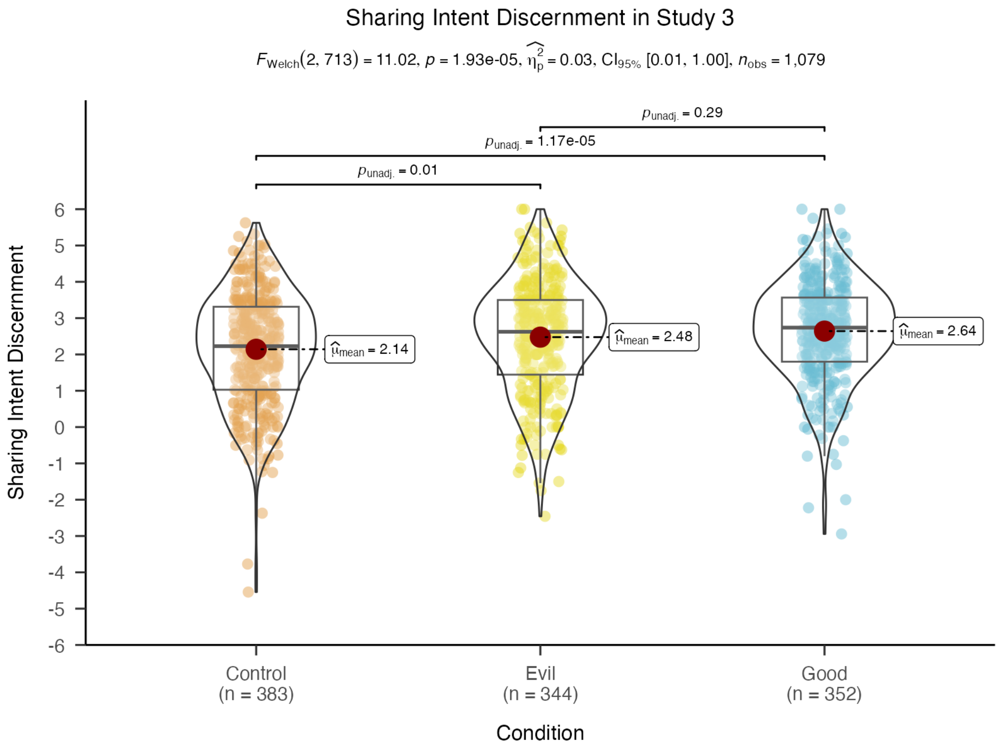

Fig. S12. Study 3: Overall and pairwise comparison of the effect of the *Bad Vaxx* game on sharing intent discernment.

Disentangling Identification of Truthfulness, Manipulativeness, and Manipulation Technique.

Correct Identification. This section contains the results of a preregistered exploratory analysis aiming to disentangle whether the *Bad Vaxx* game affects the identification of true vs. false information, manipulative vs. non-manipulative information, or the recognition of misinformation techniques.

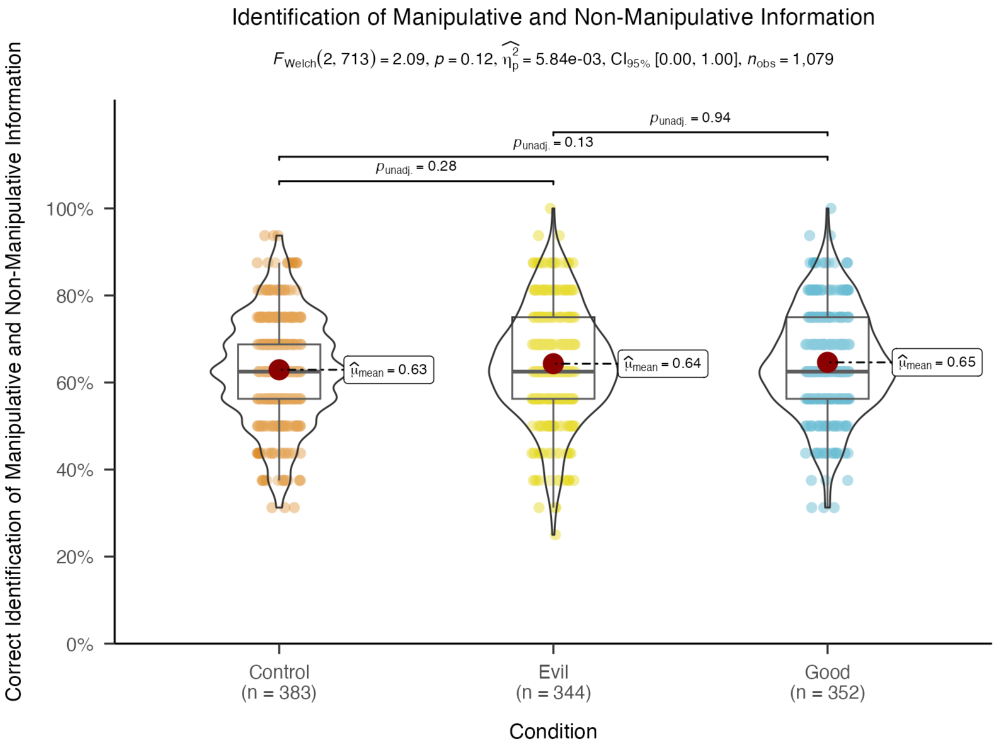

Fig. S13. Study 3: Overall and pairwise comparison of the effect of the *Bad Vaxx* game on the correct identification of manipulative vs. non-manipulative information.

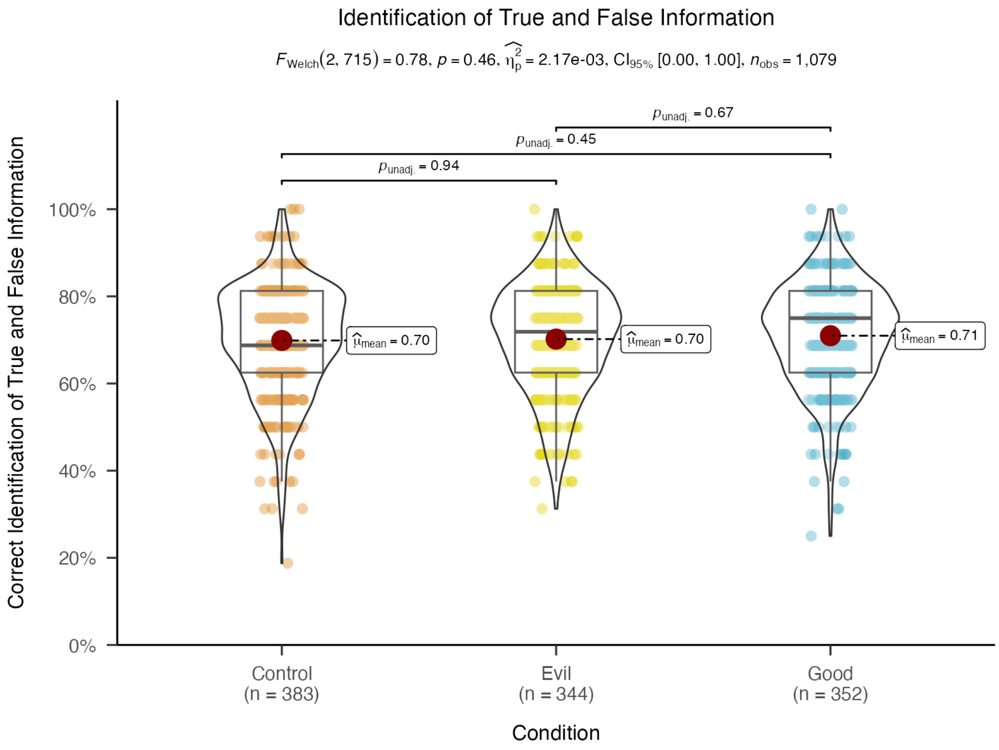

Fig. S14. Study 3: Overall and pairwise comparison of the effect of the *Bad Vaxx* game on the correct identification of true vs. false information.

Manipulativeness, Truthfulness, and Technique Recognition by Post Type. Going beyond the preregistered exploratory analysis aiming to disentangle whether the *Bad Vaxx* game affects the identification of true vs. false information, manipulative vs. non-manipulative information, or the recognition of misinformation techniques, we provide the results for effects on identification of true vs. false, manipulative vs. non manipulative, and technique recognition by post type along the dimensions manipulativeness (manipulative or non-manipulative) and truthfulness (true or false).

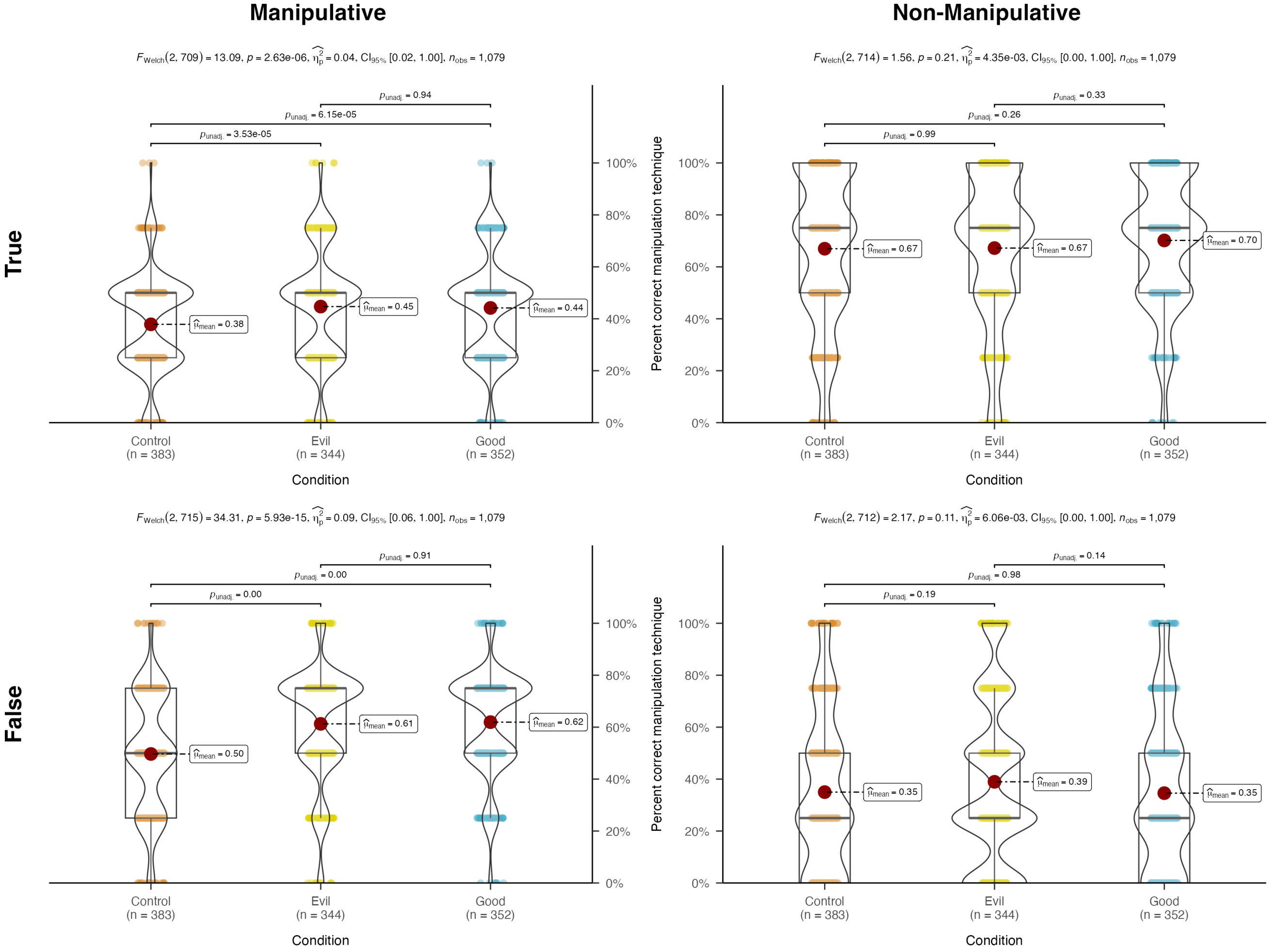

Fig. S15. Study 3: Overall and pairwise comparison of the effect of the *Bad Vaxx* game on correct technique recognition by post characteristics (manipulative vs. non-manipulative, true vs. false).

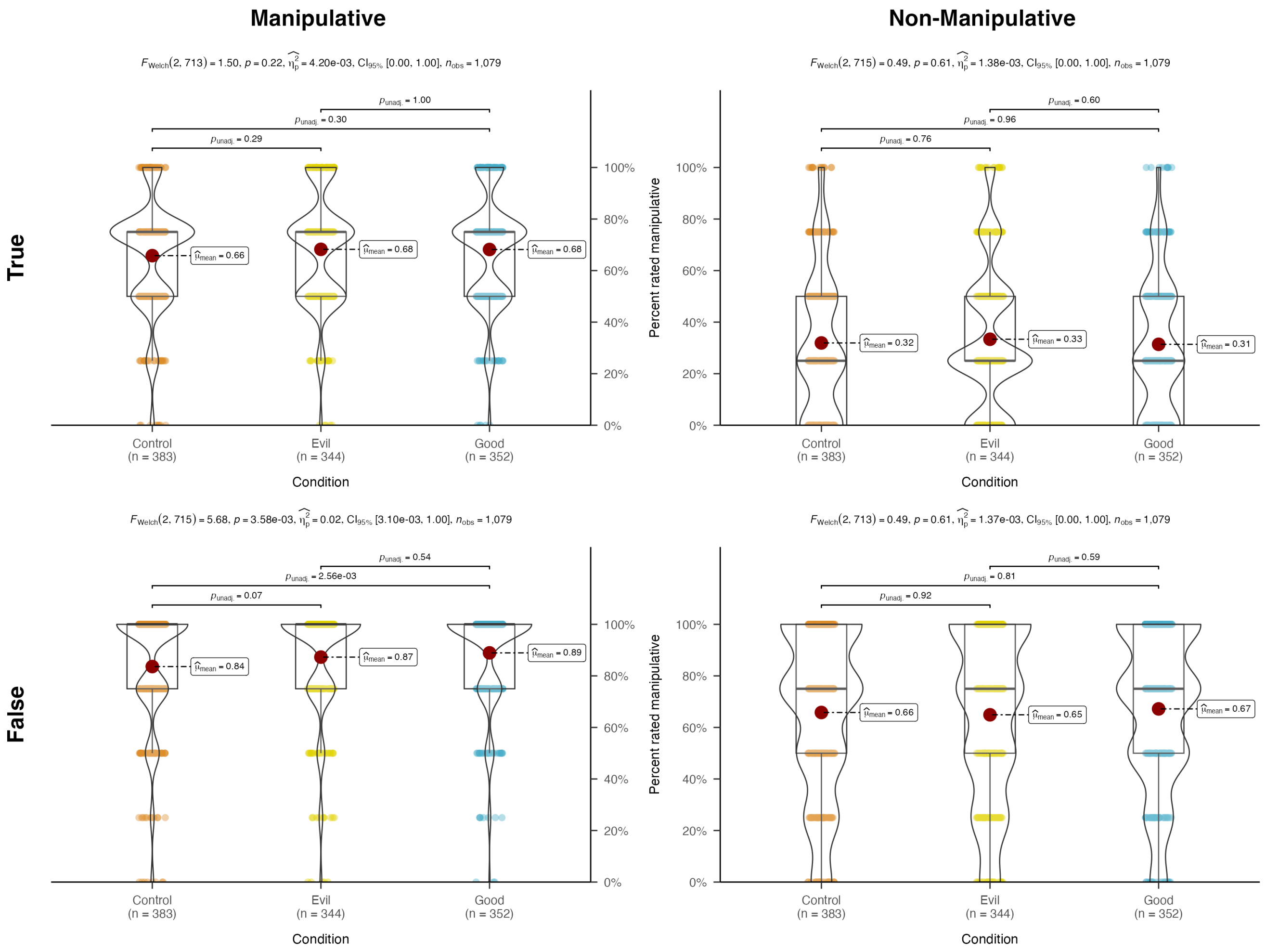

Fig. S16. Study 3: Overall and pairwise comparison of the effect of the *Bad Vaxx* game on the rating of social media posts as manipulative by post characteristics (manipulative vs. non-manipulative, true vs. false).

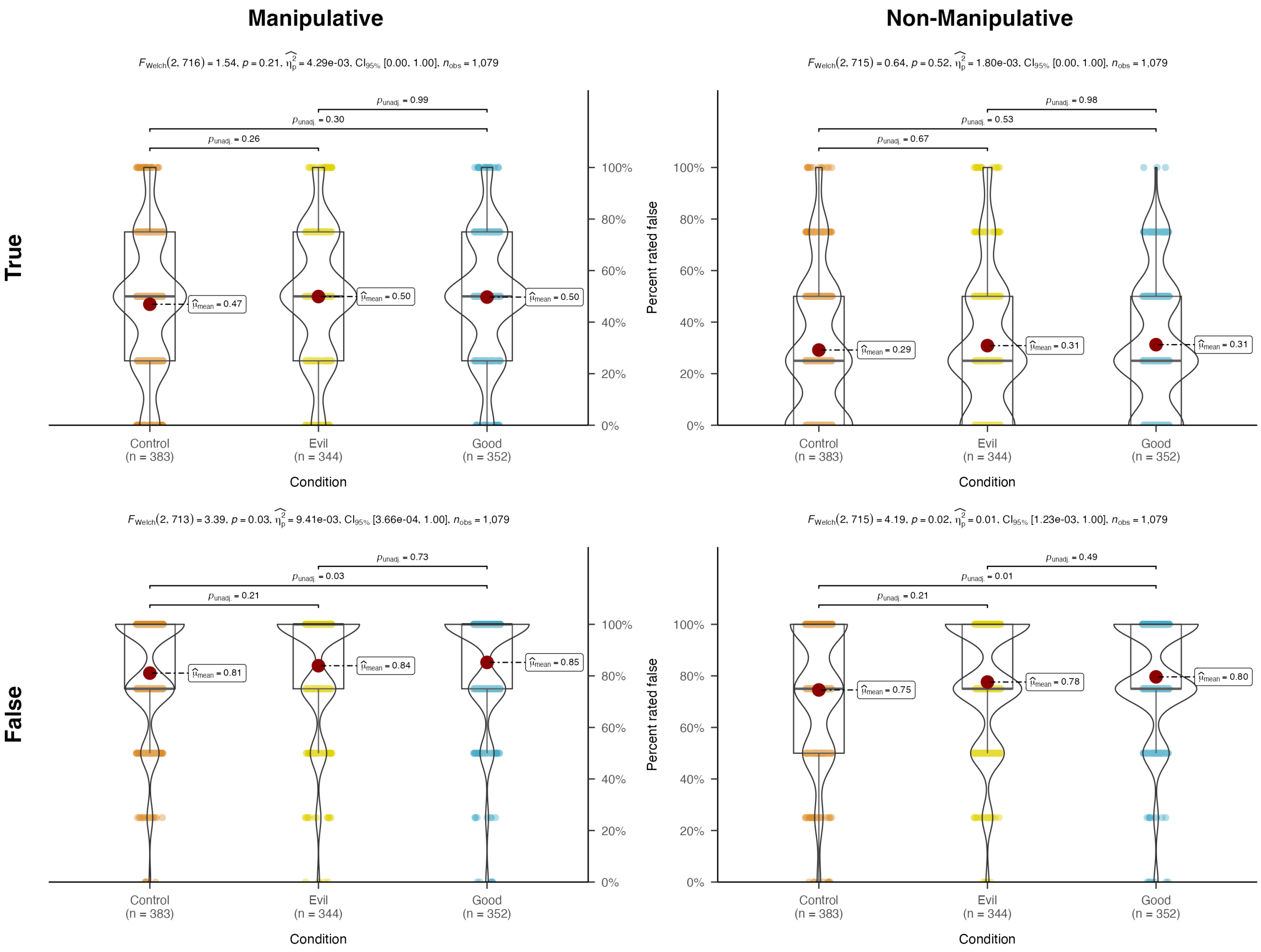

Fig. S17. Study 3: Overall and pairwise comparison of the effect of the *Bad Vaxx* game on the rating of social media posts as false by post characteristics (manipulative vs. non-manipulative, true vs. false).

Internal Meta-Analyses.

Overall Network Meta-Analysis Effects by Treatment and by Outcome.

Table S1. Overall Effects by Treatment and by Outcome

Variable	Treatment	Cohen's d	Cohen's d SE	CI lower	CI upper	p-value
Misinformation Manipulativeness	Good Version	0.33	0.05	0.23	0.43	**<0.001**
Non-Misinformation Manipulativeness	Good Version	-0.10	0.05	-0.20	0.00	0.052
Manipulativeness Discernment	Good Version	0.31	0.05	0.21	0.41	**<0.001**
Misinformation Confidence	Good Version	0.19	0.05	0.10	0.29	**<0.001**
Non-Misinformation Confidence	Good Version	0.03	0.05	-0.06	0.13	0.492
Misinformation Sharing Intent	Good Version	-0.13	0.05	-0.23	-0.03	**0.012**
Non-Misinformation Sharing Intent	Good Version	0.21	0.05	0.11	0.31	**<0.001**
Sharing Intent Discernment	Good Version	0.34	0.05	0.24	0.44	**<0.001**
Misinformation Manipulativeness	Evil Version	0.29	0.05	0.19	0.39	**<0.001**
Non-Misinformation Manipulativeness	Evil Version	0.07	0.05	-0.03	0.17	0.160
Manipulativeness Discernment	Evil Version	0.16	0.05	0.06	0.26	**0.001**
Misinformation Confidence	Evil Version	0.18	0.05	0.08	0.28	**<0.001**
Non-Misinformation Confidence	Evil Version	-0.02	0.05	-0.12	0.08	0.641
Misinformation Sharing Intent	Evil Version	-0.01	0.05	-0.11	0.09	0.852
Non-Misinformation Sharing Intent	Evil Version	0.09	0.05	-0.01	0.19	0.073
Sharing Intent Discernment	Evil Version	0.11	0.05	0.01	0.20	**0.037**

Note. Results are based on a network meta-analysis using fixed effects for Studies 1, 2 and 3. By default standard errors for this type of analysis are adjusted for the correlation between the different comparisons in multi-arm studies.

Pairwise Meta-Analyses by Outcome.

Below, we provide the full set of forest plots for the meta-analyses, one for each outcome. The plots below show the pairwise meta-analyses results across Studies 1 to 3 by treatment and outcome variable. We used the `meta` (1) and `netmeta` (2) packages to conduct the analyses and produce the plots, and by default standard errors for the pairwise analyses are unadjusted.

Manipulativeness. For the misinformation items, pairwise meta-analyses show that both the "good" (Cohen's $d = 0.33, SE = 0.05, p < 0.001$) and the "evil" version of the game (Cohen's $d = 0.29, SE = 0.05, p < 0.001$) have a significant effect across Studies 1 to 3 compared to the control group. The pairwise comparison between the "good" and the "evil" version is nonsignificant. This supports **H1a**: The *Bad Vaxx* game increases players' ability to identify manipulative social media content about vaccines.

With respect to non-misinformation, the meta-analyses show that the effect of both the "good" and the "evil" version of the Bad Vaxx game is nonsignificant compared to the control group. Participants playing the "evil" version rate non-misinformation as significantly more manipulative compared to participants playing the "good" version (Cohen's $d = 0.17, SE = 0.05, p < 0.001$).

For discernment, that is, the difference between manipulativeness ratings for manipulative and non-manipulative content (see Figure **??**), the meta-analyses show that both the "good" (Cohen's $d = 0.31, SE = 0.05, p < 0.001$) and the "evil" version (Cohen's $d = 0.16, SE = 0.05, p = 0.002$) have significantly higher discernment than the control group. Further, participants playing the "evil" version have significantly lower discernment than those playing the "good" version (Cohen's $d = -0.14, SE = 0.05, p = 0.006$). This supports **H1b**: Playing the *Bad Vaxx* game increases players' ability to discern manipulative from non-manipulative vaccine information.

Confidence. Pairwise meta-analyses show that both the "good" (Cohen's $d = 0.2, SE = 0.05, p < 0.001$) and the "evil" version of the game (Cohen's $d = 0.18, SE = 0.05, p < 0.001$) have a significant and positive effect on confidence in manipulativeness ratings for vaccine misinformation across Studies 1 to 3 compared to the control group. The pairwise comparison between the "good" and the "evil" version is nonsignificant. Confidence in manipulativeness ratings for non-misinformation does not differ significantly between treatment and control groups. These results support **H2**: Participants playing the *Bad Vaxx* game have greater confidence in their assessment of the manipulativeness of vaccine misinformation.

Sharing Intent. For the misinformation items, pairwise meta-analyses show that the "good" version of the *Bad Vaxx* game has a significant negative effect on willingness to share compared to the control condition (Cohen's $d = -0.13, SE = 0.05, p = 0.012$), but the effect of the "evil" version compared to the control condition is nonsignificant. Further, participants playing the "evil" version are significantly more willing to share misinformation than those playing the "good" version (Cohen's $d = 0.12, SE = 0.05, p = 0.022$). This partially supports hypothesis **H3a**, namely that the game reduces willingness to share vaccine misinformation, notably only for the "good" version of the game.

With respect to non-misinformation, the meta-analyses show that participants playing the "good" version are significantly more willing to share non-misinformation than those in the control condition (Cohen's $d = 0.21, SE = 0.05, p < 0.001$), while there is no effect for participants playing the "evil" version compared to the control condition. Participants playing the "evil" version have significantly lower willingness to share non-misinformation compared to those playing the "good" version (Cohen's $d = -0.12, SE = 0.05, p = 0.018$).

In terms of discernment, that is, the difference between intent to share non-misinformation and intent to share misinformation (see Figure **??**), the meta-analyses show that the "good" version (Cohen's $d = 0.34, SE = 0.05, p < 0.001$) and the "evil" version (Cohen's $d = 0.1, SE = 0.05, p = 0.043$) of the game significantly increase discernment in willingness to share compared to the control condition. Further, participants playing the "evil" version have significantly lower discernment in willingness to share compared to those playing the "good" version (Cohen's $d = -0.23, SE = 0.05, p < 0.001$). This supports hypothesis **H3b**: Playing the *Bad Vaxx* game increases the quality of people's sharing decisions, in the sense that they have a higher difference in willingness to share non-manipulative versus manipulative information about vaccines.

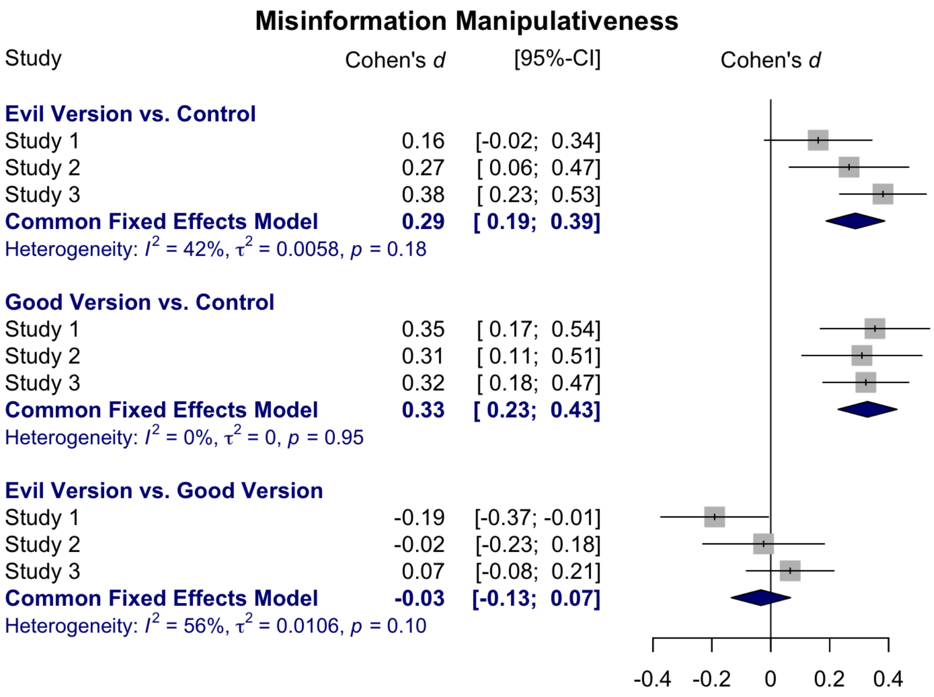

Fig. S18. Forest plot for a pairwise meta-analysis of the Misinformation Manipulativeness measure across Studies 1 to 3.

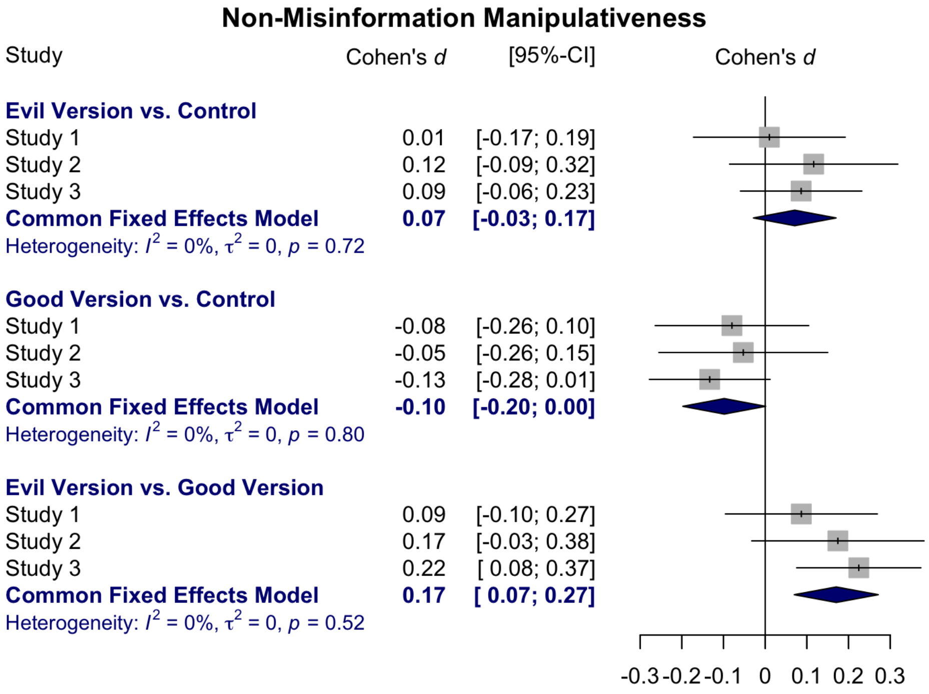

Fig. S19. Forest plot for a pairwise meta-analysis of the Non-Misinformation Manipulativeness measure across Studies 1 to 3.

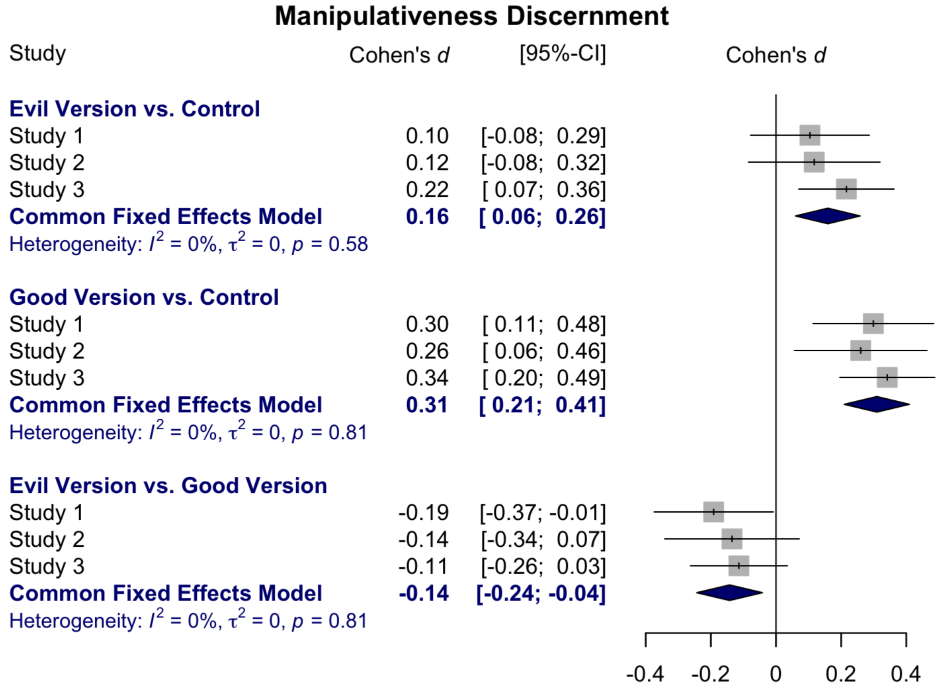

Fig. S20. Forest plot for a pairwise meta-analysis of the Manipulativeness Discernment measure across Studies 1 to 3.

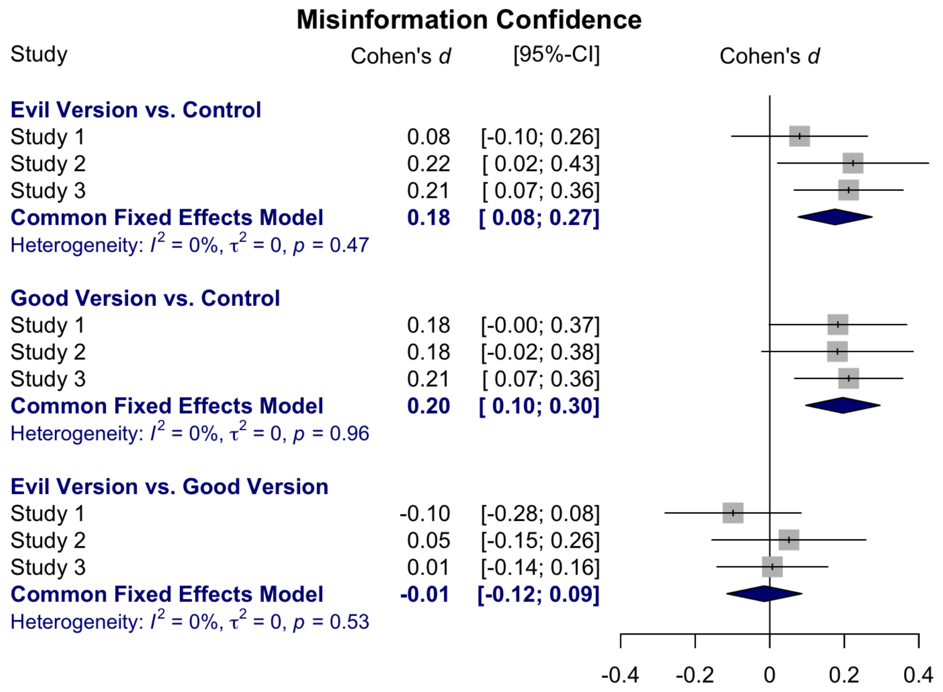

Fig. S21. Forest plot for a pairwise meta-analysis of the Misinformation Confidence measure across Studies 1 to 3.

Non-Misinformation Confidence

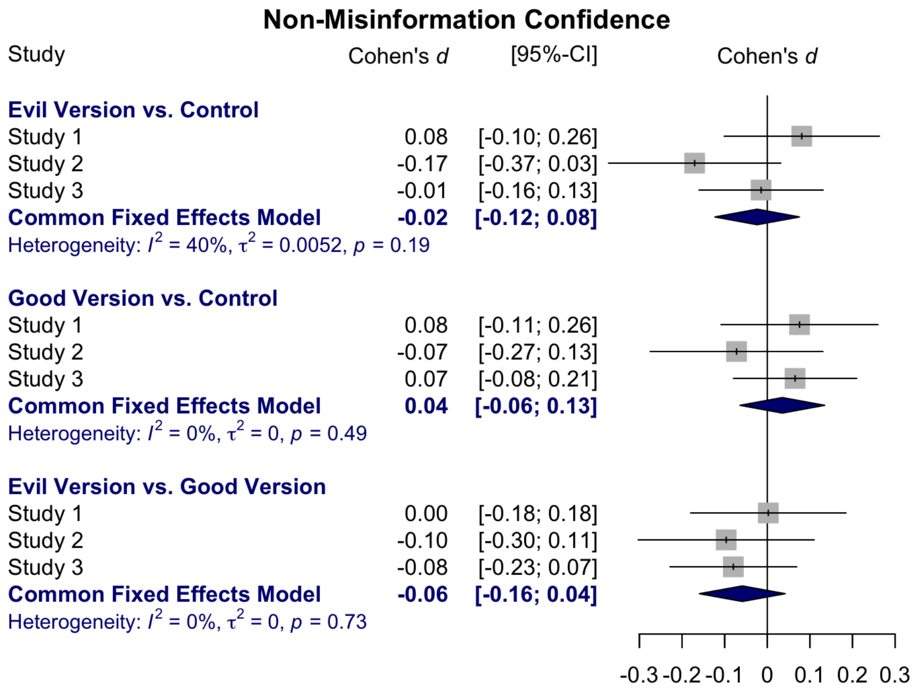

Fig. S22. Forest plot for a pairwise meta-analysis of the Non-Misinformation Confidence measure across Studies 1 to 3.

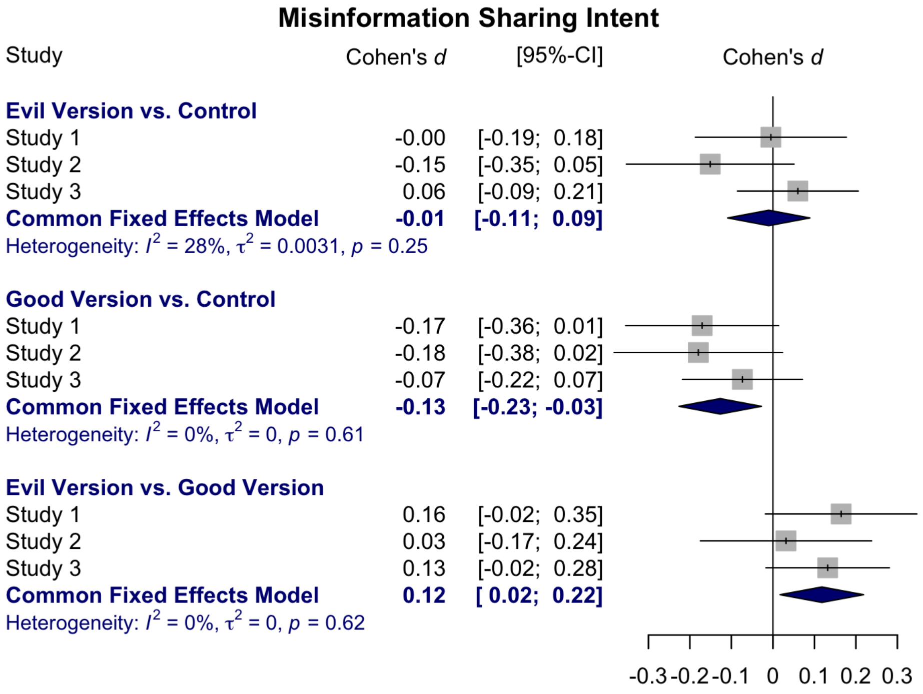

Fig. S23. Forest plot for a pairwise meta-analysis of Misinformation Sharing measure across Studies 1 to 3.

Non-Misinformation Sharing

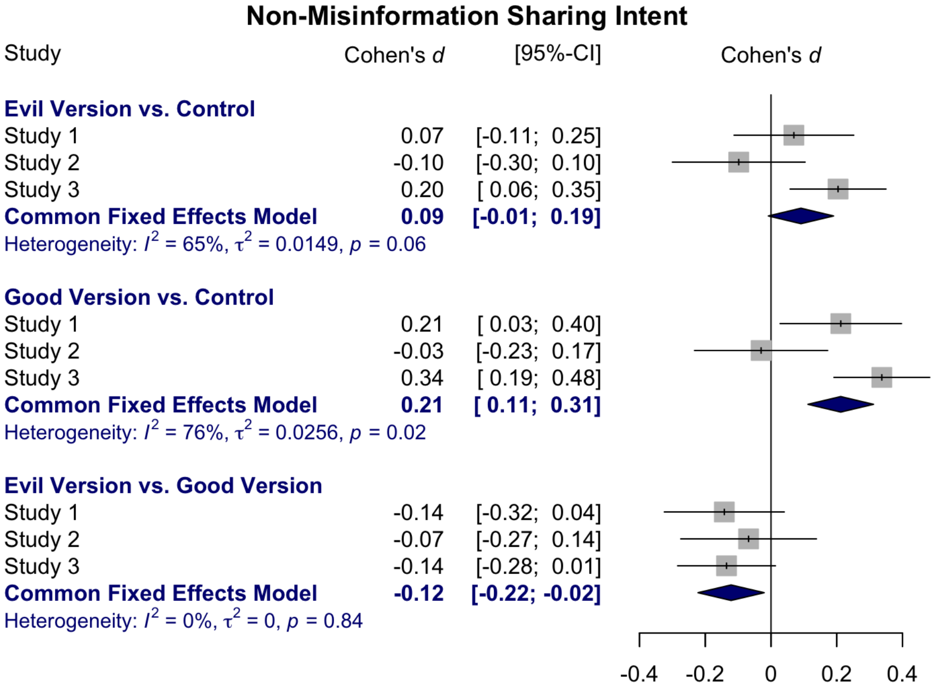

Fig. S24. Forest plot for a pairwise meta-analysis of the Non-Misinformation Sharing measure across Studies 1 to 3.

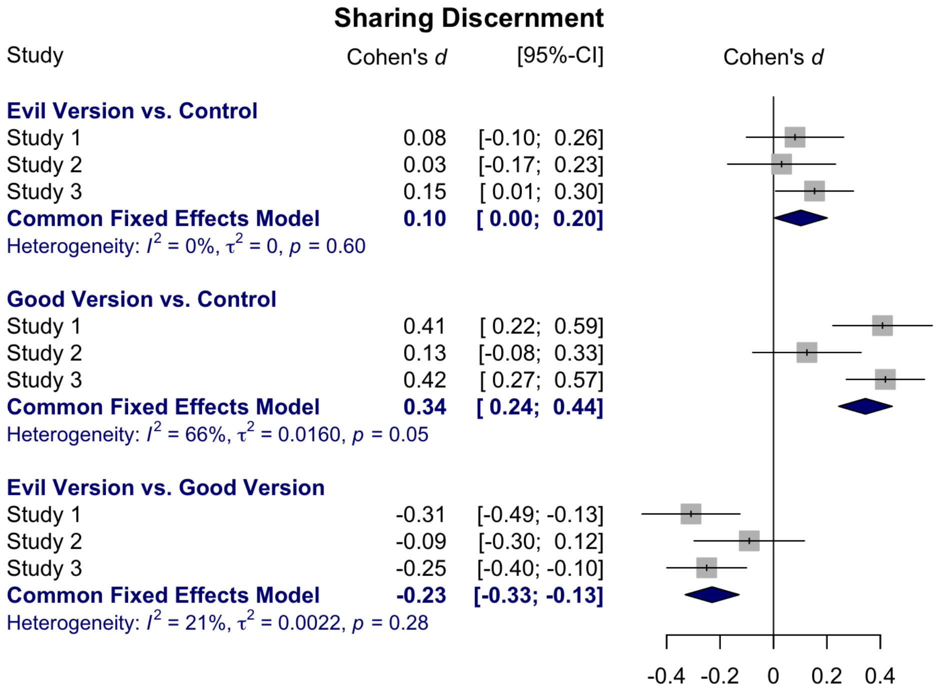

Fig. S25. Forest plot for a pairwise meta-analysis of the Sharing Discernment measure across Studies 1 to 3.

Interaction Between Condition and Covariates

This section contains the results for non-preregistered analyses on the interaction between condition and the covariates COVID-19 vaccine intentions and political ideology.

COVID-19 Vaccine Intentions.

Table S2. Linear Regression Model for Manipulativeness Ratings (Fake Items Only) Including Interaction Between Condition and COVID-Vaccine Intentions

	Study 1	Study 2	Study 3
(Intercept)	**5.15*****	**4.84*****	**3.58*****
	(0.31)	(0.37)	(0.24)
COVID.Vaccine.Intentions	**0.53****	**0.92*****	**1.03*****
	(0.17)	(0.20)	(0.15)
ConditionEvil	−0.00	0.34	**0.76*****
	(0.19)	(0.24)	(0.18)
ConditionGood	**0.52***	0.42	**0.46***
	(0.21)	(0.25)	(0.19)
Having.Children	**−0.33*****	**−0.32****	−0.06
	(0.10)	(0.10)	(0.07)
Gender_simpleMale	−0.10	**−0.27****	0.07
	(0.08)	(0.09)	(0.06)
Gender_simpleOther	0.21	0.23	−0.01
	(0.30)	(0.28)	(0.21)
Political.Ideology.AllConservative	−0.01	0.02	0.10
	(0.13)	(0.15)	(0.10)
Political.Ideology.AllLiberal	**0.22***	0.20	**0.48*****
	(0.11)	(0.13)	(0.08)
Age	0.05	0.08	0.05
	(0.04)	(0.04)	(0.03)
Education	−0.00	−0.03	−0.02
	(0.05)	(0.04)	(0.02)
News.Checking	0.06	0.03	**0.14*****
	(0.05)	(0.06)	(0.04)
Social.Media.Use	**−0.13****	−0.05	0.01
	(0.04)	(0.06)	(0.03)
COVID.Vaccine.Intentions:ConditionEvil	0.30	0.02	−0.39
	(0.22)	(0.27)	(0.20)
COVID.Vaccine.Intentions:ConditionGood	−0.13	−0.12	−0.16
	(0.24)	(0.27)	(0.21)
R^2	0.14	0.20	0.21
Adj. R^2	0.13	0.17	0.19
Num. obs.	689	555	1075

$^{***}p < 0.001$; $^{**}p < 0.01$; $^{*}p < 0.05$

Table S3. Linear Regression Model for Manipulativeness Ratings (Difference Between Fake and Real) Including Interaction Between Condition and COVID-Vaccine Intentions

	Study 1	Study 2	Study 3
(Intercept)	**2.63*****	**1.19***	−0.06
	(0.43)	(0.51)	(0.33)
COVID.Vaccine.Intentions	**0.54***	**0.96*****	**1.26*****
	(0.23)	(0.28)	(0.21)
ConditionEvil	−0.09	0.16	**0.65***
	(0.27)	(0.34)	(0.25)
ConditionGood	**0.75****	0.41	**0.69***
	(0.29)	(0.34)	(0.27)
Having.Children	**−0.77*****	**−0.52*****	−0.14
	(0.14)	(0.14)	(0.10)
Gender_simpleMale	**−0.53*****	**−0.38****	0.02
	(0.12)	(0.13)	(0.09)
Gender_simpleOther	0.56	0.53	0.13
	(0.41)	(0.39)	(0.29)
Political.Ideology.AllConservative	−0.03	−0.01	0.09
	(0.18)	(0.21)	(0.14)
Political.Ideology.AllLiberal	**0.61*****	**0.40***	**0.63*****
	(0.15)	(0.18)	(0.11)
Age	0.07	0.12	0.05
	(0.06)	(0.06)	(0.04)
Education	**−0.21****	−0.03	−0.02
	(0.07)	(0.05)	(0.03)
News.Checking	**0.18***	**0.18***	**0.19*****
	(0.08)	(0.08)	(0.05)
Social.Media.Use	**−0.23*****	−0.05	0.02
	(0.06)	(0.08)	(0.05)
COVID.Vaccine.Intentions:ConditionEvil	0.38	0.07	−0.38
	(0.31)	(0.38)	(0.28)
COVID.Vaccine.Intentions:ConditionGood	−0.31	−0.08	−0.27
	(0.33)	(0.38)	(0.29)
R^2	0.23	0.19	0.18
Adj. R^2	0.22	0.17	0.17
Num. obs.	689	555	1075

$^{***}p < 0.001$; $^{**}p < 0.01$; $^{*}p < 0.05$

Table S4. Linear Regression Model for Confidence Ratings (Fake Items Only) Including Interaction Between Condition and COVID-Vaccine Intentions

	Study 1	Study 2	Study 3
(Intercept)	**4.93*****	**5.01*****	**4.73*****
	(0.30)	(0.33)	(0.23)
COVID.Vaccine.Intentions	−0.02	**0.42***	**0.36***
	(0.16)	(0.19)	(0.15)
ConditionEvil	−0.32	0.30	0.26
	(0.19)	(0.22)	(0.18)
ConditionGood	−0.03	0.23	−0.08
	(0.20)	(0.22)	(0.19)
Having.Children	**−0.21***	−0.10	−0.03
	(0.10)	(0.09)	(0.07)
Gender__simpleMale	−0.14	−0.09	−0.04
	(0.08)	(0.09)	(0.06)
Gender__simpleOther	0.07	0.20	0.24
	(0.29)	(0.26)	(0.21)
Political.Ideology.AllConservative	−0.02	−0.16	0.08
	(0.13)	(0.14)	(0.10)
Political.Ideology.AllLiberal	**0.26***	0.12	**0.31*****
	(0.10)	(0.12)	(0.07)
Age	**0.12****	0.04	0.05
	(0.04)	(0.04)	(0.03)
Education	0.02	−0.02	−0.04
	(0.05)	(0.03)	(0.02)
News.Checking	0.06	**0.13***	**0.11****
	(0.05)	(0.05)	(0.03)
Social.Media.Use	0.00	−0.02	0.03
	(0.04)	(0.05)	(0.03)
COVID.Vaccine.Intentions:ConditionEvil	**0.57****	−0.06	−0.06
	(0.22)	(0.25)	(0.20)
COVID.Vaccine.Intentions:ConditionGood	0.30	−0.08	0.32
	(0.23)	(0.25)	(0.21)
R^2	0.09	0.09	0.09
Adj. R^2	0.07	0.06	0.08
Num. obs.	689	555	1075

$^{***}p < 0.001$; $^{**}p < 0.01$; $^{*}p < 0.05$

Table S5. Linear Regression Model for Sharing Ratings (Fake Items Only) Including Interaction Between Condition and COVID-Vaccine Intentions

	Study 1	Study 2	Study 3
(Intercept)	0.35	**1.77*****	**2.55*****
	(0.41)	(0.40)	(0.30)
COVID.Vaccine.Intentions	−0.19	**−0.75*****	**−0.47***
	(0.22)	(0.22)	(0.19)
ConditionEvil	0.26	−0.22	0.28
	(0.25)	(0.27)	(0.23)
ConditionGood	−0.18	−0.22	−0.12
	(0.27)	(0.27)	(0.25)
Having.Children	**0.74*****	**0.41*****	**0.26****
	(0.13)	(0.11)	(0.09)
Gender_simpleMale	**0.69*****	**0.40*****	0.09
	(0.11)	(0.10)	(0.08)
Gender_simpleOther	0.03	−0.14	−0.32
	(0.39)	(0.31)	(0.27)
Political.Ideology.AllConservative	0.28	0.14	−0.05
	(0.18)	(0.17)	(0.13)
Political.Ideology.AllLiberal	−0.23	0.01	**−0.51*****
	(0.14)	(0.14)	(0.10)
Age	−0.03	−0.08	−0.00
	(0.05)	(0.05)	(0.04)
Education	**0.24*****	−0.01	−0.02
	(0.07)	(0.04)	(0.03)
News.Checking	−0.10	0.07	−0.03
	(0.07)	(0.06)	(0.04)
Social.Media.Use	**0.29*****	0.09	0.05
	(0.06)	(0.06)	(0.04)
COVID.Vaccine.Intentions:ConditionEvil	−0.39	0.07	−0.25
	(0.30)	(0.30)	(0.25)
COVID.Vaccine.Intentions:ConditionGood	−0.19	0.08	0.07
	(0.31)	(0.30)	(0.27)
R^2	0.21	0.14	0.10
Adj. R^2	0.19	0.11	0.09
Num. obs.	689	555	1075

$^{***}p < 0.001$; $^{**}p < 0.01$; $^{*}p < 0.05$

Table S6. Linear Regression Model for Sharing Ratings (Difference Between Real and Fake) Including Interaction Between Condition and COVID-Vaccine Intentions

	Study 1	Study 2	Study 3
(Intercept)	0.53	−0.70	**−1.11*****
	(0.39)	(0.48)	(0.32)
COVID.Vaccine.Intentions	**0.70****	**0.82****	**0.88*****
	(0.22)	(0.27)	(0.20)
ConditionEvil	−0.10	−0.18	**0.53***
	(0.25)	(0.32)	(0.24)
ConditionGood	**0.56***	0.10	**0.62***
	(0.26)	(0.32)	(0.26)
Having.Children	**−0.50*****	−0.08	−0.16
	(0.13)	(0.13)	(0.10)
Gender_simpleMale	**−0.25***	−0.04	0.03
	(0.11)	(0.12)	(0.08)
Gender_simpleOther	−0.34	**1.00****	**0.85****
	(0.38)	(0.37)	(0.28)
Political.Ideology.AllConservative	−0.04	−0.18	−0.10
	(0.17)	(0.20)	(0.13)
Political.Ideology.AllLiberal	**0.39****	0.04	**0.51*****
	(0.14)	(0.17)	(0.10)
Age	**0.14****	0.09	0.04
	(0.05)	(0.06)	(0.04)
Education	−0.12	0.01	0.03
	(0.06)	(0.05)	(0.03)
News.Checking	0.02	0.13	**0.25*****
	(0.07)	(0.07)	(0.05)
Social.Media.Use	0.05	**0.17***	0.00
	(0.06)	(0.07)	(0.04)
COVID.Vaccine.Intentions:ConditionEvil	0.33	0.33	−0.37
	(0.29)	(0.36)	(0.27)
COVID.Vaccine.Intentions:ConditionGood	0.02	0.11	−0.07
	(0.30)	(0.36)	(0.28)
R^2	0.17	0.14	0.18
Adj. R^2	0.16	0.12	0.17
Num. obs.	689	555	1075

$^{***}p < 0.001$; $^{**}p < 0.01$; $^{*}p < 0.05$

Political Ideology.

Table S7. Linear Regression Model for Manipulativeness Ratings (Fake Items Only) Including Interaction Between Condition and Political Ideology

	Study 1	Study 2	Study 3
(Intercept)	**5.01*****	**4.90*****	**3.72*****
	(0.31)	(0.36)	(0.22)
Political.Ideology.AllConservative	0.16	−0.16	0.11
	(0.23)	(0.25)	(0.16)
Political.Ideology.AllLiberal	0.34	0.12	**0.56*****
	(0.18)	(0.21)	(0.12)
ConditionEvil	0.39	0.33	**0.56*****
	(0.20)	(0.27)	(0.15)
ConditionGood	**0.55****	0.06	**0.35***
	(0.21)	(0.27)	(0.15)
Having.Children	**−0.33****	**−0.31****	−0.06
	(0.10)	(0.10)	(0.07)
Gender_simpleMale	−0.11	**−0.27****	0.07
	(0.08)	(0.09)	(0.06)
Gender_simpleOther	0.23	0.23	−0.01
	(0.30)	(0.28)	(0.21)
COVID.Vaccine.Intentions	**0.60*****	**0.90*****	**0.84*****
	(0.11)	(0.13)	(0.09)
Age	0.05	0.08	0.05
	(0.04)	(0.04)	(0.03)
Education	−0.00	−0.03	−0.02
	(0.05)	(0.04)	(0.02)
News.Checking	0.06	0.03	**0.13*****
	(0.05)	(0.06)	(0.04)
Social.Media.Use	**−0.13****	−0.05	0.01
	(0.05)	(0.06)	(0.03)
Political.Ideology.AllConservative:ConditionEvil	−0.28	0.05	−0.11
	(0.32)	(0.37)	(0.24)
Political.Ideology.AllLiberal:ConditionEvil	−0.22	0.01	−0.18
	(0.24)	(0.30)	(0.18)
Political.Ideology.AllConservative:ConditionGood	−0.28	0.57	0.05
	(0.32)	(0.37)	(0.24)
Political.Ideology.AllLiberal:ConditionGood	−0.14	0.25	−0.05
	(0.25)	(0.30)	(0.18)
R^2	0.14	0.20	0.20
Adj. R^2	0.12	0.18	0.19
Num. obs.	689	555	1075

$^{***}p < 0.001$; $^{**}p < 0.01$; $^{*}p < 0.05$

Table S8. Linear Regression Model for Manipulativeness Ratings (Difference Between Fake and Real) Including Interaction Between Condition and Political Ideology

	Study 1	Study 2	Study 3
(Intercept)	**2.51*****	**1.19***	0.14
	(0.44)	(0.51)	(0.31)
Political.Ideology.AllConservative	0.19	−0.18	0.03
	(0.32)	(0.34)	(0.22)
Political.Ideology.AllLiberal	**0.73****	0.44	**0.68*****
	(0.25)	(0.29)	(0.17)
ConditionEvil	0.37	0.16	**0.45***
	(0.28)	(0.38)	(0.21)
ConditionGood	**0.68***	0.37	0.41
	(0.29)	(0.37)	(0.21)
Having.Children	**−0.76*****	**−0.52*****	−0.14
	(0.14)	(0.14)	(0.10)
Gender__simpleMale	**−0.55*****	**−0.39****	0.02
	(0.12)	(0.13)	(0.09)
Gender__simpleOther	0.60	0.53	0.13
	(0.41)	(0.39)	(0.30)
COVID.Vaccine.Intentions	**0.60*****	**0.98*****	**1.04*****
	(0.15)	(0.18)	(0.13)
Age	0.08	0.12	0.05
	(0.06)	(0.06)	(0.04)
Education	**−0.21****	−0.03	−0.02
	(0.07)	(0.05)	(0.03)
News.Checking	**0.17***	**0.17***	**0.18*****
	(0.08)	(0.08)	(0.05)
Social.Media.Use	**−0.23*****	−0.05	0.01
	(0.06)	(0.08)	(0.05)
Political.Ideology.AllConservative:ConditionEvil	−0.35	0.45	−0.04
	(0.45)	(0.51)	(0.33)
Political.Ideology.AllLiberal:ConditionEvil	−0.21	−0.05	−0.19
	(0.33)	(0.42)	(0.25)
Political.Ideology.AllConservative:ConditionGood	−0.35	0.11	0.20
	(0.45)	(0.52)	(0.33)
Political.Ideology.AllLiberal:ConditionGood	−0.18	−0.07	0.02
	(0.34)	(0.42)	(0.25)
R^2	0.23	0.19	0.18
Adj. R^2	0.21	0.17	0.16
Num. obs.	689	555	1075

$^{***}p < 0.001$; $^{**}p < 0.01$; $^{*}p < 0.05$

Table S9. Linear Regression Model for Confidence Ratings (Fake Items Only) Including Interaction Between Condition and Political Ideology

	Study 1	Study 2	Study 3
(Intercept)	**4.58*****	**4.79*****	**4.69*****
	(0.31)	(0.33)	(0.22)
Political.Ideology.AllConservative	0.36	0.29	0.01
	(0.22)	(0.22)	(0.16)
Political.Ideology.AllLiberal	**0.38***	0.36	**0.30***
	(0.17)	(0.19)	(0.12)
ConditionEvil	0.25	**0.70****	0.04
	(0.19)	(0.24)	(0.15)
ConditionGood	**0.46***	**0.49***	**0.30***
	(0.20)	(0.24)	(0.15)
Having.Children	**−0.20***	−0.12	−0.04
	(0.10)	(0.09)	(0.07)
Gender_simpleMale	−0.15	−0.07	−0.06
	(0.08)	(0.09)	(0.06)
Gender_simpleOther	0.10	0.21	0.24
	(0.29)	(0.26)	(0.21)
COVID.Vaccine.Intentions	**0.29****	**0.35****	**0.42*****
	(0.10)	(0.12)	(0.09)
Age	**0.11****	0.04	0.05
	(0.04)	(0.04)	(0.03)
Education	0.02	−0.03	−0.04
	(0.05)	(0.03)	(0.02)
News.Checking	0.06	**0.16****	**0.11****
	(0.05)	(0.05)	(0.03)
Social.Media.Use	0.00	−0.03	0.03
	(0.04)	(0.05)	(0.03)
Political.Ideology.AllConservative:ConditionEvil	−0.45	**−0.94****	0.21
	(0.31)	(0.33)	(0.23)
Political.Ideology.AllLiberal:ConditionEvil	−0.14	−0.41	0.22
	(0.23)	(0.27)	(0.18)
Political.Ideology.AllConservative:ConditionGood	**−0.70***	−0.48	0.04
	(0.31)	(0.34)	(0.23)
Political.Ideology.AllLiberal:ConditionGood	−0.25	−0.34	−0.18
	(0.24)	(0.27)	(0.17)
R^2	0.09	0.10	0.09
Adj. R^2	0.07	0.08	0.08
Num. obs.	689	555	1075

$^{***}p < 0.001$; $^{**}p < 0.01$; $^{*}p < 0.05$

Table S10. Linear Regression Model for Sharing Ratings (Fake Items Only) Including Interaction Between Condition and Political Ideology

	Study 1	Study 2	Study 3
(Intercept)	0.35	**1.82*****	**2.53*****
	(0.42)	(0.40)	(0.28)
Political.Ideology.AllConservative	**0.63***	0.12	0.02
	(0.30)	(0.27)	(0.20)
Political.Ideology.AllLiberal	−0.16	−0.15	**−0.37***
	(0.23)	(0.23)	(0.16)
ConditionEvil	0.01	−0.29	0.25
	(0.26)	(0.30)	(0.19)
ConditionGood	−0.04	−0.36	0.06
	(0.28)	(0.29)	(0.19)
Having.Children	**0.75*****	**0.41*****	**0.25****
	(0.13)	(0.11)	(0.09)
Gender_simpleMale	**0.70*****	**0.40*****	0.08
	(0.11)	(0.10)	(0.08)
Gender_simpleOther	−0.00	−0.14	−0.32
	(0.39)	(0.31)	(0.27)
COVID.Vaccine.Intentions	**−0.39****	**−0.71*****	**−0.54*****
	(0.14)	(0.14)	(0.12)
Age	−0.03	−0.08	−0.00
	(0.05)	(0.05)	(0.04)
Education	**0.25*****	−0.01	−0.02
	(0.07)	(0.04)	(0.03)
News.Checking	−0.10	0.08	−0.03
	(0.07)	(0.06)	(0.04)
Social.Media.Use	**0.30*****	0.09	0.05
	(0.06)	(0.06)	(0.04)
Political.Ideology.AllConservative:ConditionEvil	−0.46	−0.08	−0.17
	(0.43)	(0.40)	(0.30)
Political.Ideology.AllLiberal:ConditionEvil	0.06	0.22	−0.26
	(0.32)	(0.33)	(0.23)
Political.Ideology.AllConservative:ConditionGood	−0.64	0.14	−0.07
	(0.43)	(0.41)	(0.30)
Political.Ideology.AllLiberal:ConditionGood	−0.28	0.30	−0.19
	(0.33)	(0.33)	(0.22)
R^2	0.21	0.14	0.10
Adj. R^2	0.19	0.11	0.09
Num. obs.	689	555	1075

$^{***}p < 0.001$; $^{**}p < 0.01$; $^{*}p < 0.05$

Table S11. Linear Regression Model for Sharing Ratings (Difference Between Real and Fake) Including Interaction Between Condition and Political Ideology

	Study 1	Study 2	Study 3
(Intercept)	0.49	−0.89	**−0.97****
	(0.40)	(0.48)	(0.30)
Political.Ideology.AllConservative	−0.10	−0.33	−0.12
	(0.29)	(0.33)	(0.21)
Political.Ideology.AllLiberal	0.28	0.17	**0.56*****
	(0.23)	(0.28)	(0.17)
ConditionEvil	−0.03	0.05	0.19
	(0.25)	(0.35)	(0.20)
ConditionGood	**0.54***	0.36	**0.67*****
	(0.27)	(0.35)	(0.20)
Having.Children	**−0.50*****	−0.07	−0.17
	(0.13)	(0.13)	(0.10)
Gender_simpleMale	**−0.26***	−0.03	0.02
	(0.11)	(0.12)	(0.09)
Gender_simpleOther	−0.34	**1.01****	**0.87****
	(0.38)	(0.37)	(0.28)
COVID.Vaccine.Intentions	**0.85*****	**0.99*****	**0.71*****
	(0.13)	(0.17)	(0.12)
Age	**0.13***	0.09	0.04
	(0.05)	(0.06)	(0.04)
Education	−0.12	0.01	0.03
	(0.06)	(0.05)	(0.03)
News.Checking	0.01	0.12	**0.24*****
	(0.07)	(0.07)	(0.05)
Social.Media.Use	0.05	**0.18***	−0.00
	(0.06)	(0.07)	(0.04)
Political.Ideology.AllConservative:ConditionEvil	0.39	0.36	−0.04
	(0.41)	(0.48)	(0.31)
Political.Ideology.AllLiberal:ConditionEvil	0.18	−0.06	0.05
	(0.30)	(0.40)	(0.24)
Political.Ideology.AllConservative:ConditionGood	−0.23	0.17	0.07
	(0.41)	(0.49)	(0.32)
Political.Ideology.AllLiberal:ConditionGood	0.12	−0.31	−0.20
	(0.32)	(0.39)	(0.24)
R^2	0.18	0.14	0.18
Adj. R^2	0.16	0.12	0.16
Num. obs.	689	555	1075

$^{***}p < 0.001$; $^{**}p < 0.01$; $^{*}p < 0.05$

Inoculation Theory

Inoculation theory is built on an immunization analogy (3), but focuses on psychological rather than physiological immunization. It posits that exposure to a weakened persuasive argument, much like exposure to a weakened version of a pathogen, protects against future exposure to persuasive arguments (4). Interventions based on inoculation theory have been shown to protect against persuasive arguments in a range of contexts, including health (see 5) and politics (see 6). See (7) for a meta-analysis and (5) and (8) for reviews.

Inoculation strategies can be differentiated along three theoretical lines: therapeutic vs. prophylactic (i.e., the timing of the intervention), active vs. passive (i.e., the level of engagement by the individual), and issue-based vs. technique-based inoculation (i.e., the design of the intervention). *Therapeutic* inoculation aims to confer resistance to persuasive arguments to individuals who have already been exposed to such arguments, while *prophylactic* inoculation aims to confer resistance to individuals who have not yet been exposed (9). Importantly, inoculation can work in both therapeutic and prophylactic contexts (9), making this approach amenable for use with the general population, which includes individuals with different pre-exposure levels. However, inoculation might be less effective for individuals who already believe in vaccine misinformation like anti-vaccine conspiracy theories (10).

Active inoculation implies that individuals actively take part in the inoculation process, for example by playing a browser game where they can make choices. In contrast, *passive* inoculation only provides individuals with information aimed at inoculating them, for example in the form of an informational text. Most traditional inoculation interventions focused on passive inoculation methods (7, 11), but more recent studies have utilized active inoculation approaches, such as online games (12), which may be more effective than passive inoculation (13).

Issue-based inoculation focuses on a specific domain where inoculation is to be achieved, whereas *technique-based* inoculation focuses on the strategies that underlie the spread of persuasive arguments and is expected to be domain-general (13). Issue-based approaches, such as those with a narrow focus on specific arguments against vaccines (10) or climate change (14), work by providing counterarguments to persuasive arguments in one specific area. Technique-based approaches are informed by common misinformation techniques. For example, the *Bad News* game (https://getbadnews.com) aiming to inoculate players against generic misinformation includes the techniques of polarizing audiences and appealing to emotion (12, 15).

Identifying Vaccine Misinformation Techniques

Vaccine misinformation follows predictable tropes or misinformation techniques (16–18). We identified four such techniques that are commonly encountered online to develop our intervention: emotional storytelling, fake expertise and pseudoscience, the naturalistic fallacy, and conspiracy theories (17, 18).

Emotional storytelling is the use of emotional narratives to provoke strong emotional responses. This technique is commonly found in anti-vaccine information channels (17–19). For example, an analysis of anti-vaccination websites showed that 88% used emotional appeals to promote anti-vaccine misinformation (17).

The second technique combines fake expertise, where an individual pretends to be an expert on vaccines, and pseudoscience, where false information is given an air of credibility by drawing on scientific-seeming language (18, 20). (18) summarizes this technique as "skewing the science", where pro-vaccine content is rejected, but false scientific claims that align with anti-vaccine sentiment are promoted.

The naturalistic fallacy asserts that everything that is natural is good, and everything that is unnatural is bad (17, 18). For example, vaccines might be depicted as something unnatural and unsafe because they allegedly contain toxins (17). Proponents of this fallacy argue in favor of "naturally" acquiring immunity from diseases by contracting them, and oppose vaccination (18).

Finally, conspiracy theories that assert that vaccines are part of a conspiracy by actors such as the government or pharmaceutical companies are frequently used to spread vaccine misinformation (17, 18, 21, 22). For example, one conspiracy theory claims that vaccination supporters are only supportive of vaccines because they are paid by "Big Pharma" (18). Further, a survey analysis suggests that anti-vaccination attitudes are especially common among individuals high in conspiratorial thinking (23).

Descriptive Statistics

Number of Observations.

Demographics. Tables S13-S15 show basic demographic information about study participants in Studies 1 to 3. Age was coded in five buckets (18-24 = 1, 25-34 = 2, 35-44 = 3, 45-54 = 4 , >55 = 5), parental status in two buckets (not having children = 0, having children = 1), and political ideology in three buckets (Liberal = -1, Moderate = 0, Conservative = 1).

Education was coded in six buckets in Study 1 (No formal education above age 16 = 1, Professional or technical qualifications above age 16 = 2, School education up to age 18 = 3, Degree (Bachelor's) or equivalent = 4, Degree (Master's) or other postgraduate qualification = 5, Doctorate = 6) and in seven buckets in Studies 2 and 3 (Less than high school = 1, High school graduate = 2, Some college = 3, 2 year degree = 4, 4 year degree = 5, Professional degree = 6, Doctorate = 7).

We use a binarized gender variable to calculate mean and standard deviation (Female = 0, Male = 1). For the proportions of gender measure with three categories (Female, Male, Other), see the Balance Tables section.

Table S12. Number of Observations for Studies 1, 2 and 3

	Study 1	Study 2	Study 3
Preregistered target sample size	720	600	1200
Observations in raw data	863	671	1353
Observations after removing preview responses	863	667	1353
Observations after removing non-consenting participants	861	666	1342
Observations after removing participants who did not finish the survey	763	599	1187
Observations after removing participants with empty Prolific IDs	763	599	1157
Observations after removing participants who failed attentions check	695	563	1082
Observations after removing participants who got password wrong	690	559	1079
Observations after removing participants with missing values on Manipulativeness Discernment	690	557	1079

Table S13. Demographics for Study 1

Variable	n	mean	sd	median	min	max
Age Group (18-24 = 1, 25-34 = 2, 35-44 = 3, 45-54 = 4 , >55 = 5)	690	2.51	1.22	2.00	1	5
Gender (Female = 0, Male = 1)	675	0.51	0.50	1.00	0	1
Parental Status (not having children = 0, having children = 1)	690	0.40	0.49	0.00	0	1
Political Ideology (Liberal = -1, Moderate = 0, Conservative = 1)	690	-0.43	0.76	-1.00	-1	1
Education (No formal education above age 16 = 1, ..., Doctorate = 6)	690	3.98	0.88	4.00	1	6

Table S14. Demographics for Study 2

Variable	n	mean	sd	median	min	max
Age Group (18-24 = 1, 25-34 = 2, 35-44 = 3, 45-54 = 4 , >55 = 5)	557	2.71	1.19	2.00	1	5
Gender (Female = 0, Male = 1)	540	0.43	0.50	0.00	0	1
Parental Status (not having children = 0, having children = 1)	557	0.40	0.49	0.00	0	1
Political Ideology (Liberal = -1, Moderate = 0, Conservative = 1)	557	-0.44	0.80	-1.00	-1	1
Education (Less than high school = 1, ..., Doctorate = 7)	557	4.54	1.32	5.00	1	7

Table S15. Demographics for Study 3

Variable	n	mean	sd	median	min	max
Age Group (18-24 = 1, 25-34 = 2, 35-44 = 3, 45-54 = 4 , >55 = 5)	1079	2.65	1.39	2.00	1	5
Gender (Female = 0, Male = 1)	1051	0.50	0.50	0.00	0	1
Parental Status (not having children = 0, having children = 1)	1079	0.38	0.49	0.00	0	1
Political Ideology (Liberal = -1, Moderate = 0, Conservative = 1)	1079	-0.44	0.76	-1.00	-1	1
Education (Less than high school = 1, ..., Doctorate = 7)	1079	4.21	1.39	5.00	1	7

Group Means for Outcome Variables.

Table S16. Group means for Study 1

Variable	Condition	N	Mean	Median	SD	SE
Misinformation Manipulativeness	Control	227	5.324	5.500	1.213	0.080
	Evil	237	5.514	5.750	1.147	0.075
	Good	226	5.722	6.000	1.030	0.069
Non-Misinformation Manipulativeness	Control	227	5.324	5.500	1.213	0.080
	Evil	237	5.514	5.750	1.147	0.075
	Good	226	5.722	6.000	1.030	0.069
Manipulativeness Discernment	Control	227	5.324	5.500	1.213	0.080
	Evil	237	5.514	5.750	1.147	0.075
	Good	226	5.722	6.000	1.030	0.069
Misinformation Confidence	Control	227	5.324	5.500	1.213	0.080
	Evil	237	5.514	5.750	1.147	0.075
	Good	226	5.722	6.000	1.030	0.069
Non-Misinformation Confidence	Control	227	5.324	5.500	1.213	0.080
	Evil	237	5.514	5.750	1.147	0.075
	Good	226	5.722	6.000	1.030	0.069
Misinformation Sharing Intent	Control	227	5.324	5.500	1.213	0.080
	Evil	237	5.514	5.750	1.147	0.075
	Good	226	5.722	6.000	1.030	0.069
Non-Misinformation Sharing Intent	Control	227	5.324	5.500	1.213	0.080
	Evil	237	5.514	5.750	1.147	0.075
	Good	226	5.722	6.000	1.030	0.069
Sharing Intent Discernment	Control	227	5.324	5.500	1.213	0.080
	Evil	237	5.514	5.750	1.147	0.075
	Good	226	5.722	6.000	1.030	0.069

Table S17. Group means for Study 2

Variable	Condition	N	Mean	Median	SD	SE
Misinformation Manipulativeness	Control	196	5.438	5.667	1.248	0.089
	Evil	182	5.761	6.000	1.184	0.088
	Good	179	5.788	5.875	0.990	0.074
Non-Misinformation Manipulativeness	Control	196	5.438	5.667	1.248	0.089
	Evil	182	5.761	6.000	1.184	0.088
	Good	179	5.788	5.875	0.990	0.074
Manipulativeness Discernment	Control	196	5.438	5.667	1.248	0.089
	Evil	182	5.761	6.000	1.184	0.088
	Good	179	5.788	5.875	0.990	0.074
Misinformation Confidence	Control	196	5.438	5.667	1.248	0.089
	Evil	182	5.761	6.000	1.184	0.088
	Good	179	5.788	5.875	0.990	0.074
Non-Misinformation Confidence	Control	196	5.438	5.667	1.248	0.089
	Evil	182	5.761	6.000	1.184	0.088
	Good	179	5.788	5.875	0.990	0.074
Misinformation Sharing Intent	Control	196	5.438	5.667	1.248	0.089
	Evil	182	5.761	6.000	1.184	0.088
	Good	179	5.788	5.875	0.990	0.074
Non-Misinformation Sharing Intent	Control	196	5.438	5.667	1.248	0.089
	Evil	182	5.761	6.000	1.184	0.088
	Good	179	5.788	5.875	0.990	0.074
Sharing Intent Discernment	Control	196	5.438	5.667	1.248	0.089
	Evil	182	5.761	6.000	1.184	0.088
	Good	179	5.788	5.875	0.990	0.074

Table S18. Group means for Study 3

Variable	Condition	N	Mean	Median	SD	SE
Misinformation Manipulativeness	Control	383	5.352	5.600	1.202	0.061
	Evil	344	5.781	6.000	1.034	0.056
	Good	352	5.713	5.857	1.020	0.054
Non-Misinformation Manipulativeness	Control	383	5.352	5.600	1.202	0.061
	Evil	344	5.781	6.000	1.034	0.056
	Good	352	5.713	5.857	1.020	0.054
Manipulativeness Discernment	Control	383	5.352	5.600	1.202	0.061
	Evil	344	5.781	6.000	1.034	0.056
	Good	352	5.713	5.857	1.020	0.054
Misinformation Confidence	Control	383	5.352	5.600	1.202	0.061
	Evil	344	5.781	6.000	1.034	0.056
	Good	352	5.713	5.857	1.020	0.054
Non-Misinformation Confidence	Control	383	5.352	5.600	1.202	0.061
	Evil	344	5.781	6.000	1.034	0.056
	Good	352	5.713	5.857	1.020	0.054
Misinformation Sharing Intent	Control	383	5.352	5.600	1.202	0.061
	Evil	344	5.781	6.000	1.034	0.056
	Good	352	5.713	5.857	1.020	0.054
Non-Misinformation Sharing Intent	Control	383	5.352	5.600	1.202	0.061
	Evil	344	5.781	6.000	1.034	0.056
	Good	352	5.713	5.857	1.020	0.054
Sharing Intent Discernment	Control	383	5.352	5.600	1.202	0.061
	Evil	344	5.781	6.000	1.034	0.056
	Good	352	5.713	5.857	1.020	0.054

Balance Tables

Table S19. Balance Table for Treatment Condition for Study 1

Variable	Condition			p-value	SMD
	Control	Evil	Good		
Number of Observations	227	237	226		
Participant (mean (SD))	343.63 (186.59)	339.68 (206.33)	353.48 (204.80)	0.747	0.046
Parental Status = Having Children (N (%))	87 (38.3)	92 (38.8)	97 (42.9)	0.547	0.062
Gender (N (%))				0.817	0.075
... Female	106 (46.7)	116 (49.2)	112 (49.6)		
... Male	118 (52.0)	114 (48.3)	109 (48.2)		
... Other	3 (1.3)	6 (2.5)	5 (2.2)		
Political Ideology (N (%))				0.691	0.092
... Liberal	135 (59.5)	139 (58.6)	135 (59.7)		
... Moderate	54 (23.8)	64 (27.0)	50 (22.1)		
... Conservative	38 (16.7)	34 (14.3)	41 (18.1)		
Covid Vaccinination Intentions = Yes (N (%))	167 (73.6)	170 (71.7)	176 (77.9)	0.301	0.095
Age (N (%))				0.276	0.205
... 18-24	44 (19.4)	51 (21.5)	59 (26.1)		
... 25-34	85 (37.4)	90 (38.0)	69 (30.5)		
... 35-44	46 (20.3)	41 (17.3)	51 (22.6)		
... 45-54	27 (11.9)	36 (15.2)	32 (14.2)		
... >55	25 (11.0)	19 (8.0)	15 (6.6)		
Education (N (%))				0.880	0.137
... No formal education above age 16	0 (0.0)	0 (0.0)	1 (0.4)		
... Professional or technical qualifications above age 16	4 (1.8)	6 (2.5)	4 (1.8)		
... School education up to age 18	70 (30.8)	70 (29.5)	65 (28.8)		
... Degree (Bachelor's) or equivalent	82 (36.1)	97 (40.9)	95 (42.0)		
... Degree (Master's) or other postgraduate qualification	61 (26.9)	57 (24.1)	54 (23.9)		
... Doctorate	10 (4.4)	7 (3.0)	7 (3.1)		
News Checking Frequency (N (%))				0.494	0.181
... Never	4 (1.8)	2 (0.8)	0 (0.0)		
... Rarely	15 (6.6)	15 (6.3)	14 (6.2)		
... Sometimes	61 (26.9)	62 (26.2)	54 (23.9)		
... Frequently	110 (48.5)	108 (45.6)	106 (46.9)		
... All the time	37 (16.3)	50 (21.1)	52 (23.0)		
Social Media Use Frequency (N (%))				0.073	0.248
... Never	7 (3.1)	2 (0.8)	4 (1.8)		
... Rarely	11 (4.8)	10 (4.2)	10 (4.4)		
... Sometimes	48 (21.1)	44 (18.6)	28 (12.4)		
... Frequently	88 (38.8)	93 (39.2)	81 (35.8)		
... All the time	73 (32.2)	88 (37.1)	103 (45.6)		
Marital Status Married (mean (SD))	0.43 (0.50)	0.42 (0.49)	0.42 (0.50)	0.955	0.019
Percentage Treatment Posts (mean (SD))	0.45 (0.14)	0.47 (0.14)	0.45 (0.13)	0.388	0.077

Note: p-values result from a joint F-test for continuous variables and from a Chi-squared test for categorical variables.

Table S20. Balance Table for Treatment Condition for Study 2

Variable	Condition			p-value	SMD
	Control	Evil	Good		
Number of Observations	196	182	179		
Participant (mean (SD))	286.66 (164.16)	285.49 (163.41)	267.58 (157.42)	0.450	0.079
Parental Status = Having Children (N (%))	90 (45.9)	67 (36.8)	68 (38.0)	0.143	0.124
Gender (N (%))				0.530	0.128
... Female	107 (54.9)	93 (51.4)	106 (59.2)		
... Male	84 (43.1)	83 (45.9)	67 (37.4)		
... Other	4 (2.1)	5 (2.8)	6 (3.4)		
Political Ideology (N (%))				0.823	0.086
... Liberal	120 (61.2)	116 (63.7)	119 (66.5)		
... Moderate	34 (17.3)	30 (16.5)	30 (16.8)		
... Conservative	42 (21.4)	36 (19.8)	30 (16.8)		
Covid Vaccinination Intentions = Yes (N (%))	161 (82.1)	140 (76.9)	140 (78.2)	0.426	0.086
Age (N (%))				0.758	0.157
... 18-24	23 (11.7)	29 (15.9)	18 (10.1)		
... 25-34	80 (40.8)	73 (40.1)	69 (38.5)		
... 35-44	45 (23.0)	42 (23.1)	44 (24.6)		
... 45-54	24 (12.2)	18 (9.9)	27 (15.1)		
... >55	24 (12.2)	20 (11.0)	21 (11.7)		
Education (N (%))				0.703	0.230
... Less than high school	2 (1.0)	1 (0.5)	0 (0.0)		
... High school graduate	17 (8.7)	14 (7.7)	18 (10.1)		
... Some college	35 (17.9)	37 (20.3)	25 (14.0)		
... 2 year degree	15 (7.7)	15 (8.2)	15 (8.4)		
... 4 year degree	80 (40.8)	81 (44.5)	78 (43.6)		
... Professional degree	44 (22.4)	28 (15.4)	37 (20.7)		
... Doctorate	3 (1.5)	6 (3.3)	6 (3.4)		
News Checking Frequency (N (%))				0.749	0.160
... Never	2 (1.0)	4 (2.2)	2 (1.1)		
... Rarely	9 (4.6)	16 (8.8)	14 (7.8)		
... Sometimes	54 (27.6)	41 (22.5)	46 (25.7)		
... Frequently	91 (46.4)	88 (48.4)	82 (45.8)		
... All the time	40 (20.4)	33 (18.1)	35 (19.6)		
Social Media Use Frequency (N (%))				0.787	0.164
... Never	0 (0.0)	1 (0.5)	1 (0.6)		
... Rarely	5 (2.6)	10 (5.5)	9 (5.0)		
... Sometimes	34 (17.3)	25 (13.7)	32 (17.9)		
... Frequently	88 (44.9)	84 (46.2)	77 (43.0)		
... All the time	69 (35.2)	62 (34.1)	60 (33.5)		
Urban Rural Status = Urban (N (%))	148 (75.5)	139 (76.4)	134 (74.9)	0.945	0.023
Income (mean (SD))	7.00 (3.21)	6.96 (3.32)	7.31 (3.21)	0.532	0.072
Percentage Treatment Posts (mean (SD))	0.47 (0.14)	0.43 (0.14)	0.47 (0.14)	0.020	0.180

Note: p-values result from a joint F-test for continuous variables and from a Chi-squared test for categorical variables.

Table S21. Balance Table for Treatment Condition for Study 3

Variable	Condition			p-value	SMD
	Control	Evil	Good		
Number of Observations	383	344	352		
Participant (mean (SD))	531.53 (311.91)	537.62 (313.25)	551.55 (310.25)	0.675	0.043
Parental Status = Having Children (N (%))	155 (40.5)	138 (40.1)	121 (34.4)	0.171	0.084
Gender (N (%))				0.142	0.131
... Female	183 (47.8)	175 (51.3)	172 (49.0)		
... Male	186 (48.6)	163 (47.8)	172 (49.0)		
... Other	14 (3.7)	3 (0.9)	7 (2.0)		
Political Ideology (N (%))				0.797	0.066
... Liberal	223 (58.2)	210 (61.0)	219 (62.2)		
... Moderate	93 (24.3)	77 (22.4)	81 (23.0)		
... Conservative	67 (17.5)	57 (16.6)	52 (14.8)		
Covid Vaccinination Intentions = Yes (N (%))	325 (84.9)	283 (82.3)	304 (86.4)	0.320	0.075
Age (N (%))				0.955	0.084
... 18-24	95 (24.8)	91 (26.5)	83 (23.6)		
... 25-34	105 (27.4)	95 (27.6)	110 (31.2)		
... 35-44	71 (18.5)	65 (18.9)	63 (17.9)		
... 45-54	45 (11.7)	41 (11.9)	42 (11.9)		
... >55	67 (17.5)	52 (15.1)	54 (15.3)		
Education (N (%))				0.458	0.191
... Less than high school	6 (1.6)	0 (0.0)	4 (1.1)		
... High school graduate	44 (11.5)	41 (11.9)	52 (14.8)		
... Some college	91 (23.8)	84 (24.4)	77 (21.9)		
... 2 year degree	33 (8.6)	38 (11.0)	28 (8.0)		
... 4 year degree	141 (36.8)	121 (35.2)	136 (38.6)		
... Professional degree	61 (15.9)	54 (15.7)	46 (13.1)		
... Doctorate	7 (1.8)	6 (1.7)	9 (2.6)		
News Checking Frequency (N (%))				0.096	0.193
... Never	8 (2.1)	6 (1.7)	8 (2.3)		
... Rarely	44 (11.5)	30 (8.7)	41 (11.6)		
... Sometimes	110 (28.7)	95 (27.6)	119 (33.8)		
... Frequently	170 (44.4)	156 (45.3)	121 (34.4)		
... All the time	51 (13.3)	57 (16.6)	63 (17.9)		
Social Media Use Frequency (N (%))				0.398	0.155
... Never	10 (2.6)	2 (0.6)	8 (2.3)		
... Rarely	30 (7.8)	19 (5.5)	27 (7.7)		
... Sometimes	67 (17.5)	62 (18.0)	52 (14.8)		
... Frequently	141 (36.8)	140 (40.7)	135 (38.4)		
... All the time	135 (35.2)	121 (35.2)	130 (36.9)		
Political Party (N (%))				0.358	0.134
... Democrat	178 (47.7)	178 (53.1)	180 (53.4)		
... Independent	129 (34.6)	103 (30.7)	91 (27.0)		
... Republican	48 (12.9)	39 (11.6)	44 (13.1)		
... Other	18 (4.8)	15 (4.5)	22 (6.5)		
Percentage Treatment Posts (mean (SD))	0.45 (0.14)	0.45 (0.14)	0.47 (0.14)	0.236	0.077

Note: p-values result from a joint F-test for continuous variables and from a Chi-squared test for categorical variables.

Detailed Results

Study 1.

Bartlett Test of Homogeneity of Variances.

Table S22. Bartlett Test of Homogeneity of Variances for Study 1

Variable	Bartlett's K-squared	*df*	*p*
Misinformation Manipulativeness	6.09	2	**0.05**
Non-Misinformation Manipulativeness	2.11	2	0.35
Misinformation Manipulativeness	6.09	2	**0.05**
Manipulativeness Discernment	1.35	2	0.51
Misinformation Confidence	0.76	2	0.69
Non-Misinformation Confidence	2.07	2	0.36
Misinformation Sharing Intent	3.43	2	0.18
Non-Misinformation Sharing Intent	0.06	2	0.97
Sharing Intent Discernment	5.61	2	0.06

Linear Regression Models.

Manipulativess

Table S23. Linear Regression Model for Manipulativeness Ratings (Fake Items Only) for Study 1

Predictor	*b*	95% CI	*t*	*df*	*p*
Intercept	5.12	[4.54, 5.70]	17.38	676	< .001
ConditionEvil	0.21	[0.02, 0.41]	2.13	676	.034
ConditionGood	0.41	[0.21, 0.61]	4.06	676	< .001
Having Children	-0.33	[-0.52, -0.13]	-3.33	676	< .001
Gender simpleMale	-0.10	[-0.27, 0.06]	-1.24	676	.216
Gender simpleOther	0.23	[-0.35, 0.81]	0.78	676	.433
Political Ideology AllLiberal	0.22	[0.01, 0.42]	2.06	676	.040
Political Ideology AllConservative	-0.02	[-0.28, 0.24]	-0.18	676	.857
COVID Vaccine Intentions	0.60	[0.40, 0.81]	5.75	676	< .001
Age	0.05	[-0.03, 0.13]	1.23	676	.218
Education	0.00	[-0.10, 0.09]	-0.08	676	.933
News Checking	0.06	[-0.05, 0.16]	1.03	676	.304
Social Media Use	-0.13	[-0.22, -0.04]	-2.89	676	.004

Table S24. Linear Regression Model for Manipulativeness Ratings (Real Items Only) for Study 1

Predictor	*b*	95% CI	*t*	*df*	*p*
Intercept	2.49	[1.87, 3.10]	7.93	676	< .001
ConditionEvil	0.03	[-0.18, 0.24]	0.27	676	.784
ConditionGood	-0.10	[-0.31, 0.12]	-0.90	676	.367
Having Children	0.44	[0.23, 0.64]	4.19	676	< .001
Gender simpleMale	0.44	[0.27, 0.62]	4.95	676	< .001
Gender simpleOther	-0.36	[-0.98, 0.26]	-1.15	676	.249
Political Ideology AllLiberal	-0.38	[-0.60, -0.16]	-3.42	676	< .001
Political Ideology AllConservative	0.02	[-0.25, 0.30]	0.18	676	.859
COVID Vaccine Intentions	0.00	[-0.21, 0.22]	0.04	676	.966
Age	-0.03	[-0.12, 0.06]	-0.68	676	.495
Education	0.21	[0.10, 0.31]	3.90	676	< .001
News Checking	-0.11	[-0.23, 0.00]	-1.97	676	.049
Social Media Use	0.10	[0.01, 0.20]	2.12	676	.034

Table S25. Linear Regression Model for Manipulativeness Ratings (Difference between Fake and Real) for Study 1

Predictor	b	95% CI	t	df	p
Intercept	2.63	[1.83, 3.44]	6.43	676	$<$ .001
ConditionEvil	0.18	[-0.09, 0.46]	1.32	676	.187
ConditionGood	0.51	[0.23, 0.79]	3.61	676	$<$ .001
Having Children	-0.77	[-1.04, -0.50]	-5.61	676	$<$ .001
Gender simpleMale	-0.55	[-0.77, -0.32]	-4.68	676	$<$ .001
Gender simpleOther	0.60	[-0.21, 1.41]	1.45	676	.148
Political Ideology AllLiberal	0.60	[0.31, 0.89]	4.11	676	$<$ .001
Political Ideology AllConservative	-0.05	[-0.41, 0.31]	-0.27	676	.791
COVID Vaccine Intentions	0.60	[0.31, 0.88]	4.11	676	$<$ .001
Age	0.08	[-0.03, 0.19]	1.41	676	.159
Education	-0.21	[-0.35, -0.08]	-3.05	676	.002
News Checking	0.17	[0.02, 0.32]	2.25	676	.025
Social Media Use	-0.23	[-0.35, -0.11]	-3.70	676	$<$ .001

Confidence

Table S26. Linear Regression Model for Confidence Ratings (Fake Items Only) for Study 1

Predictor	b	95% CI	t	df	p
Intercept	4.75	[4.18, 5.31]	16.56	676	$<$ .001
ConditionEvil	0.09	[-0.10, 0.28]	0.94	676	.345
ConditionGood	0.19	[0.00, 0.38]	1.92	676	.055
Having Children	-0.21	[-0.40, -0.02]	-2.17	676	.031
Gender simpleMale	-0.14	[-0.30, 0.02]	-1.78	676	.076
Gender simpleOther	0.11	[-0.46, 0.67]	0.37	676	.711
Political Ideology AllLiberal	0.26	[0.06, 0.46]	2.53	676	.012
Political Ideology AllConservative	-0.02	[-0.28, 0.23]	-0.18	676	.857
COVID Vaccine Intentions	0.28	[0.08, 0.48]	2.79	676	.005
Age	0.11	[0.04, 0.19]	2.87	676	.004
Education	0.02	[-0.08, 0.11]	0.32	676	.750
News Checking	0.06	[-0.04, 0.16]	1.12	676	.264
Social Media Use	0.00	[-0.09, 0.08]	-0.04	676	.971

Table S27. Linear Regression Model for Confidence Ratings (Real Items Only) for Study 1

Predictor	b	95% CI	t	df	p
Intercept	4.13	[3.57, 4.69]	14.49	676	$<$ .001
ConditionEvil	0.09	[-0.10, 0.28]	0.89	676	.373
ConditionGood	0.08	[-0.11, 0.28]	0.84	676	.401
Having Children	-0.25	[-0.44, -0.06]	-2.61	676	.009
Gender simpleMale	-0.01	[-0.17, 0.15]	-0.11	676	.912
Gender simpleOther	0.10	[-0.46, 0.67]	0.36	676	.717
Political Ideology AllLiberal	0.17	[-0.03, 0.37]	1.69	676	.091
Political Ideology AllConservative	-0.08	[-0.34, 0.17]	-0.66	676	.512
COVID Vaccine Intentions	0.11	[-0.09, 0.31]	1.12	676	.265
Age	0.16	[0.08, 0.23]	3.92	676	$<$ .001
Education	0.04	[-0.05, 0.14]	0.86	676	.391
News Checking	0.07	[-0.03, 0.17]	1.34	676	.180
Social Media Use	0.06	[-0.03, 0.14]	1.30	676	.195

Sharing Intent

Table S28. Linear Regression Model for Sharing Ratings (Fake Items Only) for Study 1

Predictor	*b*	95% CI	*t*	*df*	*p*
Intercept	0.47	[-0.30, 1.23]	1.19	676	.235
ConditionEvil	-0.03	[-0.29, 0.23]	-0.20	676	.840
ConditionGood	-0.31	[-0.58, -0.05]	-2.33	676	.020
Having Children	0.74	[0.48, 1.00]	5.65	676	$< .001$
Gender simpleMale	0.70	[0.48, 0.92]	6.28	676	$< .001$
Gender simpleOther	0.00	[-0.77, 0.78]	0.01	676	.993
Political Ideology AllLiberal	-0.22	[-0.50, 0.05]	-1.60	676	.111
Political Ideology AllConservative	0.28	[-0.07, 0.62]	1.58	676	.114
COVID Vaccine Intentions	-0.40	[-0.67, -0.13]	-2.86	676	.004
Age	-0.03	[-0.13, 0.08]	-0.51	676	.608
Education	0.24	[0.11, 0.37]	3.69	676	$< .001$
News Checking	-0.10	[-0.24, 0.05]	-1.34	676	.180
Social Media Use	0.30	[0.18, 0.41]	4.98	676	$< .001$

Table S29. Linear Regression Model for Sharing Ratings (Real Items Only) for Study 1

Predictor	*b*	95% CI	*t*	*df*	*p*
Intercept	0.92	[0.13, 1.72]	2.29	676	.023
ConditionEvil	0.11	[-0.16, 0.38]	0.79	676	.431
ConditionGood	0.25	[-0.02, 0.53]	1.82	676	.069
Having Children	0.24	[-0.03, 0.50]	1.77	676	.078
Gender simpleMale	0.44	[0.21, 0.66]	3.82	676	$< .001$
Gender simpleOther	-0.31	[-1.11, 0.49]	-0.76	676	.445
Political Ideology AllLiberal	0.16	[-0.12, 0.45]	1.12	676	.261
Political Ideology AllConservative	0.23	[-0.13, 0.58]	1.25	676	.211
COVID Vaccine Intentions	0.43	[0.15, 0.71]	2.99	676	.003
Age	0.11	[0.00, 0.22]	1.96	676	.051
Education	0.12	[-0.01, 0.26]	1.78	676	.075
News Checking	-0.08	[-0.23, 0.06]	-1.12	676	.264
Social Media Use	0.34	[0.22, 0.47]	5.59	676	$< .001$

Table S30. Linear Regression Model for Sharing Ratings (Difference between Fake and Real) for Study 1

Predictor	*b*	95% CI	*t*	*df*	*p*
Intercept	0.46	[-0.28, 1.20]	1.21	676	.225
ConditionEvil	0.13	[-0.12, 0.39]	1.05	676	.294
ConditionGood	0.57	[0.31, 0.83]	4.36	676	$< .001$
Having Children	-0.50	[-0.75, -0.25]	-3.97	676	$< .001$
Gender simpleMale	-0.26	[-0.47, -0.05]	-2.42	676	.016
Gender simpleOther	-0.31	[-1.06, 0.43]	-0.83	676	.409
Political Ideology AllLiberal	0.39	[0.12, 0.65]	2.86	676	.004
Political Ideology AllConservative	-0.05	[-0.38, 0.28]	-0.30	676	.764
COVID Vaccine Intentions	0.83	[0.56, 1.09]	6.17	676	$< .001$
Age	0.14	[0.03, 0.24]	2.63	676	.009
Education	-0.12	[-0.25, 0.00]	-1.92	676	.056
News Checking	0.01	[-0.12, 0.15]	0.19	676	.846
Social Media Use	0.05	[-0.07, 0.16]	0.82	676	.412

Multilevel Models.

Manipulativess

Table S31. Multilevel Model for Manipulativeness Ratings (All Items) for Study 1

Term	$\hat{\beta}$	95% CI	t	df	p
Intercept	5.06	[4.65, 5.48]	23.84	38.50	$<$ .001
ConditionEvil	0.23	[0.05, 0.41]	2.57	1230.45	.010
ConditionGood	0.38	[0.20, 0.56]	4.18	1238.96	$<$ .001
Matched controlTRUE	-1.82	[-2.33, -1.32]	-7.14	23.85	$<$ .001
Having Children	0.07	[-0.08, 0.22]	0.91	675.83	.363
Gender simpleMale	0.17	[0.05, 0.30]	2.69	676.69	.007
Gender simpleOther	-0.06	[-0.50, 0.39]	-0.25	675.78	.802
Political Ideology AllLiberal	-0.08	[-0.24, 0.07]	-1.03	675.96	.301
Political Ideology AllConservative	-0.02	[-0.21, 0.18]	-0.15	675.88	.882
COVID Vaccine Intentions	0.29	[0.13, 0.44]	3.58	676.02	$<$ .001
Age scaled	0.01	[-0.06, 0.09]	0.32	676.36	.751
Education scaled	0.09	[0.02, 0.15]	2.59	676.16	.010
News Checking scaled	-0.01	[-0.08, 0.06]	-0.19	675.99	.846
Social Media Use scaled	-0.02	[-0.08, 0.04]	-0.59	675.92	.557
ConditionEvil × Matched controlTRUE	-0.14	[-0.31, 0.03]	-1.65	7894.93	.100
ConditionGood × Matched controlTRUE	-0.42	[-0.59, -0.25]	-4.87	7878.42	$<$ .001
**Random Effects**					
σ^2	2.39				
$\tau_{00\ \text{Participant}}$	0.47				
$\tau_{00\ \text{Item}}$	0.37				
$\text{N}_{\text{Participant}}$	689				
N_{Item}	24				
Marginal R^2	0.247				
Conditional R^2	0.442				

Confidence

Table S32. Multilevel Model for Confidence Ratings (All Items) for Study 1

Term	$\hat{\beta}$	95% CI	t	df	p
Intercept	5.41	[5.14, 5.68]	38.74	193.74	$<$ .001
ConditionEvil	0.09	[-0.09, 0.27]	0.98	898.86	.330
ConditionGood	0.20	[0.02, 0.38]	2.12	901.64	.034
Matched controlTRUE	-0.25	[-0.47, -0.03]	-2.25	27.62	.033
Having Children	-0.24	[-0.41, -0.08]	-2.88	675.99	.004
Gender simpleMale	-0.08	[-0.22, 0.06]	-1.16	676.35	.247
Gender simpleOther	0.15	[-0.35, 0.64]	0.58	675.97	.563
Political Ideology AllLiberal	0.20	[0.02, 0.37]	2.18	676.05	.030
Political Ideology AllConservative	-0.05	[-0.27, 0.17]	-0.41	676.01	.681
COVID Vaccine Intentions	0.20	[0.03, 0.38]	2.29	676.07	.022
Age scaled	0.17	[0.09, 0.26]	4.03	676.21	$<$ .001
Education scaled	0.02	[-0.05, 0.10]	0.62	676.13	.536
News Checking scaled	0.07	[-0.01, 0.15]	1.79	676.06	.074
Social Media Use scaled	0.02	[-0.05, 0.09]	0.51	676.03	.612
ConditionEvil × Matched controlTRUE	-0.04	[-0.16, 0.08]	-0.60	7711.26	.549
ConditionGood × Matched controlTRUE	-0.14	[-0.27, -0.02]	-2.26	7702.73	.024
**Random Effects**					
σ^2	1.24				
$\tau_{00\ \text{Participant}}$	0.73				
$\tau_{00\ \text{Item}}$	0.06				
$\text{N}_{\text{Participant}}$	689				
N_{Item}	24				
Marginal R^2	0.050				
Conditional R^2	0.421				

Sharing Intent

Table S33. Multilevel Model for Sharing Ratings (All Items) for Study 1

Term	$\hat{\beta}$	95% CI	t	df	p
Intercept	1.95	[1.52, 2.37]	8.94	100.40	$<$ .001
ConditionEvil	-0.05	[-0.29, 0.20]	-0.39	843.86	.696
ConditionGood	-0.32	[-0.57, -0.07]	-2.47	845.88	.014
Matched controlTRUE	0.95	[0.54, 1.35]	4.61	24.27	$<$ .001
Having Children	0.49	[0.26, 0.71]	4.18	675.96	$<$ .001
Gender simpleMale	0.57	[0.38, 0.77]	5.78	676.24	$<$ .001
Gender simpleOther	-0.08	[-0.76, 0.61]	-0.22	675.94	.827
Political Ideology AllLiberal	-0.03	[-0.28, 0.21]	-0.27	676.00	.789
Political Ideology AllConservative	0.23	[-0.07, 0.54]	1.49	675.98	.136
COVID Vaccine Intentions	0.03	[-0.21, 0.27]	0.22	676.02	.823
Age scaled	0.03	[-0.08, 0.15]	0.52	676.13	.601
Education scaled	0.17	[0.07, 0.27]	3.30	676.07	.001
News Checking scaled	-0.08	[-0.19, 0.03]	-1.50	676.01	.135
Social Media Use scaled	0.30	[0.20, 0.40]	5.93	675.99	$<$ .001
ConditionEvil × Matched controlTRUE	0.17	[0.02, 0.32]	2.27	7674.73	.023
ConditionGood × Matched controlTRUE	0.55	[0.40, 0.70]	7.18	7668.12	$<$ .001
**Random Effects**					
σ^2	1.81				
$\tau_{00\ \text{Participant}}$	1.45				
$\tau_{00\ \text{Item}}$	0.23				
$N_{\text{Participant}}$	689				
N_{Item}	24				
Marginal R^2	0.158				
Conditional R^2	0.564				

Item-Level Results.

Bartlett test items

Table S34. Item-Level Bartlett Test of Homogeneity of Variances for Study 1: Manipulativeness

Variable	Bartlett's K-squared	df	p
Conspir.Database Manipulativeness	3.480	2	0.176
Conspir.Database.C Manipulativeness	0.611	2	0.737
Conspir.Leaders Manipulativeness	7.754	2	**0.021**
Conspir.Leaders.C Manipulativeness	1.532	2	0.465
Conspir.Greatergood Manipulativeness	11.104	2	**0.004**
Conspir.Greatergood.C Manipulativeness	0.058	2	0.972
Expert.FluVaccine Manipulativeness	1.402	2	0.496
Expert.FluVaccine.C Manipulativeness	0.362	2	0.834
Expert.HPV Manipulativeness	3.478	2	0.176
Expert.HPV.C Manipulativeness	0.383	2	0.826
Expert.Autism Manipulativeness	9.969	2	**0.007**
Expert.Autism.C Manipulativeness	2.371	2	0.306
Natural.Glyphosate Manipulativeness	1.491	2	0.474
Natural.Glyphosate.C Manipulativeness	0.604	2	0.739
Natural.Homeopathy Manipulativeness	4.253	2	0.119
Natural.Homeopathy.C Manipulativeness	1.165	2	0.558
Natural.Viruses Manipulativeness	0.064	2	0.968
Natural.Viruses.C Manipulativeness	0.216	2	0.898
Story.Multiple Manipulativeness	37.815	2	**<0.001**
Story.Multiple.C Manipulativeness	3.285	2	0.193
Story.VaccineCourt Manipulativeness	2.145	2	0.342
Story.VaccineCourt.C Manipulativeness	1.641	2	0.440
Story.Hepatitis Manipulativeness	3.532	2	0.171
Story.Hepatitis.C Manipulativeness	1.390	2	0.499

Table S35. **Item-Level Bartlett Test of Homogeneity of Variances for Study 1: Confidence**

Variable	Bartlett's K-squared	*df*	*p*
Conspir.Database Confidence	0.204	2	0.903
Conspir.Database.C Confidence	0.983	2	0.612
Conspir.Leaders Confidence	0.883	2	0.643
Conspir.Leaders.C Confidence	5.096	2	0.078
Conspir.Greatergood Confidence	4.024	2	0.134
Conspir.Greatergood.C Confidence	3.015	2	0.221
Expert.FluVaccine Confidence	0.779	2	0.677
Expert.FluVaccine.C Confidence	3.267	2	0.195
Expert.HPV Confidence	4.474	2	0.107
Expert.HPV.C Confidence	0.090	2	0.956
Expert.Autism Confidence	1.193	2	0.551
Expert.Autism.C Confidence	3.934	2	0.140
Natural.Glyphosate Confidence	1.854	2	0.396
Natural.Glyphosate.C Confidence	0.272	2	0.873
Natural.Homeopathy Confidence	0.843	2	0.656
Natural.Homeopathy.C Confidence	2.126	2	0.345
Natural.Viruses Confidence	0.649	2	0.723
Natural.Viruses.C Confidence	1.903	2	0.386
Story.Multiple Confidence	0.242	2	0.886
Story.Multiple.C Confidence	0.008	2	0.996
Story.VaccineCourt Confidence	1.546	2	0.462
Story.VaccineCourt.C Confidence	0.334	2	0.846
Story.Hepatitis Confidence	1.656	2	0.437
Story.Hepatitis.C Confidence	11.365	2	**0.003**

Table S36. **Item-Level Bartlett Test of Homogeneity of Variances for Study 1: Sharing**

Variable	Bartlett's K-squared	*df*	*p*
Conspir.Database Sharing	7.198	2	**0.027**
Conspir.Database.C Sharing	2.519	2	0.284
Conspir.Leaders Sharing	2.636	2	0.268
Conspir.Leaders.C Sharing	1.437	2	0.487
Conspir.Greatergood Sharing	0.373	2	0.830
Conspir.Greatergood.C Sharing	0.412	2	0.814
Expert.FluVaccine Sharing	5.160	2	0.076
Expert.FluVaccine.C Sharing	1.256	2	0.534
Expert.HPV Sharing	0.628	2	0.731
Expert.HPV.C Sharing	2.145	2	0.342
Expert.Autism Sharing	1.374	2	0.503
Expert.Autism.C Sharing	0.198	2	0.906
Natural.Glyphosate Sharing	1.383	2	0.501
Natural.Glyphosate.C Sharing	0.848	2	0.654
Natural.Homeopathy Sharing	1.140	2	0.566
Natural.Homeopathy.C Sharing	2.198	2	0.333
Natural.Viruses Sharing	0.008	2	0.996
Natural.Viruses.C Sharing	0.170	2	0.918
Story.Multiple Sharing	6.649	2	**0.036**
Story.Multiple.C Sharing	0.434	2	0.805
Story.VaccineCourt Sharing	0.770	2	0.681
Story.VaccineCourt.C Sharing	0.298	2	0.861
Story.Hepatitis Sharing	3.989	2	0.136
Story.Hepatitis.C Sharing	0.221	2	0.895

One-Way ANOVA items

Table S37. **Item-Level One-Way ANOVA (Welch) for Study 1: Manipulativeness**

Variable	F	df1	df2	p
Conspir.Database Manipulativeness	1.113	2	224.919	0.330
Conspir.Database.C Manipulativeness	0.654	2	225.405	0.521
Conspir.Leaders Manipulativeness	1.385	2	220.015	0.252
Conspir.Leaders.C Manipulativeness	0.413	2	232.067	0.662
Conspir.Greatergood Manipulativeness	3.044	2	216.289	**0.050**
Conspir.Greatergood.C Manipulativeness	0.237	2	227.396	0.789
Expert.FluVaccine Manipulativeness	0.730	2	227.658	0.483
Expert.FluVaccine.C Manipulativeness	3.591	2	226.300	**0.029**
Expert.HPV Manipulativeness	2.517	2	218.557	0.083
Expert.HPV.C Manipulativeness	2.218	2	225.217	0.111
Expert.Autism Manipulativeness	1.682	2	219.893	0.188
Expert.Autism.C Manipulativeness	0.892	2	225.254	0.411
Natural.Glyphosate Manipulativeness	0.241	2	225.650	0.786
Natural.Glyphosate.C Manipulativeness	1.538	2	224.535	0.217
Natural.Homeopathy Manipulativeness	2.884	2	223.300	0.058
Natural.Homeopathy.C Manipulativeness	1.234	2	228.851	0.293
Natural.Viruses Manipulativeness	0.700	2	218.893	0.498
Natural.Viruses.C Manipulativeness	1.386	2	227.902	0.252
Story.Multiple Manipulativeness	9.466	2	218.485	**<0.001**
Story.Multiple.C Manipulativeness	0.663	2	223.543	0.517
Story.VaccineCourt Manipulativeness	4.104	2	229.218	**0.018**
Story.VaccineCourt.C Manipulativeness	1.503	2	221.425	0.225
Story.Hepatitis Manipulativeness	6.631	2	227.423	**0.002**
Story.Hepatitis.C Manipulativeness	1.513	2	227.268	0.222

Table S38. **Item-Level One-Way ANOVA (Welch) for Study 1: Confidence**

Variable	F	df1	df2	p
Conspir.Database Confidence	1.258	2	221.127	0.286
Conspir.Database.C Confidence	0.180	2	225.341	0.836
Conspir.Leaders Confidence	0.529	2	222.763	0.590
Conspir.Leaders.C Confidence	0.508	2	228.902	0.603
Conspir.Greatergood Confidence	0.531	2	219.548	0.589
Conspir.Greatergood.C Confidence	0.608	2	224.881	0.545
Expert.FluVaccine Confidence	0.517	2	228.385	0.597
Expert.FluVaccine.C Confidence	0.071	2	224.497	0.932
Expert.HPV Confidence	0.555	2	226.904	0.575
Expert.HPV.C Confidence	0.677	2	224.055	0.509
Expert.Autism Confidence	0.899	2	226.241	0.408
Expert.Autism.C Confidence	0.504	2	225.638	0.605
Natural.Glyphosate Confidence	0.579	2	229.285	0.561
Natural.Glyphosate.C Confidence	0.457	2	224.804	0.634
Natural.Homeopathy Confidence	1.806	2	224.792	0.167
Natural.Homeopathy.C Confidence	2.747	2	225.603	0.066
Natural.Viruses Confidence	2.626	2	216.414	0.075
Natural.Viruses.C Confidence	0.666	2	226.677	0.515
Story.Multiple Confidence	0.711	2	231.558	0.492
Story.Multiple.C Confidence	0.778	2	223.801	0.460
Story.VaccineCourt Confidence	0.268	2	227.687	0.766
Story.VaccineCourt.C Confidence	0.111	2	221.035	0.895
Story.Hepatitis Confidence	2.372	2	227.950	0.096
Story.Hepatitis.C Confidence	1.774	2	225.096	0.172

Table S39. **Item-Level One-Way ANOVA (Welch) for Study 1: Sharing**

Variable	F	df1	df2	p
Conspir.Database Sharing	4.197	2	225.896	**0.016**
Conspir.Database.C Sharing	0.752	2	222.845	0.472
Conspir.Leaders Sharing	1.449	2	222.397	0.237
Conspir.Leaders.C Sharing	0.028	2	230.523	0.972
Conspir.Greatergood Sharing	0.643	2	222.798	0.527
Conspir.Greatergood.C Sharing	3.478	2	227.202	**0.033**
Expert.FluVaccine Sharing	1.124	2	228.297	0.327
Expert.FluVaccine.C Sharing	1.145	2	226.359	0.320
Expert.HPV Sharing	0.112	2	221.611	0.894
Expert.HPV.C Sharing	4.480	2	221.420	**0.012**
Expert.Autism Sharing	0.542	2	223.618	0.582
Expert.Autism.C Sharing	3.460	2	227.363	**0.033**
Natural.Glyphosate Sharing	1.026	2	229.286	0.360
Natural.Glyphosate.C Sharing	0.036	2	225.143	0.964
Natural.Homeopathy Sharing	0.191	2	225.952	0.826
Natural.Homeopathy.C Sharing	1.682	2	226.673	0.188
Natural.Viruses Sharing	0.214	2	218.901	0.807
Natural.Viruses.C Sharing	0.660	2	228.508	0.518
Story.Multiple Sharing	3.102	2	230.455	**0.047**
Story.Multiple.C Sharing	2.103	2	223.941	0.125
Story.VaccineCourt Sharing	1.058	2	228.486	0.349
Story.VaccineCourt.C Sharing	0.200	2	221.161	0.819
Story.Hepatitis Sharing	1.282	2	227.688	0.279
Story.Hepatitis.C Sharing	1.736	2	226.004	0.179

Study 2.

Bartlett Test of Homogeneity of Variances.

Table S40. **Bartlett Test of Homogeneity of Variances for Study 2**

Variable	Bartlett's K-squared	*df*	*p*
Misinformation Manipulativeness	10.31	2	**0.01**
Non-Misinformation Manipulativeness	0.94	2	0.62
Misinformation Manipulativeness	10.31	2	**0.01**
Manipulativeness Discernment	3.62	2	0.16
Misinformation Confidence	2.66	2	0.27
Non-Misinformation Confidence	1.53	2	0.47
Misinformation Sharing Intent	3.91	2	0.14
Non-Misinformation Sharing Intent	0.40	2	0.82
Sharing Intent Discernment	1.65	2	0.44

Linear Regression Models.

Manipulativess

Table S41. Linear Regression Model for Manipulativeness Ratings (Fake Items Only) for Study 2

Predictor	*b*	95% CI	*t*	*df*	*p*
Intercept	4.86	[4.20, 5.52]	14.44	542	< .001
ConditionEvil	0.35	[0.13, 0.56]	3.18	542	.002
ConditionGood	0.32	[0.11, 0.54]	2.94	542	.003
Having Children	-0.32	[-0.52, -0.12]	-3.15	542	.002
Gender simpleMale	-0.27	[-0.46, -0.09]	-2.92	542	.004
Gender simpleOther	0.23	[-0.32, 0.79]	0.83	542	.409
Political Ideology AllLiberal	0.21	[-0.05, 0.46]	1.60	542	.109
Political Ideology AllConservative	0.03	[-0.27, 0.32]	0.17	542	.865
COVID Vaccine Intentions	0.89	[0.64, 1.14]	7.02	542	< .001
Age	0.08	[0.00, 0.16]	1.86	542	.063
Education	-0.03	[-0.10, 0.04]	-0.80	542	.427
News Checking	0.03	[-0.08, 0.14]	0.56	542	.578
Social Media Use	-0.05	[-0.16, 0.05]	-0.97	542	.330

Table S42. Linear Regression Model for Manipulativeness Ratings (Real Items Only) for Study 2

Predictor	*b*	95% CI	*t*	*df*	*p*
Intercept	3.68	[3.00, 4.35]	10.73	542	< .001
ConditionEvil	0.13	[-0.09, 0.35]	1.20	542	.232
ConditionGood	-0.03	[-0.25, 0.19]	-0.23	542	.815
Having Children	0.20	[0.00, 0.41]	1.94	542	.053
Gender simpleMale	0.11	[-0.08, 0.30]	1.14	542	.253
Gender simpleOther	-0.30	[-0.86, 0.27]	-1.03	542	.305
Political Ideology AllLiberal	-0.20	[-0.45, 0.06]	-1.48	542	.139
Political Ideology AllConservative	0.03	[-0.27, 0.34]	0.20	542	.842
COVID Vaccine Intentions	-0.07	[-0.32, 0.18]	-0.53	542	.593
Age	-0.04	[-0.13, 0.05]	-0.91	542	.361
Education	0.00	[-0.07, 0.08]	0.13	542	.894
News Checking	-0.15	[-0.27, -0.04]	-2.70	542	.007
Social Media Use	0.00	[-0.11, 0.11]	0.01	542	.992

Table S43. Linear Regression Model for Manipulativeness Ratings (Difference between Fake and Real) for Study 2

Predictor	*b*	95% CI	*t*	*df*	*p*
Intercept	1.18	[0.26, 2.10]	2.52	542	.012
ConditionEvil	0.21	[-0.08, 0.51]	1.41	542	.160
ConditionGood	0.35	[0.05, 0.65]	2.28	542	.023
Having Children	-0.52	[-0.80, -0.24]	-3.68	542	< .001
Gender simpleMale	-0.38	[-0.64, -0.13]	-2.94	542	.003
Gender simpleOther	0.53	[-0.24, 1.30]	1.34	542	.179
Political Ideology AllLiberal	0.40	[0.05, 0.76]	2.24	542	.026
Political Ideology AllConservative	-0.01	[-0.42, 0.41]	-0.02	542	.981
COVID Vaccine Intentions	0.96	[0.61, 1.31]	5.43	542	< .001
Age	0.12	[0.00, 0.24]	2.01	542	.045
Education	-0.03	[-0.13, 0.07]	-0.67	542	.504
News Checking	0.18	[0.03, 0.34]	2.38	542	.018
Social Media Use	-0.05	[-0.21, 0.10]	-0.71	542	.480

Confidence

Table S44. Linear Regression Model for Confidence Ratings (Fake Items Only) for Study 2

Predictor	b	95% CI	t	df	p
Intercept	5.05	[4.45, 5.65]	16.47	542	$<$ .001
ConditionEvil	0.25	[0.06, 0.45]	2.53	542	.012
ConditionGood	0.17	[-0.03, 0.37]	1.69	542	.092
Having Children	-0.10	[-0.29, 0.08]	-1.13	542	.260
Gender simpleMale	-0.09	[-0.25, 0.08]	-1.00	542	.315
Gender simpleOther	0.20	[-0.31, 0.70]	0.76	542	.448
Political Ideology AllLiberal	0.12	[-0.11, 0.35]	1.00	542	.320
Political Ideology AllConservative	-0.16	[-0.43, 0.11]	-1.14	542	.256
COVID Vaccine Intentions	0.37	[0.14, 0.60]	3.20	542	.001
Age	0.04	[-0.04, 0.11]	0.96	542	.339
Education	-0.02	[-0.08, 0.05]	-0.54	542	.588
News Checking	0.13	[0.03, 0.23]	2.53	542	.012
Social Media Use	-0.02	[-0.12, 0.08]	-0.41	542	.680

Table S45. Linear Regression Model for Confidence Ratings (Real Items Only) for Study 2

Predictor	b	95% CI	t	df	p
Intercept	5.20	[4.57, 5.82]	16.31	542	$<$ .001
ConditionEvil	-0.17	[-0.37, 0.03]	-1.63	542	.104
ConditionGood	-0.11	[-0.31, 0.10]	-1.02	542	.306
Having Children	0.07	[-0.12, 0.26]	0.76	542	.450
Gender simpleMale	-0.09	[-0.27, 0.08]	-1.05	542	.294
Gender simpleOther	0.60	[0.07, 1.12]	2.23	542	.026
Political Ideology AllLiberal	0.09	[-0.15, 0.33]	0.74	542	.461
Political Ideology AllConservative	-0.25	[-0.54, 0.03]	-1.77	542	.078
COVID Vaccine Intentions	-0.14	[-0.37, 0.10]	-1.15	542	.251
Age	0.06	[-0.02, 0.14]	1.48	542	.139
Education	0.04	[-0.03, 0.11]	1.16	542	.248
News Checking	0.02	[-0.08, 0.12]	0.36	542	.720
Social Media Use	0.03	[-0.07, 0.13]	0.58	542	.563

Sharing Intent

Table S46. **Linear Regression Model for Sharing Ratings (Fake Items Only) for Study 2**

Predictor	*b*	95% CI	*t*	*df*	*p*
Intercept	1.72	[0.99, 2.45]	4.65	542	$<$ .001
ConditionEvil	-0.17	[-0.41, 0.07]	-1.42	542	.157
ConditionGood	-0.15	[-0.39, 0.09]	-1.24	542	.215
Having Children	0.42	[0.19, 0.64]	3.70	542	$<$ .001
Gender simpleMale	0.40	[0.20, 0.61]	3.93	542	$<$ .001
Gender simpleOther	-0.14	[-0.75, 0.47]	-0.45	542	.650
Political Ideology AllLiberal	0.01	[-0.27, 0.29]	0.08	542	.937
Political Ideology AllConservative	0.14	[-0.19, 0.47]	0.84	542	.399
COVID Vaccine Intentions	-0.70	[-0.97, -0.42]	-5.00	542	$<$ .001
Age	-0.08	[-0.17, 0.01]	-1.68	542	.094
Education	-0.01	[-0.09, 0.07]	-0.23	542	.822
News Checking	0.07	[-0.05, 0.19]	1.20	542	.231
Social Media Use	0.09	[-0.03, 0.21]	1.43	542	.154

Table S47. **Linear Regression Model for Sharing Ratings (Real Items Only) for Study 2**

Predictor	*b*	95% CI	*t*	*df*	*p*
Intercept	0.87	[-0.03, 1.78]	1.90	542	.058
ConditionEvil	-0.09	[-0.38, 0.20]	-0.60	542	.551
ConditionGood	0.04	[-0.26, 0.34]	0.26	542	.798
Having Children	0.33	[0.06, 0.61]	2.38	542	.018
Gender simpleMale	0.37	[0.12, 0.62]	2.88	542	.004
Gender simpleOther	0.87	[0.11, 1.63]	2.24	542	.026
Political Ideology AllLiberal	0.07	[-0.28, 0.41]	0.37	542	.714
Political Ideology AllConservative	-0.04	[-0.45, 0.37]	-0.18	542	.858
COVID Vaccine Intentions	0.27	[-0.07, 0.61]	1.57	542	.118
Age	0.02	[-0.10, 0.13]	0.26	542	.792
Education	0.00	[-0.09, 0.10]	0.07	542	.941
News Checking	0.20	[0.05, 0.35]	2.63	542	.009
Social Media Use	0.26	[0.11, 0.41]	3.41	542	$<$ .001

Table S48. **Linear Regression Model for Sharing Ratings (Difference between Fake and Real) for Study 2**

Predictor	*b*	95% CI	*t*	*df*	*p*
Intercept	-0.85	[-1.72, 0.02]	-1.92	542	.056
ConditionEvil	0.08	[-0.20, 0.36]	0.56	542	.573
ConditionGood	0.19	[-0.10, 0.47]	1.31	542	.192
Having Children	-0.08	[-0.35, 0.18]	-0.61	542	.540
Gender simpleMale	-0.03	[-0.28, 0.21]	-0.28	542	.777
Gender simpleOther	1.01	[0.28, 1.74]	2.72	542	.007
Political Ideology AllLiberal	0.05	[-0.28, 0.39]	0.32	542	.752
Political Ideology AllConservative	-0.18	[-0.57, 0.21]	-0.89	542	.372
COVID Vaccine Intentions	0.97	[0.64, 1.30]	5.82	542	$<$ .001
Age	0.09	[-0.02, 0.21]	1.68	542	.093
Education	0.01	[-0.08, 0.11]	0.27	542	.790
News Checking	0.13	[-0.02, 0.27]	1.73	542	.084
Social Media Use	0.17	[0.03, 0.31]	2.36	542	.019

Multilevel Models.

Manipulativess

Table S49. Multilevel Model for Manipulativeness Ratings (All Items) for Study 2

Term	$\hat{\beta}$	95% CI	t	df	p
Intercept	5.15	[4.64, 5.67]	19.58	37.77	$< .001$
ConditionEvil	0.38	[0.19, 0.57]	4.01	1044.79	$< .001$
ConditionGood	0.41	[0.22, 0.59]	4.32	1000.35	$< .001$
Matched controlTRUE	-2.33	[-2.95, -1.70]	-7.35	23.27	$< .001$
Having Children	-0.07	[-0.22, 0.07]	-0.97	542.13	.331
Gender simpleMale	-0.08	[-0.21, 0.06]	-1.15	541.83	.252
Gender simpleOther	0.07	[-0.34, 0.47]	0.32	542.09	.751
Political Ideology AllLiberal	0.00	[-0.19, 0.18]	-0.01	542.37	.992
Political Ideology AllConservative	0.04	[-0.17, 0.26]	0.39	542.08	.695
COVID Vaccine Intentions	0.40	[0.22, 0.58]	4.30	541.97	$< .001$
Age scaled	0.04	[-0.03, 0.12]	1.17	541.96	.243
Education scaled	-0.01	[-0.08, 0.06]	-0.36	541.68	.716
News Checking scaled	-0.06	[-0.13, 0.01]	-1.73	542.00	.085
Social Media Use scaled	0.00	[-0.07, 0.06]	-0.08	543.23	.933
ConditionEvil × Matched controlTRUE	-0.15	[-0.33, 0.04]	-1.55	6360.18	.121
ConditionGood × Matched controlTRUE	-0.37	[-0.56, -0.19]	-3.97	6358.60	$< .001$
**Random Effects**					
σ^2	2.32				
$\tau_{00\ \text{Participant}}$	0.40				
$\tau_{00\ \text{Item}}$	0.57				
$\text{N}_{\text{Participant}}$	555				
N_{Item}	24				
Marginal R^2	0.327				
Conditional R^2	0.525				

Confidence

Table S50. Multilevel Model for Confidence Ratings (All Items) for Study 2

Term	$\hat{\beta}$	95% CI	t	df	p
Intercept	5.63	[5.30, 5.96]	33.49	203.56	$< .001$
ConditionEvil	0.25	[0.06, 0.44]	2.57	751.78	.010
ConditionGood	0.18	[-0.01, 0.37]	1.90	733.93	.058
Matched controlTRUE	-0.12	[-0.38, 0.14]	-0.92	26.41	.364
Having Children	0.00	[-0.16, 0.16]	0.00	542.14	.997
Gender simpleMale	-0.09	[-0.24, 0.06]	-1.22	542.01	.224
Gender simpleOther	0.39	[-0.05, 0.84]	1.73	542.12	.085
Political Ideology AllLiberal	0.12	[-0.09, 0.32]	1.11	542.24	.267
Political Ideology AllConservative	-0.19	[-0.43, 0.05]	-1.57	542.11	.117
COVID Vaccine Intentions	0.12	[-0.08, 0.32]	1.21	542.07	.228
Age scaled	0.06	[-0.03, 0.14]	1.34	542.06	.180
Education scaled	0.02	[-0.06, 0.09]	0.44	541.94	.658
News Checking scaled	0.07	[-0.01, 0.15]	1.77	542.08	.078
Social Media Use scaled	0.01	[-0.06, 0.09]	0.30	542.61	.763
ConditionEvil × Matched controlTRUE	-0.36	[-0.49, -0.23]	-5.24	6214.56	$< .001$
ConditionGood × Matched controlTRUE	-0.25	[-0.39, -0.12]	-3.67	6213.73	$< .001$
**Random Effects**					
σ^2	1.23				
$\tau_{00\ \text{Participant}}$	0.62				
$\tau_{00\ \text{Item}}$	0.09				
$\text{N}_{\text{Participant}}$	555				
N_{Item}	24				
Marginal R^2	0.037				
Conditional R^2	0.389				

Sharing Intent

Table S51. Multilevel Model for Sharing Ratings (All Items) for Study 2

Term	$\hat{\beta}$	95% CI	t	df	p
Intercept	1.71	[1.21, 2.21]	6.67	90.98	$<$ .001
ConditionEvil	-0.13	[-0.38, 0.11]	-1.07	724.34	.285
ConditionGood	-0.16	[-0.41, 0.08]	-1.30	708.91	.193
Matched controlTRUE	1.32	[0.83, 1.80]	5.35	23.72	$<$ .001
Having Children	0.38	[0.16, 0.59]	3.48	542.00	$<$ .001
Gender simpleMale	0.42	[0.22, 0.61]	4.22	541.88	$<$ .001
Gender simpleOther	0.25	[-0.34, 0.83]	0.83	541.98	.407
Political Ideology AllLiberal	0.04	[-0.23, 0.31]	0.30	542.09	.768
Political Ideology AllConservative	0.01	[-0.31, 0.32]	0.04	541.98	.968
COVID Vaccine Intentions	-0.19	[-0.45, 0.07]	-1.43	541.94	.154
Age scaled	-0.03	[-0.14, 0.07]	-0.62	541.93	.535
Education scaled	-0.01	[-0.11, 0.09]	-0.18	541.82	.854
News Checking scaled	0.13	[0.02, 0.23]	2.37	541.95	.018
Social Media Use scaled	0.14	[0.04, 0.23]	2.75	542.43	.006
ConditionEvil × Matched controlTRUE	-0.01	[-0.17, 0.16]	-0.08	6198.22	.934
ConditionGood × Matched controlTRUE	0.12	[-0.04, 0.29]	1.48	6197.45	.138
**Random Effects**					
σ^2	1.84				
$\tau_{00\ \text{Participant}}$	1.09				
$\tau_{00\ \text{Item}}$	0.34				
$\text{N}_{\text{Participant}}$	555				
N_{Item}	24				
Marginal R^2	0.150				
Conditional R^2	0.521				

Item-Level Results.

Bartlett test items

Table S52. Item-Level Bartlett Test of Homogeneity of Variances for Study 2: Manipulativeness

Variable	Bartlett's K-squared	df	p
Conspir.Database Manipulativeness	13.279	2	**0.001**
Conspir.Database.C Manipulativeness	0.017	2	0.991
Conspir.Leaders Manipulativeness	7.339	2	**0.025**
Conspir.Leaders.C Manipulativeness	0.105	2	0.949
Conspir.Greatergood Manipulativeness	6.722	2	**0.035**
Conspir.Greatergood.C Manipulativeness	1.652	2	0.438
Expert.FluVaccine Manipulativeness	1.776	2	0.412
Expert.FluVaccine.C Manipulativeness	1.101	2	0.577
Expert.HPV Manipulativeness	3.452	2	0.178
Expert.HPV.C Manipulativeness	3.741	2	0.154
Expert.Autism Manipulativeness	7.708	2	**0.021**
Expert.Autism.C Manipulativeness	0.065	2	0.968
Natural.Glyphosate Manipulativeness	9.626	2	**0.008**
Natural.Glyphosate.C Manipulativeness	0.202	2	0.904
Natural.Homeopathy Manipulativeness	6.598	2	**0.037**
Natural.Homeopathy.C Manipulativeness	2.436	2	0.296
Natural.Viruses Manipulativeness	0.314	2	0.855
Natural.Viruses.C Manipulativeness	0.605	2	0.739
Story.Multiple Manipulativeness	3.052	2	0.217
Story.Multiple.C Manipulativeness	0.072	2	0.964
Story.VaccineCourt Manipulativeness	2.492	2	0.288
Story.VaccineCourt.C Manipulativeness	1.599	2	0.450
Story.Hepatitis Manipulativeness	7.860	2	**0.020**
Story.Hepatitis.C Manipulativeness	22.459	2	**<0.001**

Table S53. **Item-Level Bartlett Test of Homogeneity of Variances for Study 2: Confidence**

Variable	Bartlett's K-squared	*df*	*p*
Conspir.Database Confidence	6.273	2	**0.043**
Conspir.Database.C Confidence	1.162	2	0.559
Conspir.Leaders Confidence	12.450	2	**0.002**
Conspir.Leaders.C Confidence	1.717	2	0.424
Conspir.Greatergood Confidence	2.107	2	0.349
Conspir.Greatergood.C Confidence	3.109	2	0.211
Expert.FluVaccine Confidence	1.335	2	0.513
Expert.FluVaccine.C Confidence	0.547	2	0.761
Expert.HPV Confidence	4.331	2	0.115
Expert.HPV.C Confidence	17.894	2	**<0.001**
Expert.Autism Confidence	1.859	2	0.395
Expert.Autism.C Confidence	2.725	2	0.256
Natural.Glyphosate Confidence	7.411	2	**0.025**
Natural.Glyphosate.C Confidence	0.945	2	0.624
Natural.Homeopathy Confidence	1.026	2	0.599
Natural.Homeopathy.C Confidence	1.082	2	0.582
Natural.Viruses Confidence	2.787	2	0.248
Natural.Viruses.C Confidence	2.721	2	0.256
Story.Multiple Confidence	0.465	2	0.793
Story.Multiple.C Confidence	1.899	2	0.387
Story.VaccineCourt Confidence	2.744	2	0.254
Story.VaccineCourt.C Confidence	2.250	2	0.325
Story.Hepatitis Confidence	0.904	2	0.636
Story.Hepatitis.C Confidence	21.679	2	**<0.001**

Table S54. **Item-Level Bartlett Test of Homogeneity of Variances for Study 2: Sharing**

Variable	Bartlett's K-squared	*df*	*p*
Conspir.Database Sharing	8.083	2	**0.018**
Conspir.Database.C Sharing	0.445	2	0.801
Conspir.Leaders Sharing	1.429	2	0.490
Conspir.Leaders.C Sharing	0.030	2	0.985
Conspir.Greatergood Sharing	7.347	2	**0.025**
Conspir.Greatergood.C Sharing	2.036	2	0.361
Expert.FluVaccine Sharing	1.272	2	0.529
Expert.FluVaccine.C Sharing	0.125	2	0.939
Expert.HPV Sharing	7.961	2	**0.019**
Expert.HPV.C Sharing	0.289	2	0.865
Expert.Autism Sharing	4.447	2	0.108
Expert.Autism.C Sharing	0.239	2	0.887
Natural.Glyphosate Sharing	2.291	2	0.318
Natural.Glyphosate.C Sharing	0.648	2	0.723
Natural.Homeopathy Sharing	7.535	2	**0.023**
Natural.Homeopathy.C Sharing	0.694	2	0.707
Natural.Viruses Sharing	2.623	2	0.269
Natural.Viruses.C Sharing	2.345	2	0.310
Story.Multiple Sharing	9.543	2	**0.008**
Story.Multiple.C Sharing	0.158	2	0.924
Story.VaccineCourt Sharing	8.611	2	**0.013**
Story.VaccineCourt.C Sharing	0.509	2	0.775
Story.Hepatitis Sharing	11.482	2	**0.003**
Story.Hepatitis.C Sharing	3.079	2	0.214

One-Way ANOVA items

Table S55. Item-Level One-Way ANOVA (Welch) for Study 2: Manipulativeness

Variable	F	df1	df2	p
Conspir.Database Manipulativeness	3.877	2	177.650	**0.022**
Conspir.Database.C Manipulativeness	2.480	2	183.030	0.087
Conspir.Leaders Manipulativeness	0.841	2	184.971	0.433
Conspir.Leaders.C Manipulativeness	0.077	2	181.865	0.926
Conspir.Greatergood Manipulativeness	0.573	2	180.072	0.565
Conspir.Greatergood.C Manipulativeness	1.491	2	185.189	0.228
Expert.FluVaccine Manipulativeness	0.360	2	179.807	0.698
Expert.FluVaccine.C Manipulativeness	0.120	2	181.665	0.887
Expert.HPV Manipulativeness	5.873	2	179.040	**0.003**
Expert.HPV.C Manipulativeness	2.781	2	186.093	0.065
Expert.Autism Manipulativeness	0.907	2	182.756	0.406
Expert.Autism.C Manipulativeness	0.001	2	181.941	0.999
Natural.Glyphosate Manipulativeness	4.650	2	185.785	**0.011**
Natural.Glyphosate.C Manipulativeness	1.342	2	180.405	0.264
Natural.Homeopathy Manipulativeness	8.127	2	182.321	**<0.001**
Natural.Homeopathy.C Manipulativeness	0.773	2	177.343	0.463
Natural.Viruses Manipulativeness	2.731	2	184.693	0.068
Natural.Viruses.C Manipulativeness	3.547	2	178.298	**0.031**
Story.Multiple Manipulativeness	2.733	2	174.798	0.068
Story.Multiple.C Manipulativeness	0.046	2	172.809	0.955
Story.VaccineCourt Manipulativeness	1.542	2	177.236	0.217
Story.VaccineCourt.C Manipulativeness	0.311	2	183.124	0.733
Story.Hepatitis Manipulativeness	5.735	2	175.941	**0.004**
Story.Hepatitis.C Manipulativeness	10.216	2	185.190	**<0.001**

Table S56. Item-Level One-Way ANOVA (Welch) for Study 2: Confidence

Variable	F	df1	df2	p
Conspir.Database Confidence	2.328	2	181.925	0.100
Conspir.Database.C Confidence	0.344	2	181.809	0.709
Conspir.Leaders Confidence	1.675	2	184.865	0.190
Conspir.Leaders.C Confidence	0.111	2	181.283	0.895
Conspir.Greatergood Confidence	0.006	2	177.326	0.994
Conspir.Greatergood.C Confidence	0.430	2	185.897	0.651
Expert.FluVaccine Confidence	0.628	2	181.839	0.535
Expert.FluVaccine.C Confidence	0.171	2	178.767	0.843
Expert.HPV Confidence	1.030	2	178.738	0.359
Expert.HPV.C Confidence	2.091	2	181.054	0.127
Expert.Autism Confidence	0.574	2	183.933	0.564
Expert.Autism.C Confidence	0.107	2	181.136	0.899
Natural.Glyphosate Confidence	5.569	2	185.757	**0.004**
Natural.Glyphosate.C Confidence	1.398	2	181.309	0.250
Natural.Homeopathy Confidence	1.407	2	184.129	0.247
Natural.Homeopathy.C Confidence	0.133	2	177.579	0.875
Natural.Viruses Confidence	1.737	2	181.467	0.179
Natural.Viruses.C Confidence	0.867	2	178.545	0.422
Story.Multiple Confidence	3.142	2	178.986	**0.046**
Story.Multiple.C Confidence	2.092	2	169.389	0.127
Story.VaccineCourt Confidence	3.813	2	181.635	**0.024**
Story.VaccineCourt.C Confidence	0.889	2	184.663	0.413
Story.Hepatitis Confidence	2.196	2	171.538	0.114
Story.Hepatitis.C Confidence	3.238	2	176.832	**0.042**

Table S57. **Item-Level One-Way ANOVA (Welch) for Study 2: Sharing**

Variable	F	df1	df2	p
Conspir.Database Sharing	1.423	2	181.317	0.244
Conspir.Database.C Sharing	0.304	2	182.319	0.738
Conspir.Leaders Sharing	0.003	2	180.010	0.997
Conspir.Leaders.C Sharing	0.593	2	181.866	0.554
Conspir.Greatergood Sharing	0.113	2	179.147	0.893
Conspir.Greatergood.C Sharing	0.924	2	186.577	0.399
Expert.FluVaccine Sharing	0.252	2	181.222	0.777
Expert.FluVaccine.C Sharing	0.124	2	179.645	0.884
Expert.HPV Sharing	1.926	2	178.812	0.149
Expert.HPV.C Sharing	0.459	2	187.278	0.632
Expert.Autism Sharing	1.238	2	179.812	0.292
Expert.Autism.C Sharing	0.478	2	181.654	0.621
Natural.Glyphosate Sharing	1.319	2	185.533	0.270
Natural.Glyphosate.C Sharing	0.005	2	180.611	0.995
Natural.Homeopathy Sharing	3.324	2	182.072	**0.038**
Natural.Homeopathy.C Sharing	0.098	2	177.940	0.907
Natural.Viruses Sharing	1.557	2	184.782	0.213
Natural.Viruses.C Sharing	2.495	2	179.287	0.085
Story.Multiple Sharing	3.050	2	169.592	**0.050**
Story.Multiple.C Sharing	0.638	2	173.715	0.530
Story.VaccineCourt Sharing	1.202	2	177.544	0.303
Story.VaccineCourt.C Sharing	0.409	2	183.209	0.665
Story.Hepatitis Sharing	2.391	2	174.318	0.095
Story.Hepatitis.C Sharing	1.448	2	179.916	0.238

Study 3.

Bartlett Test of Homogeneity of Variances.

Table S58. **Bartlett Test of Homogeneity of Variances for Study 3**

Variable	Bartlett's K-squared	*df*	*p*
Misinformation Manipulativeness	12.59	2	**0.00**
Non-Misinformation Manipulativeness	4.70	2	0.10
Misinformation Manipulativeness	12.59	2	**0.00**
Manipulativeness Discernment	6.13	2	**0.05**
Misinformation Confidence	5.92	2	0.05
Non-Misinformation Confidence	3.93	2	0.14
Misinformation Sharing Intent	15.31	2	**<0.001**
Non-Misinformation Sharing Intent	0.08	2	0.96
Sharing Intent Discernment	6.60	2	**0.04**

Linear Regression Models.

Manipulativess

Table S59. Linear Regression Model for Manipulativeness Ratings (Fake Items Only) for Study 3

Predictor	*b*	95% CI	*t*	*df*	*p*
Intercept	3.77	[3.36, 4.18]	17.98	1062	< .001
ConditionEvil	0.43	[0.29, 0.58]	5.80	1062	< .001
ConditionGood	0.33	[0.18, 0.47]	4.45	1062	< .001
Having Children	-0.06	[-0.21, 0.08]	-0.88	1062	.379
Gender simpleMale	0.06	[-0.06, 0.19]	1.00	1062	.318
Gender simpleOther	0.00	[-0.42, 0.42]	-0.01	1062	.994
Political Ideology AllLiberal	0.49	[0.33, 0.64]	6.29	1062	< .001
Political Ideology AllConservative	0.09	[-0.11, 0.28]	0.87	1062	.382
COVID Vaccine Intentions	0.84	[0.66, 1.02]	9.07	1062	< .001
Age	0.05	[-0.01, 0.10]	1.74	1062	.082
Education	-0.01	[-0.06, 0.03]	-0.61	1062	.542
News Checking	0.13	[0.06, 0.20]	3.76	1062	< .001
Social Media Use	0.01	[-0.06, 0.07]	0.23	1062	.818

Table S60. Linear Regression Model for Manipulativeness Ratings (Real Items Only) for Study 3

Predictor	*b*	95% CI	*t*	*df*	*p*
Intercept	3.61	[3.17, 4.05]	16.11	1062	< .001
ConditionEvil	0.10	[-0.06, 0.25]	1.24	1062	.214
ConditionGood	-0.13	[-0.28, 0.02]	-1.65	1062	.098
Having Children	0.07	[-0.08, 0.23]	0.90	1062	.366
Gender simpleMale	0.05	[-0.09, 0.18]	0.67	1062	.501
Gender simpleOther	-0.14	[-0.58, 0.31]	-0.61	1062	.542
Political Ideology AllLiberal	-0.15	[-0.31, 0.02]	-1.76	1062	.079
Political Ideology AllConservative	0.01	[-0.20, 0.22]	0.10	1062	.924
COVID Vaccine Intentions	-0.20	[-0.39, 0.00]	-2.01	1062	.045
Age	0.00	[-0.06, 0.06]	-0.11	1062	.910
Education	0.00	[-0.05, 0.05]	0.13	1062	.899
News Checking	-0.05	[-0.12, 0.02]	-1.34	1062	.181
Social Media Use	0.00	[-0.07, 0.06]	-0.13	1062	.897

Table S61. Linear Regression Model for Manipulativeness Ratings (Difference between Fake and Real) for Study 3

Predictor	*b*	95% CI	*t*	*df*	*p*
Intercept	0.16	[-0.41, 0.73]	0.56	1062	.577
ConditionEvil	0.33	[0.13, 0.53]	3.23	1062	.001
ConditionGood	0.46	[0.26, 0.66]	4.49	1062	< .001
Having Children	-0.14	[-0.34, 0.06]	-1.33	1062	.183
Gender simpleMale	0.02	[-0.16, 0.19]	0.20	1062	.840
Gender simpleOther	0.14	[-0.44, 0.72]	0.47	1062	.642
Political Ideology AllLiberal	0.63	[0.42, 0.84]	5.90	1062	< .001
Political Ideology AllConservative	0.08	[-0.20, 0.35]	0.56	1062	.577
COVID Vaccine Intentions	1.04	[0.79, 1.29]	8.10	1062	< .001
Age	0.05	[-0.02, 0.13]	1.35	1062	.179
Education	-0.02	[-0.08, 0.05]	-0.54	1062	.590
News Checking	0.18	[0.09, 0.28]	3.74	1062	< .001
Social Media Use	0.01	[-0.08, 0.10]	0.27	1062	.790

Confidence

Table S62. Linear Regression Model for Confidence Ratings (Fake Items Only) for Study 3

Predictor	b	95% CI	t	df	p
Intercept	4.66	[4.26, 5.06]	22.87	1062	$<$ .001
ConditionEvil	0.21	[0.07, 0.35]	2.93	1062	.003
ConditionGood	0.20	[0.05, 0.34]	2.73	1062	.006
Having Children	-0.04	[-0.18, 0.10]	-0.52	1062	.603
Gender simpleMale	-0.05	[-0.17, 0.07]	-0.76	1062	.448
Gender simpleOther	0.24	[-0.17, 0.64]	1.14	1062	.253
Political Ideology AllLiberal	0.31	[0.16, 0.46]	4.11	1062	$<$ .001
Political Ideology AllConservative	0.09	[-0.11, 0.28]	0.88	1062	.379
COVID Vaccine Intentions	0.43	[0.26, 0.61]	4.82	1062	$<$ .001
Age	0.05	[0.00, 0.10]	1.79	1062	.074
Education	-0.04	[-0.09, 0.00]	-1.84	1062	.066
News Checking	0.11	[0.04, 0.18]	3.21	1062	.001
Social Media Use	0.03	[-0.03, 0.10]	1.03	1062	.303

Table S63. Linear Regression Model for Confidence Ratings (Real Items Only) for Study 3

Predictor	b	95% CI	t	df	p
Intercept	4.59	[4.16, 5.02]	20.77	1062	$<$ .001
ConditionEvil	-0.03	[-0.18, 0.13]	-0.33	1062	.741
ConditionGood	0.07	[-0.09, 0.22]	0.86	1062	.389
Having Children	-0.01	[-0.16, 0.15]	-0.07	1062	.946
Gender simpleMale	-0.05	[-0.18, 0.08]	-0.71	1062	.477
Gender simpleOther	0.24	[-0.20, 0.68]	1.05	1062	.294
Political Ideology AllLiberal	0.36	[0.20, 0.52]	4.37	1062	$<$ .001
Political Ideology AllConservative	0.21	[0.01, 0.42]	2.03	1062	.042
COVID Vaccine Intentions	0.10	[-0.09, 0.29]	1.00	1062	.317
Age	0.04	[-0.01, 0.10]	1.47	1062	.141
Education	-0.03	[-0.08, 0.01]	-1.41	1062	.159
News Checking	0.14	[0.07, 0.21]	3.79	1062	$<$ .001
Social Media Use	0.01	[-0.06, 0.08]	0.37	1062	.714

Sharing Intent

Table S64. Linear Regression Model for Sharing Ratings (Fake Items Only) for Study 3

Predictor	*b*	95% CI	*t*	*df*	*p*
Intercept	2.62	[2.10, 3.14]	9.94	1062	< .001
ConditionEvil	0.07	[-0.11, 0.26]	0.78	1062	.436
ConditionGood	-0.06	[-0.24, 0.13]	-0.61	1062	.541
Having Children	0.25	[0.07, 0.43]	2.70	1062	.007
Gender simpleMale	0.08	[-0.07, 0.24]	1.04	1062	.300
Gender simpleOther	-0.31	[-0.84, 0.21]	-1.17	1062	.243
Political Ideology AllLiberal	-0.51	[-0.70, -0.32]	-5.29	1062	< .001
Political Ideology AllConservative	-0.05	[-0.30, 0.19]	-0.43	1062	.665
COVID Vaccine Intentions	-0.54	[-0.76, -0.31]	-4.60	1062	< .001
Age	0.00	[-0.07, 0.07]	-0.08	1062	.938
Education	-0.02	[-0.08, 0.04]	-0.66	1062	.512
News Checking	-0.03	[-0.12, 0.05]	-0.79	1062	.432
Social Media Use	0.05	[-0.03, 0.13]	1.12	1062	.265

Table S65. Linear Regression Model for Sharing Ratings (Real Items Only) for Study 3

Predictor	*b*	95% CI	*t*	*df*	*p*
Intercept	1.67	[1.07, 2.26]	5.47	1062	< .001
ConditionEvil	0.29	[0.08, 0.50]	2.68	1062	.007
ConditionGood	0.50	[0.29, 0.71]	4.71	1062	< .001
Having Children	0.08	[-0.13, 0.29]	0.78	1062	.433
Gender simpleMale	0.11	[-0.07, 0.29]	1.20	1062	.229
Gender simpleOther	0.55	[-0.06, 1.16]	1.78	1062	.076
Political Ideology AllLiberal	0.00	[-0.22, 0.22]	-0.03	1062	.975
Political Ideology AllConservative	-0.16	[-0.45, 0.12]	-1.13	1062	.258
COVID Vaccine Intentions	0.19	[-0.08, 0.45]	1.40	1062	.161
Age	0.04	[-0.04, 0.12]	0.99	1062	.323
Education	0.01	[-0.06, 0.08]	0.31	1062	.757
News Checking	0.21	[0.11, 0.31]	4.11	1062	< .001
Social Media Use	0.05	[-0.05, 0.14]	0.98	1062	.330

Table S66. Linear Regression Model for Sharing Ratings (Difference between Fake and Real) for Study 3

Predictor	*b*	95% CI	*t*	*df*	*p*
Intercept	-0.96	[-1.50, -0.41]	-3.43	1062	< .001
ConditionEvil	0.22	[0.02, 0.41]	2.19	1062	.028
ConditionGood	0.56	[0.37, 0.75]	5.72	1062	< .001
Having Children	-0.17	[-0.36, 0.03]	-1.70	1062	.090
Gender simpleMale	0.03	[-0.14, 0.19]	0.33	1062	.739
Gender simpleOther	0.86	[0.31, 1.42]	3.05	1062	.002
Political Ideology AllLiberal	0.51	[0.31, 0.71]	4.97	1062	< .001
Political Ideology AllConservative	-0.11	[-0.37, 0.15]	-0.83	1062	.409
COVID Vaccine Intentions	0.72	[0.48, 0.97]	5.89	1062	< .001
Age	0.04	[-0.03, 0.12]	1.15	1062	.249
Education	0.03	[-0.03, 0.09]	0.96	1062	.337
News Checking	0.24	[0.15, 0.34]	5.23	1062	< .001
Social Media Use	0.00	[-0.09, 0.09]	0.01	1062	.993

Multilevel Models.

Manipulativess

Table S67. **Multilevel Model for Manipulativeness Ratings (All Items) for Study 3**

Term	$\hat{\beta}$	95% CI	t	df	p
Intercept	5.04	[4.58, 5.50]	21.55	30.60	< .001
ConditionEvil	0.41	[0.28, 0.54]	6.04	2050.90	< .001
ConditionGood	0.34	[0.21, 0.48]	5.14	2022.71	< .001
Matched controlTRUE	-2.00	[-2.57, -1.42]	-6.77	22.72	< .001
Having Children	-0.01	[-0.12, 0.10]	-0.18	1061.36	.855
Gender simpleMale	0.03	[-0.06, 0.13]	0.65	1061.08	.514
Gender simpleOther	0.00	[-0.32, 0.32]	0.03	1061.95	.979
Political Ideology AllLiberal	0.15	[0.03, 0.26]	2.46	1062.30	.014
Political Ideology AllConservative	0.00	[-0.15, 0.15]	0.04	1062.04	.969
COVID Vaccine Intentions	0.26	[0.12, 0.40]	3.64	1061.30	< .001
Age scaled	0.02	[-0.04, 0.08]	0.65	1061.41	.516
Education scaled	0.01	[-0.04, 0.06]	0.42	1061.77	.675
News Checking scaled	0.06	[0.01, 0.11]	2.24	1061.78	.025
Social Media Use scaled	-0.01	[-0.06, 0.04]	-0.48	1061.84	.628
ConditionEvil × Matched controlTRUE	-0.28	[-0.42, -0.15]	-4.22	12334.67	< .001
ConditionGood × Matched controlTRUE	-0.47	[-0.60, -0.34]	-7.02	12344.57	< .001
**Random Effects**					
σ^2	2.30				
$\tau_{00\ \text{Participant}}$	0.39				
$\tau_{00\ \text{Item}}$	0.51				
$N_{\text{Participant}}$	1,075				
N_{Item}	24				
Marginal R^2	0.288				
Conditional R^2	0.487				

Confidence

Table S68. **Multilevel Model for Confidence Ratings (All Items) for Study 3**

Term	$\hat{\beta}$	95% CI	t	df	p
Intercept	5.31	[5.04, 5.57]	39.19	135.45	< .001
ConditionEvil	0.19	[0.05, 0.33]	2.70	1433.50	.007
ConditionGood	0.19	[0.05, 0.32]	2.62	1423.76	.009
Matched controlTRUE	-0.25	[-0.48, -0.02]	-2.12	24.57	.044
Having Children	-0.03	[-0.16, 0.10]	-0.46	1061.86	.643
Gender simpleMale	-0.06	[-0.17, 0.05]	-1.09	1061.75	.274
Gender simpleOther	0.20	[-0.17, 0.57]	1.05	1062.09	.292
Political Ideology AllLiberal	0.35	[0.21, 0.48]	5.04	1062.24	< .001
Political Ideology AllConservative	0.14	[-0.03, 0.32]	1.60	1062.14	.109
COVID Vaccine Intentions	0.22	[0.06, 0.38]	2.65	1061.84	.008
Age scaled	0.06	[-0.01, 0.13]	1.78	1061.88	.075
Education scaled	-0.04	[-0.10, 0.02]	-1.39	1062.03	.164
News Checking scaled	0.12	[0.06, 0.18]	3.94	1062.03	< .001
Social Media Use scaled	0.01	[-0.04, 0.07]	0.51	1062.05	.609
ConditionEvil × Matched controlTRUE	-0.21	[-0.31, -0.11]	-4.25	12030.97	< .001
ConditionGood × Matched controlTRUE	-0.12	[-0.22, -0.03]	-2.50	12036.02	.012
**Random Effects**					
σ^2	1.22				
$\tau_{00\ \text{Participant}}$	0.68				
$\tau_{00\ \text{Item}}$	0.08				
$N_{\text{Participant}}$	1,075				
N_{Item}	24				
Marginal R^2	0.046				
Conditional R^2	0.412				

Sharing Intent

Table S69. Multilevel Model for Sharing Ratings (All Items) for Study 3

Term	$\hat{\beta}$	95% CI	t	df	p
Intercept	2.16	[1.74, 2.57]	10.18	68.37	$<$ .001
ConditionEvil	0.07	[-0.11, 0.26]	0.79	1380.01	.432
ConditionGood	-0.09	[-0.27, 0.10]	-0.92	1371.74	.358
Matched controlTRUE	0.92	[0.49, 1.35]	4.22	23.10	$<$ .001
Having Children	0.15	[-0.03, 0.32]	1.66	1061.69	.097
Gender simpleMale	0.11	[-0.04, 0.26]	1.42	1061.59	.156
Gender simpleOther	0.02	[-0.47, 0.52]	0.09	1061.90	.928
Political Ideology AllLiberal	-0.27	[-0.45, -0.09]	-2.97	1062.02	.003
Political Ideology AllConservative	-0.10	[-0.33, 0.14]	-0.81	1061.93	.419
COVID Vaccine Intentions	-0.13	[-0.35, 0.08]	-1.20	1061.67	.229
Age scaled	0.04	[-0.05, 0.13]	0.90	1061.71	.369
Education scaled	-0.01	[-0.08, 0.07]	-0.21	1061.84	.830
News Checking scaled	0.08	[0.00, 0.16]	2.02	1061.84	.044
Social Media Use scaled	0.06	[-0.02, 0.13]	1.45	1061.86	.146
ConditionEvil × Matched controlTRUE	0.21	[0.09, 0.33]	3.43	12000.37	$<$ .001
ConditionGood × Matched controlTRUE	0.57	[0.45, 0.69]	9.38	12004.70	$<$ .001
**Random Effects**					
σ^2	1.86				
$\tau_{00\ \text{Participant}}$	1.23				
$\tau_{00\ \text{Item}}$	0.28				
$N_{\text{Participant}}$	1,075				
N_{Item}	24				
Marginal R^2	0.109				
Conditional R^2	0.507				

Item-Level Results.

Bartlett test items

Table S70. Item-Level Bartlett Test of Homogeneity of Variances for Study 3: Manipulativeness

Variable	Bartlett's K-squared	df	p
Conspir.Database Manipulativeness	9.106	2	**0.011**
Conspir.Database.C Manipulativeness	0.789	2	0.674
Conspir.Leaders Manipulativeness	1.243	2	0.537
Conspir.Leaders.C Manipulativeness	0.465	2	0.793
Conspir.Greatergood Manipulativeness	14.819	2	**<0.001**
Conspir.Greatergood.C Manipulativeness	0.747	2	0.688
Expert.FluVaccine Manipulativeness	1.292	2	0.524
Expert.FluVaccine.C Manipulativeness	1.602	2	0.449
Expert.HPV Manipulativeness	0.380	2	0.827
Expert.HPV.C Manipulativeness	4.236	2	0.120
Expert.Autism Manipulativeness	23.330	2	**<0.001**
Expert.Autism.C Manipulativeness	1.578	2	0.454
Natural.Glyphosate Manipulativeness	3.003	2	0.223
Natural.Glyphosate.C Manipulativeness	2.754	2	0.252
Natural.Homeopathy Manipulativeness	2.638	2	0.267
Natural.Homeopathy.C Manipulativeness	1.370	2	0.504
Natural.Viruses Manipulativeness	2.428	2	0.297
Natural.Viruses.C Manipulativeness	1.147	2	0.563
Story.Multiple Manipulativeness	0.179	2	0.914
Story.Multiple.C Manipulativeness	4.256	2	0.119
Story.VaccineCourt Manipulativeness	1.893	2	0.388
Story.VaccineCourt.C Manipulativeness	0.065	2	0.968
Story.Hepatitis Manipulativeness	4.877	2	0.087
Story.Hepatitis.C Manipulativeness	9.469	2	**0.009**

Table S71. Item-Level Bartlett Test of Homogeneity of Variances for Study 3: Confidence

Variable	Bartlett's K-squared	*df*	*p*
Conspir.Database Confidence	2.421	2	0.298
Conspir.Database.C Confidence	1.089	2	0.580
Conspir.Leaders Confidence	1.162	2	0.559
Conspir.Leaders.C Confidence	1.193	2	0.551
Conspir.Greatergood Confidence	6.438	2	**0.040**
Conspir.Greatergood.C Confidence	4.125	2	0.127
Expert.FluVaccine Confidence	4.056	2	0.132
Expert.FluVaccine.C Confidence	3.248	2	0.197
Expert.HPV Confidence	1.285	2	0.526
Expert.HPV.C Confidence	0.164	2	0.921
Expert.Autism Confidence	5.440	2	0.066
Expert.Autism.C Confidence	0.182	2	0.913
Natural.Glyphosate Confidence	1.715	2	0.424
Natural.Glyphosate.C Confidence	0.401	2	0.818
Natural.Homeopathy Confidence	1.848	2	0.397
Natural.Homeopathy.C Confidence	12.291	2	**0.002**
Natural.Viruses Confidence	3.554	2	0.169
Natural.Viruses.C Confidence	7.663	2	**0.022**
Story.Multiple Confidence	9.165	2	**0.010**
Story.Multiple.C Confidence	3.419	2	0.181
Story.VaccineCourt Confidence	3.231	2	0.199
Story.VaccineCourt.C Confidence	5.404	2	0.067
Story.Hepatitis Confidence	5.629	2	0.060
Story.Hepatitis.C Confidence	9.237	2	**0.010**

Table S72. Item-Level Bartlett Test of Homogeneity of Variances for Study 3: Sharing

Variable	Bartlett's K-squared	*df*	*p*
Conspir.Database Sharing	9.614	2	**0.008**
Conspir.Database.C Sharing	0.875	2	0.646
Conspir.Leaders Sharing	6.393	2	**0.041**
Conspir.Leaders.C Sharing	0.733	2	0.693
Conspir.Greatergood Sharing	3.695	2	0.158
Conspir.Greatergood.C Sharing	1.078	2	0.583
Expert.FluVaccine Sharing	5.766	2	0.056
Expert.FluVaccine.C Sharing	1.931	2	0.381
Expert.HPV Sharing	3.459	2	0.177
Expert.HPV.C Sharing	0.014	2	0.993
Expert.Autism Sharing	15.374	2	**<0.001**
Expert.Autism.C Sharing	0.165	2	0.921
Natural.Glyphosate Sharing	16.011	2	**<0.001**
Natural.Glyphosate.C Sharing	0.969	2	0.616
Natural.Homeopathy Sharing	8.610	2	**0.014**
Natural.Homeopathy.C Sharing	5.532	2	0.063
Natural.Viruses Sharing	4.639	2	0.098
Natural.Viruses.C Sharing	0.298	2	0.862
Story.Multiple Sharing	12.477	2	**0.002**
Story.Multiple.C Sharing	0.586	2	0.746
Story.VaccineCourt Sharing	3.881	2	0.144
Story.VaccineCourt.C Sharing	0.196	2	0.907
Story.Hepatitis Sharing	7.099	2	**0.029**
Story.Hepatitis.C Sharing	0.134	2	0.935

One-Way ANOVA items

Table S73. Item-Level One-Way ANOVA (Welch) for Study 3: Manipulativeness

Variable	F	df1	df2	p
Conspir.Database Manipulativeness	2.900	2	355.613	0.056
Conspir.Database.C Manipulativeness	1.701	2	352.551	0.184
Conspir.Leaders Manipulativeness	0.977	2	356.226	0.378
Conspir.Leaders.C Manipulativeness	0.831	2	356.474	0.437
Conspir.Greatergood Manipulativeness	3.977	2	358.525	**0.020**
Conspir.Greatergood.C Manipulativeness	1.629	2	353.391	0.198
Expert.FluVaccine Manipulativeness	2.519	2	357.209	0.082
Expert.FluVaccine.C Manipulativeness	2.396	2	354.572	0.093
Expert.HPV Manipulativeness	5.425	2	356.624	**0.005**
Expert.HPV.C Manipulativeness	2.226	2	350.332	0.110
Expert.Autism Manipulativeness	4.809	2	355.729	**0.009**
Expert.Autism.C Manipulativeness	2.935	2	357.561	0.054
Natural.Glyphosate Manipulativeness	0.359	2	359.229	0.698
Natural.Glyphosate.C Manipulativeness	6.934	2	350.056	**0.001**
Natural.Homeopathy Manipulativeness	6.567	2	357.651	**0.002**
Natural.Homeopathy.C Manipulativeness	0.578	2	349.805	0.562
Natural.Viruses Manipulativeness	12.797	2	357.834	**<0.001**
Natural.Viruses.C Manipulativeness	2.865	2	353.514	0.058
Story.Multiple Manipulativeness	0.728	2	350.011	0.484
Story.Multiple.C Manipulativeness	3.426	2	361.930	**0.034**
Story.VaccineCourt Manipulativeness	3.242	2	354.362	**0.040**
Story.VaccineCourt.C Manipulativeness	0.459	2	358.130	0.632
Story.Hepatitis Manipulativeness	10.108	2	352.475	**<0.001**
Story.Hepatitis.C Manipulativeness	1.225	2	359.126	0.295

Table S74. Item-Level One-Way ANOVA (Welch) for Study 3: Confidence

Variable	F	df1	df2	p
Conspir.Database Confidence	1.503	2	356.755	0.224
Conspir.Database.C Confidence	0.040	2	352.471	0.961
Conspir.Leaders Confidence	0.225	2	353.676	0.799
Conspir.Leaders.C Confidence	0.650	2	356.582	0.523
Conspir.Greatergood Confidence	1.803	2	359.433	0.166
Conspir.Greatergood.C Confidence	0.974	2	349.550	0.378
Expert.FluVaccine Confidence	5.648	2	357.705	**0.004**
Expert.FluVaccine.C Confidence	0.246	2	356.298	0.782
Expert.HPV Confidence	3.358	2	357.752	**0.036**
Expert.HPV.C Confidence	0.434	2	351.067	0.648
Expert.Autism Confidence	0.906	2	354.390	0.405
Expert.Autism.C Confidence	1.015	2	358.563	0.363
Natural.Glyphosate Confidence	0.001	2	358.809	0.999
Natural.Glyphosate.C Confidence	3.950	2	354.652	**0.020**
Natural.Homeopathy Confidence	1.827	2	357.904	0.162
Natural.Homeopathy.C Confidence	1.744	2	343.643	0.176
Natural.Viruses Confidence	4.503	2	355.452	**0.012**
Natural.Viruses.C Confidence	2.097	2	353.950	0.124
Story.Multiple Confidence	3.159	2	346.359	**0.044**
Story.Multiple.C Confidence	0.610	2	360.839	0.544
Story.VaccineCourt Confidence	0.216	2	348.073	0.806
Story.VaccineCourt.C Confidence	1.617	2	357.159	0.200
Story.Hepatitis Confidence	5.671	2	352.998	**0.004**
Story.Hepatitis.C Confidence	2.875	2	352.654	0.058

Table S75. **Item-Level One-Way ANOVA (Welch) for Study 3: Sharing**

Variable	F	df1	df2	p
Conspir.Database Sharing	1.485	2	351.811	0.228
Conspir.Database.C Sharing	0.598	2	352.464	0.550
Conspir.Leaders Sharing	1.464	2	350.910	0.233
Conspir.Leaders.C Sharing	4.980	2	354.328	**0.007**
Conspir.Greatergood Sharing	0.846	2	354.857	0.430
Conspir.Greatergood.C Sharing	4.258	2	353.655	**0.015**
Expert.FluVaccine Sharing	1.746	2	356.784	0.176
Expert.FluVaccine.C Sharing	5.461	2	352.186	**0.005**
Expert.HPV Sharing	0.060	2	351.896	0.942
Expert.HPV.C Sharing	1.502	2	350.500	0.224
Expert.Autism Sharing	4.501	2	349.509	**0.012**
Expert.Autism.C Sharing	8.001	2	359.083	**<0.001**
Natural.Glyphosate Sharing	1.643	2	352.226	0.195
Natural.Glyphosate.C Sharing	1.577	2	353.188	0.208
Natural.Homeopathy Sharing	1.834	2	354.145	0.161
Natural.Homeopathy.C Sharing	8.768	2	345.405	**<0.001**
Natural.Viruses Sharing	1.111	2	356.748	0.330
Natural.Viruses.C Sharing	1.962	2	353.124	0.142
Story.Multiple Sharing	3.325	2	344.056	**0.037**
Story.Multiple.C Sharing	7.453	2	358.727	**<0.001**
Story.VaccineCourt Sharing	0.183	2	348.936	0.833
Story.VaccineCourt.C Sharing	0.151	2	357.521	0.860
Story.Hepatitis Sharing	2.113	2	347.621	0.122
Story.Hepatitis.C Sharing	8.180	2	354.097	**<0.001**

References

1. Sara Balduzzi, Gerta Rücker, and Guido Schwarzer. How to perform a meta-analysis with R: a practical tutorial. *BMJ Ment Health*, 22(4):153–160, 2019. ISSN 1362-0347. .
2. Gerta Rücker, Ulrike Krahn, Jochem König, Orestis Efthimiou, Annabel Davies, Theodoros Papakonstantinou, and Guido Schwarzer. *netmeta: Network Meta-Analysis using Frequentist Methods*, 2022. URL https://CRAN.R-project.org/package=netmeta. R package version 2.1-0.
3. William J. McGuire. Inducing Resistance to Persuasion: Some Contemporary Approaches. *Advances in Experimental Social Psychology*, 1(C):191–229, 1964. ISSN 00652601. .
4. D. Papageorgis and W. J. McGuire. The generality of immunity to persuasion produced by pre-exposure to weakened counterarguments. *Journal of Abnormal and Social Psychology*, 62(3):475–481, 1961. ISSN 0096851X. .
5. Josh Compton and Michael Pfau. Inoculation Theory of Resistance to Influence at Maturity: Recent Progress In Theory Development and Application and Suggestions for Future Research. *Annals of the International Communication Association*, 29(1):97–146, 2005. ISSN 2380-8985. .
6. Josh Compton and Bobi Ivanov. Vaccinating Voters: Surveying Political Campaign Inoculation Scholarship. *Annals of the International Communication Association*, 37(1):251–283, 2013. ISSN 2380-8985. .
7. John A. Banas and Stephen A. Rains. A meta-analysis of research on inoculation theory. *Communication Monographs*, 77 (3):281–311, 2010. ISSN 03637751. .
8. Cecilie S. Traberg, Jon Roozenbeek, and Sander van der Linden. Psychological Inoculation against Misinformation: Current Evidence and Future Directions. *The ANNALS of the American Academy of Political and Social Science*, 700(1): 136–151, 2022. .
9. Josh Compton. Prophylactic Versus Therapeutic Inoculation Treatments for Resistance to Influence. *Communication Theory*, 30(3):330–343, 2020. ISSN 1050-3293. .
10. Daniel Jolley and Karen M. Douglas. Prevention is better than cure: Addressing anti-vaccine conspiracy theories. *Journal of Applied Social Psychology*, 47(8):459–469, 2017. ISSN 15591816. .
11. Josh Compton. Inoculation Theory. In James Price Dillard and Lijiang Shen, editors, *The SAGE Handbook of Persuasion*, chapter 14, pages 220–236. SAGE Publications Inc., Thousand Oaks, CA, second edition, 2013. .
12. Rakoen Maertens, Jon Roozenbeek, Melisa Basol, and Sander van der Linden. Long-Term Effectiveness of Inoculation Against Misinformation: Three Longitudinal Experiments. *Journal of Experimental Psychology: Applied*, 27, 2021. ISSN 1076898X. .
13. Melisa Basol, Jon Roozenbeek, Manon Berriche, Fatih Uenal, William P. McClanahan, and Sander van der Linden. Towards psychological herd immunity: Cross-cultural evidence for two prebunking interventions against COVID-19 misinformation. *Big Data and Society*, 8(1), 2021. ISSN 20539517. .
14. Sander van der Linden, Anthony Leiserowitz, Seth Rosenthal, and Edward Maibach. Inoculating the Public against Misinformation about Climate Change. *Global Challenges*, 1(2):1600008, 2017. ISSN 2056-6646. .
15. Jon Roozenbeek and Sander van der Linden. The fake news game: actively inoculating against the risk of misinformation. *Journal of Risk Research*, 22(5):570–580, 2019. ISSN 14664461. .
16. Sandra J. Bean. Emerging and continuing trends in vaccine opposition website content. *Vaccine*, 29(10):1874–1880, 2011. ISSN 0264410X. . URL http://dx.doi.org/10.1016/j.vaccine.2011.01.003.
17. Anna Kata. A postmodern Pandora's box: Anti-vaccination misinformation on the Internet. *Vaccine*, 28(7):1709–1716, 2010. ISSN 0264410X. .
18. Anna Kata. Anti-vaccine activists, Web 2.0, and the postmodern paradigm - An overview of tactics and tropes used online by the anti-vaccination movement. *Vaccine*, 30(25):3778–3789, 2012. ISSN 0264410X. . URL http://dx.doi.org/10.1016/j.vaccine.2011.11.112.
19. Wen Ying Sylvia Chou and Alexandra Budenz. Considering Emotion in COVID-19 Vaccine Communication: Addressing Vaccine Hesitancy and Fostering Vaccine Confidence. *Health Communication*, 35(14):1718–1722, 2020. ISSN 15327027. . URL https://doi.org/10.1080/10410236.2020.1838096.
20. Martin G. Myers and Diego Pineda. Misinformation about Vaccines. In Alan D.T. Barrett and Lawrence R. Stanberry, editors, *Vaccines for Biodefense and Emerging and Neglected Diseases*, chapter 17, pages 255–270. Academic Press, London, 2009. ISBN 978-0-12-369408-9. .
21. David A. Broniatowski, Amelia M. Jamison, Si Hua Qi, Lulwah AlKulaib, Tao Chen, Adrian Benton, Sandra C. Quinn, and Mark Dredze. Weaponized health communication: Twitter bots and Russian trolls amplify the vaccine debate. *American Journal of Public Health*, 108(10):1378–1384, 2018. ISSN 15410048. .
22. Wen Ying Sylvia Chou, Anna Gaysynsky, and Joseph N. Cappella. Where we go from here: Health misinformation on social media. *American Journal of Public Health*, 110(S3):S273–S275, 2020. ISSN 15410048. .
23. Matthew J. Hornsey, Emily A. Harris, and Kelly S. Fielding. The psychological roots of anti-vaccination attitudes: A 24-nation investigation. *Health Psychology*, 37(4):307–315, 2018. ISSN 19307810. .
